# Network Analysis of Fine Particulate Matter (PM_2.5_) Emissions in China

**DOI:** 10.1038/srep33227

**Published:** 2016-09-09

**Authors:** Shaomin Yan, Guang Wu

**Affiliations:** 1 Bioscience and Technology Research Center, Guangxi Academy of Sciences, 98 Daling Road, Nanning, Guangxi, 530007, China

## Abstract

Specification of PM_2.5_ spatial and temporal characteristics is important for understanding PM_2.5_ adverse effects and policymaking. We applied network analysis to studying the dataset MIX, which contains PM_2.5_ emissions recorded from 2168 monitoring stations in China in 2008 and 2010. The results showed that for PM_2.5_ emissions from industrial sector 8 clusters were found in 2008 but they merged together into a huge cluster in 2010, suggesting that industrial sector underwent an integrating process. For PM_2.5_ emissions from electricity generation sector, strong locality of clusters was revealed, implying that each region had its own electricity generation system. For PM_2.5_ emissions from residential sector, the same pattern of 10 clusters was uncovered in both years, implicating the household energy consumption unchanged from 2008 to 2010. For PM_2.5_ emissions from transportation sector, the same pattern of 5 clusters with many connections in-between was unraveled, indicating the high-speed development of transportation nationalwidely. Except for the known elements, mercury (Hg) surfaced as an element for particle nucleation. To our knowledge, this is the first network study in this field.

Identification of PM_2.5_ sources, elucidation of mechanism of PM_2.5_ formation, specification of PM_2.5_ spatial and temporal characteristics, enumeration of affecting factors on PM_2.5_ pollution, and determination of PM_2.5_ adverse effects on humans are important tasks for making the policies to eradicate PM_2.5_ pollution.

PM_2.5_ sources are identified as primary emission and secondary formation of aerosols^1^. The former mainly comes from combustion and dust^2^ whereas the latter involves a series of chemical processes^3^. Accordingly, the emission from residential sector can be considered as primary emission because household heating is a major source of combustion in northern China in winter^4^, and burning of biomass either for cooking or for agriculture purpose also constitutes a major source of combustion in rural area in China^5^,^6^,^7^. Indeed, regional biomass burning emission may be sometimes an important contributor in both urban and rural areas in China^8^,^9^. On the other hand, emissions from electricity generation, industrial and transportation sectors can be viewed as secondary formation of aerosols because coal burning generates sulfur dioxide, and vehicles generate volatile organic compounds and nitrogen oxide^4^, besides the emissions of primary aerosols from these sources^10^,^11^. Yet, a little amount of emission from recreation activities, e.g. barbecuing, also contributes to secondary formation of aerosols because of PM_10_ and PM_2.5_ emissions from barbecuing^7^.

Mechanism of PM_2.5_ formation is elucidated as that aerosol nucleation is the initial step for particle size and mass evolution to grow to PM_2.5_^12^,^13^. Although the size of nucleation-mode particles may be smaller than 30 nm^1^, PM_2.5_ eventually contains various components, such as elemental carbon (EC), potassium (K), sulfur (S), iron (Fe), silicon (Si), aluminum (Al), zinc (Zn), calcium (Ca), titanium (Ti)^14^, water-soluble ions (Cl^−^, SO_4_^2−^, NO_3_^−^, NH_4_^+^)^15^, and organic carbon. Thus, contribution from organic compounds to PM_2.5_ cannot be ignored, for example, 80% polycyclic aromatic hydrocarbons generated by combustion join PM_2.5_^16^ and xylenes, propane, n-pentane, 2,3-dimethylbutane, 2-methylpentane, propene, benzene and toluene are found in PM_2.5_^17^. Prior to aerosol nucleation, Al_2_O_3_^18^, Fe_2_O_3_ and MgO^19^ serve as seeds for conversion of SO_2_ to sulfate on dust surface^20^, leading to the production of H_2_SO_4_, a well-known precursor for particle nucleation^21^. Thereafter, a particle grows at about 40 nm per day when PM_2.5_ is less than 50 μg m^−3^, but about 190 nm per day when PM_2.5_ is larger than 300 μg m^−3^ in Beijing^1^.

PM_2.5_ spatial and temporal characteristics have been studied in great details in China because a national monitoring network had been established in China^22^ with strong emphases on heavily populated cities such as Beijing^23^ and Tianjin^24^ because heavily populated areas in China are often associated with heavy pollution^25^,^26^. Regionally, the levels of PM_2.5_, PM_10_, CO and SO_2_ are higher in the Northern China than in the Western and the Southeastern China^27^. Seasonally, the general pattern of PM_2.5_ emission is winter > spring > autumn and summer^28^ or winter > autumn > spring > summer^29^. Combination of locality and seasonality shows that pollution events commonly occur during the fall in the Southeastern China and during the spring in the Western China^23^. For Beijing, the events of formation of particles were counted for 50%, 20%, 35% and 45% of the measurement days in spring, summer, autumn and winter^4^.

The factors that influence PM_2.5_ pollution are many, among them meteorological influence is important. First of all, winds often have good correlations with PM_2.5_ episode^1^, so the Asian summer monsoon and tropical cyclones wash a significant portion of pollutants out from Eastern China and South Korea^30^ whereas the Asian winter monsoon pushes the severely polluted air to Yangtze River Delta from the Northern China^31^, and decreases O_3_ level in heavily polluted New Delhi, India^32^. Naturally, a good correlation was observed between pollutants and precipitation^29^ since lightning generates NO_x_ during rainfall^33^.

PM_2.5_ adverse effects on humans have been studied in various diseases^29^,^34^,^35^,^36^,^37^,^38^,^39^,^40^, because PM_2.5_ contains various elemental components, which are significant in PM_2.5_ toxicity^41^,^42^,^43^. Moreover, toxicity of particulate matter is suggested to be more directly related to particle surface area rather than to its mass^44^,^45^. Although PM_2.5_ prevails in urban areas, it does not mean that rural area is somewhat free from PM_2.5_ adverse effects because mortality is highly associated with smog episodes in rural counties^46^.

In studying PM_2.5_ spatial and temporal characteristics, it is important to get a whole picture because studies often focus on very visible places with political, economic, financial and industrial importance. The recent-complied dataset MIX, the mosaic Asian anthropogenic emission inventory for 2008 and 2010, documented the emissions from 40.125 E to 179.875 E and from 20.125 S to 89.875 N in 0.25°× 0.25° (∼25 km × 25 km) grid^47^. This dataset includes 2168 monitoring stations in China that collect monthly emission data in terms of SO_2_, NO_x_, CO, NH_3_, PM_10_, PM_2.5_, BC, OC, CO_2_, CB05, and SAPRC-99 with respect to industrial, electricity generation, residential, transportation and agricultural sectors^47^.

For any observed and complied datasets, interpretation can be made from different angles at different levels to produce various types of information with different methods. And no single method is perfect and can replace other existent methods. Methodologically, to the best of our knowledge, network analysis has not been used in the studies on PM_2.5_ emission although various methods have so far been employed. Network analysis is a powerful tool to deal with large-scale datasets and is potentially useful to PM_2.5_ research. Network analysis studies relationships between objects, which are graphically presented as nodes while a relationship between two nodes is graphically presented as an edge connecting two nodes. To date, network analysis has been applied to many research fields, for example, a relationship can be a friendship between two persons in social network; a relationship can be a road between two places in transportation network; a relationship can be chemical binding between two proteins in protein interaction network. Thus, the obstacle preventing network analysis from studying PM_2.5_ emission is how to define a relationship between two nodes because a node can be easily defined as PM_2.5_ emission recorded in a monitoring station. In this study, we define that two nodes have a relationship when a good correlation exists between two PM_2.5_ emission profiles recorded in two monitoring stations because PM_2.5_ emissions were recorded along the time course. In other words, it means that two PM_2.5_ emission profiles have similarity along the time course. Indeed, similarity was observed in CO and O_3_ emission profiles^30^, and in water-soluble ion concentrations in PM_2.5_ between Shijiazhuang, Beijing and Tianjin^15^. Essentially, similarity is attributed to various reasons, for example, about 10% of PM_2.5_ in Beijing is constituted from mineral dust^18^, which is frequently transported from Gobi desert to Beijing and North China Plain^48^,^49^,^50^. As a matter of fact, the use of correlation between two time series to build a relationship has already been widely used in the analysis of gene co-expression because genes with similar expression profiles are more likely to encode interacting proteins, to have a similar biological function, and to belong to the same biological pathway^51^. A good example of using network analysis would be transportation system, where the network can easily and visibly show how traffic congestion forms and helps to design better solution. As PM_2.5_ is closely associated with emissions from transportation system, network analysis could potentially be helpful in this regard.

Formation of new particles is generally considered as a regional event, especially when the movement of air mass is smaller than 50 km per day, then regional transport of PM_2.5_ is negligible^1^. Consequently, network analysis appears more attractive, not only it brings attention to less visible sites which could play an important role but have less political, economic and commercial interests, but also such a systematic approach is very helpful for policymaking because network analysis can group nodes into clusters where there are more connections between the nodes within a cluster than between the nodes in different clusters, so a policy could be made with respect to particular clusters.

Thus the aim of this study was designed to use network analysis to study PM_2.5_ emissions from industrial, electricity generation, residential, and transportation sectors in China for 2008 and 2010.

## Results and Discussion

**Figure 1:**
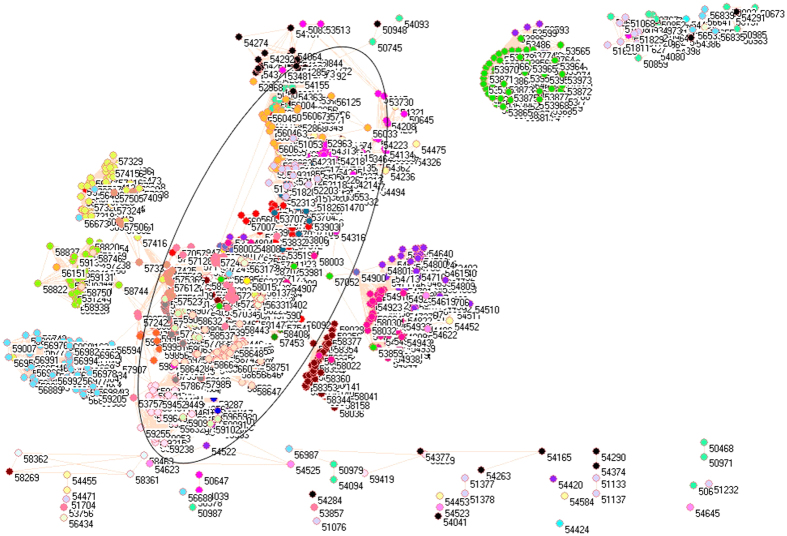
Network of PM_2.5_ emissions from industrial sector in China monitored by 1744 stations in 2008. Yellow circles are 57 monitoring stations in Anhui, cyan circles are 11 monitoring stations in Beijing, lime green circles are 48 monitoring stations in Fujian, red circles are 68 monitoring stations in Gansu, blue circles are 64 monitoring stations in Guangdong, pink circles are 60 monitoring stations in Guangxi, white circles are 63 monitoring stations in Guizhou, orange circles are 12 monitoring stations in Hainan, purple circles are 98 monitoring stations in Hebei, cadet blue circles are 81 monitoring stations in Henan, teal blue circles are 79 monitoring stations in Heilongjiang, olive green circles are 60 monitoring stations in Hubei, gray circles are 69 monitoring stations in Hunan, black circles are 48 monitoring stations in Jilin, maroon circles are 56 monitoring stations in Jiangsu, light green circles are 67 monitoring stations in Jiangxi, light yellow circles are 42 monitoring stations in Liaoning, magenta circles are 94 monitoring stations in Inner Mongolia, midnight blue circles are 18 monitoring stations in Ningxia, dandelion circles are 33 monitoring stations in Qinghai, wild strawberry circles are 77 monitoring stations in Shandong, forest green circles are 77 monitoring stations in Shanxi, salmon circles are 67 monitoring stations in Shaanxi, light sky blue circles are 4 monitoring stations in Shanghai, green yellow circles are 116 monitoring stations in Sichuan, lavender circles are 6 monitoring stations in Tianjin, light faded green circles are 21 monitoring stations in Tibet, light purple circles are 66 monitoring stations in Xinjiang, corn flower blue circles are 98 monitoring stations in Yunnan, light orange circles are 64 monitoring stations in Zhejiang, and tan circles are 27 monitoring stations in Chongqing.

**Figure 2:**
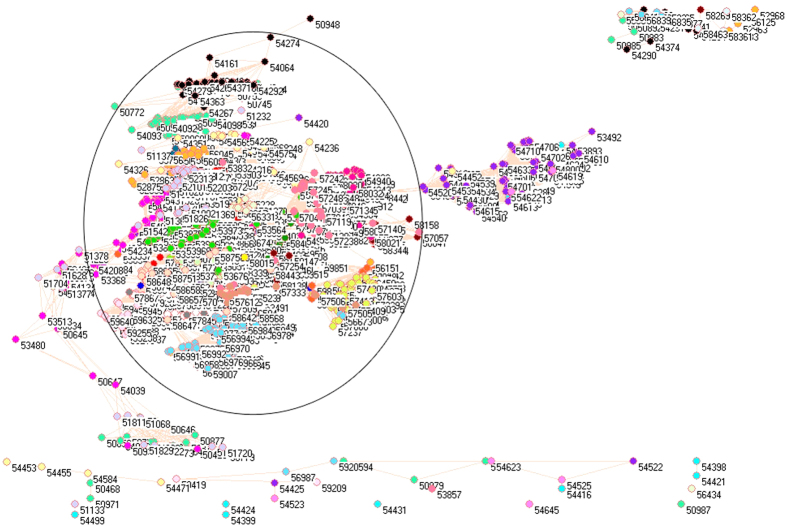
Network of PM_2.5_ emissions from industrial sector in China monitored by 1744 stations in 2010.

**Figure 3:**
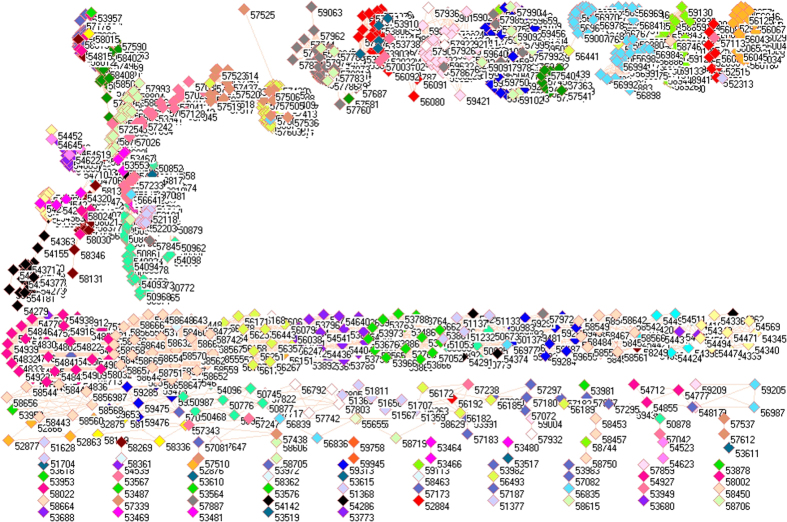
Network of PM_2.5_ emissions from electricity generation sector in China monitored by 1558 stations in 2008. Yellow diamonds are 21 monitoring stations in Anhui, cyan diamonds are 11 monitoring stations in Beijing, lime green diamonds are 48 monitoring stations in Fujian, red diamonds are 68 monitoring stations in Gansu, blue diamonds are 64 monitoring stations in Guangdong, pink diamonds are 60 monitoring stations in Guangxi, white diamonds are 17 monitoring stations in Guizhou, orange diamonds are 12 monitoring stations in Hainan, purple diamonds are 96 monitoring stations in Hebei, cadet blue diamonds are 80 monitoring stations in Henan, teal blue diamonds are 79 monitoring stations in Heilongjiang, olive green diamonds are 60 monitoring stations in Hubei, gray diamonds are 69 monitoring stations in Hunan, black diamonds are 48 monitoring stations in Jilin, maroon diamonds are 55 monitoring stations in Jiangsu, light green diamonds are 67 monitoring stations in Jiangxi, light yellow diamonds are 42 monitoring stations in Liaoning, magenta diamonds are 95 monitoring stations in Inner Mongolia, midnight blue diamonds are 18 monitoring stations in Ningxia, dandelion diamonds are 34 monitoring stations in Qinghai, wild straw berry diamonds are 46 monitoring stations in Shandong, forest green diamonds are 33 monitoring stations in Shanxi, salmon diamonds are 67 monitoring stations in Shaanxi, light sky blue diamonds are 4 monitoring stations in Shanghai, green yellow diamonds are 114 monitoring stations in Sichuan, lavender diamonds are 5 monitoring stations in Tianjin, light faded green diamond is 1 monitoring station in Tibet, light purple diamonds are 66 monitoring stations in Xinjiang, corn flower blue diamonds are 97 monitoring stations in Yunnan, light orange diamonds are 54 monitoring stations in Zhejiang, and tan diamonds are 27 monitoring stations in Chongqing.

**Figure 4:**
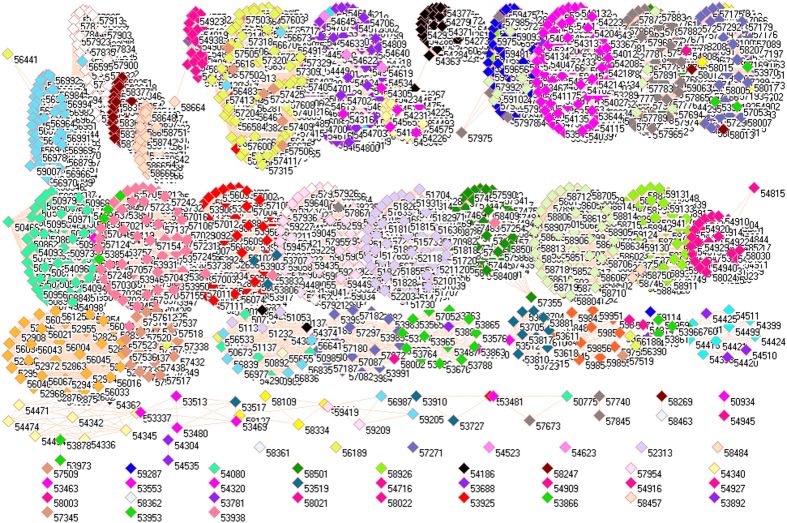
Network of PM_2.5_ emissions from electricity generation sector in China monitored by 1636 stations in 2010. Yellow diamonds are 21 monitoring stations in Anhui, cyan diamonds are 11 monitoring stations in Beijing, lime green diamonds are 48 monitoring stations in Fujian, red diamonds are 68 monitoring stations in Gansu, blue diamonds are 64 monitoring stations in Guangdong, pink diamonds are 60 monitoring stations in Guangxi, white diamonds are 63 monitoring stations in Guizhou, orange diamonds are 12 monitoring stations in Hainan, purple diamonds are 96 monitoring stations in Hebei, cadet blue diamonds are 80 monitoring stations in Henan, teal blue diamonds are 79 monitoring stations in Heilongjiang, olive green diamonds are 60 monitoring stations in Hubei, gray diamonds are 69 monitoring stations in Hunan, black diamonds are 48 monitoring stations in Jilin, maroon diamonds are 55 monitoring stations in Jiangsu, light green diamonds are 67 monitoring stations in Jiangxi, light yellow diamonds are 42 monitoring stations in Liaoning, magenta diamonds are 95 monitoring stations in Inner Mongolia, midnight blue diamonds are 18 monitoring stations in Ningxia, dandelion diamonds are 34 monitoring stations in Qinghai, wild straw berry diamonds are 77 monitoring stations in Shandong, forest green diamonds are 33 monitoring stations in Shanxi, salmon diamonds are 67 monitoring stations in Shaanxi, light sky blue diamonds are 4 monitoring stations in Shanghai, green yellow diamonds are 114 monitoring stations in Sichuan, lavender diamonds are 5 monitoring stations in Tianjin, light faded green diamond is 1 monitoring station in Tibet, light purple diamonds are 66 monitoring stations in Xinjiang, corn flower blue diamonds are 98 monitoring stations in Yunnan, light orange diamonds are 54 monitoring stations in Zhejiang, and tan diamonds are 27 monitoring stations in Chongqing.

**Figure 5:**
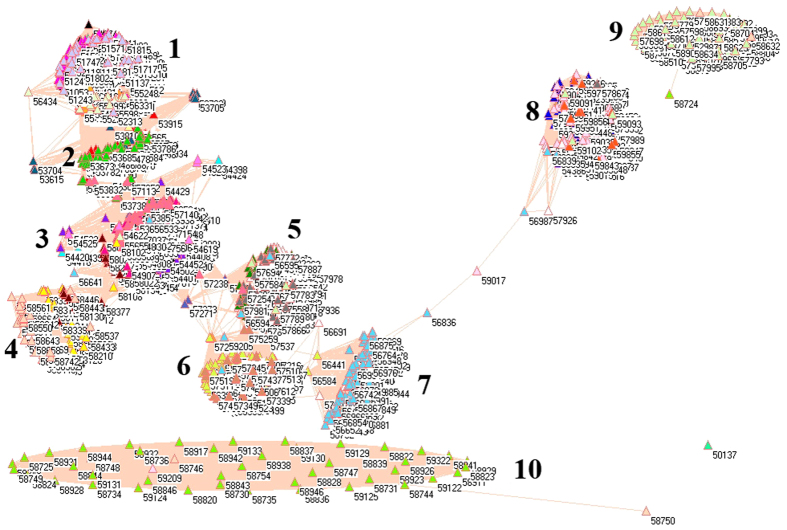
Network of PM_2.5_ emissions from residential sector in China monitored by 1742 stations in 2008. Yellow triangles are 57 monitoring stations in Anhui, cyan triangles are 11 monitoring stations in Beijing, lime green triangles are 48 monitoring stations in Fujian, red triangles are 68 monitoring stations in Gansu, blue triangles are 64 monitoring stations in Guangdong, pink triangles are 60 monitoring stations in Guangxi, white triangles are 63 monitoring stations in Guizhou, orange triangles are 12 monitoring stations in Hainan, purple triangles are 98 monitoring stations in Hebei, cadet blue triangles are 81 monitoring stations in Henan, teal blue triangles are 79 monitoring stations in Heilongjiang, olive green triangles are 60 monitoring stations in Hubei, gray triangles are 69 monitoring stations in Hunan, black triangles are 48 monitoring stations in Jilin, maroon triangles are 56 monitoring stations in Jiangsu, light green triangles are 67 monitoring stations in Jiangxi, light yellow triangles are 42 monitoring stations in Liaoning, magenta triangles are 94 monitoring stations in Inner Mongolia, midnight blue triangles are 18 monitoring stations in Ningxia, dandelion triangles are 33 monitoring stations in Qinghai, wild straw berry triangles are 77 monitoring stations in Shandong, forest green triangles are 77 monitoring stations in Shanxi, salmon triangles are 67 monitoring stations in Shaanxi, light sky blue triangles are 4 monitoring stations in Shanghai, green yellow triangles are 116 monitoring stations in Sichuan, lavender triangles are 6 monitoring stations in Tianjin, light faded green triangles are 22 monitoring stations in Tibet, light purple triangles are 66 monitoring stations in Xinjiang, corn flower blue triangles are 98 monitoring stations in Yunnan, light orange triangles are 54 monitoring stations in Zhejiang, and tan triangles are 27 monitoring stations in Chongqing.

**Figure 6:**
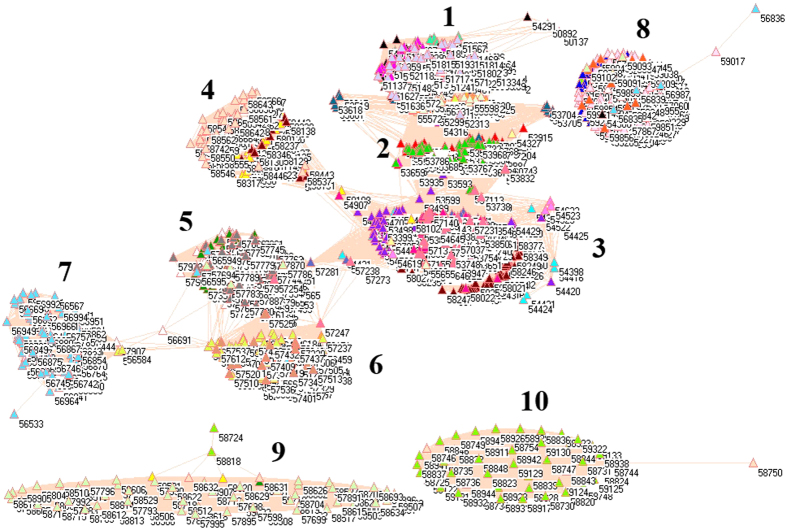
Network of PM_2.5_ emissions from residential sector in China monitored by 1743 stations in 2010. Yellow triangles are 57 monitoring stations in Anhui, cyan triangles are 11 monitoring stations in Beijing, lime green triangles are 48 monitoring stations in Fujian, red triangles are 68 monitoring stations in Gansu, blue triangles are 64 monitoring stations in Guangdong, pink triangles are 60 monitoring stations in Guangxi, white triangles are 63 monitoring stations in Guizhou, orange triangles are 12 monitoring stations in Hainan, purple triangles are 98 monitoring stations in Hebei, cadet blue triangles are 81 monitoring stations in Henan, teal blue triangles are 79 monitoring stations in Heilongjiang, olive green triangles are 60 monitoring stations in Hubei, gray triangles are 69 monitoring stations in Hunan, black triangles are 48 monitoring stations in Jilin, maroon triangles are 56 monitoring stations in Jiangsu, light green triangles are 67 monitoring stations in Jiangxi, light yellow triangles are 42 monitoring stations in Liaoning, magenta triangles are 95 monitoring stations in Inner Mongolia, midnight blue triangles are 18 monitoring stations in Ningxia, dandelion triangles are 33 monitoring stations in Qinghai, wild straw berry triangles are 77 monitoring stations in Shandong, forest green triangles are 77 monitoring stations in Shanxi, salmon triangles are 67 monitoring stations in Shaanxi, light sky blue triangles are 4 monitoring stations in Shanghai, green yellow triangles are 116 monitoring stations in Sichuan, lavender triangles are 6 monitoring stations in Tianjin, light faded green triangles are 22 monitoring stations in Tibet, light purple triangles are 66 monitoring stations in Xinjiang, corn flower blue triangles are 98 monitoring stations in Yunnan, light orange triangles are 54 monitoring stations in Zhejiang, and tan triangles are 27 monitoring stations in Chongqing.

**Figure 7:**
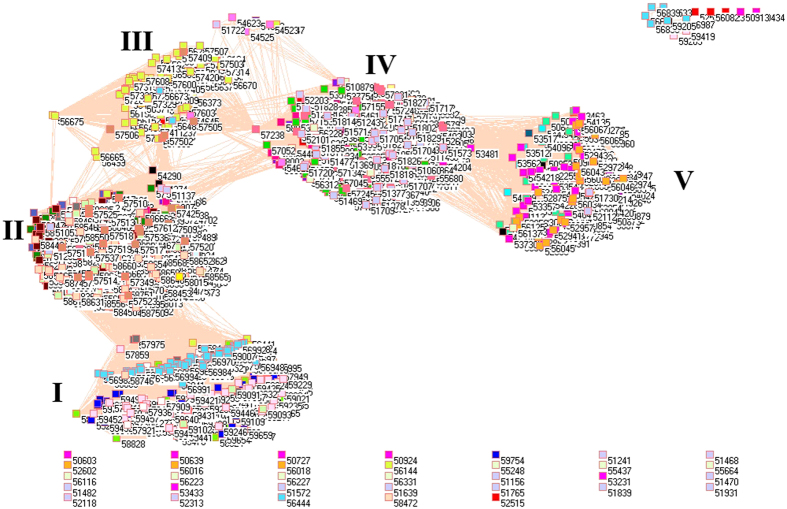
Network of PM_2.5_ emissions from transportation sector in China monitored by 1744 stations in 2008. Yellow squares are 57 monitoring stations in Anhui, cyan squares are 11 monitoring stations in Beijing, lime green squares are 48 monitoring stations in Fujian, red squares are 68 monitoring stations in Gansu, blue squares are 64 monitoring stations in Guangdong, pink squares are 60 monitoring stations in Guangxi, white squares are 63 monitoring stations in Guizhou, orange squares are 12 monitoring stations in Hainan, purple squares are 98 monitoring stations in Hebei, cadet blue squares are 81 monitoring stations in Henan, teal blue squares are 79 monitoring stations in Heilongjiang, olive green squares are 60 monitoring stations in Hubei, gray squares are 69 monitoring stations in Hunan, black squares are 48 monitoring stations in Jilin, maroon squares are 56 monitoring stations in Jiangsu, light green squares are 67 monitoring stations in Jiangxi, light yellow squares are 42 monitoring stations in Liaoning, magenta squares are 95 monitoring stations in Inner Mongolia, midnight blue squares are 18 monitoring stations in Ningxia, dandelion squares are 34 monitoring stations in Qinghai, wild straw berry squares are 77 monitoring stations in Shandong, forest green squares are 77 monitoring stations in Shanxi, salmon squares are 67 monitoring stations in Shaanxi, light sky blue squares are 4 monitoring stations in Shanghai, green yellow squares are 116 monitoring stations in Sichuan, lavender squares are 6 monitoring stations in Tianjin, light faded green squares are 22 monitoring stations in Tibet, light purple squares are 66 monitoring stations in Xinjiang, corn flower blue squares are 98 monitoring stations in Yunnan, light orange squares are 54 monitoring stations in Zhejiang, and tan squares are 27 monitoring stations in Chongqing.

**Figure 8:**
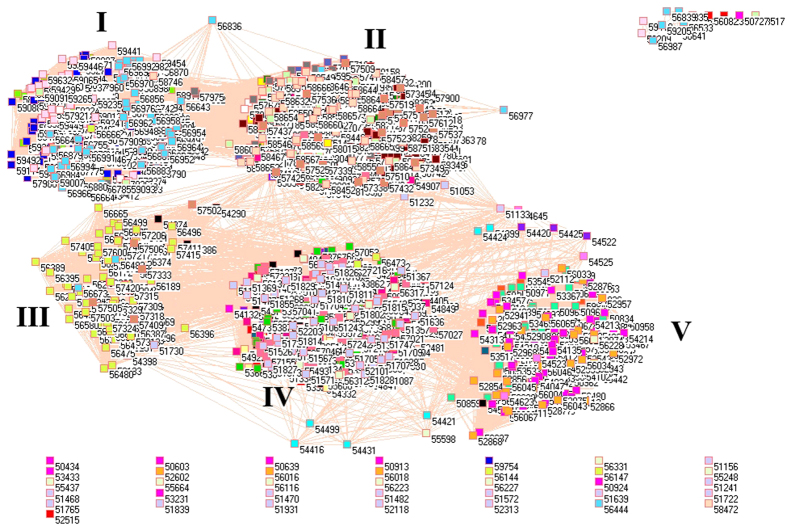
Network of PM_2.5_ emissions from transportation sector in China monitored by 1744 stations in 2010.

Because of different sources in formation of aerosols, it necessarily analyzes PM_2.5_ pollution according to the emissions from industrial, electricity generation, residential and transportation sectors, from which the PM_2.5_ emissions were delineated in Figs 1–8 by means of the network analysis. In these figures, a symbol represents a monitoring station with its code, and 31 colors donate to 22 provinces, 4 municipalities and 5 autonomous regions in China. A line between two symbols interprets that PM_2.5_ emission profiles in the two monitoring stations have a good correlation. A cluster aggregates the symbols that more densely connect each other within the given cluster but sparsely connect with the symbols in other clusters. In accordance with specific features of PM_2.5_ emissions in China, a cluster can mainly come from the places in the same province, for example, the PM_2.5_ emissions from the sector of electricity generation in 2010 (Fig. 4). By contrast, a cluster can encompass several provinces, like the PM_2.5_ emissions from transportation sector in both 2008 and 2010 (Figs 7 and 8). Collectively, network analysis in Figs 1–8 not only throws new light into PM_2.5_ emission but also calls for new solutions in pollution control, policymaking, and environmental restoration.

Figures 1 and 2 show the PM_2.5_ emissions from industrial sector in 2008 and 2010. The major contribution of annual mean PM_2.5_ was attributed to industrial sector in China^52^, which was the typical pollution source in urban environment^53^. Thus, the difference between Figs 1 and 2 describes the fast development in industrial sector in Chinese economy between 2008 and 2010 in light of PM_2.5_ emissions.

The symbols at bottom of Figs 1 and 2 are distinctive because their PM_2.5_ emission profiles are far different from one another (Tables S8 and S9 in Supplementary information). In this context, these distinctive PM_2.5_ emission profiles can be explained by their geographic characteristics. Let us take some notable places as examples. Among them, four places in Fig. 2 are in Beijing (54399, 54424, 54431, 54499) and two places are in Tianjin (54523, 54545). Interestingly, Beijing Pinggu (54424) and Tianjin Wuqing (54523) appear isolated in both Figs 1 and 2, which indicate that the PM_2.5_ emission profiles unchanged in both places between 2008 and 2010. Indeed, Beijing Pinggu is surrounded by mountains in three directions and had almost no industrial sector until very recently, and Tianjin Dagang was an important industrial place in the past, but its location is near to the sea. Being a good example of isolation in Figs 1 and 2, Helongjiang Jidong (50987) has 70% mountains, 25% water field and 5% plain. So the point is that we at first consider these distinctive places with their geographic characteristics.

The forest green symbols at the upper right corner construct a cluster without any connection with any other cluster in 2008 (Fig. 1), and this cluster almost exclusively forms from the places in Shanxi Province, which is famous for its coal mining industry. Therefore this cluster is truly reasonable. In social network analysis^54^, the node that has the largest number of edges is commonly considered to be the source, from where information begins to disperse. If we apply this concept to the PM_2.5_ emission in this cluster, the symbols with most connections are the monitoring stations in Datong (40.05 N, 113.25 E), where coal mining industry concentrates. In fact, Datong served as a controlling point in pollution studies because of its location in the upwind position to Great North China Plain^15^. Remarkably, Datong still had the largest number of connections in 2010 in Fig. 2, however, the forest green cluster losses its importance becoming a part of a big cluster, which marks the measures to control coal mining pollution effectively.

The maroon, wild straw berry and purple symbols are provinces Jiangxi, Shandong and Hebei, and they appeared together in 2008 (right to ellipse in Fig. 1). Since Shandong and Hebei were heavily industrialized provinces, inevitably, heavily pullulated area such as Handan (36.37 N, 114.28 E) had the largest number of connections between circles. Notably, Hebei Province (purple circles) still constructed an independent cluster in 2010, whereas provinces Shandong (wild straw berry circles) and Jiangxi (maroon circles) merged together with other provinces in 2010 in Fig. 2.

The corn flower blue cluster at lower left corner for 2008 in Fig. 1 came from the places of Yunnan Province, and this cluster is distinguishable as only two connections with other clusters can be visible. Yunnan was not heavily industrialized province in 2008, therefore the formation of independent cluster would be explainable. However, this was not the case in 2010, when Yunnan had more similar PM_2.5_ emission profiles with other provinces (Fig. 2).

The lime green cluster, which was located just above the corn flower blue cluster for 2008 in Fig. 1, was Fujian Province, whose industrial sector was not particularly strong in 2008. However, this cluster disappeared in 2010 in Fig. 2.

Immediately above lime green cluster for 2008 in Fig. 1 is Sichuan Province (green yellow cluster), whose fame has been attributed to its surrounding mountains since the ancient times. This geographic importance remains functioning for 2010 in Fig. 2. Apart from abovementioned clusters, the rest symbols actually gathered together in an ellipse in Fig. 1 for 2008, and this cluster became even larger in 2010 as a circle in Fig. 2. As a whole, these changes implicate that the economic development in previously less developed provinces accelerated from 2008 to 2010 and industrial sector in majority parts of China had adopted certain measures to control their pollutions.

Strikingly, network analysis uncovers that the place with the largest number of connections was Guizhou Wanshan (27.31 N, 109.12 E) in 2008, which was the capital of mercury (Hg) in China because the largest Hg mine had existed for many centuries. This is something new because PM_2.5_ emission is usually linked to silicon, Fe, CO, CO_2_, sulfur, biogenic iodine, etc., but not Hg^14^,^55^. However, Wanshan was no longer the place with the largest number of connections in 2010, because it was enlisted as a resource-exhausting city in 2009 by Chinese government. Surprisingly, the result of this policy change can be detected by network analysis.

Figures 3 and 4 exhibit the PM_2.5_ emissions produced by the sector of electricity generation. These two figures have most compelling network clusters because each province, municipality, and autonomous region compose a cluster that does not have many connections with other clusters, and this trend is particularly clear for 2010 in Fig. 4. The regional characteristic in electricity generation sector highlights that each region had its own electricity generation system in 2008 and 2010. Such a system was suitable for local environment, e.g. the hydro-electric electricity generation is normally the powerhouse in Southern China although 70% of the total energy consumption came from coal burning in China^56^.

Again let us start with isolated symbols at bottom in Figs 3 and 4 (Tables S10 and S11 in Supplementary information). These symbols include very particular places because their PM_2.5_ emission profile from electricity generation sector found no similarity in PM_2.5_ emission profiles from their regions, for example, Shanxi Datong (53487) in Fig. 3 whose importance was addressed with respect to its PM_2.5_ emission from industrial sector. Of 70 isolated places in Fig. 3, five and six places came from Inner Mongolia Autonomous Region (53464, 53466, 53469, 53480 and 53481) and Ningxia Province (53517, 53519, 53610, 53611, 53615 and 53618). This high rate of appearance could be partially mixed by the high PM_2.5_ level in winter and nighttime in Mongolia^57^. Meanwhile four isolated places were located in Chongqing Municipality (57339, 57510, 57537 and 57612) in Fig. 3. Municipalities Shanghai and Tianjin appear in Figs 3 and 4 as well, so these features stress the fact that each province, municipality and autonomous region control its electricity generation. Yet, the heavily polluted place such as Hebei Handan (53892) becomes visible in Fig. 4, which was in good agreement with its pollution statute for Beijing-Tianjin-Hebei for these years.

Basically, the clusters in Fig. 3 shaped a U-shape belt. It ran from right to left via Qinghai (dandelion cluster), Gansu (red cluster), Fujian (lime green cluster), Yunnan (corn flower blue cluster), Hubei (olive green cluster), Sichuan (green yellow cluster), Guangdong (blue cluster), Guangxi (pink cluster), Hunan (cadet blue cluster), Chongqing (tan cluster), and then went down via Shaanxi (salmon cluster), Inner Mongolia (magenta cluster), Xinjiang (light purple cluster), Heilongjiang (teal blue cluster), Liaoning (light yellow cluster), Tianjin (lavender cluster), Jilin (black cluster), Jiangsu (maroon cluster), and finally approaches to right via Shandong (wild straw berry cluster), Zhejiang (light orange cluster), Hebei (purple cluster), Shanxi (forest green cluster), and Beijing, where solid waste incineration, chemical manufacturing, coal combustion and coal-fired thermal power generation are the major sources to produce PM_2.5_^52^,^58^. Three columns of clusters in Fig. 4 look more curious: the clusters in upper column were Henan, Hunan, Inner Mongolia, Guangdong, Jilin, Hebei, Sichuan, Zhejiang and Guizhou from right to left; the clusters in middle column were Shandong, Fujian, Jiangxi, Hubei, Xinjiang, Guangxi, Gansu, Shaanxi, Heilongjiang from right to left; the clusters in lower column were Beijing, Hebei, Shanxi, Sichuan, Chongqing, Henan, Ningxia, Shandong, Yunnan, Jilin, Heilongjiang, Tibet, Xinjiang and Qinghai. Thus, Figs 3 and 4 send the strongest message that the control of PM_2.5_ emission from electricity generation should be managed at provincial level.

Figures 5 and 6 display the PM_2.5_ emissions from residential sector. As a whole, the PM_2.5_ emissions from this sector discovered the same pattern for 2008 and 2010 because there are ten similar clusters in Figs 5 and 6. Because PM_2.5_ emissions from residential sector are mainly related to cooking and heating, so these ten clusters implicate their specific points. Essentially, each cluster covered almost the same places in both Figs 5 and 6, suggesting the household energy consumption persisted from 2008 to 2010. Of these ten clusters, 3 clusters were quite separated one from another, including provinces Fujian (Cluster 10), Jiangxi (Cluster 9) and Yunnan (Cluster 7). They did not have any significant connection with other clusters, and were almost exclusively composed of the places within each province.

A completely isolated symbol in Fig. 5 is Heilongjiang Beijicun (53.28 N 122.22 E). The name of this place, Beijicun, means Village of North Pole, which is the place of the highest latitude in China. It is understandable that its residents could take a different pattern in heating and cooking, however, Beijicun was no longer unique in 2010 (Fig. 6) perhaps due to the development of tourism industry.

For both Figs 5 and 6, the other 7 clusters effectively gathered several provinces together each, outlining that the style of cooking and heating were similar in each cluster, which resulted in similar PM_2.5_ emission profiles. Cluster 1 was composed of Xinjiang (light purple triangles), Inner Mongolia (magenta triangles), Tibet (light faded green triangles), Jilin (black triangles), Heilongjiang (teal blue triangles), Liaoning (light yellow triangles) and Qinghai (dandelion triangles). As a result, Cluster 1 gathered the places with very low temperature in China. And then, Ningxia (midnight blue triangles) appears on both sides between Clusters 1 and 2. Additionally, Cluster 1 overlapped with Cluster 2 mainly including Shanxi (forest green triangles) and Gansu (red triangles), where Cluster 3 began. Cluster 3 essentially included Shandong (wild straw berry triangles), Beijing (cyan triangles), Tianjin (lavender triangles), Henan (cadet blue triangles) and Shaanxi (salmon triangles). Most places in Cluster 3 are located in North and central China, where coal burning is ubiquitous for residential sector, for example, Henan Province contributed 7% total emission of China in 2007^59^. Cluster 4 consisted of Anhui (yellow triangles), Zhejing (light orange triangles) and Jiangsu (maroon triangles), so Cluster 4 approached to East China Sea while dust and sea salt account for 10% of PM_2.5_ emission in East Asia^25^. Cluster 5 formed mainly from Hunan (gray triangles), Hubei (olive green triangles), Guizhou (white triangles), and Cluster 6 formed from Sichuan (green yellow triangles) and Chongqing (tan triangles). Very interesting is that people living in Clusters 5 and 6 are famous for their preference to hot and spicy food due to humid and cold weather, which naturally leads to particular PM_2.5_ emission profiles. Cluster 7 exclusively came from Yunnan (corn flower blue triangles). Cluster 8 included Guangdong (blue triangles), Guangxi (pink triangles), and Hainan (orange triangles), so trend once again went to sea. Clusters 9 and 10 came from Jiangxi (light green triangles) and Fujian (lime green triangles). In this circumstance, network analysis amazingly reaches at the same conclusion that two PM_2.5_ pollution belts were defined in China from 1999 to 2011: one from northeast China to Sichuan Province and the other one from Shanghai to Guangxi^60^ based on the public available data^25^,^26^.

Figures 7 and 8 illustrate the PM_2.5_ emissions from transportation sector in 2008 and 2010. Compared with other figures, the profound feature for Figs 7 and 8 is that the connections existed between huge clusters. This fact manifested enormous traffic moving between provinces, which lead to interweave PM_2.5_ emissions between different regions of China. In this sense, we would expect that the isolated symbols were the places poorly commuting with other places, and this expectation proved to be true because these places mainly came from autonomous regions Xinjiang, Tibet and Inner Mongolia (Tables S12 and S13 in Supplementary information). On the other hand, we can see more connections between clusters in 2010 than in 2008, which signified the development of transportation sector in China between 2008 and 2010.

The five huge clusters literally include same provinces, municipalities and autonomous regions for both Figs 7 and 8, and these five clusters can be deciphered as follows. Cluster I included the places mainly from Yunnan (corn flower blue squares), Guangxi (pink squares), Fujian (lime green squares) and Guangdong (blue squares). Cluster II included the places mainly from Anhui (yellow squares), Hunan (gray squares), Jiangsu (maroon squares), Henan (cadet blue squares), Guizhou (white squares), Zhejiang (light orange squares), Jiangxi (light green squares), Hubei (olive green squares) and Chongqing (tan squares). Cluster III mainly came from Sichuan Province (green yellow squares), which is plausible because of its specific geographic location. Cluster IV included Shanxi (forest green squares), Hebei (purple squares), Shaanxi (salmon squares), Shandong (wild straw berry squares), Liaoning (light yellow squares), Gansu (red squares), Jilin (black squares), Xinjiang (light purple squares), Tibet (light faded green squares) and Ningxia (midnight blue squares). Cluster V mainly composed of Heilongjiang (teal blue squares), Hainan (orange squares), Qinghai (dandelion squares), Inner Mongolia (magenta squares) and Beijing (cyan squares). At first glance, it is very strange for the formation of Cluster V, because Heilongjiang is located in the northeast of China, Inner Mongolia in the north, Qinghai in the northwest whereas Hainan in the south. However, a careful review of their geographic and transportation characteristics points out that these regions had less PM_2.5_ emissions from transportation. Although it could not be the case for the other 4 regions, less advanced economic development in these 4 regions could lead to low level of PM_2.5_ emission. Importantly, the special geographic and metrological conditions could also contribute the formation of Cluster V, because Heilongjiang, Inner Mongolia and Qinghai are under harsh weather influence from Siberia, whose winds can easily wipe the pollutants out, meanwhile Hainan as an island is subject to sea winds and precipitations.

In this study, we attempted to apply network analysis to PM_2.5_ emissions in China in 2008 and 2010, which have strong spatial and temporal characteristics because MIX dataset covers the whole China’s territory and records monthly emissions. Network analysis can simultaneously study these spatial and temporal characteristics to fill the knowledge gaps such as how many places have similar emission profiles over those periods, which place has the highest number of similarity in PM_2.5_ emissions, how different places gather together in terms of their similar emission profiles, etc. The potential implication for policymaking could be to eliminate the emission in the place with the highest number of connections, actually the removing of heavy steel enterprise away from Beijing could be considered as an example although it was done prior to network analysis. Nevertheless, network analysis plays a complimentary role in interpretation of observed data, especially in digging out the similarity between different profiles and their changes.

## Materials and Methods

### Data

The monthly PM_2.5_ emission data were obtained from MIX, the mosaic Asian anthropogenic emission inventory for 2008 and 2010, documenting the emissions from 40.125 E to 179.875 E and from 20.125 S to 89.875 N in 0.25°× 0.25° (∼25 km × 25 km) grid^47^. This dataset contains 2168 monitoring stations (Table S1 in Supplementary information).

Although the MIX dataset is the most extensive ground-based monitoring network and provides the most detailed observations so far in China, it turns out that several monitoring stations can be located in the same 0.25°× 0.25° grid, therefore only one monitoring station was selected for network analysis. For example, there are 17 monitoring stations in Beijing Municipality, however, only 13 monitoring stations were selected in order that only one monitoring station exists per 0.25°× 0.25° grid, because the 0.25°× 0.25° grid is the minimal area presenting a single monthly PM_2.5_ emission, As a matter of fact, these 13 monitoring stations in Beijing cover the area from mountain in its north to plain in its south. Undoubtedly, the PM_2.5_ emission from Beijing demonstrates a variety of PM_2.5_ emission profiles due to its diverse geological and metrological conditions.

Also, the MIX dataset is incomplete in some stations, which therefore are excluded from analysis. For the PM_2.5_ emissions from industrial sector, there are 1744 stations in 2008 and 2010 (Table S2 in Supplementary information). For the PM_2.5_ emissions from electricity generation sector, there are 1558 stations in 2008 (Table S3 in Supplementary information) and 1636 stations in 2010 (Table S4 in Supplementary information). For the PM_2.5_ emissions from residential sector, there are 1742 stations in 2008 (Table S5 in Supplementary information) and 1743 stations in 2010 (Table S6 in Supplementary information). For the PM_2.5_ emissions from transportation sector, there are 1744 stations in both years (Table S7 in Supplementary information).

### Network Analysis

As stated in Introduction, an edge between two nodes suggests a relationship, and we define that a good correlation between two PM_2.5_ emission profiles in two places means an existent relationship. For this purpose, we define that a relationship exists when a correlation coefficient is larger than 0.95, namely, an edge exists, and we have verified this value using WGCNA R package^61^. Moreover, squaring of 0.95 results in 0.9025, which is approximate to 0.91 serving as a standard to judge a method^62^. iGraph R package (http://igraph.org/) and Pajek^63^ were used in network analysis.

WGCNA, as a widely used R package in analysis of gene co-expression data, has the advantage to use the so-called soft-threshold to determine the correlation coefficient in profiles between two nodes. However, its computational capacity is limited to about 4000 nodes, which would not be suited to our future studies to include more monitoring stations, for example, India and other regions in Asia, and make comparison with China. Moreover, the clusters are formed according to the values of correlations in WGCNA, which may result in a cluster including highly-correlated but geographically irrelevant places, while we are more interested in geographically relevant places.

## Additional Information

**How to cite this article**: Yan, S. and Wu, G. Network Analysis of Fine Particulate Matter (PM_2.5_) Emissions in China. *Sci. Rep.* **6**, 33227; doi: 10.1038/srep33227 (2016).

## Supplementary Material

Supplementary Information
