# Supplementary material for: Network Analysis of Fine Particulate Matter (PM2.5) Emissions in China
Source: Sci Rep. 2016 Sep 9;6:33227. doi: 10.1038/srep33227 (PMC5016853; doi:10.1038/srep33227)
Supplement: Supplementary Information [file srep33227-s1.pdf]

Network Analysis of Fine Particulate Matter (PM2.5) Emissions in China

Shaomin Yan and Guang Wu*

Supplementary Information

List of Supplementary information

Table S1 2168 monitoring stations in MIX

Table S2. 1744 monitoring stations whose PM2.5 emissions are from industrial sector

Table S3. 1558 monitoring stations whose PM2.5 emissions are from electricity generation sector

Table S4. 1636 monitoring stations whose PM2.5 emissions are from electricity generation sector

Table S5. 1742 monitoring stations whose PM2.5 emissions are from residential sector

Table S6. 1743 monitoring stations whose PM2.5 emissions are from residential sector

Table S7. 1744 monitoring stations whose PM2.5 emissions are from transportation sector

Table S8. Isolated symbols at bottom in Figure 1

Table S9. Isolated symbols at bottom in Figure 2

Table S10. Isolated symbols at bottom in Figure 3

Table S11. Isolated symbols at bottom in Figure 4

Table S12. Isolated symbols at bottom in Figure 7

Table S13. Isolated symbols at bottom in Figure 8

Table S1 2168 monitoring stations in MIX

Anhui Bengbu	58221
Anhui Bozhou	58102
Anhui Changfeng	58220
Anhui Chaohu	58326
Anhui Chuzhou	58236
Anhui Dangshan	58015
Anhui Dangtu	58335
Anhui Dingyuan	58225
Anhui Fanchang	58337
Anhui Feidong	58323
Anhui Feixi	58320
Anhui Fengtai	58212
Anhui Fengyang	58222
Anhui Funan	58202
Anhui Fuyang	58203
Anhui Guangde	58441
Anhui Guzhen	58128
Anhui Hanshan	58330
Anhui Hefei	58321
Anhui Hexian	58331
Anhui Huaibei	58116
Anhui Huainan	58224
Anhui Huaiyuan	58127
Anhui Huangshan	58437
Anhui Huoqiu	58214
Anhui Huoshan	58314
Anhui Jieshou	58108
Anhui Jingde	58435
Anhui Jingxian	58432
Anhui Jinzhai	58306
Anhui Laian	58234
Anhui Langxi	58442
Anhui Leysin	58117
Anhui Lingbi	58125
Anhui Linquan	58107
Anhui Luan	58311
Anhui Lujiang	58327
Anhui Maanshan	58336
Anhui Mengcheng	58118
Anhui Mingguang	58223
Anhui Nanling	58431

Anhui Ningguo	58436
Anhui Qimen	58520
Anhui Shexian	58530
Anhui Shouxian	58215
Anhui Shucheng	58316
Anhui Sixian	58126
Anhui Suixi	58113
Anhui Susong	58417
Anhui Suzhou	58122
Anhui Taihe	58109
Anhui Tianchang	58240
Anhui Tianzhushan	58112
Anhui Tongcheng	58319
Anhui Tongling	58429
Anhui Tunxi	58531
Anhui Woyang	58114
Anhui Wuhe	58129
Anhui Wuhu	58334
Anhui Wuhuxian	58338
Anhui Wuwei	58329
Anhui Xiaoxian	58016
Anhui Xiuning	58534
Anhui Xuancheng	58433
Anhui Yingshang	58210
Anhui Yixian	58523
Anhui Yuexi	58317
Beijing	54511
Beijing Changping	54499
Beijing Chaoyang	54433
Beijing Daxing	54594
Beijing Fangshan district	54596
Beijing Fengtai	54514
Beijing Haidian	54399
Beijing Huairou	54419
Beijing Mentougou	54505
Beijing Miyun	54416
Beijing Miyunshangdianzi	54421
Beijing Pinggu	54424
Beijing Shijingshan	54513
Beijing Shunyi	54398
Beijing Tongzhou	54431
Beijing Xiayunling	54597
Beijing Yanqing	54406

Beijing Zhaitang	54501
Chaoyang Guangdong	59318
Chenghai Guangdong	59319
China Liuzhou	59046
Chongqing Banan	57518
Chongqing Beibei	57511
Chongqing Bishan	57514
Chongqing Changshou	57520
Chongqing Chengkou	57333
Chongqing Dazu	57502
Chongqing Dianjiang	57425
Chongqing Dwelling	57633
Chongqing Fengdu	57523
Chongqing Fengjie	57348
Chongqing Hechuan	57512
Chongqing Jiangjin	57517
Chongqing Kaixian	57338
Chongqing Mountain	57635
Chongqing Nanchuan	57519
Chongqing Pengshui	57537
Chongqing Qianjiang	57536
Chongqing Qijiang	57612
Chongqing Rongchang	57505
Chongqing Shapingba	57516
Chongqing Shizhu	57438
Chongqing Tiancheng	57431
Chongqing Tongliang	57510
Chongqing Tongnan	57409
Chongqing Wansheng	57509
Chongqing Wanzhou	57432
Chongqing Wulong	57525
Chongqing Wushan	57349
Chongqing Wuxi	57345
Chongqing Yongchuan	57506
Chongqing Yubei	57513
Chongqing Yunyang	57339
Chongqing Zhongxian	57437
Fujian Anxi	58929
Fujian Changle	58941
Fujian Changtai	59122
Fujian Changting	58911
Fujian Chongwu	59133
Fujian Datian	58923

Fujian Dehua	58935
Fujian Fuan	58748
Fujian Fuding	58754
Fujian Fuqing	58942
Fujian Fuzhou	58847
Fujian Guangze	58724
Fujian Gutian	58836
Fujian Huaan	58928
Fujian Jianning	58822
Fujian Jianou	58737
Fujian Jianyang	58734
Fujian Jiuxianshan	58931
Fujian Lianjiang	58848
Fujian Longhai	59127
Fujian Longyan	58927
Fujian Luoyuan	58845
Fujian Mingxi	58824
Fujian Minhou	58844
Fujian Nanan	59131
Fujian Nanjing	59124
Fujian Ningde	58846
Fujian Ninghuai	58818
Fujian Pinghe	59125
Fujian Pingtan	58944
Fujian Pucheng	58731
Fujian Putian	58946
Fujian Qingliu	58819
Fujian Sanming	58828
Fujian Sansha	58850
Fujian Shanghang	58918
Fujian Shaowu	58725
Fujian Shaxian	58826
Fujian Shouning	58744
Fujian Shunchang	58823
Fujian Shuqing	58839
Fujian Songxi	58735
Fujian Taining	58820
Fujian Tongan	59130
Fujian Wuping	58917
Fujian Wuyishan	58730
Fujian Xiamen	59134
Fujian Xiapu	58843
Fujian Xiuyu	58938

Fujian Yongchun	58934
Fujian Yongding	59113
Fujian Yongtai	58932
Fujian Youxi	58837
Fujian Yunxiao	59322
Fujian Zhangping	58926
Fujian Zhangpu	59129
Fujian Zhaoan	59320
Fujian Zhenghe	58736
Fujian Zherong	58749
Fujian Zhouning	58747
Gansu Anding	52995
Gansu Baiyin	52896
Gansu Chengxian	57102
Gansu Chongxin	53928
Gansu Dangchang	56095
Gansu Dibu	56084
Gansu Dongxiang	52981
Gansu Gaitai	52546
Gansu Gangu	57001
Gansu Gaolan	52884
Gansu Guanghe	52982
Gansu Gulang	52784
Gansu Hezheng	52985
Gansu Hezuo	56080
Gansu Huachi	53930
Gansu Huajialing	52996
Gansu Huanxian	53821
Gansu Huating	53927
Gansu Huining	52993
Gansu Huishui	53934
Gansu Huixian	57110
Gansu Jingchuan	53926
Gansu Jingning	53906
Gansu Jingtai	52797
Gansu Jingyuan	52895
Gansu Jiuquan	52533
Gansu Kangle	52988
Gansu Kangxian	57105
Gansu Kongtong	53915
Gansu Liangdang	57111
Gansu Lingtai	53924
Gansu Lintan	56081

Gansu Lintao	52986
Gansu Linxia	52984
Gansu Linze	52557
Gansu Lixian	57007
Gansu Longxi	56092
Gansu Luqu	56071
Gansu Maiji	57014
Gansu Maqu	56074
Gansu Mazongshan	52323
Gansu Minle	52656
Gansu Minqin	52681
Gansu Minxian	56093
Gansu Ningxian	53937
Gansu Qinan	57002
Gansu Qingcheng	53829
Gansu Qingshui	57011
Gansu Subei	52515
Gansu Sunan	52643
Gansu Tianshui	57006
Gansu Tianzhu	52881
Gansu Tongwei	53908
Gansu Weiyuan	52998
Gansu Wenxian	56192
Gansu Wudu	56096
Gansu Wushan	57004
Gansu Wushaoling	52787
Gansu Wuwei	52679
Gansu Xiahe	52978
Gansu Xifeng	53923
Gansu Xihe	57008
Gansu Yongchang	52674
Gansu Yongdeng	52885
Gansu Yongjing	52980
Gansu Yuzhong	52983
Gansu Zhangjiachuan	57012
Gansu Zhangxian	56091
Gansu Zhangye	52652
Gansu Zhengning	53935
Gansu Zhenyuan	53925
Gansu Zhouqu	56094
Gansu Zhuanglang	53917
Gansu Zhuoni	56082
Guangdong Boluo	59297

Guangdong Chaozhou	59312
Guangdong Conghua	59285
Guangdong Daipu	59116
Guangdong Deqing	59269
Guangdong Dianbai	59664
Guangdong Dongguan	59289
Guangdong Doumen	59487
Guangdong Enping	59477
Guangdong Fengkai	59264
Guangdong Fengshun	59310
Guangdong Fogang	59087
Guangdong Gaoyao	59278
Guangdong Gaozhou	59653
Guangdong Guangning	59271
Guangdong Guangzhou	59287
Guangdong Haifeng	59500
Guangdong Heping	59099
Guangdong Heshan	59473
Guangdong Heyuan	59293
Guangdong Huadu	59284
Guangdong Huaiji	59270
Guangdong Huazhou	59655
Guangdong Huidong	59492
Guangdong Huilai	59317
Guangdong Huiyang	59298
Guangdong Jiaoling	59114
Guangdong Jiexi	59306
Guangdong Jieyang	59315
Guangdong Kaiping	59475
Guangdong Lechang	57988
Guangdong Leizhou	59750
Guangdong Lianjiang	59654
Guangdong Liannan	59071
Guangdong Lianping	59096
Guangdong Lianshan	59074
Guangdong Lianzhou	59072
Guangdong Longchuan	59107
Guangdong Longmen	59290
Guangdong Lufeng	59502
Guangdong Luoding	59462
Guangdong Maoming	59659
Guangdong Meixian	59117
Guangdong Nanau	59324

Guangdong Nanhai	59288
Guangdong Nanxiong	57996
Guangdong Panyu	59481
Guangdong Pingyuan	59106
Guangdong Puning	59314
Guangdong Qingyuan	59280
Guangdong Raoping	59313
Guangdong Renhua	57989
Guangdong Ruyuan	59081
Guangdong Sanshui	59279
Guangdong Shangchuandao	59673
Guangdong Shantou	59316
Guangdong Shanwei	59501
Guangdong Shaoguan	59082
Guangdong Shenzhen	59493
Guangdong Shixing	59090
Guangdong Shunde	59480
Guangdong Sihui	59276
Guangdong Suixi	59650
Guangdong Taishan	59478
Guangdong Wengyuan	59094
Guangdong Wuchuan	59656
Guangdong Wuhua	59303
Guangdong Xinfeng	59097
Guangdong Xingning	59109
Guangdong Xinhui	59476
Guangdong Xinxing	59470
Guangdong Xinyi	59456
Guangdong Xuwen	59754
Guangdong Yangchun	59469
Guangdong Yangjiang	59663
Guangdong Yangshan	59075
Guangdong Yingde	59088
Guangdong Yunan	59268
Guangdong Yunfu	59471
Guangdong Zengcheng	59294
Guangdong Zhanjiang	59658
Guangdong Zhongshan	59485
Guangdong Zhuhai	59488
Guangdong Zijin	59304
Guangxi Baise	59211
Guangxi Bama	59027
Guangxi Beihai	59644

Guangxi Beiliu	59451
Guangxi Binyang	59238
Guangxi Bobai	59449
Guangxi Cangwu	59266
Guangxi Cenxi	59454
Guangxi Daxin	59421
Guangxi Debao	59215
Guangxi Donglan	59025
Guangxi Duan	59037
Guangxi Fangchenggang	59635
Guangxi Fengshan	59021
Guangxi Gongcheng	59052
Guangxi Guanyang	57964
Guangxi Guilin	57957
Guangxi Hechi	59023
Guangxi Hengxian	59441
Guangxi Hepu	59640
Guangxi Hezhou	59065
Guangxi Huanjiang	59031
Guangxi Jingxi	59218
Guangxi Jinxiu	59057
Guangxi Laibin	59242
Guangxi Leye	59012
Guangxi Lingchuan	57956
Guangxi Lingshan	59446
Guangxi Lingui	57954
Guangxi Lingyun	59015
Guangxi Lipu	59055
Guangxi Liucheng	59041
Guangxi Liujiang	59047
Guangxi Longan	59229
Guangxi Longlin	59001
Guangxi Longsheng	57942
Guangxi Luchuan	59457
Guangxi Luzhai	59045
Guangxi Mashan	59230
Guangxi Mengshan	59058
Guangxi Nanning	59431
Guangxi Nanning	59432
Guangxi Napo	59209
Guangxi Pingguo	59228
Guangxi Pingle	59053
Guangxi Pingnan	59255

Guangxi Pingxiang	59419
Guangxi Pubei	59448
Guangxi Qinzhou	59632
Guangxi Quanzhou	57960
Guangxi Rongan	57947
Guangxi Rongshui	57948
Guangxi Rongxian	59452
Guangxi Sanjiang	57941
Guangxi Shanglin	59235
Guangxi Shangsi	59429
Guangxi Shatang	59044
Guangxi Tengxian	59256
Guangxi Tiandeng	59227
Guangxi Tiandong	59224
Guangxi Tiane	57927
Guangxi Tianlin	59017
Guangxi Wuming	59237
Guangxi Wuxuan	59246
Guangxi Wuzhou	59265
Guangxi Xiangzhou	59241
Guangxi Xilin	59004
Guangxi Xincheng	59038
Guangxi Xingan	57955
Guangxi Yizhou	59034
Guangxi Yongfu	57949
Guangxi Yongning	59435
Guangxi Zhaoping	59059
Guangxi Ziyuan	57859
Guizhou Anlong	57908
Guizhou Anshun	57806
Guizhou Baiyun	57911
Guizhou Bijie	57707
Guizhou Ceheng	57909
Guizhou Cengong	57735
Guizhou Congjiang	57936
Guizhou Dafang	57708
Guizhou Danzhai County	57829
Guizhou Daozhen	57623
Guizhou Dejiang	57637
Guizhou Dushan	57922
Guizhou Duyun	57827
Guizhou Fenggang	57723
Guizhou Fuquan	57821

Guizhou Guanling	57903
Guizhou Guiding	57824
Guizhou Guiyang	57816
Guizhou Hezhang	56598
Guizhou Huangping	57822
Guizhou Huaxi	57914
Guizhou Huishui	57912
Guizhou Jiangkou	57736
Guizhou Jianhe	57835
Guizhou Jinping	57844
Guizhou Jinsha	57714
Guizhou Kaili	57825
Guizhou Kaiyang	57719
Guizhou Leishan	57837
Guizhou Libo	57926
Guizhou Liping	57839
Guizhou Liuzhi	57807
Guizhou Longli	57913
Guizhou Luodian	57916
Guizhou Majiang	57828
Guizhou Meitan	57722
Guizhou Nayong	57800
Guizhou Panxian	56793
Guizhou Pingba	57814
Guizhou Pingtang	57921
Guizhou Puan	56792
Guizhou Puding	57808
Guizhou Qinglong	57900
Guizhou Renhuai	57710
Guizhou Rongjiang	57932
Guizhou Sandu	57923
Guizhou Sansui	57832
Guizhou Shibing	57737
Guizhou Shiqian	57734
Guizhou Sinan	57731
Guizhou Songtao	57647
Guizhou Taijiang	57834
Guizhou Tianzhu	57840
Guizhou Tongren	57741
Guizhou Tongzi	57606
Guizhou Wangmo	57906
Guizhou Wanshan	57742
Guizhou Weining	56691

Guizhou Wengan	57728
Guizhou Wuchuan	57634
Guizhou Wudang	57915
Guizhou Xianxi	57803
Guizhou Xifeng	57718
Guizhou Xingren	57902
Guizhou Xingyi	57907
Guizhou Xiuwen	57811
Guizhou Yanhe	57636
Guizhou Yinjiang	57732
Guizhou Yuping	57739
Guizhou Yuqing	57729
Guizhou Zhenfeng	57905
Guizhou Zhengan	57625
Guizhou Zhenning	57809
Guizhou Zhenyuan	57738
Guizhou Zhijin	57805
Guizhou Ziyun	57910
Guizhou Zunyi	57717
Hainan Baisha	59848
Hainan Baoting	59945
Hainan Changjiang	59847
Hainan Chengmai	59843
Hainan Danzhou	59845
Hainan Dingan	59851
Hainan Dongfang	59838
Hainan Haikou	59758
Hainan Lingao	59842
Hainan Qiongshan	59757
Hainan Qiongzhong	59849
Hainan Qonghai	59855
Hainan Tunchang	59854
Hainan Wanning	59951
Hainan Wenchang	59856
Hainan Wuzhishan	59941
Hebei Weixian	53896
Hebei Anguo	54604
Hebei Anping	54609
Hebei Anxin	54605
Hebei Baixiang	53785
Hebei Baoding	54602
Hebei Bazhou	54518
Hebei Botou	54618

Hebei Cangzhou	54616
Hebei Caofeidian	54535
Hebei Changli	54540
Hebei Chengan	53996
Hebei Chengde	54423
Hebei Chengdexian	54430
Hebei Chicheng	54404
Hebei Chongli	54304
Hebei Cixian	53897
Hebei Dachang	54510
Hebei Dacheng	54613
Hebei Daming	54804
Hebei Dingzhou	53696
Hebei Dongguang	54713
Hebei Feixiang	53980
Hebei Fengfeng	53894
Hebei Fengnan	54533
Hebei Fengning	54308
Hebei Fengrun	54532
Hebei Fucheng	54710
Hebei Funing	54541
Hebei Fuping	53690
Hebei Gaobeidian	54506
Hebei Gaocheng	53697
Hebei Gaoyi	53790
Hebei Goyang	54603
Hebei Guan	54512
Hebei Guangping	53899
Hebei Guangzong	54631
Hebei Guantao	54809
Hebei Gucheng	54707
Hebei Guyuan	54301
Hebei Haixing	54628
Hebei Handan	53892
Hebei Heiqiu	53797
Hebei Hejian	54614
Hebei Hengshui	54702
Hebei Huaian	53491
Hebei Huailai	54405
Hebei Huanghua	54624
Hebei Jingjing	53693
Hebei Jingxian	54711
Hebei Jinzhou	53689

Hebei Jize	54640
Hebei Jizhou	54704
Hebei Julu	53799
Hebei Kangbao	53392
Hebei Kuancheng	54432
Hebei Laiyuan	53599
Hebei Langfang	54515
Hebei Leting	54539
Hebei Lincheng	53792
Hebei Lingshou	53680
Hebei Linxi	54801
Hebei Linzhang	53773
Hebei Lixian	54620
Hebei Longhua	54318
Hebei Longyao	53794
Hebei Luancheng	53789
Hebei Luannan	54437
Hebei Luanping	54420
Hebei Luanxian	54531
Hebei Lulong	54438
Hebei Mancheng	54611
Hebei Mengcun	54644
Hebei Nangong	54705
Hebei Nanhe	53891
Hebei Nanpi	54719
Hebei Ningjin	53796
Hebei Pingquan	54319
Hebei Pingshan	53694
Hebei Pingxiang	54632
Hebei Qianan	54439
Hebei Qianxi	54434
Hebei Qinghe	54706
Hebei Qinglong	54436
Hebei Qingxian	54615
Hebei Qinhuangdao	54449
Hebei Qiuxian	54820
Hebei Quyang	53682
Hebei Quzhou	53893
Hebei Raoyang	54606
Hebei Renqiu	54610
Hebei Renxian	53883
Hebei Rongcheng	54503
Hebei Sanhe	54520

Hebei Shahe	53781
Hebei Shangyi	53397
Hebei Shenze	54621
Hebei Shenzhou	54608
Hebei Shexian	53886
Hebei Shunping	53596
Hebei Suning	54626
Hebei Tangshan	54534
Hebei Tangxian	53692
Hebei Wangdu	54607
Hebei Wanquan	53499
Hebei Weichang	54311
Hebei Weixian	53593
Hebei Wenan	54612
Hebei Wuan	53890
Hebei Wuji	53699
Hebei Wuqiang	54700
Hebei Wuqiao	54717
Hebei Wuyi	54703
Hebei Xianghe	54521
Hebei Xianxian	54617
Hebei Xinglong	54425
Hebei Xingtai	53798
Hebei Xingtang	53688
Hebei Xinhe	54633
Hebei Xinji	54701
Hebei Xinle	53695
Hebei Xiongxian	54636
Hebei Xuanhua	53498
Hebei Xushui	54601
Hebei Yangyuan	53492
Hebei Yanshan	54627
Hebei Yixian	54507
Hebei Yongnian	53895
Hebei Yongqing	54519
Hebei Yuanshi	53791
Hebei Yutian	54522
Hebei Zanhuang	53795
Hebei Zaoqiang	54708
Hebei Zhangbei	53399
Hebei Zhangjiakou	54401
Hebei Zhaoxian	53784
Hebei Zhengding	53691

Hebei Zhulu	54408
Hebei Zhuozhou	54502
Hebei Zunhua	54429
Hebei-Weixian	54800
Heilongjiang Acheng	50958
Heilongjiang Aihui	50468
Heilongjiang Anda	50854
Heilongjiang Baiquan	50755
Heilongjiang Baoqing	50888
Heilongjiang Bayan	50867
Heilongjiang Beian	50656
Heilongjiang Beijicun	50137
Heilongjiang Beilin	50853
Heilongjiang Binxian	50960
Heilongjiang Boli	50973
Heilongjiang Daqing	50850
Heilongjiang Dongning	54099
Heilongjiang Dumeng	50842
Heilongjiang Fujin	50788
Heilongjiang Fuyu	50742
Heilongjiang Fuyuan	50779
Heilongjiang Gannan	50741
Heilongjiang Haerbin	50953
Heilongjiang Hailin	54092
Heilongjiang Hailun	50756
Heilongjiang Hegang	50775
Heilongjiang Huachuan	50878
Heilongjiang Huanan	50879
Heilongjiang Hulan	50956
Heilongjiang Hulin	50983
Heilongjiang Huma	50353
Heilongjiang Huzhong	50247
Heilongjiang Jiagedaqi	50442
Heilongjiang Jiamusi	50873
Heilongjiang Jiayin	50673
Heilongjiang Jidong	50987
Heilongjiang Jixi	50978
Heilongjiang Jixian	50880
Heilongjiang Kedong	50659
Heilongjiang Keshan	50658
Heilongjiang Lansi	50859
Heilongjiang Lindian	50749
Heilongjiang Linkou	50979

Heilongjiang Longjiang	50739
Heilongjiang Luobei	50776
Heilongjiang Minshui	50758
Heilongjiang Mishan	50985
Heilongjiang Mohe	50136
Heilongjiang Mudanjiang	54094
Heilongjiang Mulan	50962
Heilongjiang Muling	54093
Heilongjiang Nehe	50646
Heilongjiang Nenjiang	50557
Heilongjiang Ningan	54098
Heilongjiang Qingan	50861
Heilongjiang Qinggang	50851
Heilongjiang Qiqihaer	50745
Heilongjiang Qitaihe	50971
Heilongjiang Raohe	50892
Heilongjiang Shangzhi	50968
Heilongjiang Shuangyashan	50884
Heilongjiang Suibin	50787
Heilongjiang Suifenhe	54096
Heilongjiang Suileng	50767
Heilongjiang Sunwu	50564
Heilongjiang Tahe	50246
Heilongjiang Tailai	50844
Heilongjiang Tangyuan	50871
Heilongjiang Tieli	50862
Heilongjiang Tonghe	50963
Heilongjiang Tongjiang	50778
Heilongjiang Wangkui	50852
Heilongjiang Wuchang	54080
Heilongjiang Wudalianchi	50655
Heilongjiang Wuyiling	50674
Heilongjiang Wuying	50772
Heilongjiang Xinlin	50349
Heilongjiang Xunke	50566
Heilongjiang Yanshou	50965
Heilongjiang Yian	50750
Heilongjiang Yichun	50774
Heilongjiang Yilang	50877
Heilongjiang Zhaodong	50858
Heilongjiang Zhaoyuan	50954
Heilongjiang Zhaozhou	50950
Heilongjiang Zhengfang	50964

Henan　Qixian	57096
Henan　Xixian	57396
Henan Anyang	53898
Henan Baofeng	57181
Henan Biyang	57281
Henan Boai	53979
Henan Changge	57087
Henan Changheng	53998
Henan Chuanhui	57195
Henan Dancheng	58100
Henan Dengfeng	57082
Henan Dengzhou	57274
Henan Echeng	58007
Henan Fangcheng	57179
Henan Fanxian	54903
Henan Fengqiu	53983
Henan Fugou	57098
Henan Gongyi	57080
Henan Guangshan	57299
Henan Gushi	58208
Henan Huaibin	58205
Henan Huaiyang	57192
Henan Huangchuan	58207
Henan Huaxian	53995
Henan Huixian	53985
Henan Huojia	53988
Henan Jiaozuo	53982
Henan Jiaxian	57180
Henan Jigongshan	57390
Henan Jiyuan	53978
Henan Junxian	53992
Henan Kaifeng	57091
Henan Lankao	57093
Henan Lingbao	57056
Henan Linying	57183
Henan Linzhou	53889
Henan Luohe	57186
Henan Luoning	57066
Henan Lushan	57173
Henan Luyi	58101
Henan Mengjin	57071
Henan Mengzhou	57072
Henan Mianchi	57063

Henan Minquan	58004
Henan Nanle	54901
Henan Nanyang	57178
Henan Nanzhao	57176
Henan Neihuang	53993
Henan Neixiang	57169
Henan Ningling	58008
Henan Pingyu	57292
Henan Puyang	54900
Henan Qingfeng	54902
Henan Qinyang	53972
Henan Qixian	53974
Henan Queshan	57294
Henan Roshan	57298
Henan Runan	57197
Henan Ruyang	57078
Henan Ruzhou	57075
Henan Sanmenxia	57051
Henan Shangcheng	58301
Henan Shangqiu	58005
Henan Shangshui	57198
Henan Shangxai	57194
Henan Shenqiu	58104
Henan Sheqi	57187
Henan Songshan	57084
Henan Songxian	57162
Henan Suiping	57189
Henan Suixian	58001
Henan Taikang	57099
Henan Taiqian	54817
Henan Tanghe	57273
Henan Tangyin	53991
Henan Tongbai	57285
Henan Tongxu	57191
Henan Weihui	53994
Henan Weishi	57094
Henan Wenxian	57079
Henan Wugang	57177
Henan Wuyang	57185
Henan Wuzhi	53987
Henan Xiangcheng	57182
Henan Xiangcheng	57196
Henan Xiayi	58017

Henan Xichuan	57261
Henan Xihua	57193
Henan Xinan	57070
Henan Xincai	57293
Henan Xingyang	57081
Henan Xinmi	57085
Henan Xinxiang	53986
Henan Xinyang	57297
Henan Xinye	57271
Henan Xinzheng	57086
Henan Xiping	57188
Henan Xiuwu	53984
Henan Xixia	57156
Henan Xixian	57296
Henan Xuchang	57089
Henan Yanjin	53997
Henan Yanling	57095
Henan Yanshi	57076
Henan Yellow River Flood Zone Agricultural Experimentation Station	57190
Henan Yexian	57184
Henan Yichuan	57074
Henan Yongcheng	58111
Henan Yuanyang	53989
Henan Yucheng	58006
Henan Yuzhou	57088
Henan Zhengyang	57295
Henan Zhengzhou	57083
Henan Zhenping	57175
Henan Zhongmou	57090
Henan Zhumadian	57290
Hubei Anlu	57388
Hubei Baokang	57361
Hubei Caidian	57489
Hubei Changyang	57464
Hubei Chibi	57582
Hubei Chongyang	57586
Hubei Danjiangkou	57260
Hubei Dawu	57395
Hubei Daye	57499
Hubei Enshi	57447
Hubei Ezhou	57496
Hubei Fangxian	57259

Hubei Gongan	57477
Hubei Gucheng	57268
Hubei Hanchuan	57486
Hubei Hefeng	57543
Hubei Hongan	57398
Hubei Honghu	57581
Hubei Huanggang	57498
Hubei Huangmei	58409
Hubei Jianli	57573
Hubei Jianshi	57445
Hubei Jiayu	57583
Hubei Jingmen	57377
Hubei Jingshan	57387
Hubei Jingzhou	57476
Hubei Jinsha	57596
Hubei Laifeng	57545
Hubei Laohekou	57265
Hubei Lichuan	57439
Hubei Luotian	58401
Hubei Macheng	57399
Hubei Nanzhang	57363
Hubei Padang	57355
Hubei Qianjiang	57475
Hubei Qichun	58408
Hubei Shayang	57484
Hubei Shennongjia	57362
Hubei Shishou	57571
Hubei Shiyan	57256
Hubei Songzi	57469
Hubei Suizhou	57381
Hubei Tianmen	57483
Hubei Tongcheng	57589
Hubei Tuanfeng	57495
Hubei Wufeng	57458
Hubei Wuhan	57494
Hubei Wuxue	58501
Hubei Xianfeng	57540
Hubei Xiangyang	57278
Hubei Xianning	57590
Hubei Xiantao	57485
Hubei Xiaochang	57386
Hubei Xiaogan	57482
Hubei Xingshan	57359

Hubei Xinzhou	57492
Hubei Xishui	58404
Hubei Xuanen	57541
Hubei Yangxin	58500
Hubei Yichang	57461
Hubei Yicheng	57370
Hubei Yidu	57465
Hubei Yiling	57453
Hubei Yingcheng	57481
Hubei Yingshan	58402
Hubei Yunmeng	57389
Hubei Yunxi	57251
Hubei Yunxian	57253
Hubei Zaoyang	57279
Hubei Zhongxiang	57378
Hubei Zhushan	57257
Hubei Zhuxi	57249
Hunan Anhua	57669
Hunan Anren	57881
Hunan Anxiang	57577
Hunan Baojing	57642
Hunan Chaling	57882
Hunan Changde	57662
Hunan Changning	57874
Hunan Changsha	57687
Hunan Chenxi	57658
Hunan Chenzhou	57972
Hunan Cili	57564
Hunan Daoxian	57965
Hunan Dongan	57867
Hunan Fenghuang	57740
Hunan Guangxi	57889
Hunan Guiyang	57973
Hunan Guzhang	57646
Hunan Hanshou	57663
Hunan Hengdong	57778
Hunan Hengnan	57875
Hunan Hengshan	57777
Hunan Hengyang	57872
Hunan Hengyangxian	57871
Hunan Heshan	57674
Hunan Hongjiang	57754
Hunan Huarong	57575

Hunan Huayuan	57640
Hunan Jiahe	57974
Hunan Jianghua	59063
Hunan Jiangyong	57969
Hunan Jishou	57649
Hunan Lanshan	57975
Hunan Leiyang	57876
Hunan Lengshuijiang	57760
Hunan Lengshuitan	57865
Hunan Lianyuan	57762
Hunan Liling	57781
Hunan Linli	57566
Hunan Linwu	57978
Hunan Linxiang	57585
Hunan Liuyang	57688
Hunan Lixian	57565
Hunan Longshan	57544
Hunan Loudi	57763
Hunan Luxi	57657
Hunan Mapoling	57679
Hunan Mayang	57743
Hunan Milo	57680
Hunan Nanxian	57574
Hunan Nanyue	57776
Hunan Ningxiang	57678
Hunan Ningyuan	57966
Hunan Pingjiang	57682
Hunan Qidong	57870
Hunan Qiyang	57868
Hunan Rucheng	57985
Hunan Sangzhi	57554
Hunan Shaoshan	57771
Hunan Shimen	57562
Hunan Shuangfeng	57774
Hunan Shuangpai	57962
Hunan Taojiang	57666
Hunan Taoyuan	57661
Hunan Tongtao	57845
Hunan Xiangtan	57773
Hunan Xiangxiang	57772
Hunan Xiangyin	57673
Hunan Xinhua	57761
Hunan Xinhuang	57744

Hunan Xintian	57971
Hunan Xupu	57752
Hunan Yanling	57886
Hunan Yizhang	57976
Hunan Yongshun	57643
Hunan Yongxing	57887
Hunan Yongzhou	57866
Hunan Youxian	57779
Hunan Yuanjiang	57671
Hunan Yuanling	57655
Hunan Yueyang	57584
Hunan Zhangjiajie	57558
Hunan Zhijiang	57745
Hunan Zhuzhou	57780
Hunan Zixing	57981
Inner Mongolia Abagaqi	53192
Inner Mongolia Aershan	50727
Inner Mongolia Alukeerqinqi	54122
Inner Mongolia Aohanqi	54225
Inner Mongolia Arunqi	50647
Inner Mongolia Balihan	54316
Inner Mongolia Balinyouqi	54113
Inner Mongolia Balinzuoqi	54027
Inner Mongolia Baoguotu	54226
Inner Mongolia Baotou	53446
Inner Mongolia Bayaertuhushuo	50928
Inner Mongolia Chayouhouqi	53384
Inner Mongolia Chayouqianqi	53481
Inner Mongolia Chayouzhongqi	53378
Inner Mongolia Chenbaerhuqi	50524
Inner Mongolia Chifeng	54218
Inner Mongolia Dalateqi	53457
Inner Mongolia Damaoqi	53352
Inner Mongolia Dashetai	53348
Inner Mongolia Dengkou	53419
Inner Mongolia Dongsheng	53543
Inner Mongolia Duolunxian	54208
Inner Mongolia Eduokeqianqi	53730
Inner Mongolia Eerguna	50425
Inner Mongolia Ejinhuoluoqi	53545
Inner Mongolia Elunchunqi	50445
Inner Mongolia Erlianhaote	53068
Inner Mongolia Etukeqi	53529

Inner Mongolia Evenkeqi	50525
Inner Mongolia Fengzhen	53484
Inner Mongolia Fuhe	54024
Inner Mongolia Gangzi	54214
Inner Mongolia Gaoliban	54031
Inner Mongolia Genhe	50431
Inner Mongolia Guyangxian	53357
Inner Mongolia Hailaer	50527
Inner Mongolia Hailisu	53231
Inner Mongolia Hangjinhouqi	53420
Inner Mongolia Hangjinqi	53533
Inner Mongolia Helingeerxian	53469
Inner Mongolia Henan	53732
Inner Mongolia Houlinguole	50924
Inner Mongolia Huade	53391
Inner Mongolia Huhehaote	53463
Inner Mongolia Huhehaote suburb	53466
Inner Mongolia Jining	53480
Inner Mongolia Kailu	54134
Inner Mongolia Kalaqinqi	54313
Inner Mongolia Keerxinyouyizhongqi	50937
Inner Mongolia Keshiketengqi	54117
Inner Mongolia Kezuohouqi	54231
Inner Mongolia Kezuozhongqi	54047
Inner Mongolia Kulun	54234
Inner Mongolia Liangcheng	53475
Inner Mongolia Linhe	53513
Inner Mongolia Linxixian	54115
Inner Mongolia Mandula	53149
Inner Mongolia Manzhouli	50514
Inner Mongolia Moulidawawoer	50645
Inner Mongolia Narenbaolige	53083
Inner Mongolia Neiman	54223
Inner Mongolia Ningchengxian	54320
Inner Mongolia Qinglongshan	54132
Inner Mongolia Qingshuihexian	53562
Inner Mongolia Shangdu	53385
Inner Mongolia Shebotu	54039
Inner Mongolia Siziwang	53362
Inner Mongolia Suolun	50834
Inner Mongolia Taibushiqi	54305
Inner Mongolia Tongliao	54135
Inner Mongolia Tulihe	50434

Inner Mongolia Tumutezuoqi	53464
Inner Mongolia Tuoketuoxian	53467
Inner Mongolia Tuquan	50934
Inner Mongolia Tuyouqi	53455
Inner Mongolia Wengniuteqi	54213
Inner Mongolia Wuchuanxian	53368
Inner Mongolia Wuhai	53512
Inner Mongolia Wulagai	50913
Inner Mongolia Wulateqianqi	53433
Inner Mongolia Wulatezhongqi	53336
Inner Mongolia Wushenqi	53644
Inner Mongolia Wuyuan	53337
Inner Mongolia Wuzhumuqindong	50915
Inner Mongolia Xianghuangqi	53289
Inner Mongolia Xiaoergou	50548
Inner Mongolia Xilamuren	53367
Inner Mongolia Xilinhaote	54102
Inner Mongolia Xinbaerhuyouqi	50603
Inner Mongolia Xinbaerhuzuoqi	50618
Inner Mongolia Xinghe	53483
Inner Mongolia Xiwuzhumuqin	54012
Inner Mongolia Yakeshi	50526
Inner Mongolia Yikewusu	53522
Inner Mongolia Zhalantun	50639
Inner Mongolia Zhalute	54026
Inner Mongolia Zhenglanqi	54205
Inner Mongolia Zhengxiangbaiqi	54204
Inner Mongolia Zhungeerqi	53553
Inner Mongolia Zhuozi	53472
Jiangsu Baoying	58148
Jiangsu Binhai	58049
Jiangsu Changshu	58352
Jiangsu Chuzhou	58145
Jiangsu Dafeng	58158
Jiangsu Dantu	58252
Jiangsu Danyang	58341
Jiangsu Donghai	58036
Jiangsu Dongshan	58358
Jiangsu Dongtai	58251
Jiangsu Fengxian	58012
Jiangsu Funing	58143
Jiangsu Ganyu	58040
Jiangsu Gaochun	58339

Jiangsu Gaoyou	58241
Jiangsu Guannan	58048
Jiangsu Guanyun	58047
Jiangsu Haian	58254
Jiangsu Haimen	58360
Jiangsu Hongze	58139
Jiangsu Huaian	58141
Jiangsu Jiangdu	58244
Jiangsu Jiangyan	58250
Jiangsu Jiangyin	58351
Jiangsu Jianhu	58146
Jiangsu Jingjiang	58257
Jiangsu Jinhu	58147
Jiangsu Jintan	58342
Jiangsu Jurong	58344
Jiangsu Kunshan	58356
Jiangsu Lianshui	58140
Jiangsu Lianyungang	58044
Jiangsu Lishui	58340
Jiangsu Liuhe	58235
Jiangsu Liyang	58345
Jiangsu Lvsi	58265
Jiangsu Nanjing	58238
Jiangsu Nantong	58259
Jiangsu Peixian	58013
Jiangsu Pizhou	58026
Jiangsu Pukou	58237
Jiangsu Qidong	58269
Jiangsu Rudong	58264
Jiangsu Rugao	58255
Jiangsu Sheyang	58150
Jiangsu Shuyang	58038
Jiangsu Sihong	58135
Jiangsu Siyang	58132
Jiangsu Suining	58130
Jiangsu Suyu	58131
Jiangsu Suzhou	58349
Jiangsu Taicang	58377
Jiangsu Taixing	58249
Jiangsu Taizhou	58246
Jiangsu Tongzhou	58268
Jiangsu Wujiang	58359
Jiangsu Wuxi	58354

Jiangsu Xiangshui	58045
Jiangsu Xiliandao	58041
Jiangsu Xinghua	58243
Jiangsu Xinyi	58035
Jiangsu Xuyi	58138
Jiangsu Xuzhou	58027
Jiangsu Yancheng	58154
Jiangsu Yangzhong	58247
Jiangsu Yangzhou	58245
Jiangsu Yixing	58346
Jiangsu Yizheng	58242
Jiangsu Zhangjiagang	58353
Jiangxi Anfu	57798
Jiangxi Anyi	58602
Jiangxi Anyuan	58907
Jiangxi Chongren	58710
Jiangxi Chongyi	57990
Jiangxi Dayu	57994
Jiangxi Dean	58508
Jiangxi Dexing	58622
Jiangxi Dingnan	59093
Jiangxi Dongxiang	58618
Jiangxi Duchang	58517
Jiangxi Fengcheng	58609
Jiangxi Fengxin	58601
Jiangxi Fenyi	57792
Jiangxi Fuliang	58524
Jiangxi Ganxian	57993
Jiangxi Gaoan	58605
Jiangxi Guangchang	58813
Jiangxi Guangfeng	58635
Jiangxi Guixi	58626
Jiangxi Hengfeng	58625
Jiangxi Huichang	58906
Jiangxi Hukou	58510
Jiangxi Jianxian	57799
Jiangxi Jingan	58600
Jiangxi Jingdezhen	58527
Jiangxi Jinggangshan	57894
Jiangxi Jinxi	58712
Jiangxi Jinxian	58614
Jiangxi Jishui	58707
Jiangxi Jiujiang	58502

Jiangxi Jiujiangxian	58505
Jiangxi Lean	58706
Jiangxi Leping	58620
Jiangxi Lianhua	57789
Jiangxi Lichuan	58719
Jiangxi Linchuan	58619
Jiangxi Longnan	59092
Jiangxi Lushan	58506
Jiangxi Luxi	57794
Jiangxi Nanchang	58606
Jiangxi Nanchangxian	58607
Jiangxi Nancheng	58715
Jiangxi Nanfeng	58718
Jiangxi Nankang	57992
Jiangxi Ningdu	58806
Jiangxi Pengze	58512
Jiangxi Pingxiang	57786
Jiangxi Poyang	58519
Jiangxi Qianshan	58629
Jiangxi Quannan	59091
Jiangxi Ruichang	58503
Jiangxi Ruijin	58903
Jiangxi Shanggao	57699
Jiangxi Shangli	57783
Jiangxi Shangrao	58637
Jiangxi Shangraoxian	58623
Jiangxi Shangyou	57991
Jiangxi Shicheng	58814
Jiangxi Suichuan	57896
Jiangxi Taihe	57899
Jiangxi Tonggu	57694
Jiangxi Wanan	57895
Jiangxi Wannian	58615
Jiangxi Wanzai	57698
Jiangxi Wuning	58507
Jiangxi Wuyuan	58529
Jiangxi Xiajiang	58704
Jiangxi Xiaping	57883
Jiangxi Xinfeng	57995
Jiangxi Xingan	58701
Jiangxi Xingguo	58804
Jiangxi Xingzi	58514
Jiangxi Xinjian	58693

Jiangxi Xinyu	57796
Jiangxi Xiushui	57598
Jiangxi Xunwu	59102
Jiangxi Yichun	57793
Jiangxi Yifeng	57696
Jiangxi Yihuang	58714
Jiangxi Yingtan	58627
Jiangxi Yiyang	58624
Jiangxi Yongfeng	58705
Jiangxi Yongxin	57891
Jiangxi Yongxiu	58509
Jiangxi Yudu	58905
Jiangxi Yugan	58612
Jiangxi Yujiang	58616
Jiangxi Yushan	58634
Jiangxi Zhangshu	58608
Jiangxi Zixi	58713
Jilin Antu	54187
Jilin Baicheng	50936
Jilin Baishan	54371
Jilin Changbai	54386
Jilin Changchun	54161
Jilin Changling	54049
Jilin Daan	50945
Jilin Dehui	54065
Jilin Donggang	54284
Jilin Dunhua	54186
Jilin Erdao	54285
Jilin Fuyu	54063
Jilin Gujiazi	54155
Jilin Helong	54286
Jilin Huadian	54273
Jilin Huichun	54291
Jilin Huinan	54274
Jilin Jian	54377
Jilin Jiangyuan	54279
Jilin Jiaohe	54181
Jilin Jilin Suburb	54172
Jilin Jingyu	54276
Jilin Jiutai	54069
Jilin Liaoyuan	54260
Jilin Linjiang	54374
Jilin Lishu	54154

Jilin Liuhe	54267
Jilin Longjing	54290
Jilin Luozigou	54192
Jilin Meihekou	54266
Jilin Nongan	54064
Jilin Panshi	54263
Jilin Qianan	50948
Jilin Qianguo	50949
Jilin Shuangliao	54142
Jilin Shuangyang	54165
Jilin Shulan	54076
Jilin Siping	54157
Jilin Songyuan	50946
Jilin Taonan	50939
Jilin Tongfeng	54261
Jilin Tonghua	54363
Jilin Tonghuaxian	54362
Jilin Tongyu	54041
Jilin Tumen	54293
Jilin Wangqing	54195
Jilin Yanji	54292
Jilin Yantongshan	54169
Jilin Yitong	54164
Jilin Yongji	54171
Jilin Yushu	54072
Jilin Zhenlai	50940
Liaoning Anshan	54339
Liaoning Beipiao	54323
Liaoning Benxi	54346
Liaoning Caohekou	54483
Liaoning Changhai	54579
Liaoning Changtu	54243
Liaoning Changxingdao	54565
Liaoning Chaoyang	54324
Liaoning Dalian	54662
Liaoning Dandong	54497
Liaoning Dashiqiao	54475
Liaoning Dawa	54470
Liaoning Dengta	54348
Liaoning Donggang	54590
Liaoning Faku	54245
Liaoning Fengcheng	54494
Liaoning Fushun	54351

Liaoning Gaizhou	54474
Liaoning Jianchang	54452
Liaoning Jianpingxian	54326
Liaoning Jianpingzhen	54321
Liaoning Jinzhou	54568
Liaoning Kaiyuan	54254
Liaoning Kangping	54244
Liaoning Kazuo	54328
Liaoning Kuandian	54493
Liaoning Lianshan	54453
Liaoning Liaoyang	54347
Liaoning Liaoyangxian	54345
Liaoning Liaozhong	54332
Liaoning Lingyuan	54327
Liaoning Panshan	54338
Liaoning Pikou	54575
Liaoning Pulandian	54569
Liaoning Qingyuan	54259
Liaoning Shenbei	54248
Liaoning Shenyang	54342
Liaoning Suizhong	54454
Liaoning Sujiatun	54340
Liaoning Taian	54336
Liaoning Tieling	54249
Liaoning Wafangdian	54563
Liaoning Xifeng	54252
Liaoning Xinbin	54353
Liaoning Xingcheng	54455
Liaoning Xinmin	54333
Liaoning Xiongyue	54476
Liaoning Xiuyan	54486
Liaoning Yangshan	54325
Liaoning Yingkou	54471
Liaoning Zhangwu	54236
Liaoning Zhuanghe	54584
Ningxia Guyuan	53817
Ningxia Haiyuan	53806
Ningxia Helan	53610
Ningxia Huinong	53519
Ningxia Jingyuan	53916
Ningxia Lingwu	53619
Ningxia Liupanshan	53910
Ningxia Lunde	53914

Ningxia Maihuangshan	53727
Ningxia Pingluo	53611
Ningxia Qingtongxia	53617
Ningxia Sahu	53603
Ningxia Shitanjing	53517
Ningxia Shizuishan	53518
Ningxia Taole	53615
Ningxia Tongxin	53810
Ningxia Weizhou	53881
Ningxia Wuzhong	53612
Ningxia Xiji	53903
Ningxia Xingren	53707
Ningxia Yanchi	53723
Ningxia Yinchuan	53614
Ningxia Yongning	53618
Ningxia Zhongning	53705
Ningxia Zhongwei	53704
Qinghai Banma	56151
Qinghai Dari	56046
Qinghai Doulan	52836
Qinghai Gander	56045
Qinghai Geermu	52818
Qinghai Gonghe	52856
Qinghai Guide	52868
Qinghai Guinan	52955
Qinghai Henan	56065
Qinghai Hualong	52877
Qinghai Huangzhong	52869
Qinghai Huzhu	52863
Qinghai Jianzha	52963
Qinghai Jiuzhi	56067
Qinghai Lenghu	52602
Qinghai Maduo	56033
Qinghai Maqin	56043
Qinghai Minhe	52876
Qinghai Nangqian	56125
Qinghai Nuomuhong	52825
Qinghai Pingan	52875
Qinghai Qinghaihu 151	52854
Qinghai Qingshuihe	56034
Qinghai Qumacai	56021
Qinghai Shazhuyu	52941
Qinghai Tongde	52957

Qinghai Tongren	52974
Qinghai Tuotuohe	56004
Qinghai Wudaoliang	52908
Qinghai Xiaozaohuo	52707
Qinghai Xinghai	52943
Qinghai Xining	52866
Qinghai Xunhua	52972
Qinghai Yushu	56029
Qinghai Zaduo	56018
Qinghai Zeku	52968
Qinghai Zhiduo	56016
Shaanxi Ankang	57245
Shaanxi Ansai	53841
Shaanxi Baihe	57254
Shaanxi Baishui	53941
Shaanxi Baoji	57016
Shaanxi Baojixian	57020
Shaanxi Binxian	57023
Shaanxi Changwu	53929
Shaanxi Chengcheng	53949
Shaanxi Chenggu	57128
Shaanxi Chunhua	57031
Shaanxi Dali	57043
Shaanxi Danfeng	57153
Shaanxi Dingbian	53725
Shaanxi Fengxian	57113
Shaanxi Fengxiang	57025
Shaanxi Foping	57134
Shaanxi Fufeng	57026
Shaanxi Fugu	53567
Shaanxi Fuping	57042
Shaanxi Fuxian	53931
Shaanxi Ganquan	53848
Shaanxi Ganxian	57035
Shaanxi Hanyin	57233
Shaanxi Hanzhong	57127
Shaanxi Hengshan	53740
Shaanxi Heyang	53950
Shaanxi Huangling	53944
Shaanxi Huanglong	53946
Shaanxi Huashan	57046
Shaanxi Huayin	57055
Shaanxi Jiaxian	53658

Shaanxi Jingbian	53735
Shaanxi Jinghe	57131
Shaanxi Jingyang	57033
Shaanxi Langao	57247
Shaanxi Linyou	57022
Shaanxi Liquan	57029
Shaanxi Liuba	57124
Shaanxi Longxian	57003
Shaanxi Lueyang	57106
Shaanxi Luochuan	53942
Shaanxi Luonan	57057
Shaanxi Meixian	57027
Shaanxi Mianxian	57119
Shaanxi Mizhi	53750
Shaanxi Nanzheng	57213
Shaanxi Ningqiang	57211
Shaanxi Ningshan	57137
Shaanxi Pingli	57248
Shaanxi Pucheng	53948
Shaanxi Qianyang	57021
Shaanxi Qindu	57048
Shaanxi Qingjian	53757
Shaanxi Qishan	57024
Shaanxi Sanyuan	57041
Shaanxi Shangnan	57154
Shaanxi Shangxian	57143
Shaanxi Shanyang	57155
Shaanxi Shenmu	53651
Shaanxi Shiquan	57232
Shaanxi Suide	53754
Shaanxi Taibai	57028
Shaanxi Tongchuan	53947
Shaanxi Tongguan	57054
Shaanxi Weinan	57045
Shaanxi Wubao	53756
Shaanxi Wugong	57034
Shaanxi Wuqi	53738
Shaanxi Xingping	57038
Shaanxi Xunyang	57242
Shaanxi Xunyi	53938
Shaanxi Yanchang	53854
Shaanxi Yanchuan	53850
Shaanxi Yangling	57123

Shaanxi Yaoxian	57037
Shaanxi Yichuan	53857
Shaanxi Yijun	53945
Shaanxi Yongshou	57030
Shaanxi Yulin	53646
Shaanxi Zhashui	57140
Shaanxi Zhenam	57144
Shaanxi Zhenba	57238
Shaanxi Zhenping	57343
Shaanxi Zhidan	53832
Shaanxi Zichang	53748
Shaanxi Ziyang	57231
Shaanxi Zizhou	53751
Shandong Linyi	54938
Shandong Anqiu	54844
Shandong Binzhou	54734
Shandong Boshan	54825
Shandong Cangshan	58030
Shandong Caoxian	58002
Shandong Changle	54837
Shandong Changqing	54816
Shandong Changyi	54841
Shandong Chengshantou	54776
Shandong Chengwu	58003
Shandong Chiping	54814
Shandong Danxian	58011
Shandong Dezhou	54714
Shandong Dingtao	54909
Shandong Donge	54815
Shandong Dongming	54908
Shandong Dongping	54911
Shandong Dongying	54736
Shandong Feicheng	54819
Shandong Feixian	54929
Shandong Fushan	54764
Shandong Gaomi	54846
Shandong Gaoqing	54729
Shandong Haiyang	54863
Shandong Heze	54906
Shandong Huangdao	54943
Shandong Huantai	54833
Shandong Huimin	54725
Shandong Jiaozhou	54849

Shandong Jiaxiang	54921
Shandong Jimo	54855
Shandong Jinan	54823
Shandong Jining	54915
Shandong Jinxiang	54917
Shandong Jiyang	54821
Shandong Juancheng	54904
Shandong Junan	54939
Shandong Juxian	54936
Shandong Juye	54914
Shandong Kenli	54744
Shandong Laiwu	54828
Shandong Laixi	54851
Shandong Laiyang	54852
Shandong Laizhou	54749
Shandong Laoshan	54853
Shandong Leling	54726
Shandong Liangshan	54910
Shandong Liaocheng	54806
Shandong Lingxian	54715
Shandong Linqing	54802
Shandong Linqu	54835
Shandong Linshu	58032
Shandong Linyi	54712
Shandong Longdao	54751
Shandong Longkou	54753
Shandong Mengyin	54923
Shandong Ningjin	54716
Shandong Ningyang	54913
Shandong Penglai	54752
Shandong Pingdu	54842
Shandong Pingyi	54925
Shandong Pingyin	54818
Shandong Qihe	54812
Shandong Qingdao	54857
Shandong Qingyun	54728
Shandong Qingzhou	54831
Shandong Qixia	54759
Shandong Qufu	54918
Shandong Rizhao	54945
Shandong Rushan	54861
Shandong Shanghe	54724
Shandong Shidao	54871

Shandong Shouguang	54832
Shandong Sishui	54920
Shandong Taian	54827
Shandong Taierzhuang	58025
Shandong Taishan	54826
Shandong Tengzhou	54927
Shandong Weifang	54843
Shandong Weishan	58020
Shandong Wendeng	54777
Shandong Wenshang	54912
Shandong Wucheng	54709
Shandong Wudi	54722
Shandong Wulian	54940
Shandong Xintai	54922
Shandong Xinxian	54808
Shandong Xuechen	58021
Shandong Yanggu	54807
Shandong Yangxin	54723
Shandong Yantai	54765
Shandong Yanzhou	54916
Shandong Yicheng	58022
Shandong Yinan	54935
Shandong Yishui	54932
Shandong Yiyuan	54836
Shandong Yuncheng	54905
Shandong Yutai	54907
Shandong Zaozhuang	58024
Shandong Zhangqiu	54727
Shandong Zhaoyuan	54755
Shandong Zhoucun	54829
Shandong Zhucheng	54848
Shandong Zibo	54830
Shandong Zibo	54834
Shandong Zichuan	54824
Shandong Zoucheng	54919
Shandong Zouping	54822
Shanghai Baoshan	58362
Shanghai Chongming	58366
Shanghai Fengxian	58463
Shanghai Jiading	58365
Shanghai Jinshan	58460
Shanghai Minhang	58361
Shanghai Nanhui	58369

Shanghai Pudong	58370
Shanghai Qingpu	58461
Shanghai Songjiang	58462
Shanghai Xujiahui	58367
Shanxi Pinglu	53574
Shanxi Anze	53877
Shanxi Baode	53660
Shanxi Changzhi	53882
Shanxi Changzi	53873
Shanxi Daixian	53579
Shanxi Daning	53856
Shanxi Datong	53487
Shanxi Datongxian	53488
Shanxi Dingxiang	53676
Shanxi Fangshan	53760
Shanxi Fanshi	53585
Shanxi Fenxi	53865
Shanxi Fenyang	53769
Shanxi Fushan	53966
Shanxi Gaoping	53973
Shanxi Guangling	53590
Shanxi Gujiao	53763
Shanxi Guxian	53874
Shanxi Hejin	53957
Shanxi Hequ	53564
Shanxi Heshun	53788
Shanxi Hongtong	53866
Shanxi Houma	53963
Shanxi Huguan	53885
Shanxi Hunyuan	53582
Shanxi Huozhou	53869
Shanxi Jiancaoping	53677
Shanxi Jiangxian	53965
Shanxi Jiaocheng	53777
Shanxi Jiaokou	53860
Shanxi Jiexiu	53863
Shanxi Jincheng	53976
Shanxi Jingle	53666
Shanxi Jishan	53954
Shanxi Jixian	53859
Shanxi Kelan	53662
Shanxi Lanxian	53665
Shanxi Licheng	53878

Shanxi Linfen	53868
Shanxi Lingchuan	53981
Shanxi Lingqiu	53594
Shanxi Lingshi	53862
Shanxi Linxian	53659
Shanxi Linyi	53958
Shanxi Lishi	53764
Shanxi Liulin	53753
Shanxi Loufan	53669
Shanxi Lucheng	53880
Shanxi Ningwu	53577
Shanxi Pianguan	53565
Shanxi Pingding	53687
Shanxi Pinglu	57061
Shanxi Pingshun	53888
Shanxi Pingyao	53778
Shanxi Puxian	53864
Shanxi Qingshui	53970
Shanxi Qingxu	53774
Shanxi Qinxian	53872
Shanxi Qinyuan	53875
Shanxi Qixian	53770
Shanxi Quwo	53961
Shanxi Ruicheng	57053
Shanxi Shanyin	53576
Shanxi Shenchi	53575
Shanxi Shilou	53759
Shanxi Shouyang	53780
Shanxi Shuozhou	53578
Shanxi Taigu	53775
Shanxi Taiyuan	53772
Shanxi Tianzhen	53490
Shanxi Tunliu	53879
Shanxi Wanrong	53956
Shanxi Wenshui	53771
Shanxi Wenxi	53967
Shanxi Wutaishan	53588
Shanxi Wutaixian	53681
Shanxi Wuxiang	53871
Shanxi Wuzhai	53663
Shanxi Xiangfen	53861
Shanxi Xiangning	53953
Shanxi Xiangyuan	53884

Shanxi Xiaodian	53679
Shanxi Xiaoyi	53768
Shanxi Xiaxian	57060
Shanxi Xinfu	53674
Shanxi Xingxian	53664
Shanxi Xinjiang	53964
Shanxi Xixian	53853
Shanxi Xiyang	53783
Shanxi Yangcheng	53975
Shanxi Yanggao	53486
Shanxi Yangq2u	53678
Shanxi Yangquan	53782
Shanxi Yanhu	53959
Shanxi Yicheng	53962
Shanxi Yingxian	53584
Shanxi Yonghe	53852
Shanxi Yongji	57052
Shanxi Youyu	53478
Shanxi Yuanping	53673
Shanxi Yuanqu	53968
Shanxi Yushe	53787
Shanxi Yuxian	53685
Shanxi Zhongyang	53767
Shanxi Zuoquan	53786
Shanxi Zuoyun	53573
Sichuan Aba	56171
Sichuan Anyue	57408
Sichuan Baiyu	56147
Sichuan Baoxing	56273
Sichuan Batang	56247
Sichuan Bazhong	57313
Sichuan Butuo	56580
Sichuan Cangxi	57303
Sichuan Changning	56593
Sichuan Chaojue	56479
Sichuan Chongzhou	56181
Sichuan Dachuan	57328
Sichuan Danba	56263
Sichuan Danling	56381
Sichuan Daocheng	56357
Sichuan Daofu	56167
Sichuan Dayi	56285
Sichuan Dazhu	57420

Sichuan Dechang	56569
Sichuan Dege	56144
Sichuan Derong	56441
Sichuan Deyang	56198
Sichuan Dongxing	57503
Sichuan Dujiangyan	56188
Sichuan Ebian	56387
Sichuan Emei	56384
Sichuan Emeishan	56385
Sichuan Fushun	56399
Sichuan Ganluo	56473
Sichuan Ganzi	56146
Sichuan Gaoping	57411
Sichuan Gaoxian	56592
Sichuan Gongxian	56499
Sichuan Guangan	57415
Sichuan Guangyuan	57206
Sichuan Hanyuan	56376
Sichuan Heishui	56185
Sichuan Hejiang	57603
Sichuan Hongya	56380
Sichuan Hongyuan	56173
Sichuan Huidong	56675
Sichuan Huili	56671
Sichuan Jiajiang	56382
Sichuan Jiangan	57600
Sichuan Jiange	57208
Sichuan Jinchuan	56168
Sichuan Jingyan	56390
Sichuan Jintang	56296
Sichuan Jinyang	56584
Sichuan Jiulong	56462
Sichuan Jiuzhaigou	56097
Sichuan Junlian	56498
Sichuan Kaijiang	57329
Sichuan Kangding	56374
Sichuan Langzhong	57306
Sichuan Leibo	56485
Sichuan Leshan	56386
Sichuan Lezhi	57407
Sichuan Linshui	57416
Sichuan Litang	56257
Sichuan Lixian	56184

Sichuan Longchang	57507
Sichuan Longquanyi	56286
Sichuan Luding	56371
Sichuan Luhuo	56158
Sichuan Lushan	56279
Sichuan Luxian	57508
Sichuan Mabian	56480
Sichuan Maerkang	56172
Sichuan Maoxian	56180
Sichuan Meigu	56487
Sichuan Meishan	56391
Sichuan Mianning	56474
Sichuan Mianzhu	56186
Sichuan Mingshan	56280
Sichuan Miyi	56670
Sichuan Muchuan	56490
Sichuan Muli	56459
Sichuan Nanbu	57314
Sichuan Nanjiang	57216
Sichuan Nanxi	56493
Sichuan Ningnan	56578
Sichuan Panzhihua	56666
Sichuan Pengan	57317
Sichuan Pengshan	56289
Sichuan Pengxi	57402
Sichuan Pengzhou	56189
Sichuan Pingchang	57324
Sichuan Pingshan	56494
Sichuan Pixian	56272
Sichuan Puge	56575
Sichuan Pujiang	56281
Sichuan Qianwei	56389
Sichuan Qingchuan	57204
Sichuan Qingshen	56383
Sichuan Qionglai	56284
Sichuan Quxian	57413
Sichuan Rangtang	56164
Sichuan Renhe	56674
Sichuan Renshou	56297
Sichuan Rongxian	56394
Sichuan Ruoergai	56079
Sichuan Santai	57307
Sichuan Seda	56152

Sichuan Shehong	57401
Sichuan Shifang	56197
Sichuan Shimian	56378
Sichuan Shiqu	56038
Sichuan Shuangliu	56288
Sichuan Songpan	56182
Sichuan Suining	57405
Sichuan Tianquan	56278
Sichuan Tongjiang	57320
Sichuan Wangcang	57217
Sichuan Wanyuan	57237
Sichuan Weiyuan	56395
Sichuan Wenchuan	56183
Sichuan Wenjiang	56187
Sichuan Wusheng	57417
Sichuan Xiangcheng	56443
Sichuan Xiaojin	56178
Sichuan Xichang	56571
Sichuan Xichong	57309
Sichuan Xide	56478
Sichuan Xindu	56290
Sichuan Xingwen	56496
Sichuan Xinjin	56276
Sichuan Xinlong	56251
Sichuan Xuanhan	57326
Sichuan Xuyong	57608
Sichuan Yajiang	56267
Sichuan Yanbian	56665
Sichuan Yanting	57308
Sichuan Yanyuan	56565
Sichuan Yibin	56492
Sichuan Yibinxian	56491
Sichuan Yilong	57315
Sichuan Yingjing	56373
Sichuan Yingshan	57318
Sichuan Yuechi	57414
Sichuan Yuexi	56475
Sichuan Zhongjiang	56199
Sichuan Zigong	56396
Sichuan Ziyang	56298
Sichuan Zizhong	56393
Tianjin Baodi	54525
Tianjin Beichen	54528

Tianjin Dagang	54645
Tianjin Dongli	54526
Tianjin Hangu	54530
Tianjin Jinghai	54619
Tianjin Jinnan	54622
Tianjin Jixian	54428
Tianjin Ninghe	54529
Tianjin Tanggu	54623
Tianjin Tianjin	54527
Tianjin Wuqing	54523
Tibet Basu	56228
Tibet Bomi	56227
Tibet Changdu	56137
Tibet Chayu	56434
Tibet Dangxiong	55493
Tibet Dingqing	56116
Tibet Dingri	55664
Tibet Gaize	55248
Tibet Jiangzi	55680
Tibet Lazi	55569
Tibet Leiwuqi	56128
Tibet Linzhi	56312
Tibet Luolong	56223
Tibet Miling	56317
Tibet Muozhugongka	55593
Tibet Nanmulin	55572
Tibet Naqu	55299
Tibet Nielamu	55655
Tibet Pulan	55437
Tibet Qiongjie	55597
Tibet Shiquanhe	55228
Tibet Zedang	55598
Tibet Zuogong	56331
Xinjiang Akedala	51058
Xinjiang Akesu	51628
Xinjiang Alaer	51730
Xinjiang Alashankou	51232
Xinjiang Aletai	51076
Xinjiang Atushi	51704
Xinjiang Awati	51722
Xinjiang Balikun	52101
Xinjiang Bayinbuluke	51542
Xinjiang Bole	51238

Xinjiang Buerjin	51060
Xinjiang Cele	51826
Xinjiang Changji	51368
Xinjiang Dabancheng	51477
Xinjiang Emin	51145
Xinjiang Fuhai	51068
Xinjiang Fukang	51377
Xinjiang Fuyun	51087
Xinjiang Habahe	51053
Xinjiang Hami	52203
Xinjiang Hebukesaier	51156
Xinjiang Hetan	51828
Xinjiang Hongliuhe	52313
Xinjiang Hutubi	51367
Xinjiang Jiashi	51707
Xinjiang Jimusaer	51378
Xinjiang Jinghe	51334
Xinjiang Kashi	51709
Xinjiang Kelamayi	51243
Xinjiang Kepin	51720
Xinjiang Kuerle	51656
Xinjiang Kumishi	51526
Xinjiang Luopu	51829
Xinjiang Maigaiti	51810
Xinjiang Manasi	51359
Xinjiang Minfeng	51839
Xinjiang Miquan	51369
Xinjiang Moyu	51827
Xinjiang Mulei	51482
Xinjiang Naomaohu	52112
Xinjiang Pishan	51818
Xinjiang Qiemuo	51855
Xinjiang Shashe	51811
Xinjiang Shawan	51357
Xinjiang Shaya	51639
Xinjiang Tacheng	51133
Xinjiang Tazhong	51747
Xinjiang Tianchi	51470
Xinjiang Tianshandaxigou	51468
Xinjiang Tieqianlike	51765
Xinjiang Touli	51241
Xinjiang Tulufan	51573
Xinjiang Tulufandongkan	51572

Xinjiang Tuokexun	51571
Xinjiang Urumqi Pastoral Test Station	51469
Xinjiang Wuqia	51705
Xinjiang Wushi	51627
Xinjiang Xinhe	51636
Xinjiang Yanqi	51567
Xinjiang Yecheng	51814
Xinjiang Yengjisha	51802
Xinjiang Yiwu	52118
Xinjiang Yuepuhu	51717
Xinjiang Yumin	51137
Xinjiang Yutian	51931
Xinjiang Zepu	51815
Yuci Shanxi	53776
Yunnan Yiliang	56880
Yunnan Anning	56863
Yunnan Baoshan	56748
Yunnan Binchuan	56752
Yunnan Cangyuan	56944
Yunnan Changning	56843
Yunnan Chenggong	56882
Yunnan Chengjiang	56873
Yunnan Daguan	56582
Yunnan Dali	56751
Yunnan Dayao	56761
Yunnan Deqin	56444
Yunnan Dongchuan	56688
Yunnan Eryuan	56649
Yunnan Eshan	56898
Yunnan Fengqing	56846
Yunnan Fugong	56641
Yunnan Fumin	56772
Yunnan Funing	59205
Yunnan Fuyuan	56790
Yunnan Gejiu	56984
Yunnan Gengma	56946
Yunnan Gongshan	56533
Yunnan Guangnan	59007
Yunnan Heqing	56654
Yunnan Honghe	56975
Yunnan Huaning	56879
Yunnan Huaping	56664
Yunnan Huize	56684

Yunnan Jianchuan	56646
Yunnan Jiangcheng	56977
Yunnan Jiangchuan	56876
Yunnan Jingdong	56856
Yunnan Jinggu	56952
Yunnan Jinning	56871
Yunnan Jinping	56987
Yunnan Kaiyuan	56982
Yunnan Kunming	56778
Yunnan Lancang	56954
Yunnan Lanping	56645
Yunnan Lianghe	56840
Yunnan Lijiang	56651
Yunnan Lincang	56951
Yunnan Liuku	56643
Yunnan Longchuan	56835
Yunnan Longling	56841
Yunnan Luchun	56978
Yunnan Ludian	56585
Yunnan Lufeng	56777
Yunnan Luoping	56891
Yunnan Luxi	56886
Yunnan Maguan	56995
Yunnan Malong	56782
Yunnan Mangshi	56844
Yunnan Menghai	56958
Yunnan Mengla	56969
Yunnan Menglian	56949
Yunnan Midu	56755
Yunnan Mile	56885
Yunnan Mojiang	56962
Yunnan Mouding	56766
Yunnan Nanhua	56767
Yunnan Nanjian	56851
Yunnan Ninglang	56567
Yunnan Qiaojia	56673
Yunnan Qiubei	56889
Yunnan Qujing	56783
Yunnan Shidian	56842
Yunnan Shilin	56881
Yunnan Shiping	56970
Yunnan Shizong	56883
Yunnan Shuangbai	56862

Yunnan Shuangjiang	56950
Yunnan Simao	56964
Yunnan Songming	56785
Yunnan Suijiang	56483
Yunnan Tengchong	56739
Yunnan Tonghai	56878
Yunnan Weishan	56757
Yunnan Weixi	56548
Yunnan Weixin	56596
Yunnan Wenshan	56994
Yunnan Wuding	56774
Yunnan Xianggelila	56543
Yunnan Xichou	56992
Yunnan Ximeng	56948
Yunnan Xinping	56869
Yunnan Xuanwei	56697
Yunnan Yangbi	56745
Yunnan Yanjin	56497
Yunnan Yanshan	56991
Yunnan Yaoan	56764
Yunnan Yiliang	56594
Yunnan Yimen	56870
Yunnan Yingjiang	56836
Yunnan Yongde	56849
Yunnan Yongping	56746
Yunnan Yongren	56669
Yunnan Yongshan	56489
Yunnan Yongsheng	56652
Yunnan Yuanjiang	56966
Yunnan Yuanyang	56976
Yunnan Yunlong	56742
Yunnan Yunxian	56854
Yunnan Yuxi	56875
Yunnan Zhenkang	56839
Yunnan Zhenxiong	56595
Yunnan Zhenyuan	56867
Zhaotong Yunnan	56586
Zhejiang Anji	58446
Zhejiang Beilun	58563
Zhejiang Changshan	58631
Zhejiang Changxing	58443
Zhejiang Chunan	58543
Zhejiang Cixi	58467

Zhejiang Dachen	58666
Zhejiang Daishan	58484
Zhejiang Deqing	58454
Zhejiang Dinghai	58477
Zhejiang Dongyang	58558
Zhejiang Fenghua	58565
Zhejiang Fuyang	58449
Zhejiang Haining	58455
Zhejiang Haiyan	58458
Zhejiang Hangzhou	58457
Zhejiang Hongjia	58665
Zhejiang Huzhou	58450
Zhejiang Jiande	58544
Zhejiang Jiangshan	58632
Zhejiang Jiashan	58451
Zhejiang Jiaxing	58452
Zhejiang Jingning	58648
Zhejiang Jinhua	58549
Zhejiang Jinyun	58654
Zhejiang Kaihua	58537
Zhejiang Lanxi	58548
Zhejiang Leqing	58656
Zhejiang Linan	58448
Zhejiang Linhai	58660
Zhejiang Lishui	58646
Zhejiang Longquan	58647
Zhejiang Longyou	58547
Zhejiang Ninghai	58567
Zhejiang Panan	58560
Zhejiang Pinghu	58464
Zhejiang Pingyang	58751
Zhejiang Pujiang	58546
Zhejiang Putuo	58570
Zhejiang Qingtian	58657
Zhejiang Qingyuan	58745
Zhejiang Ruian	58752
Zhejiang Sanmen	58568
Zhejiang Shangyu	58553
Zhejiang Shaoxing	58453
Zhejiang Shengsi	58472
Zhejiang Shengzhou	58556
Zhejiang Shipu	58569
Zhejiang Suichang	58644

Zhejiang Taishun	58746
Zhejiang Tiantai	58559
Zhejiang Tonglu	58542
Zhejiang Tongxiang	58456
Zhejiang Wencheng	58750
Zhejiang Wenling	58664
Zhejiang Wenzhou	58659
Zhejiang Wuyi	58642
Zhejiang Xiangshan	58566
Zhejiang Xianju	58652
Zhejiang Xiaoshan	58459
Zhejiang Xinchang	58555
Zhejiang Yinzhou	58562
Zhejiang Yiwu	58557
Zhejiang Yongjia	58658
Zhejiang Yongkang	58643
Zhejiang Yuhuan	58667
Zhejiang Yunhe	58742
Zhejiang Yuyao	58468
Zhejiang Zhenhai	58561
Zhejiang Zhuji	58550

Table S2. 1744 monitoring stations whose PM2.5 emissions are from industrial sector

58015	Anhui Dangshan
58016	Anhui Xiaoxian
58102	Anhui Bozhou
58108	Anhui Jieshou
58109	Anhui Taihe
58112	Anhui Tianzhushan
58113	Anhui Suixi
58114	Anhui Woyang
58117	Anhui Leysin
58118	Anhui Mengcheng
58122	Anhui Suzhou
58125	Anhui Lingbi
58126	Anhui Sixian
58127	Anhui Huaiyuan
58128	Anhui Guzhen
58129	Anhui Wuhe
58202	Anhui Funan
58203	Anhui Fuyang
58210	Anhui Yingshang
58212	Anhui Fengtai
58214	Anhui Huoqiu
58220	Anhui Changfeng
58222	Anhui Fengyang
58223	Anhui Mingguang
58225	Anhui Dingyuan
58234	Anhui Laian
58236	Anhui Chuzhou
58240	Anhui Tianchang
58306	Anhui Jinzhai
58311	Anhui Luan
58314	Anhui Huoshan
58316	Anhui Shucheng
58317	Anhui Yuexi
58319	Anhui Tongcheng
58320	Anhui Feixi
58323	Anhui Feidong
58326	Anhui Chaohu
58327	Anhui Lujiang
58329	Anhui Wuwei
58330	Anhui Hanshan

58334	Anhui Wuhu
58336	Anhui Maanshan
58338	Anhui Wuhuxian
58417	Anhui Susong
58429	Anhui Tongling
58431	Anhui Nanling
58432	Anhui Jingxian
58433	Anhui Xuancheng
58435	Anhui Jingde
58436	Anhui Ningguo
58437	Anhui Huangshan
58441	Anhui Guangde
58442	Anhui Langxi
58520	Anhui Qimen
58523	Anhui Yixian
58530	Anhui Shexian
58531	Anhui Tunxi
54398	Beijing Shunyi
54399	Beijing Haidian
54406	Beijing Yanqing
54416	Beijing Miyun
54421	Beijing Miyunshangdianzi
54424	Beijing Pinggu
54431	Beijing Tongzhou
54499	Beijing Changping
54501	Beijing Zhaitang
54511	Beijing
54597	Beijing Xiayunling
58724	Fujian Guangze
58725	Fujian Shaowu
58730	Fujian Wuyishan
58731	Fujian Pucheng
58734	Fujian Jianyang
58735	Fujian Songxi
58736	Fujian Zhenghe
58744	Fujian Shouning
58747	Fujian Zhouning
58748	Fujian Fuan
58749	Fujian Zherong
58754	Fujian Fuding
58818	Fujian Ninghuai
58820	Fujian Taining
58822	Fujian Jianning

58823	Fujian Shunchang
58824	Fujian Mingxi
58828	Fujian Sanming
58836	Fujian Gutian
58837	Fujian Youxi
58839	Fujian Shuqing
58843	Fujian Xiapu
58844	Fujian Minhou
58846	Fujian Ningde
58848	Fujian Lianjiang
58911	Fujian Changting
58917	Fujian Wuping
58923	Fujian Datian
58926	Fujian Zhangping
58928	Fujian Huaan
58929	Fujian Anxi
58931	Fujian Jiuxianshan
58932	Fujian Yongtai
58938	Fujian Xiuyu
58941	Fujian Changle
58942	Fujian Fuqing
58944	Fujian Pingtan
58946	Fujian Putian
59113	Fujian Yongding
59122	Fujian Changtai
59124	Fujian Nanjing
59125	Fujian Pinghe
59129	Fujian Zhangpu
59130	Fujian Tongan
59131	Fujian Nanan
59133	Fujian Chongwu
59320	Fujian Zhaoan
59322	Fujian Yunxiao
52323	Gansu Mazongshan
52515	Gansu Subei
52533	Gansu Jiuquan
52546	Gansu Gaitai
52557	Gansu Linze
52643	Gansu Sunan
52656	Gansu Minle
52674	Gansu Yongchang
52679	Gansu Wuwei
52681	Gansu Minqin

52784	Gansu Gulang
52787	Gansu Wushaoling
52797	Gansu Jingtai
52881	Gansu Tianzhu
52884	Gansu Gaolan
52885	Gansu Yongdeng
52895	Gansu Jingyuan
52896	Gansu Baiyin
52978	Gansu Xiahe
52980	Gansu Yongjing
52981	Gansu Dongxiang
52982	Gansu Guanghe
52983	Gansu Yuzhong
52985	Gansu Hezheng
52986	Gansu Lintao
52988	Gansu Kangle
52993	Gansu Huining
52995	Gansu Anding
52996	Gansu Huajialing
52998	Gansu Weiyuan
53821	Gansu Huanxian
53829	Gansu Qingcheng
53906	Gansu Jingning
53915	Gansu Kongtong
53917	Gansu Zhuanglang
53923	Gansu Xifeng
53924	Gansu Lingtai
53925	Gansu Zhenyuan
53926	Gansu Jingchuan
53927	Gansu Huating
53930	Gansu Huachi
53934	Gansu Huishui
53935	Gansu Zhengning
56071	Gansu Luqu
56074	Gansu Maqu
56080	Gansu Hezuo
56081	Gansu Lintan
56082	Gansu Zhuoni
56084	Gansu Dibu
56091	Gansu Zhangxian
56092	Gansu Longxi
56093	Gansu Minxian
56094	Gansu Zhouqu

56095	Gansu Dangchang
56096	Gansu Wudu
56192	Gansu Wenxian
57001	Gansu Gangu
57002	Gansu Qinan
57004	Gansu Wushan
57006	Gansu Tianshui
57007	Gansu Lixian
57011	Gansu Qingshui
57012	Gansu Zhangjiachuan
57014	Gansu Maiji
57102	Gansu Chengxian
57105	Gansu Kangxian
57110	Gansu Huixian
57111	Gansu Liangdang
57988	Guangdong Lechang
57989	Guangdong Renhua
57996	Guangdong Nanxiong
59074	Guangdong Lianshan
59075	Guangdong Yangshan
59081	Guangdong Ruyuan
59082	Guangdong Shaoguan
59087	Guangdong Fogang
59088	Guangdong Yingde
59090	Guangdong Shixing
59094	Guangdong Wengyuan
59096	Guangdong Lianping
59099	Guangdong Heping
59106	Guangdong Pingyuan
59107	Guangdong Longchuan
59109	Guangdong Xingning
59114	Guangdong Jiaoling
59116	Guangdong Daipu
59117	Guangdong Meixian
59268	Guangdong Yunan
59270	Guangdong Huaiji
59271	Guangdong Guangning
59276	Guangdong Sihui
59279	Guangdong Sanshui
59280	Guangdong Qingyuan
59284	Guangdong Huadu
59285	Guangdong Conghua
59287	Guangdong Guangzhou

59289	Guangdong Dongguan
59290	Guangdong Longmen
59293	Guangdong Heyuan
59297	Guangdong Boluo
59303	Guangdong Wuhua
59304	Guangdong Zijin
59306	Guangdong Jiexi
59310	Guangdong Fengshun
59312	Guangdong Chaozhou
59313	Guangdong Raoping
59314	Guangdong Puning
59316	Guangdong Shantou
59456	Guangdong Xinyi
59462	Guangdong Luoding
59469	Guangdong Yangchun
59470	Guangdong Xinxing
59471	Guangdong Yunfu
59473	Guangdong Heshan
59475	Guangdong Kaiping
59476	Guangdong Xinhui
59477	Guangdong Enping
59481	Guangdong Panyu
59487	Guangdong Doumen
59488	Guangdong Zhuhai
59492	Guangdong Huidong
59493	Guangdong Shenzhen
59500	Guangdong Haifeng
59502	Guangdong Lufeng
59650	Guangdong Suixi
59653	Guangdong Gaozhou
59654	Guangdong Lianjiang
59655	Guangdong Huazhou
59656	Guangdong Wuchuan
59659	Guangdong Maoming
59750	Guangdong Leizhou
59754	Guangdong Xuwen
57859	Guangxi Ziyuan
57927	Guangxi Tiane
57941	Guangxi Sanjiang
57942	Guangxi Longsheng
57948	Guangxi Rongshui
57949	Guangxi Yongfu
57954	Guangxi Lingui

57955	Guangxi Xingan
57960	Guangxi Quanzhou
57964	Guangxi Guanyang
59004	Guangxi Xilin
59012	Guangxi Leye
59015	Guangxi Lingyun
59017	Guangxi Tianlin
59021	Guangxi Fengshan
59023	Guangxi Hechi
59027	Guangxi Bama
59034	Guangxi Yizhou
59037	Guangxi Duan
59038	Guangxi Xincheng
59041	Guangxi Liucheng
59045	Guangxi Luzhai
59053	Guangxi Pingle
59055	Guangxi Lipu
59057	Guangxi Jinxiu
59058	Guangxi Mengshan
59065	Guangxi Hezhou
59209	Guangxi Napo
59211	Guangxi Baise
59215	Guangxi Debao
59218	Guangxi Jingxi
59224	Guangxi Tiandong
59227	Guangxi Tiandeng
59229	Guangxi Longan
59230	Guangxi Mashan
59235	Guangxi Shanglin
59237	Guangxi Wuming
59238	Guangxi Binyang
59241	Guangxi Xiangzhou
59242	Guangxi Laibin
59246	Guangxi Wuxuan
59255	Guangxi Pingnan
59256	Guangxi Tengxian
59265	Guangxi Wuzhou
59266	Guangxi Cangwu
59419	Guangxi Pingxiang
59421	Guangxi Daxin
59429	Guangxi Shangsi
59431	Guangxi Nanning
59435	Guangxi Yongning

59441	Guangxi Hengxian
59446	Guangxi Lingshan
59448	Guangxi Pubei
59449	Guangxi Bobai
59451	Guangxi Beiliu
59452	Guangxi Rongxian
59454	Guangxi Cenxi
59457	Guangxi Luchuan
59632	Guangxi Qinzhou
59640	Guangxi Hepu
56598	Guizhou Hezhang
56691	Guizhou Weining
56792	Guizhou Puan
56793	Guizhou Panxian
57606	Guizhou Tongzi
57623	Guizhou Daozhen
57625	Guizhou Zhengan
57634	Guizhou Wuchuan
57636	Guizhou Yanhe
57637	Guizhou Dejiang
57647	Guizhou Songtao
57707	Guizhou Bijie
57708	Guizhou Dafang
57714	Guizhou Jinsha
57717	Guizhou Zunyi
57718	Guizhou Xifeng
57719	Guizhou Kaiyang
57722	Guizhou Meitan
57723	Guizhou Fenggang
57728	Guizhou Wengan
57729	Guizhou Yuqing
57731	Guizhou Sinan
57734	Guizhou Shiqian
57735	Guizhou Cengong
57736	Guizhou Jiangkou
57737	Guizhou Shibing
57739	Guizhou Yuping
57742	Guizhou Wanshan
57800	Guizhou Nayong
57803	Guizhou Xianxi
57805	Guizhou Zhijin
57806	Guizhou Anshun
57807	Guizhou Liuzhi

57811	Guizhou Xiuwen
57814	Guizhou Pingba
57821	Guizhou Fuquan
57822	Guizhou Huangping
57824	Guizhou Guiding
57825	Guizhou Kaili
57827	Guizhou Duyun
57832	Guizhou Sansui
57834	Guizhou Taijiang
57835	Guizhou Jianhe
57837	Guizhou Leishan
57839	Guizhou Liping
57840	Guizhou Tianzhu
57844	Guizhou Jinping
57900	Guizhou Qinglong
57903	Guizhou Guanling
57905	Guizhou Zhenfeng
57906	Guizhou Wangmo
57907	Guizhou Xingyi
57909	Guizhou Ceheng
57910	Guizhou Ziyun
57912	Guizhou Huishui
57913	Guizhou Longli
57916	Guizhou Luodian
57921	Guizhou Pingtang
57922	Guizhou Dushan
57923	Guizhou Sandu
57926	Guizhou Libo
57932	Guizhou Rongjiang
57936	Guizhou Congjiang
59758	Hainan Haikou
59842	Hainan Lingao
59843	Hainan Chengmai
59845	Hainan Danzhou
59847	Hainan Changjiang
59848	Hainan Baisha
59851	Hainan Dingan
59854	Hainan Tunchang
59855	Hainan Qonghai
59856	Hainan Wenchang
59945	Hainan Baoting
59951	Hainan Wanning
53392	Hebei Kangbao

53397	Hebei Shangyi
53399	Hebei Zhangbei
53491	Hebei Huaian
53492	Hebei Yangyuan
53498	Hebei Xuanhua
53499	Hebei Wanquan
53593	Hebei Weixian
53596	Hebei Shunping
53599	Hebei Laiyuan
53680	Hebei Lingshou
53682	Hebei Quyang
53688	Hebei Xingtang
53689	Hebei Jinzhou
53690	Hebei Fuping
53692	Hebei Tangxian
53696	Hebei Dingzhou
53699	Hebei Wuji
53773	Hebei Linzhang
53781	Hebei Shahe
53785	Hebei Baixiang
53789	Hebei Luancheng
53794	Hebei Longyao
53795	Hebei Zanhuang
53796	Hebei Ningjin
53799	Hebei Julu
53886	Hebei Shexian
53890	Hebei Wuan
53892	Hebei Handan
53893	Hebei Quzhou
53894	Hebei Fengfeng
53896	Hebei Weixian
54301	Hebei Guyuan
54304	Hebei Chongli
54308	Hebei Fengning
54311	Hebei Weichang
54318	Hebei Longhua
54319	Hebei Pingquan
54401	Hebei Zhangjiakou
54404	Hebei Chicheng
54405	Hebei Huailai
54408	Hebei Zhulu
54420	Hebei Luanping
54423	Hebei Chengde

54425	Hebei Xinglong
54429	Hebei Zunhua
54430	Hebei Chengdexian
54432	Hebei Kuancheng
54434	Hebei Qianxi
54436	Hebei Qinglong
54437	Hebei Luannan
54438	Hebei Lulong
54439	Hebei Qianan
54449	Hebei Qinhuangdao
54502	Hebei Zhuozhou
54503	Hebei Rongcheng
54506	Hebei Gaobeidian
54510	Hebei Dachang
54512	Hebei Guan
54519	Hebei Yongqing
54520	Hebei Sanhe
54521	Hebei Xianghe
54522	Hebei Yutian
54534	Hebei Tangshan
54535	Hebei Caofeidian
54539	Hebei Leting
54540	Hebei Changli
54541	Hebei Funing
54601	Hebei Xushui
54603	Hebei Goyang
54605	Hebei Anxin
54606	Hebei Raoyang
54608	Hebei Shenzhou
54610	Hebei Renqiu
54612	Hebei Wenan
54613	Hebei Dacheng
54614	Hebei Hejian
54615	Hebei Qingxian
54616	Hebei Cangzhou
54618	Hebei Botou
54624	Hebei Huanghua
54628	Hebei Haixing
54631	Hebei Guangzong
54633	Hebei Xinhe
54640	Hebei Jize
54700	Hebei Wuqiang
54701	Hebei Xinji

54702	Hebei Hengshui
54703	Hebei Wuyi
54706	Hebei Qinghe
54707	Hebei Gucheng
54710	Hebei Fucheng
54711	Hebei Jingxian
54713	Hebei Dongguang
54800	Hebei-Weixian
54801	Hebei Linxi
54804	Hebei Daming
54809	Hebei Guantao
53889	Henan Linzhou
53972	Henan Qinyang
53974	Henan Qixian
53978	Henan Jiyuan
53982	Henan Jiaozuo
53983	Henan Fengqiu
53984	Henan Xiuwu
53985	Henan Huixian
53986	Henan Xinxiang
53991	Henan Tangyin
53992	Henan Junxian
53993	Henan Neihuang
53998	Henan Changheng
54817	Henan Taiqian
54900	Henan Puyang
54902	Henan Qingfeng
54903	Henan Fanxian
57051	Henan Sanmenxia
57056	Henan Lingbao
57063	Henan Mianchi
57066	Henan Luoning
57070	Henan Xinan
57071	Henan Mengjin
57072	Henan Mengzhou
57074	Henan Yichuan
57075	Henan Ruzhou
57078	Henan Ruyang
57079	Henan Wenxian
57080	Henan Gongyi
57081	Henan Xingyang
57082	Henan Dengfeng
57087	Henan Changge

57089	Henan Xuchang
57090	Henan Zhongmou
57091	Henan Kaifeng
57093	Henan Lankao
57095	Henan Yanling
57096	Henan Qixian
57099	Henan Taikang
57156	Henan Xixia
57169	Henan Neixiang
57173	Henan Lushan
57175	Henan Zhenping
57176	Henan Nanzhao
57177	Henan Wugang
57178	Henan Nanyang
57179	Henan Fangcheng
57180	Henan Jiaxian
57182	Henan Xiangcheng
57183	Henan Linying
57184	Henan Yexian
57185	Henan Wuyang
57186	Henan Luohe
57187	Henan Sheqi
57188	Henan Xiping
57189	Henan Suiping
57192	Henan Huaiyang
57193	Henan Xihua
57197	Henan Runan
57271	Henan Xinye
57273	Henan Tanghe
57281	Henan Biyang
57285	Henan Tongbai
57290	Henan Zhumadian
57292	Henan Pingyu
57293	Henan Xincai
57295	Henan Zhengyang
57296	Henan Xixian
57297	Henan Xinyang
57299	Henan Guangshan
57390	Henan Jigongshan
57396	Henan Xixian
58004	Henan Minquan
58005	Henan Shangqiu
58006	Henan Yucheng

58007	Henan Echeng
58017	Henan Xiayi
58100	Henan Dancheng
58207	Henan Huangchuan
58208	Henan Gushi
58301	Henan Shangcheng
50136	Heilongjiang Mohe
50137	Heilongjiang Beijicun
50246	Heilongjiang Tahe
50247	Heilongjiang Huzhong
50349	Heilongjiang Xinlin
50353	Heilongjiang Huma
50442	Heilongjiang Jiagedaqi
50468	Heilongjiang Aihui
50557	Heilongjiang Nenjiang
50564	Heilongjiang Sunwu
50566	Heilongjiang Xunke
50646	Heilongjiang Nehe
50655	Heilongjiang Wudalianchi
50656	Heilongjiang Beian
50658	Heilongjiang Keshan
50659	Heilongjiang Kedong
50673	Heilongjiang Jiayin
50674	Heilongjiang Wuyiling
50739	Heilongjiang Longjiang
50741	Heilongjiang Gannan
50742	Heilongjiang Fuyu
50745	Heilongjiang Qiqihaer
50749	Heilongjiang Lindian
50750	Heilongjiang Yian
50755	Heilongjiang Baiquan
50756	Heilongjiang Hailun
50758	Heilongjiang Minshui
50767	Heilongjiang Suileng
50772	Heilongjiang Wuying
50774	Heilongjiang Yichun
50775	Heilongjiang Hegang
50776	Heilongjiang Luobei
50778	Heilongjiang Tongjiang
50779	Heilongjiang Fuyuan
50787	Heilongjiang Suibin
50788	Heilongjiang Fujin
50842	Heilongjiang Dumeng

50844	Heilongjiang Tailai
50850	Heilongjiang Daqing
50851	Heilongjiang Qinggang
50852	Heilongjiang Wangkui
50853	Heilongjiang Beilin
50854	Heilongjiang Anda
50858	Heilongjiang Zhaodong
50859	Heilongjiang Lansi
50861	Heilongjiang Qingan
50862	Heilongjiang Tieli
50867	Heilongjiang Bayan
50871	Heilongjiang Tangyuan
50873	Heilongjiang Jiamusi
50877	Heilongjiang Yilang
50878	Heilongjiang Huachuan
50879	Heilongjiang Huanan
50884	Heilongjiang Shuangyashan
50888	Heilongjiang Baoqing
50892	Heilongjiang Raohe
50950	Heilongjiang Zhaozhou
50953	Heilongjiang Haerbin
50956	Heilongjiang Hulan
50958	Heilongjiang Acheng
50960	Heilongjiang Binxian
50962	Heilongjiang Mulan
50963	Heilongjiang Tonghe
50964	Heilongjiang Zhengfang
50965	Heilongjiang Yanshou
50968	Heilongjiang Shangzhi
50971	Heilongjiang Qitaihe
50973	Heilongjiang Boli
50978	Heilongjiang Jixi
50979	Heilongjiang Linkou
50983	Heilongjiang Hulin
50985	Heilongjiang Mishan
50987	Heilongjiang Jidong
54080	Heilongjiang Wuchang
54092	Heilongjiang Hailin
54093	Heilongjiang Muling
54094	Heilongjiang Mudanjiang
54096	Heilongjiang Suifenhe
54098	Heilongjiang Ningan
57249	Hubei Zhuxi

57251	Hubei Yunxi
57253	Hubei Yunxian
57256	Hubei Shiyan
57257	Hubei Zhushan
57259	Hubei Fangxian
57260	Hubei Danjiangkou
57268	Hubei Gucheng
57278	Hubei Xiangyang
57279	Hubei Zaoyang
57355	Hubei Padang
57359	Hubei Xingshan
57361	Hubei Baokang
57362	Hubei Shennongjia
57363	Hubei Nanzhang
57370	Hubei Yicheng
57377	Hubei Jingmen
57378	Hubei Zhongxiang
57381	Hubei Suizhou
57386	Hubei Xiaochang
57387	Hubei Jingshan
57388	Hubei Anlu
57398	Hubei Hongan
57399	Hubei Macheng
57439	Hubei Lichuan
57445	Hubei Jianshi
57447	Hubei Enshi
57453	Hubei Yiling
57458	Hubei Wufeng
57469	Hubei Songzi
57475	Hubei Qianjiang
57477	Hubei Gongan
57481	Hubei Yingcheng
57482	Hubei Xiaogan
57483	Hubei Tianmen
57484	Hubei Shayang
57485	Hubei Xiantao
57486	Hubei Hanchuan
57489	Hubei Caidian
57492	Hubei Xinzhou
57494	Hubei Wuhan
57495	Hubei Tuanfeng
57496	Hubei Ezhou
57540	Hubei Xianfeng

57541	Hubei Xuanen
57543	Hubei Hefeng
57571	Hubei Shishou
57573	Hubei Jianli
57581	Hubei Honghu
57582	Hubei Chibi
57583	Hubei Jiayu
57586	Hubei Chongyang
57589	Hubei Tongcheng
57590	Hubei Xianning
58401	Hubei Luotian
58402	Hubei Yingshan
58408	Hubei Qichun
58409	Hubei Huangmei
58500	Hubei Yangxin
58501	Hubei Wuxue
57544	Hunan Longshan
57554	Hunan Sangzhi
57558	Hunan Zhangjiajie
57565	Hunan Lixian
57574	Hunan Nanxian
57575	Hunan Huarong
57584	Hunan Yueyang
57642	Hunan Baojing
57643	Hunan Yongshun
57646	Hunan Guzhang
57649	Hunan Jishou
57655	Hunan Yuanling
57657	Hunan Luxi
57661	Hunan Taoyuan
57662	Hunan Changde
57663	Hunan Hanshou
57666	Hunan Taojiang
57669	Hunan Anhua
57671	Hunan Yuanjiang
57673	Hunan Xiangyin
57678	Hunan Ningxiang
57680	Hunan Milo
57682	Hunan Pingjiang
57687	Hunan Changsha
57688	Hunan Liuyang
57740	Hunan Fenghuang
57744	Hunan Xinhuang

57745	Hunan Zhijiang
57752	Hunan Xupu
57754	Hunan Hongjiang
57760	Hunan Lengshuijiang
57761	Hunan Xinhua
57763	Hunan Loudi
57771	Hunan Shaoshan
57772	Hunan Xiangxiang
57774	Hunan Shuangfeng
57776	Hunan Nanyue
57777	Hunan Hengshan
57779	Hunan Youxian
57780	Hunan Zhuzhou
57781	Hunan Liling
57845	Hunan Tongtao
57865	Hunan Lengshuitan
57866	Hunan Yongzhou
57867	Hunan Dongan
57868	Hunan Qiyang
57870	Hunan Qidong
57871	Hunan Hengyangxian
57872	Hunan Hengyang
57874	Hunan Changning
57875	Hunan Hengnan
57881	Hunan Anren
57882	Hunan Chaling
57887	Hunan Yongxing
57889	Hunan Guangxi
57962	Hunan Shuangpai
57965	Hunan Daoxian
57966	Hunan Ningyuan
57969	Hunan Jiangyong
57971	Hunan Xintian
57972	Hunan Chenzhou
57973	Hunan Guiyang
57974	Hunan Jiahe
57975	Hunan Lanshan
57976	Hunan Yizhang
57978	Hunan Linwu
57981	Hunan Zixing
57985	Hunan Rucheng
59063	Hunan Jianghua
50936	Jilin Baicheng

50939	Jilin Taonan
50940	Jilin Zhenlai
50945	Jilin Daan
50946	Jilin Songyuan
50948	Jilin Qianan
50949	Jilin Qianguo
54041	Jilin Tongyu
54049	Jilin Changling
54063	Jilin Fuyu
54064	Jilin Nongan
54065	Jilin Dehui
54069	Jilin Jiutai
54072	Jilin Yushu
54076	Jilin Shulan
54142	Jilin Shuangliao
54154	Jilin Lishu
54155	Jilin Gujiazi
54161	Jilin Changchun
54164	Jilin Yitong
54165	Jilin Shuangyang
54169	Jilin Yantongshan
54171	Jilin Yongji
54172	Jilin Jilin Suburb
54181	Jilin Jiaohe
54186	Jilin Dunhua
54187	Jilin Antu
54192	Jilin Luozigou
54195	Jilin Wangqing
54260	Jilin Liaoyuan
54261	Jilin Tongfeng
54263	Jilin Panshi
54267	Jilin Liuhe
54273	Jilin Huadian
54274	Jilin Huinan
54279	Jilin Jiangyuan
54284	Jilin Donggang
54285	Jilin Erdao
54286	Jilin Helong
54290	Jilin Longjing
54291	Jilin Huichun
54292	Jilin Yanji
54362	Jilin Tonghuaxian
54363	Jilin Tonghua

54371	Jilin Baishan
54374	Jilin Linjiang
54377	Jilin Jian
54386	Jilin Changbai
58012	Jiangsu Fengxian
58013	Jiangsu Peixian
58026	Jiangsu Pizhou
58027	Jiangsu Xuzhou
58036	Jiangsu Donghai
58038	Jiangsu Shuyang
58040	Jiangsu Ganyu
58041	Jiangsu Xiliandao
58044	Jiangsu Lianyungang
58045	Jiangsu Xiangshui
58047	Jiangsu Guanyun
58130	Jiangsu Suining
58131	Jiangsu Suyu
58132	Jiangsu Siyang
58135	Jiangsu Sihong
58138	Jiangsu Xuyi
58139	Jiangsu Hongze
58140	Jiangsu Lianshui
58141	Jiangsu Huaian
58143	Jiangsu Funing
58146	Jiangsu Jianhu
58147	Jiangsu Jinhu
58148	Jiangsu Baoying
58154	Jiangsu Yancheng
58158	Jiangsu Dafeng
58235	Jiangsu Liuhe
58237	Jiangsu Pukou
58238	Jiangsu Nanjing
58241	Jiangsu Gaoyou
58242	Jiangsu Yizheng
58243	Jiangsu Xinghua
58246	Jiangsu Taizhou
58247	Jiangsu Yangzhong
58249	Jiangsu Taixing
58250	Jiangsu Jiangyan
58251	Jiangsu Dongtai
58254	Jiangsu Haian
58255	Jiangsu Rugao
58257	Jiangsu Jingjiang

58259	Jiangsu Nantong
58264	Jiangsu Rudong
58265	Jiangsu Lvsi
58269	Jiangsu Qidong
58339	Jiangsu Gaochun
58340	Jiangsu Lishui
58341	Jiangsu Danyang
58342	Jiangsu Jintan
58344	Jiangsu Jurong
58346	Jiangsu Yixing
58349	Jiangsu Suzhou
58352	Jiangsu Changshu
58353	Jiangsu Zhangjiagang
58354	Jiangsu Wuxi
58356	Jiangsu Kunshan
58360	Jiangsu Haimen
58377	Jiangsu Taicang
57598	Jiangxi Xiushui
57694	Jiangxi Tonggu
57698	Jiangxi Wanzai
57699	Jiangxi Shanggao
57786	Jiangxi Pingxiang
57789	Jiangxi Lianhua
57792	Jiangxi Fenyi
57793	Jiangxi Yichun
57796	Jiangxi Xinyu
57798	Jiangxi Anfu
57799	Jiangxi Jianxian
57883	Jiangxi Xiaping
57891	Jiangxi Yongxin
57895	Jiangxi Wanan
57896	Jiangxi Suichuan
57899	Jiangxi Taihe
57990	Jiangxi Chongyi
57992	Jiangxi Nankang
57993	Jiangxi Ganxian
57995	Jiangxi Xinfeng
58502	Jiangxi Jiujiang
58503	Jiangxi Ruichang
58506	Jiangxi Lushan
58507	Jiangxi Wuning
58508	Jiangxi Dean
58510	Jiangxi Hukou

58512	Jiangxi Pengze
58517	Jiangxi Duchang
58519	Jiangxi Poyang
58527	Jiangxi Jingdezhen
58529	Jiangxi Wuyuan
58600	Jiangxi Jingan
58601	Jiangxi Fengxin
58602	Jiangxi Anyi
58605	Jiangxi Gaoan
58606	Jiangxi Nanchang
58612	Jiangxi Yugan
58614	Jiangxi Jinxian
58615	Jiangxi Wannian
58618	Jiangxi Dongxiang
58619	Jiangxi Linchuan
58622	Jiangxi Dexing
58623	Jiangxi Shangraoxian
58626	Jiangxi Guixi
58629	Jiangxi Qianshan
58634	Jiangxi Yushan
58693	Jiangxi Xinjian
58704	Jiangxi Xiajiang
58705	Jiangxi Yongfeng
58706	Jiangxi Lean
58710	Jiangxi Chongren
58712	Jiangxi Jinxi
58715	Jiangxi Nancheng
58718	Jiangxi Nanfeng
58719	Jiangxi Lichuan
58804	Jiangxi Xingguo
58806	Jiangxi Ningdu
58813	Jiangxi Guangchang
58814	Jiangxi Shicheng
58905	Jiangxi Yudu
58906	Jiangxi Huichang
58907	Jiangxi Anyuan
59091	Jiangxi Quannan
59092	Jiangxi Longnan
59093	Jiangxi Dingnan
59102	Jiangxi Xunwu
57783	Jiangxi Shangli
54236	Liaoning Zhangwu
54243	Liaoning Changtu

54244	Liaoning Kangping
54248	Liaoning Shenbei
54249	Liaoning Tieling
54252	Liaoning Xifeng
54259	Liaoning Qingyuan
54321	Liaoning Jianpingzhen
54324	Liaoning Chaoyang
54325	Liaoning Yangshan
54326	Liaoning Jianpingxian
54327	Liaoning Lingyuan
54332	Liaoning Liaozhong
54333	Liaoning Xinmin
54336	Liaoning Taian
54338	Liaoning Panshan
54339	Liaoning Anshan
54340	Liaoning Sujiatun
54342	Liaoning Shenyang
54345	Liaoning Liaoyangxian
54346	Liaoning Benxi
54351	Liaoning Fushun
54353	Liaoning Xinbin
54452	Liaoning Jianchang
54453	Liaoning Lianshan
54454	Liaoning Suizhong
54455	Liaoning Xingcheng
54471	Liaoning Yingkou
54474	Liaoning Gaizhou
54475	Liaoning Dashiqiao
54483	Liaoning Caohekou
54486	Liaoning Xiuyan
54493	Liaoning Kuandian
54494	Liaoning Fengcheng
54497	Liaoning Dandong
54563	Liaoning Wafangdian
54568	Liaoning Jinzhou
54569	Liaoning Pulandian
54575	Liaoning Pikou
54579	Liaoning Changhai
54584	Liaoning Zhuanghe
54565	Liaoning Changxingdao
50425	Inner Mongolia Eerguna
50434	Inner Mongolia Tulihe
50445	Inner Mongolia Elunchunqi

50514	Inner Mongolia Manzhouli
50525	Inner Mongolia Evenkeqi
50526	Inner Mongolia Yakeshi
50548	Inner Mongolia Xiaoergou
50603	Inner Mongolia Xinbaerhuyouqi
50618	Inner Mongolia Xinbaerhuzuoqi
50639	Inner Mongolia Zhalantun
50645	Inner Mongolia Moulidawawoer
50647	Inner Mongolia Arunqi
50727	Inner Mongolia Aershan
50834	Inner Mongolia Suolun
50913	Inner Mongolia Wulagai
50915	Inner Mongolia Wuzhumuqindong
50924	Inner Mongolia Houlinguole
50928	Inner Mongolia Bayaertuhushuo
50934	Inner Mongolia Tuquan
53068	Inner Mongolia Erlianhaote
53083	Inner Mongolia Narenbaolige
53149	Inner Mongolia Mandula
53192	Inner Mongolia Abagaqi
53231	Inner Mongolia Hailisu
53289	Inner Mongolia Xianghuangqi
53336	Inner Mongolia Wulatezhongqi
53337	Inner Mongolia Wuyuan
53348	Inner Mongolia Dashetai
53352	Inner Mongolia Damaoqi
53357	Inner Mongolia Guyangxian
53362	Inner Mongolia Siziwang
53367	Inner Mongolia Xilamuren
53368	Inner Mongolia Wuchuanxian
53378	Inner Mongolia Chayouzhongqi
53384	Inner Mongolia Chayouhouqi
53385	Inner Mongolia Shangdu
53391	Inner Mongolia Huade
53419	Inner Mongolia Dengkou
53420	Inner Mongolia Hangjinhouqi
53433	Inner Mongolia Wulateqianqi
53446	Inner Mongolia Baotou
53455	Inner Mongolia Tuyouqi
53457	Inner Mongolia Dalateqi
53463	Inner Mongolia Huhehaote
53464	Inner Mongolia Tumutezuoqi
53466	Inner Mongolia Huhehaote suburb

53467	Inner Mongolia Tuoketuoxian
53469	Inner Mongolia Helingeerxian
53472	Inner Mongolia Zhuozi
53475	Inner Mongolia Liangcheng
53480	Inner Mongolia Jining
53481	Inner Mongolia Chayouqianqi
53483	Inner Mongolia Xinghe
53512	Inner Mongolia Wuhai
53513	Inner Mongolia Linhe
53522	Inner Mongolia Yikewusu
53529	Inner Mongolia Etukeqi
53533	Inner Mongolia Hangjinqi
53543	Inner Mongolia Dongsheng
53545	Inner Mongolia Ejinhuoluoqi
53553	Inner Mongolia Zhungeerqi
53562	Inner Mongolia Qingshuihexian
53644	Inner Mongolia Wushenqi
53730	Inner Mongolia Eduokeqianqi
53732	Inner Mongolia Henan
54012	Inner Mongolia Xiwuzhumuqin
54024	Inner Mongolia Fuhe
54026	Inner Mongolia Zhalute
54027	Inner Mongolia Balinzuoqi
54031	Inner Mongolia Gaoliban
54039	Inner Mongolia Shebotu
54047	Inner Mongolia Kezuozhongqi
54102	Inner Mongolia Xilinhaote
54113	Inner Mongolia Balinyouqi
54115	Inner Mongolia Linxixian
54117	Inner Mongolia Keshiketengqi
54122	Inner Mongolia Alukeerqinqi
54132	Inner Mongolia Qinglongshan
54134	Inner Mongolia Kailu
54135	Inner Mongolia Tongliao
54204	Inner Mongolia Zhengxiangbaiqi
54205	Inner Mongolia Zhenglanqi
54208	Inner Mongolia Duolunxian
54213	Inner Mongolia Wengniuteqi
54214	Inner Mongolia Gangzi
54218	Inner Mongolia Chifeng
54223	Inner Mongolia Neiman
54225	Inner Mongolia Aohanqi
54226	Inner Mongolia Baoguotu

54231	Inner Mongolia Kezuohouqi
54234	Inner Mongolia Kulun
54305	Inner Mongolia Taibushiqi
54313	Inner Mongolia Kalaqinqi
54316	Inner Mongolia Balihan
54320	Inner Mongolia Ningchengxian
53517	Ningxia Shitanjing
53519	Ningxia Huinong
53610	Ningxia Helan
53611	Ningxia Pingluo
53612	Ningxia Wuzhong
53615	Ningxia Taole
53618	Ningxia Yongning
53704	Ningxia Zhongwei
53705	Ningxia Zhongning
53707	Ningxia Xingren
53723	Ningxia Yanchi
53727	Ningxia Maihuangshan
53806	Ningxia Haiyuan
53810	Ningxia Tongxin
53817	Ningxia Guyuan
53881	Ningxia Weizhou
53903	Ningxia Xiji
53910	Ningxia Liupanshan
52602	Qinghai Lenghu
52825	Qinghai Nuomuhong
52836	Qinghai Doulan
52854	Qinghai Qinghaihu 151
52856	Qinghai Gonghe
52863	Qinghai Huzhu
52866	Qinghai Xining
52868	Qinghai Guide
52875	Qinghai Pingan
52876	Qinghai Minhe
52877	Qinghai Hualong
52908	Qinghai Wudaoliang
52941	Qinghai Shazhuyu
52943	Qinghai Xinghai
52955	Qinghai Guinan
52957	Qinghai Tongde
52963	Qinghai Jianzha
52968	Qinghai Zeku
52972	Qinghai Xunhua

52974	Qinghai Tongren
56004	Qinghai Tuotuohe
56016	Qinghai Zhiduo
56018	Qinghai Zaduo
56021	Qinghai Qumacai
56029	Qinghai Yushu
56033	Qinghai Maduo
56034	Qinghai Qingshuihe
56043	Qinghai Maqin
56045	Qinghai Gander
56046	Qinghai Dari
56065	Qinghai Henan
56067	Qinghai Jiuzhi
56125	Qinghai Nangqian
56151	Qinghai Banma
54709	Shandong Wucheng
54712	Shandong Linyi
54716	Shandong Ningjin
54723	Shandong Yangxin
54724	Shandong Shanghe
54726	Shandong Leling
54727	Shandong Zhangqiu
54729	Shandong Gaoqing
54734	Shandong Binzhou
54744	Shandong Kenli
54749	Shandong Laizhou
54751	Shandong Longdao
54752	Shandong Penglai
54753	Shandong Longkou
54755	Shandong Zhaoyuan
54759	Shandong Qixia
54764	Shandong Fushan
54765	Shandong Yantai
54776	Shandong Chengshantou
54777	Shandong Wendeng
54802	Shandong Linqing
54806	Shandong Liaocheng
54808	Shandong Xinxian
54812	Shandong Qihe
54814	Shandong Chiping
54815	Shandong Donge
54819	Shandong Feicheng
54821	Shandong Jiyang

54822	Shandong Zouping
54823	Shandong Jinan
54827	Shandong Taian
54828	Shandong Laiwu
54830	Shandong Zibo
54831	Shandong Qingzhou
54832	Shandong Shouguang
54833	Shandong Huantai
54836	Shandong Yiyuan
54841	Shandong Changyi
54842	Shandong Pingdu
54843	Shandong Weifang
54844	Shandong Anqiu
54846	Shandong Gaomi
54848	Shandong Zhucheng
54849	Shandong Jiaozhou
54852	Shandong Laiyang
54855	Shandong Jimo
54861	Shandong Rushan
54904	Shandong Juancheng
54905	Shandong Yuncheng
54906	Shandong Heze
54907	Shandong Yutai
54909	Shandong Dingtao
54910	Shandong Liangshan
54911	Shandong Dongping
54912	Shandong Wenshang
54914	Shandong Juye
54916	Shandong Yanzhou
54919	Shandong Zoucheng
54920	Shandong Sishui
54922	Shandong Xintai
54923	Shandong Mengyin
54925	Shandong Pingyi
54927	Shandong Tengzhou
54929	Shandong Feixian
54932	Shandong Yishui
54938	Shandong Linyi
54939	Shandong Junan
54940	Shandong Wulian
54943	Shandong Huangdao
54945	Shandong Rizhao
58002	Shandong Caoxian

58003	Shandong Chengwu
58021	Shandong Xuechen
58022	Shandong Yicheng
58024	Shandong Zaozhuang
58030	Shandong Cangshan
58032	Shandong Linshu
53478	Shanxi Youyu
53486	Shanxi Yanggao
53487	Shanxi Datong
53564	Shanxi Hequ
53565	Shanxi Pianguan
53574	Shanxi Pinglu
53575	Shanxi Shenchi
53576	Shanxi Shanyin
53577	Shanxi Ningwu
53578	Shanxi Shuozhou
53579	Shanxi Daixian
53582	Shanxi Hunyuan
53584	Shanxi Yingxian
53585	Shanxi Fanshi
53588	Shanxi Wutaishan
53590	Shanxi Guangling
53659	Shanxi Linxian
53662	Shanxi Kelan
53663	Shanxi Wuzhai
53664	Shanxi Xingxian
53665	Shanxi Lanxian
53666	Shanxi Jingle
53673	Shanxi Yuanping
53674	Shanxi Xinfu
53676	Shanxi Dingxiang
53677	Shanxi Jiancaoping
53679	Shanxi Xiaodian
53681	Shanxi Wutaixian
53685	Shanxi Yuxian
53687	Shanxi Pingding
53753	Shanxi Liulin
53759	Shanxi Shilou
53760	Shanxi Fangshan
53763	Shanxi Gujiao
53764	Shanxi Lishi
53767	Shanxi Zhongyang
53768	Shanxi Xiaoyi

53774	Shanxi Qingxu
53775	Shanxi Taigu
53778	Shanxi Pingyao
53780	Shanxi Shouyang
53782	Shanxi Yangquan
53786	Shanxi Zuoquan
53787	Shanxi Yushe
53788	Shanxi Heshun
53852	Shanxi Yonghe
53853	Shanxi Xixian
53859	Shanxi Jixian
53860	Shanxi Jiaokou
53861	Shanxi Xiangfen
53862	Shanxi Lingshi
53863	Shanxi Jiexiu
53864	Shanxi Puxian
53865	Shanxi Fenxi
53866	Shanxi Hongtong
53871	Shanxi Wuxiang
53872	Shanxi Qinxian
53873	Shanxi Changzi
53874	Shanxi Guxian
53875	Shanxi Qinyuan
53877	Shanxi Anze
53878	Shanxi Licheng
53880	Shanxi Lucheng
53953	Shanxi Xiangning
53954	Shanxi Jishan
53956	Shanxi Wanrong
53957	Shanxi Hejin
53959	Shanxi Yanhu
53964	Shanxi Xinjiang
53965	Shanxi Jiangxian
53966	Shanxi Fushan
53968	Shanxi Yuanqu
53970	Shanxi Qingshui
53973	Shanxi Gaoping
53981	Shanxi Lingchuan
57052	Shanxi Yongji
57053	Shanxi Ruicheng
53567	Shaanxi Fugu
53646	Shaanxi Yulin
53651	Shaanxi Shenmu

53658	Shaanxi Jiaxian
53725	Shaanxi Dingbian
53735	Shaanxi Jingbian
53738	Shaanxi Wuqi
53740	Shaanxi Hengshan
53748	Shaanxi Zichang
53754	Shaanxi Suide
53756	Shaanxi Wubao
53757	Shaanxi Qingjian
53832	Shaanxi Zhidan
53841	Shaanxi Ansai
53848	Shaanxi Ganquan
53850	Shaanxi Yanchuan
53854	Shaanxi Yanchang
53857	Shaanxi Yichuan
53931	Shaanxi Fuxian
53938	Shaanxi Xunyi
53941	Shaanxi Baishui
53944	Shaanxi Huangling
53946	Shaanxi Huanglong
53947	Shaanxi Tongchuan
53948	Shaanxi Pucheng
53949	Shaanxi Chengcheng
53950	Shaanxi Heyang
57003	Shaanxi Longxian
57016	Shaanxi Baoji
57021	Shaanxi Qianyang
57022	Shaanxi Linyou
57026	Shaanxi Fufeng
57027	Shaanxi Meixian
57029	Shaanxi Liquan
57030	Shaanxi Yongshou
57034	Shaanxi Wugong
57037	Shaanxi Yaoxian
57038	Shaanxi Xingping
57041	Shaanxi Sanyuan
57042	Shaanxi Fuping
57043	Shaanxi Dali
57045	Shaanxi Weinan
57046	Shaanxi Huashan
57057	Shaanxi Luonan
57106	Shaanxi Lueyang
57113	Shaanxi Fengxian

57119	Shaanxi Mianxian
57124	Shaanxi Liuba
57128	Shaanxi Chenggu
57134	Shaanxi Foping
57137	Shaanxi Ningshan
57140	Shaanxi Zhashui
57143	Shaanxi Shangxian
57153	Shaanxi Danfeng
57154	Shaanxi Shangnan
57155	Shaanxi Shanyang
57211	Shaanxi Ningqiang
57213	Shaanxi Nanzheng
57231	Shaanxi Ziyang
57233	Shaanxi Hanyin
57238	Shaanxi Zhenba
57242	Shaanxi Xunyang
57245	Shaanxi Ankang
57247	Shaanxi Langao
57248	Shaanxi Pingli
57254	Shaanxi Baihe
57343	Shaanxi Zhenping
58361	Shanghai Minhang
58362	Shanghai Baoshan
58460	Shanghai Jinshan
58463	Shanghai Fengxian
56038	Sichuan Shiqu
56079	Sichuan Ruoergai
56097	Sichuan Jiuzhaigou
56144	Sichuan Dege
56146	Sichuan Ganzi
56147	Sichuan Baiyu
56152	Sichuan Seda
56158	Sichuan Luhuo
56164	Sichuan Rangtang
56167	Sichuan Daofu
56168	Sichuan Jinchuan
56171	Sichuan Aba
56172	Sichuan Maerkang
56173	Sichuan Hongyuan
56178	Sichuan Xiaojin
56180	Sichuan Maoxian
56181	Sichuan Chongzhou
56182	Sichuan Songpan

56183	Sichuan Wenchuan
56184	Sichuan Lixian
56185	Sichuan Heishui
56186	Sichuan Mianzhu
56187	Sichuan Wenjiang
56188	Sichuan Dujiangyan
56189	Sichuan Pengzhou
56198	Sichuan Deyang
56247	Sichuan Batang
56251	Sichuan Xinlong
56257	Sichuan Litang
56263	Sichuan Danba
56267	Sichuan Yajiang
56273	Sichuan Baoxing
56279	Sichuan Lushan
56280	Sichuan Mingshan
56281	Sichuan Pujiang
56286	Sichuan Longquanyi
56289	Sichuan Pengshan
56296	Sichuan Jintang
56297	Sichuan Renshou
56298	Sichuan Ziyang
56357	Sichuan Daocheng
56371	Sichuan Luding
56373	Sichuan Yingjing
56374	Sichuan Kangding
56376	Sichuan Hanyuan
56378	Sichuan Shimian
56380	Sichuan Hongya
56382	Sichuan Jiajiang
56383	Sichuan Qingshen
56385	Sichuan Emeishan
56387	Sichuan Ebian
56389	Sichuan Qianwei
56390	Sichuan Jingyan
56393	Sichuan Zizhong
56395	Sichuan Weiyuan
56396	Sichuan Zigong
56399	Sichuan Fushun
56441	Sichuan Derong
56443	Sichuan Xiangcheng
56459	Sichuan Muli
56462	Sichuan Jiulong

56473	Sichuan Ganluo
56474	Sichuan Mianning
56475	Sichuan Yuexi
56478	Sichuan Xide
56479	Sichuan Chaojue
56480	Sichuan Mabian
56485	Sichuan Leibo
56487	Sichuan Meigu
56490	Sichuan Muchuan
56491	Sichuan Yibinxian
56493	Sichuan Nanxi
56494	Sichuan Pingshan
56496	Sichuan Xingwen
56499	Sichuan Gongxian
56565	Sichuan Yanyuan
56569	Sichuan Dechang
56571	Sichuan Xichang
56575	Sichuan Puge
56580	Sichuan Butuo
56584	Sichuan Jinyang
56593	Sichuan Changning
56665	Sichuan Yanbian
56666	Sichuan Panzhihua
56670	Sichuan Miyi
56671	Sichuan Huili
56675	Sichuan Huidong
57204	Sichuan Qingchuan
57206	Sichuan Guangyuan
57208	Sichuan Jiange
57216	Sichuan Nanjiang
57217	Sichuan Wangcang
57237	Sichuan Wanyuan
57306	Sichuan Langzhong
57309	Sichuan Xichong
57313	Sichuan Bazhong
57314	Sichuan Nanbu
57315	Sichuan Yilong
57318	Sichuan Yingshan
57320	Sichuan Tongjiang
57324	Sichuan Pingchang
57328	Sichuan Dachuan
57329	Sichuan Kaijiang
57401	Sichuan Shehong

57405	Sichuan Suining
57411	Sichuan Gaoping
57413	Sichuan Quxian
57415	Sichuan Guangan
57416	Sichuan Linshui
57417	Sichuan Wusheng
57420	Sichuan Dazhu
57503	Sichuan Dongxing
57507	Sichuan Longchang
57600	Sichuan Jiangan
57603	Sichuan Hejiang
57608	Sichuan Xuyong
54523	Tianjin Wuqing
54525	Tianjin Baodi
54619	Tianjin Jinghai
54622	Tianjin Jinnan
54623	Tianjin Tanggu
54645	Tianjin Dagang
55228	Tibet Shiquanhe
55248	Tibet Gaize
55299	Tibet Naqu
55437	Tibet Pulan
55493	Tibet Dangxiong
55569	Tibet Lazi
55572	Tibet Nanmulin
55593	Tibet Muozhugongka
55598	Tibet Zedang
55655	Tibet Nielamu
55664	Tibet Dingri
55680	Tibet Jiangzi
56116	Tibet Dingqing
56128	Tibet Leiwuqi
56137	Tibet Changdu
56223	Tibet Luolong
56227	Tibet Bomi
56228	Tibet Basu
56312	Tibet Linzhi
56317	Tibet Miling
56331	Tibet Zuogong
56434	Tibet Chayu
51053	Xinjiang Habahe
51058	Xinjiang Akedala
51060	Xinjiang Buerjin

51068	Xinjiang Fuhai
51076	Xinjiang Aletai
51087	Xinjiang Fuyun
51133	Xinjiang Tacheng
51137	Xinjiang Yumin
51145	Xinjiang Emin
51156	Xinjiang Hebukesaier
51232	Xinjiang Alashankou
51238	Xinjiang Bole
51241	Xinjiang Touli
51243	Xinjiang Kelamayi
51334	Xinjiang Jinghe
51357	Xinjiang Shawan
51359	Xinjiang Manasi
51367	Xinjiang Hutubi
51368	Xinjiang Changji
51369	Xinjiang Miquan
51377	Xinjiang Fukang
51378	Xinjiang Jimusaer
51468	Xinjiang Tianshandaxigou
51469	Xinjiang Urumqi Pastoral Test Station
51470	Xinjiang Tianchi
51477	Xinjiang Dabancheng
51482	Xinjiang Mulei
51526	Xinjiang Kumishi
51542	Xinjiang Bayinbuluke
51567	Xinjiang Yanqi
51571	Xinjiang Tuokexun
51572	Xinjiang Tulufandongkan
51573	Xinjiang Tulufan
51627	Xinjiang Wushi
51628	Xinjiang Akesu
51636	Xinjiang Xinhe
51639	Xinjiang Shaya
51656	Xinjiang Kuerle
51704	Xinjiang Atushi
51705	Xinjiang Wuqia
51707	Xinjiang Jiashi
51709	Xinjiang Kashi
51717	Xinjiang Yuepuhu
51720	Xinjiang Kepin
51722	Xinjiang Awati
51730	Xinjiang Alaer

51747	Xinjiang Tazhong
51765	Xinjiang Tieqianlike
51802	Xinjiang Yengjisha
51810	Xinjiang Maigaiti
51811	Xinjiang Shashe
51814	Xinjiang Yecheng
51815	Xinjiang Zepu
51818	Xinjiang Pishan
51826	Xinjiang Cele
51827	Xinjiang Moyu
51828	Xinjiang Hetan
51829	Xinjiang Luopu
51839	Xinjiang Minfeng
51855	Xinjiang Qiemuo
51931	Xinjiang Yutian
52101	Xinjiang Balikun
52112	Xinjiang Naomaohu
52118	Xinjiang Yiwu
52203	Xinjiang Hami
52313	Xinjiang Hongliuhe
56444	Yunnan Deqin
56483	Yunnan Suijiang
56497	Yunnan Yanjin
56533	Yunnan Gongshan
56543	Yunnan Xianggelila
56548	Yunnan Weixi
56567	Yunnan Ninglang
56582	Yunnan Daguan
56585	Yunnan Ludian
56594	Yunnan Yiliang
56595	Yunnan Zhenxiong
56596	Yunnan Weixin
56641	Yunnan Fugong
56643	Yunnan Liuku
56645	Yunnan Lanping
56646	Yunnan Jianchuan
56649	Yunnan Eryuan
56651	Yunnan Lijiang
56652	Yunnan Yongsheng
56654	Yunnan Heqing
56664	Yunnan Huaping
56669	Yunnan Yongren
56673	Yunnan Qiaojia

56688	Yunnan Dongchuan
56697	Yunnan Xuanwei
56739	Yunnan Tengchong
56742	Yunnan Yunlong
56745	Yunnan Yangbi
56746	Yunnan Yongping
56748	Yunnan Baoshan
56751	Yunnan Dali
56752	Yunnan Binchuan
56755	Yunnan Midu
56757	Yunnan Weishan
56764	Yunnan Yaoan
56766	Yunnan Mouding
56767	Yunnan Nanhua
56772	Yunnan Fumin
56774	Yunnan Wuding
56777	Yunnan Lufeng
56778	Yunnan Kunming
56782	Yunnan Malong
56783	Yunnan Qujing
56785	Yunnan Songming
56790	Yunnan Fuyuan
56835	Yunnan Longchuan
56836	Yunnan Yingjiang
56839	Yunnan Zhenkang
56840	Yunnan Lianghe
56841	Yunnan Longling
56842	Yunnan Shidian
56843	Yunnan Changning
56846	Yunnan Fengqing
56849	Yunnan Yongde
56854	Yunnan Yunxian
56856	Yunnan Jingdong
56862	Yunnan Shuangbai
56863	Yunnan Anning
56867	Yunnan Zhenyuan
56869	Yunnan Xinping
56870	Yunnan Yimen
56871	Yunnan Jinning
56873	Yunnan Chengjiang
56875	Yunnan Yuxi
56879	Yunnan Huaning
56880	Yunnan Yiliang

56881	Yunnan Shilin
56883	Yunnan Shizong
56885	Yunnan Mile
56886	Yunnan Luxi
56889	Yunnan Qiubei
56891	Yunnan Luoping
56898	Yunnan Eshan
56944	Yunnan Cangyuan
56946	Yunnan Gengma
56948	Yunnan Ximeng
56949	Yunnan Menglian
56950	Yunnan Shuangjiang
56951	Yunnan Lincang
56952	Yunnan Jinggu
56954	Yunnan Lancang
56958	Yunnan Menghai
56962	Yunnan Mojiang
56964	Yunnan Simao
56966	Yunnan Yuanjiang
56969	Yunnan Mengla
56970	Yunnan Shiping
56976	Yunnan Yuanyang
56977	Yunnan Jiangcheng
56978	Yunnan Luchun
56982	Yunnan Kaiyuan
56984	Yunnan Gejiu
56987	Yunnan Jinping
56991	Yunnan Yanshan
56992	Yunnan Xichou
56994	Yunnan Wenshan
59007	Yunnan Guangnan
59205	Yunnan Funing
58443	Zhejiang Changxing
58446	Zhejiang Anji
58448	Zhejiang Linan
58449	Zhejiang Fuyang
58450	Zhejiang Huzhou
58451	Zhejiang Jiashan
58452	Zhejiang Jiaxing
58453	Zhejiang Shaoxing
58454	Zhejiang Deqing
58457	Zhejiang Hangzhou
58467	Zhejiang Cixi

58472	Zhejiang Shengsi
58484	Zhejiang Daishan
58537	Zhejiang Kaihua
58542	Zhejiang Tonglu
58544	Zhejiang Jiande
58546	Zhejiang Pujiang
58547	Zhejiang Longyou
58549	Zhejiang Jinhua
58550	Zhejiang Zhuji
58555	Zhejiang Xinchang
58556	Zhejiang Shengzhou
58558	Zhejiang Dongyang
58559	Zhejiang Tiantai
58560	Zhejiang Panan
58561	Zhejiang Zhenhai
58562	Zhejiang Yinzhou
58565	Zhejiang Fenghua
58566	Zhejiang Xiangshan
58567	Zhejiang Ninghai
58568	Zhejiang Sanmen
58569	Zhejiang Shipu
58570	Zhejiang Putuo
58631	Zhejiang Changshan
58632	Zhejiang Jiangshan
58642	Zhejiang Wuyi
58643	Zhejiang Yongkang
58644	Zhejiang Suichang
58646	Zhejiang Lishui
58647	Zhejiang Longquan
58652	Zhejiang Xianju
58654	Zhejiang Jinyun
58656	Zhejiang Leqing
58657	Zhejiang Qingtian
58658	Zhejiang Yongjia
58660	Zhejiang Linhai
58664	Zhejiang Wenling
58665	Zhejiang Hongjia
58666	Zhejiang Dachen
58742	Zhejiang Yunhe
58746	Zhejiang Taishun
58750	Zhejiang Wencheng
58751	Zhejiang Pingyang
58648	Zhejiang Jingning

57333	Chongqing Chengkou
57338	Chongqing Kaixian
57339	Chongqing Yunyang
57345	Chongqing Wuxi
57349	Chongqing Wushan
57409	Chongqing Tongnan
57425	Chongqing Dianjiang
57432	Chongqing Wanzhou
57437	Chongqing Zhongxian
57438	Chongqing Shizhu
57502	Chongqing Dazu
57505	Chongqing Rongchang
57506	Chongqing Yongchuan
57509	Chongqing Wansheng
57510	Chongqing Tongliang
57511	Chongqing Beibei
57513	Chongqing Yubei
57514	Chongqing Bishan
57517	Chongqing Jiangjin
57518	Chongqing Banan
57519	Chongqing Nanchuan
57520	Chongqing Changshou
57523	Chongqing Fengdu
57525	Chongqing Wulong
57536	Chongqing Qianjiang
57537	Chongqing Pengshui
57612	Chongqing Qijiang

Table S3. 1558 monitoring stations whose PM2.5 emissions are from electricity generation sector

58015	Anhui Dangshan
58016	Anhui Xiaoxian
58102	Anhui Bozhou
58108	Anhui Jieshou
58109	Anhui Taihe
58113	Anhui Suixi
58114	Anhui Woyang
58127	Anhui Huaiyuan
58202	Anhui Funan
58203	Anhui Fuyang
58240	Anhui Tianchang
58306	Anhui Jinzhai
58314	Anhui Huoshan
58336	Anhui Maanshan
58417	Anhui Susong
58441	Anhui Guangde
58442	Anhui Langxi
58520	Anhui Qimen
58523	Anhui Yixian
58530	Anhui Shexian
58531	Anhui Tunxi
54398	Beijing Shunyi
54399	Beijing Haidian
54406	Beijing Yanqing
54416	Beijing Miyun
54421	Beijing Miyunshangdianzi
54424	Beijing Pinggu
54431	Beijing Tongzhou
54499	Beijing Changping
54501	Beijing Zhaitang
54511	Beijing
54597	Beijing Xiayunling
58724	Fujian Guangze
58725	Fujian Shaowu
58730	Fujian Wuyishan
58731	Fujian Pucheng
58734	Fujian Jianyang
58735	Fujian Songxi
58736	Fujian Zhenghe
58744	Fujian Shouning

58747	Fujian Zhouning
58748	Fujian Fuan
58749	Fujian Zherong
58754	Fujian Fuding
58818	Fujian Ninghuai
58820	Fujian Taining
58822	Fujian Jianning
58823	Fujian Shunchang
58824	Fujian Mingxi
58828	Fujian Sanming
58836	Fujian Gutian
58837	Fujian Youxi
58839	Fujian Shuqing
58843	Fujian Xiapu
58844	Fujian Minhou
58846	Fujian Ningde
58848	Fujian Lianjiang
58911	Fujian Changting
58917	Fujian Wuping
58923	Fujian Datian
58926	Fujian Zhangping
58928	Fujian Huaan
58929	Fujian Anxi
58931	Fujian Jiuxianshan
58932	Fujian Yongtai
58938	Fujian Xiuyu
58941	Fujian Changle
58942	Fujian Fuqing
58944	Fujian Pingtan
58946	Fujian Putian
59113	Fujian Yongding
59122	Fujian Changtai
59124	Fujian Nanjing
59125	Fujian Pinghe
59129	Fujian Zhangpu
59130	Fujian Tongan
59131	Fujian Nanan
59133	Fujian Chongwu
59320	Fujian Zhaoan
59322	Fujian Yunxiao
52323	Gansu Mazongshan
52515	Gansu Subei
52533	Gansu Jiuquan

52546	Gansu Gaitai
52557	Gansu Linze
52643	Gansu Sunan
52656	Gansu Minle
52674	Gansu Yongchang
52679	Gansu Wuwei
52681	Gansu Minqin
52784	Gansu Gulang
52787	Gansu Wushaoling
52797	Gansu Jingtai
52881	Gansu Tianzhu
52884	Gansu Gaolan
52885	Gansu Yongdeng
52895	Gansu Jingyuan
52896	Gansu Baiyin
52978	Gansu Xiahe
52980	Gansu Yongjing
52981	Gansu Dongxiang
52982	Gansu Guanghe
52983	Gansu Yuzhong
52985	Gansu Hezheng
52986	Gansu Lintao
52988	Gansu Kangle
52993	Gansu Huining
52995	Gansu Anding
52996	Gansu Huajialing
52998	Gansu Weiyuan
53821	Gansu Huanxian
53829	Gansu Qingcheng
53906	Gansu Jingning
53915	Gansu Kongtong
53917	Gansu Zhuanglang
53923	Gansu Xifeng
53924	Gansu Lingtai
53925	Gansu Zhenyuan
53926	Gansu Jingchuan
53927	Gansu Huating
53930	Gansu Huachi
53934	Gansu Huishui
53935	Gansu Zhengning
56071	Gansu Luqu
56074	Gansu Maqu
56080	Gansu Hezuo

56081	Gansu Lintan
56082	Gansu Zhuoni
56084	Gansu Dibu
56091	Gansu Zhangxian
56092	Gansu Longxi
56093	Gansu Minxian
56094	Gansu Zhouqu
56095	Gansu Dangchang
56096	Gansu Wudu
56192	Gansu Wenxian
57001	Gansu Gangu
57002	Gansu Qinan
57004	Gansu Wushan
57006	Gansu Tianshui
57007	Gansu Lixian
57011	Gansu Qingshui
57012	Gansu Zhangjiachuan
57014	Gansu Maiji
57102	Gansu Chengxian
57105	Gansu Kangxian
57110	Gansu Huixian
57111	Gansu Liangdang
57988	Guangdong Lechang
57989	Guangdong Renhua
57996	Guangdong Nanxiong
59074	Guangdong Lianshan
59075	Guangdong Yangshan
59081	Guangdong Ruyuan
59082	Guangdong Shaoguan
59087	Guangdong Fogang
59088	Guangdong Yingde
59090	Guangdong Shixing
59094	Guangdong Wengyuan
59096	Guangdong Lianping
59099	Guangdong Heping
59106	Guangdong Pingyuan
59107	Guangdong Longchuan
59109	Guangdong Xingning
59114	Guangdong Jiaoling
59116	Guangdong Daipu
59117	Guangdong Meixian
59268	Guangdong Yunan
59270	Guangdong Huaiji

59271	Guangdong Guangning
59276	Guangdong Sihui
59279	Guangdong Sanshui
59280	Guangdong Qingyuan
59284	Guangdong Huadu
59285	Guangdong Conghua
59287	Guangdong Guangzhou
59289	Guangdong Dongguan
59290	Guangdong Longmen
59293	Guangdong Heyuan
59297	Guangdong Boluo
59303	Guangdong Wuhua
59304	Guangdong Zijin
59306	Guangdong Jiexi
59310	Guangdong Fengshun
59312	Guangdong Chaozhou
59313	Guangdong Raoping
59314	Guangdong Puning
59316	Guangdong Shantou
59456	Guangdong Xinyi
59462	Guangdong Luoding
59469	Guangdong Yangchun
59470	Guangdong Xinxing
59471	Guangdong Yunfu
59473	Guangdong Heshan
59475	Guangdong Kaiping
59476	Guangdong Xinhui
59477	Guangdong Enping
59481	Guangdong Panyu
59487	Guangdong Doumen
59488	Guangdong Zhuhai
59492	Guangdong Huidong
59493	Guangdong Shenzhen
59500	Guangdong Haifeng
59502	Guangdong Lufeng
59650	Guangdong Suixi
59653	Guangdong Gaozhou
59654	Guangdong Lianjiang
59655	Guangdong Huazhou
59656	Guangdong Wuchuan
59659	Guangdong Maoming
59750	Guangdong Leizhou
59754	Guangdong Xuwen

57859	Guangxi Ziyuan
57927	Guangxi Tiane
57941	Guangxi Sanjiang
57942	Guangxi Longsheng
57948	Guangxi Rongshui
57949	Guangxi Yongfu
57954	Guangxi Lingui
57955	Guangxi Xingan
57960	Guangxi Quanzhou
57964	Guangxi Guanyang
59004	Guangxi Xilin
59012	Guangxi Leye
59015	Guangxi Lingyun
59017	Guangxi Tianlin
59021	Guangxi Fengshan
59023	Guangxi Hechi
59027	Guangxi Bama
59034	Guangxi Yizhou
59037	Guangxi Duan
59038	Guangxi Xincheng
59041	Guangxi Liucheng
59045	Guangxi Luzhai
59053	Guangxi Pingle
59055	Guangxi Lipu
59057	Guangxi Jinxiu
59058	Guangxi Mengshan
59065	Guangxi Hezhou
59209	Guangxi Napo
59211	Guangxi Baise
59215	Guangxi Debao
59218	Guangxi Jingxi
59224	Guangxi Tiandong
59227	Guangxi Tiandeng
59229	Guangxi Longan
59230	Guangxi Mashan
59235	Guangxi Shanglin
59237	Guangxi Wuming
59238	Guangxi Binyang
59241	Guangxi Xiangzhou
59242	Guangxi Laibin
59246	Guangxi Wuxuan
59255	Guangxi Pingnan
59256	Guangxi Tengxian

59265	Guangxi Wuzhou
59266	Guangxi Cangwu
59419	Guangxi Pingxiang
59421	Guangxi Daxin
59429	Guangxi Shangsi
59431	Guangxi Nanning
59435	Guangxi Yongning
59441	Guangxi Hengxian
59446	Guangxi Lingshan
59448	Guangxi Pubei
59449	Guangxi Bobai
59451	Guangxi Beiliu
59452	Guangxi Rongxian
59454	Guangxi Cenxi
59457	Guangxi Luchuan
59632	Guangxi Qinzhou
59640	Guangxi Hepu
56691	Guizhou Weining
56792	Guizhou Puan
56793	Guizhou Panxian
57606	Guizhou Tongzi
57647	Guizhou Songtao
57717	Guizhou Zunyi
57742	Guizhou Wanshan
57803	Guizhou Xianxi
57805	Guizhou Zhijin
57822	Guizhou Huangping
57907	Guizhou Xingyi
57909	Guizhou Ceheng
57921	Guizhou Pingtang
57922	Guizhou Dushan
57926	Guizhou Libo
57932	Guizhou Rongjiang
57936	Guizhou Congjiang
59758	Hainan Haikou
59842	Hainan Lingao
59843	Hainan Chengmai
59845	Hainan Danzhou
59847	Hainan Changjiang
59848	Hainan Baisha
59851	Hainan Dingan
59854	Hainan Tunchang
59855	Hainan Qonghai

59856	Hainan Wenchang
59945	Hainan Baoting
59951	Hainan Wanning
53392	Hebei Kangbao
53397	Hebei Shangyi
53399	Hebei Zhangbei
53492	Hebei Yangyuan
53498	Hebei Xuanhua
53499	Hebei Wanquan
53593	Hebei Weixian
53596	Hebei Shunping
53599	Hebei Laiyuan
53680	Hebei Lingshou
53682	Hebei Quyang
53688	Hebei Xingtang
53689	Hebei Jinzhou
53690	Hebei Fuping
53692	Hebei Tangxian
53696	Hebei Dingzhou
53699	Hebei Wuji
53773	Hebei Linzhang
53781	Hebei Shahe
53785	Hebei Baixiang
53789	Hebei Luancheng
53794	Hebei Longyao
53795	Hebei Zanhuang
53796	Hebei Ningjin
53799	Hebei Julu
53886	Hebei Shexian
53890	Hebei Wuan
53892	Hebei Handan
53893	Hebei Quzhou
53894	Hebei Fengfeng
53896	Hebei Weixian
54301	Hebei Guyuan
54304	Hebei Chongli
54308	Hebei Fengning
54311	Hebei Weichang
54318	Hebei Longhua
54319	Hebei Pingquan
54401	Hebei Zhangjiakou
54404	Hebei Chicheng
54405	Hebei Huailai

54408	Hebei Zhulu
54420	Hebei Luanping
54423	Hebei Chengde
54425	Hebei Xinglong
54429	Hebei Zunhua
54430	Hebei Chengdexian
54432	Hebei Kuancheng
54434	Hebei Qianxi
54436	Hebei Qinglong
54437	Hebei Luannan
54438	Hebei Lulong
54439	Hebei Qianan
54449	Hebei Qinhuangdao
54502	Hebei Zhuozhou
54503	Hebei Rongcheng
54506	Hebei Gaobeidian
54510	Hebei Dachang
54512	Hebei Guan
54519	Hebei Yongqing
54520	Hebei Sanhe
54521	Hebei Xianghe
54534	Hebei Tangshan
54535	Hebei Caofeidian
54539	Hebei Leting
54540	Hebei Changli
54541	Hebei Funing
54601	Hebei Xushui
54603	Hebei Goyang
54605	Hebei Anxin
54606	Hebei Raoyang
54608	Hebei Shenzhou
54610	Hebei Renqiu
54612	Hebei Wenan
54613	Hebei Dacheng
54614	Hebei Hejian
54615	Hebei Qingxian
54616	Hebei Cangzhou
54618	Hebei Botou
54624	Hebei Huanghua
54628	Hebei Haixing
54631	Hebei Guangzong
54633	Hebei Xinhe
54640	Hebei Jize

54700	Hebei Wuqiang
54701	Hebei Xinji
54702	Hebei Hengshui
54703	Hebei Wuyi
54706	Hebei Qinghe
54707	Hebei Gucheng
54710	Hebei Fucheng
54711	Hebei Jingxian
54713	Hebei Dongguang
54800	Hebei-Weixian
54801	Hebei Linxi
54804	Hebei Daming
54809	Hebei Guantao
53972	Henan Qinyang
53974	Henan Qixian
53978	Henan Jiyuan
53982	Henan Jiaozuo
53983	Henan Fengqiu
53984	Henan Xiuwu
53985	Henan Huixian
53986	Henan Xinxiang
53991	Henan Tangyin
53992	Henan Junxian
53993	Henan Neihuang
53998	Henan Changheng
54817	Henan Taiqian
54900	Henan Puyang
54902	Henan Qingfeng
54903	Henan Fanxian
57051	Henan Sanmenxia
57056	Henan Lingbao
57063	Henan Mianchi
57066	Henan Luoning
57070	Henan Xinan
57071	Henan Mengjin
57072	Henan Mengzhou
57074	Henan Yichuan
57075	Henan Ruzhou
57078	Henan Ruyang
57079	Henan Wenxian
57080	Henan Gongyi
57081	Henan Xingyang
57082	Henan Dengfeng

57087	Henan Changge
57089	Henan Xuchang
57090	Henan Zhongmou
57091	Henan Kaifeng
57093	Henan Lankao
57095	Henan Yanling
57096	Henan Qixian
57099	Henan Taikang
57156	Henan Xixia
57169	Henan Neixiang
57173	Henan Lushan
57175	Henan Zhenping
57176	Henan Nanzhao
57177	Henan Wugang
57178	Henan Nanyang
57179	Henan Fangcheng
57180	Henan Jiaxian
57182	Henan Xiangcheng
57183	Henan Linying
57184	Henan Yexian
57185	Henan Wuyang
57186	Henan Luohe
57187	Henan Sheqi
57188	Henan Xiping
57189	Henan Suiping
57192	Henan Huaiyang
57193	Henan Xihua
57197	Henan Runan
57271	Henan Xinye
57273	Henan Tanghe
57281	Henan Biyang
57285	Henan Tongbai
57290	Henan Zhumadian
57292	Henan Pingyu
57293	Henan Xincai
57295	Henan Zhengyang
57296	Henan Xixian
57297	Henan Xinyang
57299	Henan Guangshan
57390	Henan Jigongshan
57396	Henan Xixian
58004	Henan Minquan
58005	Henan Shangqiu

58006	Henan Yucheng
58007	Henan Echeng
58017	Henan Xiayi
58100	Henan Dancheng
58207	Henan Huangchuan
58208	Henan Gushi
58301	Henan Shangcheng
50136	Heilongjiang Mohe
50137	Heilongjiang Beijicun
50246	Heilongjiang Tahe
50247	Heilongjiang Huzhong
50349	Heilongjiang Xinlin
50353	Heilongjiang Huma
50442	Heilongjiang Jiagedaqi
50468	Heilongjiang Aihui
50557	Heilongjiang Nenjiang
50564	Heilongjiang Sunwu
50566	Heilongjiang Xunke
50646	Heilongjiang Nehe
50655	Heilongjiang Wudalianchi
50656	Heilongjiang Beian
50658	Heilongjiang Keshan
50659	Heilongjiang Kedong
50673	Heilongjiang Jiayin
50674	Heilongjiang Wuyiling
50739	Heilongjiang Longjiang
50741	Heilongjiang Gannan
50742	Heilongjiang Fuyu
50745	Heilongjiang Qiqihaer
50749	Heilongjiang Lindian
50750	Heilongjiang Yian
50755	Heilongjiang Baiquan
50756	Heilongjiang Hailun
50758	Heilongjiang Minshui
50767	Heilongjiang Suileng
50772	Heilongjiang Wuying
50774	Heilongjiang Yichun
50775	Heilongjiang Hegang
50776	Heilongjiang Luobei
50778	Heilongjiang Tongjiang
50779	Heilongjiang Fuyuan
50787	Heilongjiang Suibin
50788	Heilongjiang Fujin

50842	Heilongjiang Dumeng
50844	Heilongjiang Tailai
50850	Heilongjiang Daqing
50851	Heilongjiang Qinggang
50852	Heilongjiang Wangkui
50853	Heilongjiang Beilin
50854	Heilongjiang Anda
50858	Heilongjiang Zhaodong
50859	Heilongjiang Lansi
50861	Heilongjiang Qingan
50862	Heilongjiang Tieli
50867	Heilongjiang Bayan
50871	Heilongjiang Tangyuan
50873	Heilongjiang Jiamusi
50877	Heilongjiang Yilang
50878	Heilongjiang Huachuan
50879	Heilongjiang Huanan
50884	Heilongjiang Shuangyashan
50888	Heilongjiang Baoqing
50892	Heilongjiang Raohe
50950	Heilongjiang Zhaozhou
50953	Heilongjiang Haerbin
50956	Heilongjiang Hulan
50958	Heilongjiang Acheng
50960	Heilongjiang Binxian
50962	Heilongjiang Mulan
50963	Heilongjiang Tonghe
50964	Heilongjiang Zhengfang
50965	Heilongjiang Yanshou
50968	Heilongjiang Shangzhi
50971	Heilongjiang Qitaihe
50973	Heilongjiang Boli
50978	Heilongjiang Jixi
50979	Heilongjiang Linkou
50983	Heilongjiang Hulin
50985	Heilongjiang Mishan
50987	Heilongjiang Jidong
54080	Heilongjiang Wuchang
54092	Heilongjiang Hailin
54093	Heilongjiang Muling
54094	Heilongjiang Mudanjiang
54096	Heilongjiang Suifenhe
54098	Heilongjiang Ningan

57249	Hubei Zhuxi
57251	Hubei Yunxi
57253	Hubei Yunxian
57256	Hubei Shiyan
57257	Hubei Zhushan
57259	Hubei Fangxian
57260	Hubei Danjiangkou
57268	Hubei Gucheng
57278	Hubei Xiangyang
57279	Hubei Zaoyang
57355	Hubei Padang
57359	Hubei Xingshan
57361	Hubei Baokang
57362	Hubei Shennongjia
57363	Hubei Nanzhang
57370	Hubei Yicheng
57377	Hubei Jingmen
57378	Hubei Zhongxiang
57381	Hubei Suizhou
57386	Hubei Xiaochang
57387	Hubei Jingshan
57388	Hubei Anlu
57398	Hubei Hongan
57399	Hubei Macheng
57439	Hubei Lichuan
57445	Hubei Jianshi
57447	Hubei Enshi
57453	Hubei Yiling
57458	Hubei Wufeng
57469	Hubei Songzi
57475	Hubei Qianjiang
57477	Hubei Gongan
57481	Hubei Yingcheng
57482	Hubei Xiaogan
57483	Hubei Tianmen
57484	Hubei Shayang
57485	Hubei Xiantao
57486	Hubei Hanchuan
57489	Hubei Caidian
57492	Hubei Xinzhou
57494	Hubei Wuhan
57495	Hubei Tuanfeng
57496	Hubei Ezhou

57540	Hubei Xianfeng
57541	Hubei Xuanen
57543	Hubei Hefeng
57571	Hubei Shishou
57573	Hubei Jianli
57581	Hubei Honghu
57582	Hubei Chibi
57583	Hubei Jiayu
57586	Hubei Chongyang
57589	Hubei Tongcheng
57590	Hubei Xianning
58401	Hubei Luotian
58402	Hubei Yingshan
58408	Hubei Qichun
58409	Hubei Huangmei
58500	Hubei Yangxin
58501	Hubei Wuxue
57544	Hunan Longshan
57554	Hunan Sangzhi
57558	Hunan Zhangjiajie
57565	Hunan Lixian
57574	Hunan Nanxian
57575	Hunan Huarong
57584	Hunan Yueyang
57642	Hunan Baojing
57643	Hunan Yongshun
57646	Hunan Guzhang
57649	Hunan Jishou
57655	Hunan Yuanling
57657	Hunan Luxi
57661	Hunan Taoyuan
57662	Hunan Changde
57663	Hunan Hanshou
57666	Hunan Taojiang
57669	Hunan Anhua
57671	Hunan Yuanjiang
57673	Hunan Xiangyin
57678	Hunan Ningxiang
57680	Hunan Milo
57682	Hunan Pingjiang
57687	Hunan Changsha
57688	Hunan Liuyang
57740	Hunan Fenghuang

57744	Hunan Xinhuang
57745	Hunan Zhijiang
57752	Hunan Xupu
57754	Hunan Hongjiang
57760	Hunan Lengshuijiang
57761	Hunan Xinhua
57763	Hunan Loudi
57771	Hunan Shaoshan
57772	Hunan Xiangxiang
57774	Hunan Shuangfeng
57776	Hunan Nanyue
57777	Hunan Hengshan
57779	Hunan Youxian
57780	Hunan Zhuzhou
57781	Hunan Liling
57845	Hunan Tongtao
57865	Hunan Lengshuitan
57866	Hunan Yongzhou
57867	Hunan Dongan
57868	Hunan Qiyang
57870	Hunan Qidong
57871	Hunan Hengyangxian
57872	Hunan Hengyang
57874	Hunan Changning
57875	Hunan Hengnan
57881	Hunan Anren
57882	Hunan Chaling
57887	Hunan Yongxing
57889	Hunan Guangxi
57962	Hunan Shuangpai
57965	Hunan Daoxian
57966	Hunan Ningyuan
57969	Hunan Jiangyong
57971	Hunan Xintian
57972	Hunan Chenzhou
57973	Hunan Guiyang
57974	Hunan Jiahe
57975	Hunan Lanshan
57976	Hunan Yizhang
57978	Hunan Linwu
57981	Hunan Zixing
57985	Hunan Rucheng
59063	Hunan Jianghua

50936	Jilin Baicheng
50939	Jilin Taonan
50940	Jilin Zhenlai
50945	Jilin Daan
50946	Jilin Songyuan
50948	Jilin Qianan
50949	Jilin Qianguo
54041	Jilin Tongyu
54049	Jilin Changling
54063	Jilin Fuyu
54064	Jilin Nongan
54065	Jilin Dehui
54069	Jilin Jiutai
54072	Jilin Yushu
54076	Jilin Shulan
54142	Jilin Shuangliao
54154	Jilin Lishu
54155	Jilin Gujiazi
54161	Jilin Changchun
54164	Jilin Yitong
54165	Jilin Shuangyang
54169	Jilin Yantongshan
54171	Jilin Yongji
54172	Jilin Jilin Suburb
54181	Jilin Jiaohe
54186	Jilin Dunhua
54187	Jilin Antu
54192	Jilin Luozigou
54195	Jilin Wangqing
54260	Jilin Liaoyuan
54261	Jilin Tongfeng
54263	Jilin Panshi
54267	Jilin Liuhe
54273	Jilin Huadian
54274	Jilin Huinan
54279	Jilin Jiangyuan
54284	Jilin Donggang
54285	Jilin Erdao
54286	Jilin Helong
54290	Jilin Longjing
54291	Jilin Huichun
54292	Jilin Yanji
54362	Jilin Tonghuaxian

54363	Jilin Tonghua
54371	Jilin Baishan
54374	Jilin Linjiang
54377	Jilin Jian
54386	Jilin Changbai
58012	Jiangsu Fengxian
58013	Jiangsu Peixian
58026	Jiangsu Pizhou
58027	Jiangsu Xuzhou
58036	Jiangsu Donghai
58038	Jiangsu Shuyang
58040	Jiangsu Ganyu
58041	Jiangsu Xiliandao
58044	Jiangsu Lianyungang
58045	Jiangsu Xiangshui
58047	Jiangsu Guanyun
58130	Jiangsu Suining
58131	Jiangsu Suyu
58132	Jiangsu Siyang
58135	Jiangsu Sihong
58138	Jiangsu Xuyi
58139	Jiangsu Hongze
58140	Jiangsu Lianshui
58141	Jiangsu Huaian
58143	Jiangsu Funing
58146	Jiangsu Jianhu
58147	Jiangsu Jinhu
58148	Jiangsu Baoying
58154	Jiangsu Yancheng
58158	Jiangsu Dafeng
58235	Jiangsu Liuhe
58237	Jiangsu Pukou
58238	Jiangsu Nanjing
58241	Jiangsu Gaoyou
58242	Jiangsu Yizheng
58243	Jiangsu Xinghua
58246	Jiangsu Taizhou
58247	Jiangsu Yangzhong
58249	Jiangsu Taixing
58250	Jiangsu Jiangyan
58251	Jiangsu Dongtai
58254	Jiangsu Haian
58255	Jiangsu Rugao

58257	Jiangsu Jingjiang
58259	Jiangsu Nantong
58264	Jiangsu Rudong
58265	Jiangsu Lvsi
58269	Jiangsu Qidong
58340	Jiangsu Lishui
58341	Jiangsu Danyang
58342	Jiangsu Jintan
58344	Jiangsu Jurong
58346	Jiangsu Yixing
58349	Jiangsu Suzhou
58352	Jiangsu Changshu
58353	Jiangsu Zhangjiagang
58354	Jiangsu Wuxi
58356	Jiangsu Kunshan
58360	Jiangsu Haimen
58377	Jiangsu Taicang
57598	Jiangxi Xiushui
57694	Jiangxi Tonggu
57698	Jiangxi Wanzai
57699	Jiangxi Shanggao
57786	Jiangxi Pingxiang
57789	Jiangxi Lianhua
57792	Jiangxi Fenyi
57793	Jiangxi Yichun
57796	Jiangxi Xinyu
57798	Jiangxi Anfu
57799	Jiangxi Jianxian
57883	Jiangxi Xiaping
57891	Jiangxi Yongxin
57895	Jiangxi Wanan
57896	Jiangxi Suichuan
57899	Jiangxi Taihe
57990	Jiangxi Chongyi
57992	Jiangxi Nankang
57993	Jiangxi Ganxian
57995	Jiangxi Xinfeng
58502	Jiangxi Jiujiang
58503	Jiangxi Ruichang
58506	Jiangxi Lushan
58507	Jiangxi Wuning
58508	Jiangxi Dean
58510	Jiangxi Hukou

58512	Jiangxi Pengze
58517	Jiangxi Duchang
58519	Jiangxi Poyang
58527	Jiangxi Jingdezhen
58529	Jiangxi Wuyuan
58600	Jiangxi Jingan
58601	Jiangxi Fengxin
58602	Jiangxi Anyi
58605	Jiangxi Gaoan
58606	Jiangxi Nanchang
58612	Jiangxi Yugan
58614	Jiangxi Jinxian
58615	Jiangxi Wannian
58618	Jiangxi Dongxiang
58619	Jiangxi Linchuan
58622	Jiangxi Dexing
58623	Jiangxi Shangraoxian
58626	Jiangxi Guixi
58629	Jiangxi Qianshan
58634	Jiangxi Yushan
58693	Jiangxi Xinjian
58704	Jiangxi Xiajiang
58705	Jiangxi Yongfeng
58706	Jiangxi Lean
58710	Jiangxi Chongren
58712	Jiangxi Jinxi
58715	Jiangxi Nancheng
58718	Jiangxi Nanfeng
58719	Jiangxi Lichuan
58804	Jiangxi Xingguo
58806	Jiangxi Ningdu
58813	Jiangxi Guangchang
58814	Jiangxi Shicheng
58905	Jiangxi Yudu
58906	Jiangxi Huichang
58907	Jiangxi Anyuan
59091	Jiangxi Quannan
59092	Jiangxi Longnan
59093	Jiangxi Dingnan
59102	Jiangxi Xunwu
57783	Jiangxi Shangli
54236	Liaoning Zhangwu
54243	Liaoning Changtu

54244	Liaoning Kangping
54248	Liaoning Shenbei
54249	Liaoning Tieling
54252	Liaoning Xifeng
54259	Liaoning Qingyuan
54321	Liaoning Jianpingzhen
54324	Liaoning Chaoyang
54325	Liaoning Yangshan
54326	Liaoning Jianpingxian
54327	Liaoning Lingyuan
54332	Liaoning Liaozhong
54333	Liaoning Xinmin
54336	Liaoning Taian
54338	Liaoning Panshan
54339	Liaoning Anshan
54340	Liaoning Sujiatun
54342	Liaoning Shenyang
54345	Liaoning Liaoyangxian
54346	Liaoning Benxi
54351	Liaoning Fushun
54353	Liaoning Xinbin
54452	Liaoning Jianchang
54453	Liaoning Lianshan
54454	Liaoning Suizhong
54455	Liaoning Xingcheng
54471	Liaoning Yingkou
54474	Liaoning Gaizhou
54475	Liaoning Dashiqiao
54483	Liaoning Caohekou
54486	Liaoning Xiuyan
54493	Liaoning Kuandian
54494	Liaoning Fengcheng
54497	Liaoning Dandong
54563	Liaoning Wafangdian
54568	Liaoning Jinzhou
54569	Liaoning Pulandian
54575	Liaoning Pikou
54579	Liaoning Changhai
54584	Liaoning Zhuanghe
54565	Liaoning Changxingdao
50425	Inner Mongolia Eerguna
50434	Inner Mongolia Tulihe
50445	Inner Mongolia Elunchunqi

50514	Inner Mongolia Manzhouli
50525	Inner Mongolia Evenkeqi
50526	Inner Mongolia Yakeshi
50548	Inner Mongolia Xiaoergou
50603	Inner Mongolia Xinbaerhuyouqi
50618	Inner Mongolia Xinbaerhuzuoqi
50639	Inner Mongolia Zhalantun
50645	Inner Mongolia Moulidawawoer
50647	Inner Mongolia Arunqi
50727	Inner Mongolia Aershan
50834	Inner Mongolia Suolun
50913	Inner Mongolia Wulagai
50915	Inner Mongolia Wuzhumuqindong
50924	Inner Mongolia Houlinguole
50928	Inner Mongolia Bayaertuhushuo
50934	Inner Mongolia Tuquan
53068	Inner Mongolia Erlianhaote
53083	Inner Mongolia Narenbaolige
53149	Inner Mongolia Mandula
53192	Inner Mongolia Abagaqi
53231	Inner Mongolia Hailisu
53289	Inner Mongolia Xianghuangqi
53336	Inner Mongolia Wulatezhongqi
53337	Inner Mongolia Wuyuan
53348	Inner Mongolia Dashetai
53352	Inner Mongolia Damaoqi
53357	Inner Mongolia Guyangxian
53362	Inner Mongolia Siziwang
53367	Inner Mongolia Xilamuren
53368	Inner Mongolia Wuchuanxian
53378	Inner Mongolia Chayouzhongqi
53384	Inner Mongolia Chayouhouqi
53385	Inner Mongolia Shangdu
53391	Inner Mongolia Huade
53419	Inner Mongolia Dengkou
53420	Inner Mongolia Hangjinhouqi
53433	Inner Mongolia Wulateqianqi
53446	Inner Mongolia Baotou
53455	Inner Mongolia Tuyouqi
53457	Inner Mongolia Dalateqi
53463	Inner Mongolia Huhehaote
53464	Inner Mongolia Tumutezuoqi
53466	Inner Mongolia Huhehaote suburb

53467	Inner Mongolia Tuoketuoxian
53469	Inner Mongolia Helingeerxian
53472	Inner Mongolia Zhuozi
53475	Inner Mongolia Liangcheng
53480	Inner Mongolia Jining
53481	Inner Mongolia Chayouqianqi
53483	Inner Mongolia Xinghe
53512	Inner Mongolia Wuhai
53513	Inner Mongolia Linhe
53522	Inner Mongolia Yikewusu
53529	Inner Mongolia Etukeqi
53533	Inner Mongolia Hangjinqi
53543	Inner Mongolia Dongsheng
53545	Inner Mongolia Ejinhuoluoqi
53553	Inner Mongolia Zhungeerqi
53562	Inner Mongolia Qingshuihexian
53644	Inner Mongolia Wushenqi
53730	Inner Mongolia Eduokeqianqi
53732	Inner Mongolia Henan
54012	Inner Mongolia Xiwuzhumuqin
54024	Inner Mongolia Fuhe
54026	Inner Mongolia Zhalute
54027	Inner Mongolia Balinzuoqi
54031	Inner Mongolia Gaoliban
54039	Inner Mongolia Shebotu
54047	Inner Mongolia Kezuozhongqi
54102	Inner Mongolia Xilinhaote
54113	Inner Mongolia Balinyouqi
54115	Inner Mongolia Linxixian
54117	Inner Mongolia Keshiketengqi
54122	Inner Mongolia Alukeerqinqi
54132	Inner Mongolia Qinglongshan
54134	Inner Mongolia Kailu
54135	Inner Mongolia Tongliao
54204	Inner Mongolia Zhengxiangbaiqi
54205	Inner Mongolia Zhenglanqi
54208	Inner Mongolia Duolunxian
54213	Inner Mongolia Wengniuteqi
54214	Inner Mongolia Gangzi
54218	Inner Mongolia Chifeng
54223	Inner Mongolia Neiman
54225	Inner Mongolia Aohanqi
54226	Inner Mongolia Baoguotu

54231	Inner Mongolia Kezuohouqi
54234	Inner Mongolia Kulun
54305	Inner Mongolia Taibushiqi
54313	Inner Mongolia Kalaqinqi
54316	Inner Mongolia Balihan
54320	Inner Mongolia Ningchengxian
53517	Ningxia Shitanjing
53519	Ningxia Huinong
53610	Ningxia Helan
53611	Ningxia Pingluo
53612	Ningxia Wuzhong
53615	Ningxia Taole
53618	Ningxia Yongning
53704	Ningxia Zhongwei
53705	Ningxia Zhongning
53707	Ningxia Xingren
53723	Ningxia Yanchi
53727	Ningxia Maihuangshan
53806	Ningxia Haiyuan
53810	Ningxia Tongxin
53817	Ningxia Guyuan
53881	Ningxia Weizhou
53903	Ningxia Xiji
53910	Ningxia Liupanshan
52602	Qinghai Lenghu
52825	Qinghai Nuomuhong
52836	Qinghai Doulan
52854	Qinghai Qinghaihu 151
52856	Qinghai Gonghe
52863	Qinghai Huzhu
52866	Qinghai Xining
52868	Qinghai Guide
52875	Qinghai Pingan
52876	Qinghai Minhe
52877	Qinghai Hualong
52908	Qinghai Wudaoliang
52941	Qinghai Shazhuyu
52943	Qinghai Xinghai
52955	Qinghai Guinan
52957	Qinghai Tongde
52963	Qinghai Jianzha
52968	Qinghai Zeku
52972	Qinghai Xunhua

52974	Qinghai Tongren
56004	Qinghai Tuotuohe
56016	Qinghai Zhiduo
56018	Qinghai Zaduo
56021	Qinghai Qumacai
56029	Qinghai Yushu
56033	Qinghai Maduo
56034	Qinghai Qingshuihe
56043	Qinghai Maqin
56045	Qinghai Gander
56046	Qinghai Dari
56065	Qinghai Henan
56067	Qinghai Jiuzhi
56125	Qinghai Nangqian
56151	Qinghai Banma
54712	Shandong Linyi
54716	Shandong Ningjin
54734	Shandong Binzhou
54751	Shandong Longdao
54752	Shandong Penglai
54764	Shandong Fushan
54765	Shandong Yantai
54776	Shandong Chengshantou
54777	Shandong Wendeng
54802	Shandong Linqing
54808	Shandong Xinxian
54812	Shandong Qihe
54814	Shandong Chiping
54815	Shandong Donge
54821	Shandong Jiyang
54822	Shandong Zouping
54827	Shandong Taian
54830	Shandong Zibo
54832	Shandong Shouguang
54833	Shandong Huantai
54836	Shandong Yiyuan
54841	Shandong Changyi
54842	Shandong Pingdu
54843	Shandong Weifang
54846	Shandong Gaomi
54849	Shandong Jiaozhou
54855	Shandong Jimo
54904	Shandong Juancheng

54905	Shandong Yuncheng
54907	Shandong Yutai
54909	Shandong Dingtao
54912	Shandong Wenshang
54916	Shandong Yanzhou
54919	Shandong Zoucheng
54923	Shandong Mengyin
54927	Shandong Tengzhou
54938	Shandong Linyi
54939	Shandong Junan
54945	Shandong Rizhao
58002	Shandong Caoxian
58003	Shandong Chengwu
58021	Shandong Xuechen
58022	Shandong Yicheng
58024	Shandong Zaozhuang
58030	Shandong Cangshan
58032	Shandong Linshu
53486	Shanxi Yanggao
53487	Shanxi Datong
53564	Shanxi Hequ
53565	Shanxi Pianguan
53576	Shanxi Shanyin
53659	Shanxi Linxian
53662	Shanxi Kelan
53676	Shanxi Dingxiang
53753	Shanxi Liulin
53759	Shanxi Shilou
53760	Shanxi Fangshan
53763	Shanxi Gujiao
53764	Shanxi Lishi
53774	Shanxi Qingxu
53788	Shanxi Heshun
53852	Shanxi Yonghe
53859	Shanxi Jixian
53861	Shanxi Xiangfen
53865	Shanxi Fenxi
53866	Shanxi Hongtong
53878	Shanxi Licheng
53953	Shanxi Xiangning
53954	Shanxi Jishan
53956	Shanxi Wanrong
53957	Shanxi Hejin

53959	Shanxi Yanhu
53965	Shanxi Jiangxian
53966	Shanxi Fushan
53970	Shanxi Qingshui
53973	Shanxi Gaoping
53981	Shanxi Lingchuan
57052	Shanxi Yongji
57053	Shanxi Ruicheng
53567	Shaanxi Fugu
53646	Shaanxi Yulin
53651	Shaanxi Shenmu
53658	Shaanxi Jiaxian
53725	Shaanxi Dingbian
53735	Shaanxi Jingbian
53738	Shaanxi Wuqi
53740	Shaanxi Hengshan
53748	Shaanxi Zichang
53754	Shaanxi Suide
53756	Shaanxi Wubao
53757	Shaanxi Qingjian
53832	Shaanxi Zhidan
53841	Shaanxi Ansai
53848	Shaanxi Ganquan
53850	Shaanxi Yanchuan
53854	Shaanxi Yanchang
53857	Shaanxi Yichuan
53931	Shaanxi Fuxian
53938	Shaanxi Xunyi
53941	Shaanxi Baishui
53944	Shaanxi Huangling
53946	Shaanxi Huanglong
53947	Shaanxi Tongchuan
53948	Shaanxi Pucheng
53949	Shaanxi Chengcheng
53950	Shaanxi Heyang
57003	Shaanxi Longxian
57016	Shaanxi Baoji
57021	Shaanxi Qianyang
57022	Shaanxi Linyou
57026	Shaanxi Fufeng
57027	Shaanxi Meixian
57029	Shaanxi Liquan
57030	Shaanxi Yongshou

57034	Shaanxi Wugong
57037	Shaanxi Yaoxian
57038	Shaanxi Xingping
57041	Shaanxi Sanyuan
57042	Shaanxi Fuping
57043	Shaanxi Dali
57045	Shaanxi Weinan
57046	Shaanxi Huashan
57057	Shaanxi Luonan
57106	Shaanxi Lueyang
57113	Shaanxi Fengxian
57119	Shaanxi Mianxian
57124	Shaanxi Liuba
57128	Shaanxi Chenggu
57134	Shaanxi Foping
57137	Shaanxi Ningshan
57140	Shaanxi Zhashui
57143	Shaanxi Shangxian
57153	Shaanxi Danfeng
57154	Shaanxi Shangnan
57155	Shaanxi Shanyang
57211	Shaanxi Ningqiang
57213	Shaanxi Nanzheng
57231	Shaanxi Ziyang
57233	Shaanxi Hanyin
57238	Shaanxi Zhenba
57242	Shaanxi Xunyang
57245	Shaanxi Ankang
57247	Shaanxi Langao
57248	Shaanxi Pingli
57254	Shaanxi Baihe
57343	Shaanxi Zhenping
58361	Shanghai Minhang
58362	Shanghai Baoshan
58460	Shanghai Jinshan
58463	Shanghai Fengxian
56038	Sichuan Shiqu
56079	Sichuan Ruoergai
56097	Sichuan Jiuzhaigou
56146	Sichuan Ganzi
56152	Sichuan Seda
56158	Sichuan Luhuo
56164	Sichuan Rangtang

56167	Sichuan Daofu
56168	Sichuan Jinchuan
56171	Sichuan Aba
56172	Sichuan Maerkang
56173	Sichuan Hongyuan
56178	Sichuan Xiaojin
56180	Sichuan Maoxian
56181	Sichuan Chongzhou
56182	Sichuan Songpan
56183	Sichuan Wenchuan
56184	Sichuan Lixian
56185	Sichuan Heishui
56186	Sichuan Mianzhu
56187	Sichuan Wenjiang
56188	Sichuan Dujiangyan
56189	Sichuan Pengzhou
56198	Sichuan Deyang
56247	Sichuan Batang
56251	Sichuan Xinlong
56257	Sichuan Litang
56263	Sichuan Danba
56267	Sichuan Yajiang
56273	Sichuan Baoxing
56279	Sichuan Lushan
56280	Sichuan Mingshan
56281	Sichuan Pujiang
56286	Sichuan Longquanyi
56289	Sichuan Pengshan
56296	Sichuan Jintang
56297	Sichuan Renshou
56298	Sichuan Ziyang
56357	Sichuan Daocheng
56371	Sichuan Luding
56373	Sichuan Yingjing
56374	Sichuan Kangding
56376	Sichuan Hanyuan
56378	Sichuan Shimian
56380	Sichuan Hongya
56382	Sichuan Jiajiang
56383	Sichuan Qingshen
56385	Sichuan Emeishan
56387	Sichuan Ebian
56389	Sichuan Qianwei

56390	Sichuan Jingyan
56393	Sichuan Zizhong
56395	Sichuan Weiyuan
56396	Sichuan Zigong
56399	Sichuan Fushun
56441	Sichuan Derong
56443	Sichuan Xiangcheng
56459	Sichuan Muli
56462	Sichuan Jiulong
56473	Sichuan Ganluo
56474	Sichuan Mianning
56475	Sichuan Yuexi
56478	Sichuan Xide
56479	Sichuan Chaojue
56480	Sichuan Mabian
56485	Sichuan Leibo
56487	Sichuan Meigu
56490	Sichuan Muchuan
56491	Sichuan Yibinxian
56493	Sichuan Nanxi
56494	Sichuan Pingshan
56496	Sichuan Xingwen
56499	Sichuan Gongxian
56565	Sichuan Yanyuan
56569	Sichuan Dechang
56571	Sichuan Xichang
56575	Sichuan Puge
56580	Sichuan Butuo
56584	Sichuan Jinyang
56593	Sichuan Changning
56665	Sichuan Yanbian
56666	Sichuan Panzhihua
56670	Sichuan Miyi
56671	Sichuan Huili
56675	Sichuan Huidong
57204	Sichuan Qingchuan
57206	Sichuan Guangyuan
57208	Sichuan Jiange
57216	Sichuan Nanjiang
57217	Sichuan Wangcang
57237	Sichuan Wanyuan
57306	Sichuan Langzhong
57309	Sichuan Xichong

57313	Sichuan Bazhong
57314	Sichuan Nanbu
57315	Sichuan Yilong
57318	Sichuan Yingshan
57320	Sichuan Tongjiang
57324	Sichuan Pingchang
57328	Sichuan Dachuan
57329	Sichuan Kaijiang
57401	Sichuan Shehong
57405	Sichuan Suining
57411	Sichuan Gaoping
57413	Sichuan Quxian
57415	Sichuan Guangan
57416	Sichuan Linshui
57417	Sichuan Wusheng
57420	Sichuan Dazhu
57503	Sichuan Dongxing
57507	Sichuan Longchang
57600	Sichuan Jiangan
57603	Sichuan Hejiang
57608	Sichuan Xuyong
54523	Tianjin Wuqing
54619	Tianjin Jinghai
54622	Tianjin Jinnan
54623	Tianjin Tanggu
54645	Tianjin Dagang
55655	Tibet Nielamu
51053	Xinjiang Habahe
51058	Xinjiang Akedala
51060	Xinjiang Buerjin
51068	Xinjiang Fuhai
51076	Xinjiang Aletai
51087	Xinjiang Fuyun
51133	Xinjiang Tacheng
51137	Xinjiang Yumin
51145	Xinjiang Emin
51156	Xinjiang Hebukesaier
51232	Xinjiang Alashankou
51238	Xinjiang Bole
51241	Xinjiang Touli
51243	Xinjiang Kelamayi
51334	Xinjiang Jinghe
51357	Xinjiang Shawan

51359	Xinjiang Manasi
51367	Xinjiang Hutubi
51368	Xinjiang Changji
51369	Xinjiang Miquan
51377	Xinjiang Fukang
51378	Xinjiang Jimusaer
51468	Xinjiang Tianshandaxigou
51469	Xinjiang Urumqi Pastoral Test Station
51470	Xinjiang Tianchi
51477	Xinjiang Dabancheng
51482	Xinjiang Mulei
51526	Xinjiang Kumishi
51542	Xinjiang Bayinbuluke
51567	Xinjiang Yanqi
51571	Xinjiang Tuokexun
51572	Xinjiang Tulufandongkan
51573	Xinjiang Tulufan
51627	Xinjiang Wushi
51628	Xinjiang Akesu
51636	Xinjiang Xinhe
51639	Xinjiang Shaya
51656	Xinjiang Kuerle
51704	Xinjiang Atushi
51705	Xinjiang Wuqia
51707	Xinjiang Jiashi
51709	Xinjiang Kashi
51717	Xinjiang Yuepuhu
51720	Xinjiang Kepin
51722	Xinjiang Awati
51730	Xinjiang Alaer
51747	Xinjiang Tazhong
51765	Xinjiang Tieqianlike
51802	Xinjiang Yengjisha
51810	Xinjiang Maigaiti
51811	Xinjiang Shashe
51814	Xinjiang Yecheng
51815	Xinjiang Zepu
51818	Xinjiang Pishan
51826	Xinjiang Cele
51827	Xinjiang Moyu
51828	Xinjiang Hetan
51829	Xinjiang Luopu
51839	Xinjiang Minfeng

51855	Xinjiang Qiemuo
51931	Xinjiang Yutian
52101	Xinjiang Balikun
52112	Xinjiang Naomaohu
52118	Xinjiang Yiwu
52203	Xinjiang Hami
52313	Xinjiang Hongliuhe
56444	Yunnan Deqin
56483	Yunnan Suijiang
56497	Yunnan Yanjin
56533	Yunnan Gongshan
56543	Yunnan Xianggelila
56548	Yunnan Weixi
56567	Yunnan Ninglang
56582	Yunnan Daguan
56585	Yunnan Ludian
56594	Yunnan Yiliang
56596	Yunnan Weixin
56641	Yunnan Fugong
56643	Yunnan Liuku
56645	Yunnan Lanping
56646	Yunnan Jianchuan
56649	Yunnan Eryuan
56651	Yunnan Lijiang
56652	Yunnan Yongsheng
56654	Yunnan Heqing
56664	Yunnan Huaping
56669	Yunnan Yongren
56673	Yunnan Qiaojia
56688	Yunnan Dongchuan
56697	Yunnan Xuanwei
56739	Yunnan Tengchong
56742	Yunnan Yunlong
56745	Yunnan Yangbi
56746	Yunnan Yongping
56748	Yunnan Baoshan
56751	Yunnan Dali
56752	Yunnan Binchuan
56755	Yunnan Midu
56757	Yunnan Weishan
56764	Yunnan Yaoan
56766	Yunnan Mouding
56767	Yunnan Nanhua

56772	Yunnan Fumin
56774	Yunnan Wuding
56777	Yunnan Lufeng
56778	Yunnan Kunming
56782	Yunnan Malong
56783	Yunnan Qujing
56785	Yunnan Songming
56790	Yunnan Fuyuan
56835	Yunnan Longchuan
56836	Yunnan Yingjiang
56839	Yunnan Zhenkang
56840	Yunnan Lianghe
56841	Yunnan Longling
56842	Yunnan Shidian
56843	Yunnan Changning
56846	Yunnan Fengqing
56849	Yunnan Yongde
56854	Yunnan Yunxian
56856	Yunnan Jingdong
56862	Yunnan Shuangbai
56863	Yunnan Anning
56867	Yunnan Zhenyuan
56869	Yunnan Xinping
56870	Yunnan Yimen
56871	Yunnan Jinning
56873	Yunnan Chengjiang
56875	Yunnan Yuxi
56879	Yunnan Huaning
56880	Yunnan Yiliang
56881	Yunnan Shilin
56883	Yunnan Shizong
56885	Yunnan Mile
56886	Yunnan Luxi
56889	Yunnan Qiubei
56891	Yunnan Luoping
56898	Yunnan Eshan
56944	Yunnan Cangyuan
56946	Yunnan Gengma
56948	Yunnan Ximeng
56949	Yunnan Menglian
56950	Yunnan Shuangjiang
56951	Yunnan Lincang
56952	Yunnan Jinggu

56954	Yunnan Lancang
56958	Yunnan Menghai
56962	Yunnan Mojiang
56964	Yunnan Simao
56966	Yunnan Yuanjiang
56969	Yunnan Mengla
56970	Yunnan Shiping
56976	Yunnan Yuanyang
56977	Yunnan Jiangcheng
56978	Yunnan Luchun
56982	Yunnan Kaiyuan
56984	Yunnan Gejiu
56987	Yunnan Jinping
56991	Yunnan Yanshan
56992	Yunnan Xichou
56994	Yunnan Wenshan
59007	Yunnan Guangnan
59205	Yunnan Funing
58443	Zhejiang Changxing
58446	Zhejiang Anji
58448	Zhejiang Linan
58449	Zhejiang Fuyang
58450	Zhejiang Huzhou
58451	Zhejiang Jiashan
58452	Zhejiang Jiaxing
58453	Zhejiang Shaoxing
58454	Zhejiang Deqing
58457	Zhejiang Hangzhou
58467	Zhejiang Cixi
58472	Zhejiang Shengsi
58484	Zhejiang Daishan
58537	Zhejiang Kaihua
58542	Zhejiang Tonglu
58544	Zhejiang Jiande
58546	Zhejiang Pujiang
58547	Zhejiang Longyou
58549	Zhejiang Jinhua
58550	Zhejiang Zhuji
58555	Zhejiang Xinchang
58556	Zhejiang Shengzhou
58558	Zhejiang Dongyang
58559	Zhejiang Tiantai
58560	Zhejiang Panan

58561	Zhejiang Zhenhai
58562	Zhejiang Yinzhou
58565	Zhejiang Fenghua
58566	Zhejiang Xiangshan
58567	Zhejiang Ninghai
58568	Zhejiang Sanmen
58569	Zhejiang Shipu
58570	Zhejiang Putuo
58631	Zhejiang Changshan
58632	Zhejiang Jiangshan
58642	Zhejiang Wuyi
58643	Zhejiang Yongkang
58644	Zhejiang Suichang
58646	Zhejiang Lishui
58647	Zhejiang Longquan
58652	Zhejiang Xianju
58654	Zhejiang Jinyun
58656	Zhejiang Leqing
58657	Zhejiang Qingtian
58658	Zhejiang Yongjia
58660	Zhejiang Linhai
58664	Zhejiang Wenling
58665	Zhejiang Hongjia
58666	Zhejiang Dachen
58742	Zhejiang Yunhe
58746	Zhejiang Taishun
58750	Zhejiang Wencheng
58751	Zhejiang Pingyang
58648	Zhejiang Jingning
57333	Chongqing Chengkou
57338	Chongqing Kaixian
57339	Chongqing Yunyang
57345	Chongqing Wuxi
57349	Chongqing Wushan
57409	Chongqing Tongnan
57425	Chongqing Dianjiang
57432	Chongqing Wanzhou
57437	Chongqing Zhongxian
57438	Chongqing Shizhu
57502	Chongqing Dazu
57505	Chongqing Rongchang
57506	Chongqing Yongchuan
57509	Chongqing Wansheng

57510	Chongqing Tongliang
57511	Chongqing Beibei
57513	Chongqing Yubei
57514	Chongqing Bishan
57517	Chongqing Jiangjin
57518	Chongqing Banan
57519	Chongqing Nanchuan
57520	Chongqing Changshou
57523	Chongqing Fengdu
57525	Chongqing Wulong
57536	Chongqing Qianjiang
57537	Chongqing Pengshui
57612	Chongqing Qijiang

Table S4. 1636 monitoring stations whose PM2.5 emissions are from electricity generation sector

58015	Anhui Dangshan
58016	Anhui Xiaoxian
58102	Anhui Bozhou
58108	Anhui Jieshou
58109	Anhui Taihe
58113	Anhui Suixi
58114	Anhui Woyang
58127	Anhui Huaiyuan
58202	Anhui Funan
58203	Anhui Fuyang
58240	Anhui Tianchang
58306	Anhui Jinzhai
58314	Anhui Huoshan
58334	Anhui Wuhu
58417	Anhui Susong
58441	Anhui Guangde
58442	Anhui Langxi
58520	Anhui Qimen
58523	Anhui Yixian
58530	Anhui Shexian
58531	Anhui Tunxi
54398	Beijing Shunyi
54399	Beijing Haidian
54406	Beijing Yanqing
54416	Beijing Miyun
54421	Beijing Miyunshangdianzi
54424	Beijing Pinggu
54431	Beijing Tongzhou
54499	Beijing Changping
54501	Beijing Zhaitang
54511	Beijing
54597	Beijing Xiayunling
58724	Fujian Guangze
58725	Fujian Shaowu
58730	Fujian Wuyishan
58731	Fujian Pucheng
58734	Fujian Jianyang
58735	Fujian Songxi
58736	Fujian Zhenghe
58744	Fujian Shouning

58747	Fujian Zhouning
58748	Fujian Fuan
58749	Fujian Zherong
58754	Fujian Fuding
58818	Fujian Ninghuai
58820	Fujian Taining
58822	Fujian Jianning
58823	Fujian Shunchang
58824	Fujian Mingxi
58828	Fujian Sanming
58836	Fujian Gutian
58837	Fujian Youxi
58839	Fujian Shuqing
58843	Fujian Xiapu
58844	Fujian Minhou
58846	Fujian Ningde
58848	Fujian Lianjiang
58911	Fujian Changting
58917	Fujian Wuping
58923	Fujian Datian
58926	Fujian Zhangping
58928	Fujian Huaan
58929	Fujian Anxi
58931	Fujian Jiuxianshan
58932	Fujian Yongtai
58938	Fujian Xiuyu
58941	Fujian Changle
58942	Fujian Fuqing
58944	Fujian Pingtan
58946	Fujian Putian
59113	Fujian Yongding
59122	Fujian Changtai
59124	Fujian Nanjing
59125	Fujian Pinghe
59129	Fujian Zhangpu
59130	Fujian Tongan
59131	Fujian Nanan
59133	Fujian Chongwu
59320	Fujian Zhaoan
59322	Fujian Yunxiao
52323	Gansu Mazongshan
52515	Gansu Subei
52533	Gansu Jiuquan

52546	Gansu Gaitai
52557	Gansu Linze
52643	Gansu Sunan
52656	Gansu Minle
52674	Gansu Yongchang
52679	Gansu Wuwei
52681	Gansu Minqin
52784	Gansu Gulang
52787	Gansu Wushaoling
52797	Gansu Jingtai
52881	Gansu Tianzhu
52884	Gansu Gaolan
52885	Gansu Yongdeng
52895	Gansu Jingyuan
52896	Gansu Baiyin
52978	Gansu Xiahe
52980	Gansu Yongjing
52981	Gansu Dongxiang
52982	Gansu Guanghe
52983	Gansu Yuzhong
52985	Gansu Hezheng
52986	Gansu Lintao
52988	Gansu Kangle
52993	Gansu Huining
52995	Gansu Anding
52996	Gansu Huajialing
52998	Gansu Weiyuan
53821	Gansu Huanxian
53829	Gansu Qingcheng
53906	Gansu Jingning
53915	Gansu Kongtong
53917	Gansu Zhuanglang
53923	Gansu Xifeng
53924	Gansu Lingtai
53925	Gansu Zhenyuan
53926	Gansu Jingchuan
53927	Gansu Huating
53930	Gansu Huachi
53934	Gansu Huishui
53935	Gansu Zhengning
56071	Gansu Luqu
56074	Gansu Maqu
56080	Gansu Hezuo

56081	Gansu Lintan
56082	Gansu Zhuoni
56084	Gansu Dibu
56091	Gansu Zhangxian
56092	Gansu Longxi
56093	Gansu Minxian
56094	Gansu Zhouqu
56095	Gansu Dangchang
56096	Gansu Wudu
56192	Gansu Wenxian
57001	Gansu Gangu
57002	Gansu Qinan
57004	Gansu Wushan
57006	Gansu Tianshui
57007	Gansu Lixian
57011	Gansu Qingshui
57012	Gansu Zhangjiachuan
57014	Gansu Maiji
57102	Gansu Chengxian
57105	Gansu Kangxian
57110	Gansu Huixian
57111	Gansu Liangdang
57988	Guangdong Lechang
57989	Guangdong Renhua
57996	Guangdong Nanxiong
59074	Guangdong Lianshan
59075	Guangdong Yangshan
59081	Guangdong Ruyuan
59082	Guangdong Shaoguan
59087	Guangdong Fogang
59088	Guangdong Yingde
59090	Guangdong Shixing
59094	Guangdong Wengyuan
59096	Guangdong Lianping
59099	Guangdong Heping
59106	Guangdong Pingyuan
59107	Guangdong Longchuan
59109	Guangdong Xingning
59114	Guangdong Jiaoling
59116	Guangdong Daipu
59117	Guangdong Meixian
59268	Guangdong Yunan
59270	Guangdong Huaiji

59271	Guangdong Guangning
59276	Guangdong Sihui
59279	Guangdong Sanshui
59280	Guangdong Qingyuan
59284	Guangdong Huadu
59285	Guangdong Conghua
59287	Guangdong Guangzhou
59289	Guangdong Dongguan
59290	Guangdong Longmen
59293	Guangdong Heyuan
59297	Guangdong Boluo
59303	Guangdong Wuhua
59304	Guangdong Zijin
59306	Guangdong Jiexi
59310	Guangdong Fengshun
59312	Guangdong Chaozhou
59313	Guangdong Raoping
59314	Guangdong Puning
59316	Guangdong Shantou
59456	Guangdong Xinyi
59462	Guangdong Luoding
59469	Guangdong Yangchun
59470	Guangdong Xinxing
59471	Guangdong Yunfu
59473	Guangdong Heshan
59475	Guangdong Kaiping
59476	Guangdong Xinhui
59477	Guangdong Enping
59481	Guangdong Panyu
59487	Guangdong Doumen
59488	Guangdong Zhuhai
59492	Guangdong Huidong
59493	Guangdong Shenzhen
59500	Guangdong Haifeng
59502	Guangdong Lufeng
59650	Guangdong Suixi
59653	Guangdong Gaozhou
59654	Guangdong Lianjiang
59655	Guangdong Huazhou
59656	Guangdong Wuchuan
59659	Guangdong Maoming
59750	Guangdong Leizhou
59754	Guangdong Xuwen

57859	Guangxi Ziyuan
57927	Guangxi Tiane
57941	Guangxi Sanjiang
57942	Guangxi Longsheng
57948	Guangxi Rongshui
57949	Guangxi Yongfu
57954	Guangxi Lingui
57955	Guangxi Xingan
57960	Guangxi Quanzhou
57964	Guangxi Guanyang
59004	Guangxi Xilin
59012	Guangxi Leye
59015	Guangxi Lingyun
59017	Guangxi Tianlin
59021	Guangxi Fengshan
59023	Guangxi Hechi
59027	Guangxi Bama
59034	Guangxi Yizhou
59037	Guangxi Duan
59038	Guangxi Xincheng
59041	Guangxi Liucheng
59045	Guangxi Luzhai
59053	Guangxi Pingle
59055	Guangxi Lipu
59057	Guangxi Jinxiu
59058	Guangxi Mengshan
59065	Guangxi Hezhou
59209	Guangxi Napo
59211	Guangxi Baise
59215	Guangxi Debao
59218	Guangxi Jingxi
59224	Guangxi Tiandong
59227	Guangxi Tiandeng
59229	Guangxi Longan
59230	Guangxi Mashan
59235	Guangxi Shanglin
59237	Guangxi Wuming
59238	Guangxi Binyang
59241	Guangxi Xiangzhou
59242	Guangxi Laibin
59246	Guangxi Wuxuan
59255	Guangxi Pingnan
59256	Guangxi Tengxian

59265	Guangxi Wuzhou
59266	Guangxi Cangwu
59419	Guangxi Pingxiang
59421	Guangxi Daxin
59429	Guangxi Shangsi
59431	Guangxi Nanning
59435	Guangxi Yongning
59441	Guangxi Hengxian
59446	Guangxi Lingshan
59448	Guangxi Pubei
59449	Guangxi Bobai
59451	Guangxi Beiliu
59452	Guangxi Rongxian
59454	Guangxi Cenxi
59457	Guangxi Luchuan
59632	Guangxi Qinzhou
59640	Guangxi Hepu
56598	Guizhou Hezhang
56691	Guizhou Weining
56792	Guizhou Puan
56793	Guizhou Panxian
57606	Guizhou Tongzi
57623	Guizhou Daozhen
57625	Guizhou Zhengan
57634	Guizhou Wuchuan
57636	Guizhou Yanhe
57637	Guizhou Dejiang
57647	Guizhou Songtao
57707	Guizhou Bijie
57708	Guizhou Dafang
57714	Guizhou Jinsha
57717	Guizhou Zunyi
57718	Guizhou Xifeng
57719	Guizhou Kaiyang
57722	Guizhou Meitan
57723	Guizhou Fenggang
57728	Guizhou Wengan
57729	Guizhou Yuqing
57731	Guizhou Sinan
57734	Guizhou Shiqian
57735	Guizhou Cengong
57736	Guizhou Jiangkou
57737	Guizhou Shibing

57739	Guizhou Yuping
57742	Guizhou Wanshan
57800	Guizhou Nayong
57803	Guizhou Xianxi
57805	Guizhou Zhijin
57806	Guizhou Anshun
57807	Guizhou Liuzhi
57811	Guizhou Xiuwen
57814	Guizhou Pingba
57821	Guizhou Fuquan
57822	Guizhou Huangping
57824	Guizhou Guiding
57825	Guizhou Kaili
57827	Guizhou Duyun
57832	Guizhou Sansui
57834	Guizhou Taijiang
57835	Guizhou Jianhe
57837	Guizhou Leishan
57839	Guizhou Liping
57840	Guizhou Tianzhu
57844	Guizhou Jinping
57900	Guizhou Qinglong
57903	Guizhou Guanling
57905	Guizhou Zhenfeng
57906	Guizhou Wangmo
57907	Guizhou Xingyi
57909	Guizhou Ceheng
57910	Guizhou Ziyun
57912	Guizhou Huishui
57913	Guizhou Longli
57916	Guizhou Luodian
57921	Guizhou Pingtang
57922	Guizhou Dushan
57923	Guizhou Sandu
57926	Guizhou Libo
57932	Guizhou Rongjiang
57936	Guizhou Congjiang
59758	Hainan Haikou
59842	Hainan Lingao
59843	Hainan Chengmai
59845	Hainan Danzhou
59847	Hainan Changjiang
59848	Hainan Baisha

59851	Hainan Dingan
59854	Hainan Tunchang
59855	Hainan Qonghai
59856	Hainan Wenchang
59945	Hainan Baoting
59951	Hainan Wanning
53392	Hebei Kangbao
53397	Hebei Shangyi
53399	Hebei Zhangbei
53492	Hebei Yangyuan
53498	Hebei Xuanhua
53499	Hebei Wanquan
53593	Hebei Weixian
53596	Hebei Shunping
53599	Hebei Laiyuan
53680	Hebei Lingshou
53682	Hebei Quyang
53688	Hebei Xingtang
53689	Hebei Jinzhou
53690	Hebei Fuping
53692	Hebei Tangxian
53696	Hebei Dingzhou
53699	Hebei Wuji
53773	Hebei Linzhang
53781	Hebei Shahe
53785	Hebei Baixiang
53789	Hebei Luancheng
53794	Hebei Longyao
53795	Hebei Zanhuang
53796	Hebei Ningjin
53799	Hebei Julu
53886	Hebei Shexian
53890	Hebei Wuan
53892	Hebei Handan
53893	Hebei Quzhou
53894	Hebei Fengfeng
53896	Hebei Weixian
54301	Hebei Guyuan
54304	Hebei Chongli
54308	Hebei Fengning
54311	Hebei Weichang
54318	Hebei Longhua
54319	Hebei Pingquan

54401	Hebei Zhangjiakou
54404	Hebei Chicheng
54405	Hebei Huailai
54408	Hebei Zhulu
54420	Hebei Luanping
54423	Hebei Chengde
54425	Hebei Xinglong
54429	Hebei Zunhua
54430	Hebei Chengdexian
54432	Hebei Kuancheng
54434	Hebei Qianxi
54436	Hebei Qinglong
54437	Hebei Luannan
54438	Hebei Lulong
54439	Hebei Qianan
54449	Hebei Qinhuangdao
54502	Hebei Zhuozhou
54503	Hebei Rongcheng
54506	Hebei Gaobeidian
54510	Hebei Dachang
54512	Hebei Guan
54519	Hebei Yongqing
54520	Hebei Sanhe
54521	Hebei Xianghe
54534	Hebei Tangshan
54535	Hebei Caofeidian
54539	Hebei Leting
54540	Hebei Changli
54541	Hebei Funing
54601	Hebei Xushui
54603	Hebei Goyang
54605	Hebei Anxin
54606	Hebei Raoyang
54608	Hebei Shenzhou
54610	Hebei Renqiu
54612	Hebei Wenan
54613	Hebei Dacheng
54614	Hebei Hejian
54615	Hebei Qingxian
54616	Hebei Cangzhou
54618	Hebei Botou
54624	Hebei Huanghua
54628	Hebei Haixing

54631	Hebei Guangzong
54633	Hebei Xinhe
54640	Hebei Jize
54700	Hebei Wuqiang
54701	Hebei Xinji
54702	Hebei Hengshui
54703	Hebei Wuyi
54706	Hebei Qinghe
54707	Hebei Gucheng
54710	Hebei Fucheng
54711	Hebei Jingxian
54713	Hebei Dongguang
54800	Hebei-Weixian
54801	Hebei Linxi
54804	Hebei Daming
54809	Hebei Guantao
53972	Henan Qinyang
53974	Henan Qixian
53978	Henan Jiyuan
53982	Henan Jiaozuo
53983	Henan Fengqiu
53984	Henan Xiuwu
53985	Henan Huixian
53986	Henan Xinxiang
53991	Henan Tangyin
53992	Henan Junxian
53993	Henan Neihuang
53998	Henan Changheng
54817	Henan Taiqian
54900	Henan Puyang
54902	Henan Qingfeng
54903	Henan Fanxian
57051	Henan Sanmenxia
57056	Henan Lingbao
57063	Henan Mianchi
57066	Henan Luoning
57070	Henan Xinan
57071	Henan Mengjin
57072	Henan Mengzhou
57074	Henan Yichuan
57075	Henan Ruzhou
57078	Henan Ruyang
57079	Henan Wenxian

57080	Henan Gongyi
57081	Henan Xingyang
57082	Henan Dengfeng
57087	Henan Changge
57089	Henan Xuchang
57090	Henan Zhongmou
57091	Henan Kaifeng
57093	Henan Lankao
57095	Henan Yanling
57096	Henan Qixian
57099	Henan Taikang
57156	Henan Xixia
57169	Henan Neixiang
57173	Henan Lushan
57175	Henan Zhenping
57176	Henan Nanzhao
57177	Henan Wugang
57178	Henan Nanyang
57179	Henan Fangcheng
57180	Henan Jiaxian
57182	Henan Xiangcheng
57183	Henan Linying
57184	Henan Yexian
57185	Henan Wuyang
57186	Henan Luohe
57187	Henan Sheqi
57188	Henan Xiping
57189	Henan Suiping
57192	Henan Huaiyang
57193	Henan Xihua
57197	Henan Runan
57271	Henan Xinye
57273	Henan Tanghe
57281	Henan Biyang
57285	Henan Tongbai
57290	Henan Zhumadian
57292	Henan Pingyu
57293	Henan Xincai
57295	Henan Zhengyang
57296	Henan Xixian
57297	Henan Xinyang
57299	Henan Guangshan
57390	Henan Jigongshan

57396	Henan Xixian
58004	Henan Minquan
58005	Henan Shangqiu
58006	Henan Yucheng
58007	Henan Echeng
58017	Henan Xiayi
58100	Henan Dancheng
58207	Henan Huangchuan
58208	Henan Gushi
58301	Henan Shangcheng
50136	Heilongjiang Mohe
50137	Heilongjiang Beijicun
50246	Heilongjiang Tahe
50247	Heilongjiang Huzhong
50349	Heilongjiang Xinlin
50353	Heilongjiang Huma
50442	Heilongjiang Jiagedaqi
50468	Heilongjiang Aihui
50557	Heilongjiang Nenjiang
50564	Heilongjiang Sunwu
50566	Heilongjiang Xunke
50646	Heilongjiang Nehe
50655	Heilongjiang Wudalianchi
50656	Heilongjiang Beian
50658	Heilongjiang Keshan
50659	Heilongjiang Kedong
50673	Heilongjiang Jiayin
50674	Heilongjiang Wuyiling
50739	Heilongjiang Longjiang
50741	Heilongjiang Gannan
50742	Heilongjiang Fuyu
50745	Heilongjiang Qiqihaer
50749	Heilongjiang Lindian
50750	Heilongjiang Yian
50755	Heilongjiang Baiquan
50756	Heilongjiang Hailun
50758	Heilongjiang Minshui
50767	Heilongjiang Suileng
50772	Heilongjiang Wuying
50774	Heilongjiang Yichun
50775	Heilongjiang Hegang
50776	Heilongjiang Luobei
50778	Heilongjiang Tongjiang

50779	Heilongjiang Fuyuan
50787	Heilongjiang Suibin
50788	Heilongjiang Fujin
50842	Heilongjiang Dumeng
50844	Heilongjiang Tailai
50850	Heilongjiang Daqing
50851	Heilongjiang Qinggang
50852	Heilongjiang Wangkui
50853	Heilongjiang Beilin
50854	Heilongjiang Anda
50858	Heilongjiang Zhaodong
50859	Heilongjiang Lansi
50861	Heilongjiang Qingan
50862	Heilongjiang Tieli
50867	Heilongjiang Bayan
50871	Heilongjiang Tangyuan
50873	Heilongjiang Jiamusi
50877	Heilongjiang Yilang
50878	Heilongjiang Huachuan
50879	Heilongjiang Huanan
50884	Heilongjiang Shuangyashan
50888	Heilongjiang Baoqing
50892	Heilongjiang Raohe
50950	Heilongjiang Zhaozhou
50953	Heilongjiang Haerbin
50956	Heilongjiang Hulan
50958	Heilongjiang Acheng
50960	Heilongjiang Binxian
50962	Heilongjiang Mulan
50963	Heilongjiang Tonghe
50964	Heilongjiang Zhengfang
50965	Heilongjiang Yanshou
50968	Heilongjiang Shangzhi
50971	Heilongjiang Qitaihe
50973	Heilongjiang Boli
50978	Heilongjiang Jixi
50979	Heilongjiang Linkou
50983	Heilongjiang Hulin
50985	Heilongjiang Mishan
50987	Heilongjiang Jidong
54080	Heilongjiang Wuchang
54092	Heilongjiang Hailin
54093	Heilongjiang Muling

54094	Heilongjiang Mudanjiang
54096	Heilongjiang Suifenhe
54098	Heilongjiang Ningan
57249	Hubei Zhuxi
57251	Hubei Yunxi
57253	Hubei Yunxian
57256	Hubei Shiyan
57257	Hubei Zhushan
57259	Hubei Fangxian
57260	Hubei Danjiangkou
57268	Hubei Gucheng
57278	Hubei Xiangyang
57279	Hubei Zaoyang
57355	Hubei Padang
57359	Hubei Xingshan
57361	Hubei Baokang
57362	Hubei Shennongjia
57363	Hubei Nanzhang
57370	Hubei Yicheng
57377	Hubei Jingmen
57378	Hubei Zhongxiang
57381	Hubei Suizhou
57386	Hubei Xiaochang
57387	Hubei Jingshan
57388	Hubei Anlu
57398	Hubei Hongan
57399	Hubei Macheng
57439	Hubei Lichuan
57445	Hubei Jianshi
57447	Hubei Enshi
57453	Hubei Yiling
57458	Hubei Wufeng
57469	Hubei Songzi
57475	Hubei Qianjiang
57477	Hubei Gongan
57481	Hubei Yingcheng
57482	Hubei Xiaogan
57483	Hubei Tianmen
57484	Hubei Shayang
57485	Hubei Xiantao
57486	Hubei Hanchuan
57489	Hubei Caidian
57492	Hubei Xinzhou

57494	Hubei Wuhan
57495	Hubei Tuanfeng
57496	Hubei Ezhou
57540	Hubei Xianfeng
57541	Hubei Xuanen
57543	Hubei Hefeng
57571	Hubei Shishou
57573	Hubei Jianli
57581	Hubei Honghu
57582	Hubei Chibi
57583	Hubei Jiayu
57586	Hubei Chongyang
57589	Hubei Tongcheng
57590	Hubei Xianning
58401	Hubei Luotian
58402	Hubei Yingshan
58408	Hubei Qichun
58409	Hubei Huangmei
58500	Hubei Yangxin
58501	Hubei Wuxue
57544	Hunan Longshan
57554	Hunan Sangzhi
57558	Hunan Zhangjiajie
57565	Hunan Lixian
57574	Hunan Nanxian
57575	Hunan Huarong
57584	Hunan Yueyang
57642	Hunan Baojing
57643	Hunan Yongshun
57646	Hunan Guzhang
57649	Hunan Jishou
57655	Hunan Yuanling
57657	Hunan Luxi
57661	Hunan Taoyuan
57662	Hunan Changde
57663	Hunan Hanshou
57666	Hunan Taojiang
57669	Hunan Anhua
57671	Hunan Yuanjiang
57673	Hunan Xiangyin
57678	Hunan Ningxiang
57680	Hunan Milo
57682	Hunan Pingjiang

57687	Hunan Changsha
57688	Hunan Liuyang
57740	Hunan Fenghuang
57744	Hunan Xinhuang
57745	Hunan Zhijiang
57752	Hunan Xupu
57754	Hunan Hongjiang
57760	Hunan Lengshuijiang
57761	Hunan Xinhua
57763	Hunan Loudi
57771	Hunan Shaoshan
57772	Hunan Xiangxiang
57774	Hunan Shuangfeng
57776	Hunan Nanyue
57777	Hunan Hengshan
57779	Hunan Youxian
57780	Hunan Zhuzhou
57781	Hunan Liling
57845	Hunan Tongtao
57865	Hunan Lengshuitan
57866	Hunan Yongzhou
57867	Hunan Dongan
57868	Hunan Qiyang
57870	Hunan Qidong
57871	Hunan Hengyangxian
57872	Hunan Hengyang
57874	Hunan Changning
57875	Hunan Hengnan
57881	Hunan Anren
57882	Hunan Chaling
57887	Hunan Yongxing
57889	Hunan Guangxi
57962	Hunan Shuangpai
57965	Hunan Daoxian
57966	Hunan Ningyuan
57969	Hunan Jiangyong
57971	Hunan Xintian
57972	Hunan Chenzhou
57973	Hunan Guiyang
57974	Hunan Jiahe
57975	Hunan Lanshan
57976	Hunan Yizhang
57978	Hunan Linwu

57981	Hunan Zixing
57985	Hunan Rucheng
59063	Hunan Jianghua
50936	Jilin Baicheng
50939	Jilin Taonan
50940	Jilin Zhenlai
50945	Jilin Daan
50946	Jilin Songyuan
50948	Jilin Qianan
50949	Jilin Qianguo
54041	Jilin Tongyu
54049	Jilin Changling
54063	Jilin Fuyu
54064	Jilin Nongan
54065	Jilin Dehui
54069	Jilin Jiutai
54072	Jilin Yushu
54076	Jilin Shulan
54142	Jilin Shuangliao
54154	Jilin Lishu
54155	Jilin Gujiazi
54161	Jilin Changchun
54164	Jilin Yitong
54165	Jilin Shuangyang
54169	Jilin Yantongshan
54171	Jilin Yongji
54172	Jilin Jilin Suburb
54181	Jilin Jiaohe
54186	Jilin Dunhua
54187	Jilin Antu
54192	Jilin Luozigou
54195	Jilin Wangqing
54260	Jilin Liaoyuan
54261	Jilin Tongfeng
54263	Jilin Panshi
54267	Jilin Liuhe
54273	Jilin Huadian
54274	Jilin Huinan
54279	Jilin Jiangyuan
54284	Jilin Donggang
54285	Jilin Erdao
54286	Jilin Helong
54290	Jilin Longjing

54291	Jilin Huichun
54292	Jilin Yanji
54362	Jilin Tonghuaxian
54363	Jilin Tonghua
54371	Jilin Baishan
54374	Jilin Linjiang
54377	Jilin Jian
54386	Jilin Changbai
58012	Jiangsu Fengxian
58013	Jiangsu Peixian
58026	Jiangsu Pizhou
58027	Jiangsu Xuzhou
58036	Jiangsu Donghai
58038	Jiangsu Shuyang
58040	Jiangsu Ganyu
58041	Jiangsu Xiliandao
58044	Jiangsu Lianyungang
58045	Jiangsu Xiangshui
58047	Jiangsu Guanyun
58130	Jiangsu Suining
58131	Jiangsu Suyu
58132	Jiangsu Siyang
58135	Jiangsu Sihong
58138	Jiangsu Xuyi
58139	Jiangsu Hongze
58140	Jiangsu Lianshui
58141	Jiangsu Huaian
58143	Jiangsu Funing
58146	Jiangsu Jianhu
58147	Jiangsu Jinhu
58148	Jiangsu Baoying
58154	Jiangsu Yancheng
58158	Jiangsu Dafeng
58235	Jiangsu Liuhe
58237	Jiangsu Pukou
58238	Jiangsu Nanjing
58241	Jiangsu Gaoyou
58242	Jiangsu Yizheng
58243	Jiangsu Xinghua
58246	Jiangsu Taizhou
58247	Jiangsu Yangzhong
58249	Jiangsu Taixing
58250	Jiangsu Jiangyan

58251	Jiangsu Dongtai
58254	Jiangsu Haian
58255	Jiangsu Rugao
58257	Jiangsu Jingjiang
58259	Jiangsu Nantong
58264	Jiangsu Rudong
58265	Jiangsu Lvsi
58269	Jiangsu Qidong
58340	Jiangsu Lishui
58341	Jiangsu Danyang
58342	Jiangsu Jintan
58344	Jiangsu Jurong
58346	Jiangsu Yixing
58349	Jiangsu Suzhou
58352	Jiangsu Changshu
58353	Jiangsu Zhangjiagang
58354	Jiangsu Wuxi
58356	Jiangsu Kunshan
58360	Jiangsu Haimen
58377	Jiangsu Taicang
57598	Jiangxi Xiushui
57694	Jiangxi Tonggu
57698	Jiangxi Wanzai
57699	Jiangxi Shanggao
57786	Jiangxi Pingxiang
57789	Jiangxi Lianhua
57792	Jiangxi Fenyi
57793	Jiangxi Yichun
57796	Jiangxi Xinyu
57798	Jiangxi Anfu
57799	Jiangxi Jianxian
57883	Jiangxi Xiaping
57891	Jiangxi Yongxin
57895	Jiangxi Wanan
57896	Jiangxi Suichuan
57899	Jiangxi Taihe
57990	Jiangxi Chongyi
57992	Jiangxi Nankang
57993	Jiangxi Ganxian
57995	Jiangxi Xinfeng
58502	Jiangxi Jiujiang
58503	Jiangxi Ruichang
58506	Jiangxi Lushan

58507	Jiangxi Wuning
58508	Jiangxi Dean
58510	Jiangxi Hukou
58512	Jiangxi Pengze
58517	Jiangxi Duchang
58519	Jiangxi Poyang
58527	Jiangxi Jingdezhen
58529	Jiangxi Wuyuan
58600	Jiangxi Jingan
58601	Jiangxi Fengxin
58602	Jiangxi Anyi
58605	Jiangxi Gaoan
58606	Jiangxi Nanchang
58612	Jiangxi Yugan
58614	Jiangxi Jinxian
58615	Jiangxi Wannian
58618	Jiangxi Dongxiang
58619	Jiangxi Linchuan
58622	Jiangxi Dexing
58623	Jiangxi Shangraoxian
58626	Jiangxi Guixi
58629	Jiangxi Qianshan
58634	Jiangxi Yushan
58693	Jiangxi Xinjian
58704	Jiangxi Xiajiang
58705	Jiangxi Yongfeng
58706	Jiangxi Lean
58710	Jiangxi Chongren
58712	Jiangxi Jinxi
58715	Jiangxi Nancheng
58718	Jiangxi Nanfeng
58719	Jiangxi Lichuan
58804	Jiangxi Xingguo
58806	Jiangxi Ningdu
58813	Jiangxi Guangchang
58814	Jiangxi Shicheng
58905	Jiangxi Yudu
58906	Jiangxi Huichang
58907	Jiangxi Anyuan
59091	Jiangxi Quannan
59092	Jiangxi Longnan
59093	Jiangxi Dingnan
59102	Jiangxi Xunwu

57783	Jiangxi Shangli
54236	Liaoning Zhangwu
54243	Liaoning Changtu
54244	Liaoning Kangping
54248	Liaoning Shenbei
54249	Liaoning Tieling
54252	Liaoning Xifeng
54259	Liaoning Qingyuan
54321	Liaoning Jianpingzhen
54324	Liaoning Chaoyang
54325	Liaoning Yangshan
54326	Liaoning Jianpingxian
54327	Liaoning Lingyuan
54332	Liaoning Liaozhong
54333	Liaoning Xinmin
54336	Liaoning Taian
54338	Liaoning Panshan
54339	Liaoning Anshan
54340	Liaoning Sujiatun
54342	Liaoning Shenyang
54345	Liaoning Liaoyangxian
54346	Liaoning Benxi
54351	Liaoning Fushun
54353	Liaoning Xinbin
54452	Liaoning Jianchang
54453	Liaoning Lianshan
54454	Liaoning Suizhong
54455	Liaoning Xingcheng
54471	Liaoning Yingkou
54474	Liaoning Gaizhou
54475	Liaoning Dashiqiao
54483	Liaoning Caohekou
54486	Liaoning Xiuyan
54493	Liaoning Kuandian
54494	Liaoning Fengcheng
54497	Liaoning Dandong
54563	Liaoning Wafangdian
54568	Liaoning Jinzhou
54569	Liaoning Pulandian
54575	Liaoning Pikou
54579	Liaoning Changhai
54584	Liaoning Zhuanghe
54565	Liaoning Changxingdao

50425	Inner Mongolia Eerguna
50434	Inner Mongolia Tulihe
50445	Inner Mongolia Elunchunqi
50514	Inner Mongolia Manzhouli
50525	Inner Mongolia Evenkeqi
50526	Inner Mongolia Yakeshi
50548	Inner Mongolia Xiaoergou
50603	Inner Mongolia Xinbaerhuyouqi
50618	Inner Mongolia Xinbaerhuzuoqi
50639	Inner Mongolia Zhalantun
50645	Inner Mongolia Moulidawawoer
50647	Inner Mongolia Arunqi
50727	Inner Mongolia Aershan
50834	Inner Mongolia Suolun
50913	Inner Mongolia Wulagai
50915	Inner Mongolia Wuzhumuqindong
50924	Inner Mongolia Houlinguole
50928	Inner Mongolia Bayaertuhushuo
50934	Inner Mongolia Tuquan
53068	Inner Mongolia Erlianhaote
53083	Inner Mongolia Narenbaolige
53149	Inner Mongolia Mandula
53192	Inner Mongolia Abagaqi
53231	Inner Mongolia Hailisu
53289	Inner Mongolia Xianghuangqi
53336	Inner Mongolia Wulatezhongqi
53337	Inner Mongolia Wuyuan
53348	Inner Mongolia Dashetai
53352	Inner Mongolia Damaoqi
53357	Inner Mongolia Guyangxian
53362	Inner Mongolia Siziwang
53367	Inner Mongolia Xilamuren
53368	Inner Mongolia Wuchuanxian
53378	Inner Mongolia Chayouzhongqi
53384	Inner Mongolia Chayouhouqi
53385	Inner Mongolia Shangdu
53391	Inner Mongolia Huade
53419	Inner Mongolia Dengkou
53420	Inner Mongolia Hangjinhouqi
53433	Inner Mongolia Wulateqianqi
53446	Inner Mongolia Baotou
53455	Inner Mongolia Tuyouqi
53457	Inner Mongolia Dalateqi

53463	Inner Mongolia Huhehaote
53464	Inner Mongolia Tumutezuoqi
53466	Inner Mongolia Huhehaote suburb
53467	Inner Mongolia Tuoketuoxian
53469	Inner Mongolia Helingeerxian
53472	Inner Mongolia Zhuozi
53475	Inner Mongolia Liangcheng
53480	Inner Mongolia Jining
53481	Inner Mongolia Chayouqianqi
53483	Inner Mongolia Xinghe
53512	Inner Mongolia Wuhai
53513	Inner Mongolia Linhe
53522	Inner Mongolia Yikewusu
53529	Inner Mongolia Etukeqi
53533	Inner Mongolia Hangjinqi
53543	Inner Mongolia Dongsheng
53545	Inner Mongolia Ejinhuoluoqi
53553	Inner Mongolia Zhungeerqi
53562	Inner Mongolia Qingshuihexian
53644	Inner Mongolia Wushenqi
53730	Inner Mongolia Eduokeqianqi
53732	Inner Mongolia Henan
54012	Inner Mongolia Xiwuzhumuqin
54024	Inner Mongolia Fuhe
54026	Inner Mongolia Zhalute
54027	Inner Mongolia Balinzuoqi
54031	Inner Mongolia Gaoliban
54039	Inner Mongolia Shebotu
54047	Inner Mongolia Kezuozhongqi
54102	Inner Mongolia Xilinhaote
54113	Inner Mongolia Balinyouqi
54115	Inner Mongolia Linxixian
54117	Inner Mongolia Keshiketengqi
54122	Inner Mongolia Alukeerqinqi
54132	Inner Mongolia Qinglongshan
54134	Inner Mongolia Kailu
54135	Inner Mongolia Tongliao
54204	Inner Mongolia Zhengxiangbaiqi
54205	Inner Mongolia Zhenglanqi
54208	Inner Mongolia Duolunxian
54213	Inner Mongolia Wengniuteqi
54214	Inner Mongolia Gangzi
54218	Inner Mongolia Chifeng

54223	Inner Mongolia Neiman
54225	Inner Mongolia Aohanqi
54226	Inner Mongolia Baoguotu
54231	Inner Mongolia Kezuohouqi
54234	Inner Mongolia Kulun
54305	Inner Mongolia Taibushiqi
54313	Inner Mongolia Kalaqinqi
54316	Inner Mongolia Balihan
54320	Inner Mongolia Ningchengxian
53517	Ningxia Shitanjing
53519	Ningxia Huinong
53610	Ningxia Helan
53611	Ningxia Pingluo
53612	Ningxia Wuzhong
53615	Ningxia Taole
53618	Ningxia Yongning
53704	Ningxia Zhongwei
53705	Ningxia Zhongning
53707	Ningxia Xingren
53723	Ningxia Yanchi
53727	Ningxia Maihuangshan
53806	Ningxia Haiyuan
53810	Ningxia Tongxin
53817	Ningxia Guyuan
53881	Ningxia Weizhou
53903	Ningxia Xiji
53910	Ningxia Liupanshan
52602	Qinghai Lenghu
52825	Qinghai Nuomuhong
52836	Qinghai Doulan
52854	Qinghai Qinghaihu 151
52856	Qinghai Gonghe
52863	Qinghai Huzhu
52866	Qinghai Xining
52868	Qinghai Guide
52875	Qinghai Pingan
52876	Qinghai Minhe
52877	Qinghai Hualong
52908	Qinghai Wudaoliang
52941	Qinghai Shazhuyu
52943	Qinghai Xinghai
52955	Qinghai Guinan
52957	Qinghai Tongde

52963	Qinghai Jianzha
52968	Qinghai Zeku
52972	Qinghai Xunhua
52974	Qinghai Tongren
56004	Qinghai Tuotuohe
56016	Qinghai Zhiduo
56018	Qinghai Zaduo
56021	Qinghai Qumacai
56029	Qinghai Yushu
56033	Qinghai Maduo
56034	Qinghai Qingshuihe
56043	Qinghai Maqin
56045	Qinghai Gander
56046	Qinghai Dari
56065	Qinghai Henan
56067	Qinghai Jiuzhi
56125	Qinghai Nangqian
56151	Qinghai Banma
54709	Shandong Wucheng
54712	Shandong Linyi
54716	Shandong Ningjin
54723	Shandong Yangxin
54724	Shandong Shanghe
54726	Shandong Leling
54727	Shandong Zhangqiu
54729	Shandong Gaoqing
54734	Shandong Binzhou
54744	Shandong Kenli
54749	Shandong Laizhou
54751	Shandong Longdao
54752	Shandong Penglai
54753	Shandong Longkou
54755	Shandong Zhaoyuan
54759	Shandong Qixia
54764	Shandong Fushan
54765	Shandong Yantai
54776	Shandong Chengshantou
54777	Shandong Wendeng
54802	Shandong Linqing
54806	Shandong Liaocheng
54808	Shandong Xinxian
54812	Shandong Qihe
54814	Shandong Chiping

54815	Shandong Donge
54819	Shandong Feicheng
54821	Shandong Jiyang
54822	Shandong Zouping
54823	Shandong Jinan
54827	Shandong Taian
54828	Shandong Laiwu
54830	Shandong Zibo
54831	Shandong Qingzhou
54832	Shandong Shouguang
54833	Shandong Huantai
54836	Shandong Yiyuan
54841	Shandong Changyi
54842	Shandong Pingdu
54843	Shandong Weifang
54844	Shandong Anqiu
54846	Shandong Gaomi
54848	Shandong Zhucheng
54849	Shandong Jiaozhou
54852	Shandong Laiyang
54855	Shandong Jimo
54861	Shandong Rushan
54904	Shandong Juancheng
54905	Shandong Yuncheng
54906	Shandong Heze
54907	Shandong Yutai
54909	Shandong Dingtao
54910	Shandong Liangshan
54911	Shandong Dongping
54912	Shandong Wenshang
54914	Shandong Juye
54916	Shandong Yanzhou
54919	Shandong Zoucheng
54920	Shandong Sishui
54922	Shandong Xintai
54923	Shandong Mengyin
54925	Shandong Pingyi
54927	Shandong Tengzhou
54929	Shandong Feixian
54932	Shandong Yishui
54938	Shandong Linyi
54939	Shandong Junan
54940	Shandong Wulian

54943	Shandong Huangdao
54945	Shandong Rizhao
58002	Shandong Caoxian
58003	Shandong Chengwu
58021	Shandong Xuechen
58022	Shandong Yicheng
58024	Shandong Zaozhuang
58030	Shandong Cangshan
58032	Shandong Linshu
53486	Shanxi Yanggao
53487	Shanxi Datong
53564	Shanxi Hequ
53565	Shanxi Pianguan
53576	Shanxi Shanyin
53659	Shanxi Linxian
53676	Shanxi Dingxiang
53753	Shanxi Liulin
53759	Shanxi Shilou
53760	Shanxi Fangshan
53763	Shanxi Gujiao
53764	Shanxi Lishi
53774	Shanxi Qingxu
53788	Shanxi Heshun
53852	Shanxi Yonghe
53859	Shanxi Jixian
53861	Shanxi Xiangfen
53863	Shanxi Jiexiu
53865	Shanxi Fenxi
53866	Shanxi Hongtong
53878	Shanxi Licheng
53953	Shanxi Xiangning
53954	Shanxi Jishan
53956	Shanxi Wanrong
53957	Shanxi Hejin
53959	Shanxi Yanhu
53965	Shanxi Jiangxian
53966	Shanxi Fushan
53970	Shanxi Qingshui
53973	Shanxi Gaoping
53981	Shanxi Lingchuan
57052	Shanxi Yongji
57053	Shanxi Ruicheng
53567	Shaanxi Fugu

53646	Shaanxi Yulin
53651	Shaanxi Shenmu
53658	Shaanxi Jiaxian
53725	Shaanxi Dingbian
53735	Shaanxi Jingbian
53738	Shaanxi Wuqi
53740	Shaanxi Hengshan
53748	Shaanxi Zichang
53754	Shaanxi Suide
53756	Shaanxi Wubao
53757	Shaanxi Qingjian
53832	Shaanxi Zhidan
53841	Shaanxi Ansai
53848	Shaanxi Ganquan
53850	Shaanxi Yanchuan
53854	Shaanxi Yanchang
53857	Shaanxi Yichuan
53931	Shaanxi Fuxian
53938	Shaanxi Xunyi
53941	Shaanxi Baishui
53944	Shaanxi Huangling
53946	Shaanxi Huanglong
53947	Shaanxi Tongchuan
53948	Shaanxi Pucheng
53949	Shaanxi Chengcheng
53950	Shaanxi Heyang
57003	Shaanxi Longxian
57016	Shaanxi Baoji
57021	Shaanxi Qianyang
57022	Shaanxi Linyou
57026	Shaanxi Fufeng
57027	Shaanxi Meixian
57029	Shaanxi Liquan
57030	Shaanxi Yongshou
57034	Shaanxi Wugong
57037	Shaanxi Yaoxian
57038	Shaanxi Xingping
57041	Shaanxi Sanyuan
57042	Shaanxi Fuping
57043	Shaanxi Dali
57045	Shaanxi Weinan
57046	Shaanxi Huashan
57057	Shaanxi Luonan

57106	Shaanxi Lueyang
57113	Shaanxi Fengxian
57119	Shaanxi Mianxian
57124	Shaanxi Liuba
57128	Shaanxi Chenggu
57134	Shaanxi Foping
57137	Shaanxi Ningshan
57140	Shaanxi Zhashui
57143	Shaanxi Shangxian
57153	Shaanxi Danfeng
57154	Shaanxi Shangnan
57155	Shaanxi Shanyang
57211	Shaanxi Ningqiang
57213	Shaanxi Nanzheng
57231	Shaanxi Ziyang
57233	Shaanxi Hanyin
57238	Shaanxi Zhenba
57242	Shaanxi Xunyang
57245	Shaanxi Ankang
57247	Shaanxi Langao
57248	Shaanxi Pingli
57254	Shaanxi Baihe
57343	Shaanxi Zhenping
58361	Shanghai Minhang
58362	Shanghai Baoshan
58460	Shanghai Jinshan
58463	Shanghai Fengxian
56038	Sichuan Shiqu
56079	Sichuan Ruoergai
56097	Sichuan Jiuzhaigou
56146	Sichuan Ganzi
56152	Sichuan Seda
56158	Sichuan Luhuo
56164	Sichuan Rangtang
56167	Sichuan Daofu
56168	Sichuan Jinchuan
56171	Sichuan Aba
56172	Sichuan Maerkang
56173	Sichuan Hongyuan
56178	Sichuan Xiaojin
56180	Sichuan Maoxian
56181	Sichuan Chongzhou
56182	Sichuan Songpan

56183	Sichuan Wenchuan
56184	Sichuan Lixian
56185	Sichuan Heishui
56186	Sichuan Mianzhu
56187	Sichuan Wenjiang
56188	Sichuan Dujiangyan
56189	Sichuan Pengzhou
56198	Sichuan Deyang
56247	Sichuan Batang
56251	Sichuan Xinlong
56257	Sichuan Litang
56263	Sichuan Danba
56267	Sichuan Yajiang
56273	Sichuan Baoxing
56279	Sichuan Lushan
56280	Sichuan Mingshan
56281	Sichuan Pujiang
56286	Sichuan Longquanyi
56289	Sichuan Pengshan
56296	Sichuan Jintang
56297	Sichuan Renshou
56298	Sichuan Ziyang
56357	Sichuan Daocheng
56371	Sichuan Luding
56373	Sichuan Yingjing
56374	Sichuan Kangding
56376	Sichuan Hanyuan
56378	Sichuan Shimian
56380	Sichuan Hongya
56382	Sichuan Jiajiang
56383	Sichuan Qingshen
56385	Sichuan Emeishan
56387	Sichuan Ebian
56389	Sichuan Qianwei
56390	Sichuan Jingyan
56393	Sichuan Zizhong
56395	Sichuan Weiyuan
56396	Sichuan Zigong
56399	Sichuan Fushun
56441	Sichuan Derong
56443	Sichuan Xiangcheng
56459	Sichuan Muli
56462	Sichuan Jiulong

56473	Sichuan Ganluo
56474	Sichuan Mianning
56475	Sichuan Yuexi
56478	Sichuan Xide
56479	Sichuan Chaojue
56480	Sichuan Mabian
56485	Sichuan Leibo
56487	Sichuan Meigu
56490	Sichuan Muchuan
56491	Sichuan Yibinxian
56493	Sichuan Nanxi
56494	Sichuan Pingshan
56496	Sichuan Xingwen
56499	Sichuan Gongxian
56565	Sichuan Yanyuan
56569	Sichuan Dechang
56571	Sichuan Xichang
56575	Sichuan Puge
56580	Sichuan Butuo
56584	Sichuan Jinyang
56593	Sichuan Changning
56665	Sichuan Yanbian
56666	Sichuan Panzhihua
56670	Sichuan Miyi
56671	Sichuan Huili
56675	Sichuan Huidong
57204	Sichuan Qingchuan
57206	Sichuan Guangyuan
57208	Sichuan Jiange
57216	Sichuan Nanjiang
57217	Sichuan Wangcang
57237	Sichuan Wanyuan
57306	Sichuan Langzhong
57309	Sichuan Xichong
57313	Sichuan Bazhong
57314	Sichuan Nanbu
57315	Sichuan Yilong
57318	Sichuan Yingshan
57320	Sichuan Tongjiang
57324	Sichuan Pingchang
57328	Sichuan Dachuan
57329	Sichuan Kaijiang
57401	Sichuan Shehong

57405	Sichuan Suining
57411	Sichuan Gaoping
57413	Sichuan Quxian
57415	Sichuan Guangan
57416	Sichuan Linshui
57417	Sichuan Wusheng
57420	Sichuan Dazhu
57503	Sichuan Dongxing
57507	Sichuan Longchang
57600	Sichuan Jiangan
57603	Sichuan Hejiang
57608	Sichuan Xuyong
54523	Tianjin Wuqing
54619	Tianjin Jinghai
54622	Tianjin Jinnan
54623	Tianjin Tanggu
54645	Tianjin Dagang
55655	Tibet Nielamu
51053	Xinjiang Habahe
51058	Xinjiang Akedala
51060	Xinjiang Buerjin
51068	Xinjiang Fuhai
51076	Xinjiang Aletai
51087	Xinjiang Fuyun
51133	Xinjiang Tacheng
51137	Xinjiang Yumin
51145	Xinjiang Emin
51156	Xinjiang Hebukesaier
51232	Xinjiang Alashankou
51238	Xinjiang Bole
51241	Xinjiang Touli
51243	Xinjiang Kelamayi
51334	Xinjiang Jinghe
51357	Xinjiang Shawan
51359	Xinjiang Manasi
51367	Xinjiang Hutubi
51368	Xinjiang Changji
51369	Xinjiang Miquan
51377	Xinjiang Fukang
51378	Xinjiang Jimusaer
51468	Xinjiang Tianshandaxigou
51469	Xinjiang Urumqi Pastoral Test Station
51470	Xinjiang Tianchi

51477	Xinjiang Dabancheng
51482	Xinjiang Mulei
51526	Xinjiang Kumishi
51542	Xinjiang Bayinbuluke
51567	Xinjiang Yanqi
51571	Xinjiang Tuokexun
51572	Xinjiang Tulufandongkan
51573	Xinjiang Tulufan
51627	Xinjiang Wushi
51628	Xinjiang Akesu
51636	Xinjiang Xinhe
51639	Xinjiang Shaya
51656	Xinjiang Kuerle
51704	Xinjiang Atushi
51705	Xinjiang Wuqia
51707	Xinjiang Jiashi
51709	Xinjiang Kashi
51717	Xinjiang Yuepuhu
51720	Xinjiang Kepin
51722	Xinjiang Awati
51730	Xinjiang Alaer
51747	Xinjiang Tazhong
51765	Xinjiang Tieqianlike
51802	Xinjiang Yengjisha
51810	Xinjiang Maigaiti
51811	Xinjiang Shashe
51814	Xinjiang Yecheng
51815	Xinjiang Zepu
51818	Xinjiang Pishan
51826	Xinjiang Cele
51827	Xinjiang Moyu
51828	Xinjiang Hetan
51829	Xinjiang Luopu
51839	Xinjiang Minfeng
51855	Xinjiang Qiemuo
51931	Xinjiang Yutian
52101	Xinjiang Balikun
52112	Xinjiang Naomaohu
52118	Xinjiang Yiwu
52203	Xinjiang Hami
52313	Xinjiang Hongliuhe
56444	Yunnan Deqin
56483	Yunnan Suijiang

56497	Yunnan Yanjin
56533	Yunnan Gongshan
56543	Yunnan Xianggelila
56548	Yunnan Weixi
56567	Yunnan Ninglang
56582	Yunnan Daguan
56585	Yunnan Ludian
56594	Yunnan Yiliang
56595	Yunnan Zhenxiong
56596	Yunnan Weixin
56641	Yunnan Fugong
56643	Yunnan Liuku
56645	Yunnan Lanping
56646	Yunnan Jianchuan
56649	Yunnan Eryuan
56651	Yunnan Lijiang
56652	Yunnan Yongsheng
56654	Yunnan Heqing
56664	Yunnan Huaping
56669	Yunnan Yongren
56673	Yunnan Qiaojia
56688	Yunnan Dongchuan
56697	Yunnan Xuanwei
56739	Yunnan Tengchong
56742	Yunnan Yunlong
56745	Yunnan Yangbi
56746	Yunnan Yongping
56748	Yunnan Baoshan
56751	Yunnan Dali
56752	Yunnan Binchuan
56755	Yunnan Midu
56757	Yunnan Weishan
56764	Yunnan Yaoan
56766	Yunnan Mouding
56767	Yunnan Nanhua
56772	Yunnan Fumin
56774	Yunnan Wuding
56777	Yunnan Lufeng
56778	Yunnan Kunming
56782	Yunnan Malong
56783	Yunnan Qujing
56785	Yunnan Songming
56790	Yunnan Fuyuan

56835	Yunnan Longchuan
56836	Yunnan Yingjiang
56839	Yunnan Zhenkang
56840	Yunnan Lianghe
56841	Yunnan Longling
56842	Yunnan Shidian
56843	Yunnan Changning
56846	Yunnan Fengqing
56849	Yunnan Yongde
56854	Yunnan Yunxian
56856	Yunnan Jingdong
56862	Yunnan Shuangbai
56863	Yunnan Anning
56867	Yunnan Zhenyuan
56869	Yunnan Xinping
56870	Yunnan Yimen
56871	Yunnan Jinning
56873	Yunnan Chengjiang
56875	Yunnan Yuxi
56879	Yunnan Huaning
56880	Yunnan Yiliang
56881	Yunnan Shilin
56883	Yunnan Shizong
56885	Yunnan Mile
56886	Yunnan Luxi
56889	Yunnan Qiubei
56891	Yunnan Luoping
56898	Yunnan Eshan
56944	Yunnan Cangyuan
56946	Yunnan Gengma
56948	Yunnan Ximeng
56949	Yunnan Menglian
56950	Yunnan Shuangjiang
56951	Yunnan Lincang
56952	Yunnan Jinggu
56954	Yunnan Lancang
56958	Yunnan Menghai
56962	Yunnan Mojiang
56964	Yunnan Simao
56966	Yunnan Yuanjiang
56969	Yunnan Mengla
56970	Yunnan Shiping
56976	Yunnan Yuanyang

56977	Yunnan Jiangcheng
56978	Yunnan Luchun
56982	Yunnan Kaiyuan
56984	Yunnan Gejiu
56987	Yunnan Jinping
56991	Yunnan Yanshan
56992	Yunnan Xichou
56994	Yunnan Wenshan
59007	Yunnan Guangnan
59205	Yunnan Funing
58443	Zhejiang Changxing
58446	Zhejiang Anji
58448	Zhejiang Linan
58449	Zhejiang Fuyang
58450	Zhejiang Huzhou
58451	Zhejiang Jiashan
58452	Zhejiang Jiaxing
58453	Zhejiang Shaoxing
58454	Zhejiang Deqing
58457	Zhejiang Hangzhou
58467	Zhejiang Cixi
58472	Zhejiang Shengsi
58484	Zhejiang Daishan
58537	Zhejiang Kaihua
58542	Zhejiang Tonglu
58544	Zhejiang Jiande
58546	Zhejiang Pujiang
58547	Zhejiang Longyou
58549	Zhejiang Jinhua
58550	Zhejiang Zhuji
58555	Zhejiang Xinchang
58556	Zhejiang Shengzhou
58558	Zhejiang Dongyang
58559	Zhejiang Tiantai
58560	Zhejiang Panan
58561	Zhejiang Zhenhai
58562	Zhejiang Yinzhou
58565	Zhejiang Fenghua
58566	Zhejiang Xiangshan
58567	Zhejiang Ninghai
58568	Zhejiang Sanmen
58569	Zhejiang Shipu
58570	Zhejiang Putuo

58631	Zhejiang Changshan
58632	Zhejiang Jiangshan
58642	Zhejiang Wuyi
58643	Zhejiang Yongkang
58644	Zhejiang Suichang
58646	Zhejiang Lishui
58647	Zhejiang Longquan
58652	Zhejiang Xianju
58654	Zhejiang Jinyun
58656	Zhejiang Leqing
58657	Zhejiang Qingtian
58658	Zhejiang Yongjia
58660	Zhejiang Linhai
58664	Zhejiang Wenling
58665	Zhejiang Hongjia
58666	Zhejiang Dachen
58742	Zhejiang Yunhe
58746	Zhejiang Taishun
58750	Zhejiang Wencheng
58751	Zhejiang Pingyang
58648	Zhejiang Jingning
57333	Chongqing Chengkou
57338	Chongqing Kaixian
57339	Chongqing Yunyang
57345	Chongqing Wuxi
57349	Chongqing Wushan
57409	Chongqing Tongnan
57425	Chongqing Dianjiang
57432	Chongqing Wanzhou
57437	Chongqing Zhongxian
57438	Chongqing Shizhu
57502	Chongqing Dazu
57505	Chongqing Rongchang
57506	Chongqing Yongchuan
57509	Chongqing Wansheng
57510	Chongqing Tongliang
57511	Chongqing Beibei
57513	Chongqing Yubei
57514	Chongqing Bishan
57517	Chongqing Jiangjin
57518	Chongqing Banan
57519	Chongqing Nanchuan
57520	Chongqing Changshou

57523	Chongqing Fengdu
57525	Chongqing Wulong
57536	Chongqing Qianjiang
57537	Chongqing Pengshui
57612	Chongqing Qijiang

Table S5. 1742 monitoring stations whose PM2.5 emissions are from residential sector

58102	Anhui Bozhou
58220	Anhui Changfeng
58326	Anhui Chaohu
58236	Anhui Chuzhou
58015	Anhui Dangshan
58225	Anhui Dingyuan
58323	Anhui Feidong
58320	Anhui Feixi
58212	Anhui Fengtai
58222	Anhui Fengyang
58202	Anhui Funan
58203	Anhui Fuyang
58128	Anhui Guzhen
58441	Anhui Guangde
58330	Anhui Hanshan
58127	Anhui Huaiyuan
58437	Anhui Huangshan
58214	Anhui Huoqiu
58314	Anhui Huoshan
58108	Anhui Jieshou
58306	Anhui Jinzhai
58432	Anhui Jingxian
58435	Anhui Jingde
58234	Anhui Laian
58442	Anhui Langxi
58117	Anhui Leysin
58125	Anhui Lingbi
58311	Anhui Luan
58327	Anhui Lujiang
58336	Anhui Maanshan
58118	Anhui Mengcheng
58223	Anhui Mingguang
58431	Anhui Nanling
58436	Anhui Ningguo
58520	Anhui Qimen
58316	Anhui Shucheng
58126	Anhui Sixian
58417	Anhui Susong
58122	Anhui Suzhou
58113	Anhui Suixi

58109	Anhui Taihe
58240	Anhui Tianchang
58112	Anhui Tianzhushan
58319	Anhui Tongcheng
58429	Anhui Tongling
58531	Anhui Tunxi
58114	Anhui Woyang
58329	Anhui Wuwei
58334	Anhui Wuhu
58338	Anhui Wuhuxian
58129	Anhui Wuhe
58530	Anhui Shexian
58016	Anhui Xiaoxian
58433	Anhui Xuancheng
58523	Anhui Yixian
58210	Anhui Yingshang
58317	Anhui Yuexi
54511	Beijing
54499	Beijing Changping
54399	Beijing Haidian
54416	Beijing Miyun
54421	Beijing Miyunshangdianzi
54424	Beijing Pinggu
54398	Beijing Shunyi
54431	Beijing Tongzhou
54597	Beijing Xiayunling
54406	Beijing Yanqing
54501	Beijing Zhaitang
58929	Fujian Anxi
58941	Fujian Changle
59122	Fujian Changtai
58911	Fujian Changting
59133	Fujian Chongwu
58923	Fujian Datian
58748	Fujian Fuan
58754	Fujian Fuding
58942	Fujian Fuqing
58836	Fujian Gutian
58724	Fujian Guangze
58928	Fujian Huaan
58822	Fujian Jianning
58734	Fujian Jianyang
58931	Fujian Jiuxianshan

58848	Fujian Lianjiang
58844	Fujian Minhou
58839	Fujian Shuqing
58824	Fujian Mingxi
59131	Fujian Nanan
59124	Fujian Nanjing
58846	Fujian Ningde
58818	Fujian Ninghuai
59125	Fujian Pinghe
58944	Fujian Pingtan
58946	Fujian Putian
58731	Fujian Pucheng
58828	Fujian Sanming
58725	Fujian Shaowu
58744	Fujian Shouning
58823	Fujian Shunchang
58735	Fujian Songxi
58820	Fujian Taining
59130	Fujian Tongan
58917	Fujian Wuping
58730	Fujian Wuyishan
58843	Fujian Xiapu
58938	Fujian Xiuyu
59113	Fujian Yongding
58932	Fujian Yongtai
58837	Fujian Youxi
59322	Fujian Yunxiao
58926	Fujian Zhangping
59129	Fujian Zhangpu
59320	Fujian Zhaoan
58749	Fujian Zherong
58736	Fujian Zhenghe
58747	Fujian Zhouning
52995	Gansu Anding
52896	Gansu Baiyin
57102	Gansu Chengxian
56095	Gansu Dangchang
56084	Gansu Dibu
52981	Gansu Dongxiang
57001	Gansu Gangu
52884	Gansu Gaolan
52546	Gansu Gaitai
52784	Gansu Gulang

52982	Gansu Guanghe
53934	Gansu Huishui
56080	Gansu Hezuo
52985	Gansu Hezheng
53930	Gansu Huachi
52996	Gansu Huajialing
53927	Gansu Huating
53821	Gansu Huanxian
57110	Gansu Huixian
52993	Gansu Huining
53926	Gansu Jingchuan
52797	Gansu Jingtai
52895	Gansu Jingyuan
53906	Gansu Jingning
52533	Gansu Jiuquan
52988	Gansu Kangle
57105	Gansu Kangxian
53915	Gansu Kongtong
57007	Gansu Lixian
57111	Gansu Liangdang
56081	Gansu Lintan
52986	Gansu Lintao
52557	Gansu Linze
53924	Gansu Lingtai
56092	Gansu Longxi
56071	Gansu Luqu
52323	Gansu Mazongshan
56074	Gansu Maqu
57014	Gansu Maiji
52656	Gansu Minle
52681	Gansu Minqin
56093	Gansu Minxian
57002	Gansu Qinan
57011	Gansu Qingshui
53829	Gansu Qingcheng
52515	Gansu Subei
52643	Gansu Sunan
57006	Gansu Tianshui
52881	Gansu Tianzhu
52998	Gansu Weiyuan
56192	Gansu Wenxian
52787	Gansu Wushaoling
56096	Gansu Wudu

57004	Gansu Wushan
52679	Gansu Wuwei
53923	Gansu Xifeng
52978	Gansu Xiahe
52674	Gansu Yongchang
52885	Gansu Yongdeng
52980	Gansu Yongjing
52983	Gansu Yuzhong
57012	Gansu Zhangjiachuan
56091	Gansu Zhangxian
53925	Gansu Zhenyuan
53935	Gansu Zhengning
56094	Gansu Zhouqu
53917	Gansu Zhuanglang
56082	Gansu Zhuoni
59297	Guangdong Boluo
59312	Guangdong Chaozhou
59285	Guangdong Conghua
59116	Guangdong Daipu
59289	Guangdong Dongguan
59487	Guangdong Doumen
59477	Guangdong Enping
59481	Guangdong Panyu
59310	Guangdong Fengshun
59087	Guangdong Fogang
59653	Guangdong Gaozhou
59271	Guangdong Guangning
59287	Guangdong Guangzhou
59500	Guangdong Haifeng
59099	Guangdong Heping
59293	Guangdong Heyuan
59473	Guangdong Heshan
59284	Guangdong Huadu
59655	Guangdong Huazhou
59270	Guangdong Huaiji
59492	Guangdong Huidong
59114	Guangdong Jiaoling
59306	Guangdong Jiexi
59475	Guangdong Kaiping
57988	Guangdong Lechang
59750	Guangdong Leizhou
59096	Guangdong Lianping
59074	Guangdong Lianshan

59654	Guangdong Lianjiang
59107	Guangdong Longchuan
59290	Guangdong Longmen
59502	Guangdong Lufeng
59462	Guangdong Luoding
59659	Guangdong Maoming
59117	Guangdong Meixian
57996	Guangdong Nanxiong
59106	Guangdong Pingyuan
59314	Guangdong Puning
59280	Guangdong Qingyuan
59313	Guangdong Raoping
57989	Guangdong Renhua
59081	Guangdong Ruyuan
59279	Guangdong Sanshui
59316	Guangdong Shantou
59082	Guangdong Shaoguan
59493	Guangdong Shenzhen
59090	Guangdong Shixing
59276	Guangdong Sihui
59650	Guangdong Suixi
59094	Guangdong Wengyuan
59656	Guangdong Wuchuan
59303	Guangdong Wuhua
59476	Guangdong Xinhui
59470	Guangdong Xinxing
59456	Guangdong Xinyi
59109	Guangdong Xingning
59754	Guangdong Xuwen
59469	Guangdong Yangchun
59075	Guangdong Yangshan
59088	Guangdong Yingde
59268	Guangdong Yunan
59471	Guangdong Yunfu
59488	Guangdong Zhuhai
59304	Guangdong Zijin
59027	Guangxi Bama
59211	Guangxi Baise
59451	Guangxi Beiliu
59238	Guangxi Binyang
59449	Guangxi Bobai
59266	Guangxi Cangwu
59454	Guangxi Cenxi

59421	Guangxi Daxin
59215	Guangxi Debao
59037	Guangxi Duan
59021	Guangxi Fengshan
57964	Guangxi Guanyang
59640	Guangxi Hepu
59023	Guangxi Hechi
59065	Guangxi Hezhou
59441	Guangxi Hengxian
59057	Guangxi Jinxiu
59218	Guangxi Jingxi
59242	Guangxi Laibin
59012	Guangxi Leye
59055	Guangxi Lipu
57954	Guangxi Lingui
59446	Guangxi Lingshan
59015	Guangxi Lingyun
59041	Guangxi Liucheng
57942	Guangxi Longsheng
59229	Guangxi Longan
59457	Guangxi Luchuan
59045	Guangxi Luzhai
59230	Guangxi Mashan
59058	Guangxi Mengshan
59209	Guangxi Napo
59431	Guangxi Nanning
59053	Guangxi Pingle
59255	Guangxi Pingnan
59419	Guangxi Pingxiang
59448	Guangxi Pubei
59632	Guangxi Qinzhou
57960	Guangxi Quanzhou
59452	Guangxi Rongxian
57948	Guangxi Rongshui
57941	Guangxi Sanjiang
59235	Guangxi Shanglin
59429	Guangxi Shangsi
59256	Guangxi Tengxian
59227	Guangxi Tiandeng
57927	Guangxi Tiane
59224	Guangxi Tiandong
59017	Guangxi Tianlin
59265	Guangxi Wuzhou

59237	Guangxi Wuming
59246	Guangxi Wuxuan
59004	Guangxi Xilin
59241	Guangxi Xiangzhou
59038	Guangxi Xincheng
57955	Guangxi Xingan
59034	Guangxi Yizhou
59435	Guangxi Yongning
57949	Guangxi Yongfu
57859	Guangxi Ziyuan
57806	Guizhou Anshun
57707	Guizhou Bijie
57909	Guizhou Ceheng
57735	Guizhou Cengong
57936	Guizhou Congjiang
57708	Guizhou Dafang
57623	Guizhou Daozhen
57637	Guizhou Dejiang
57827	Guizhou Duyun
57922	Guizhou Dushan
57723	Guizhou Fenggang
57821	Guizhou Fuquan
57903	Guizhou Guanling
57824	Guizhou Guiding
56598	Guizhou Hezhang
57822	Guizhou Huangping
57912	Guizhou Huishui
57835	Guizhou Jianhe
57736	Guizhou Jiangkou
57714	Guizhou Jinsha
57844	Guizhou Jinping
57719	Guizhou Kaiyang
57825	Guizhou Kaili
57837	Guizhou Leishan
57839	Guizhou Liping
57926	Guizhou Libo
57807	Guizhou Liuzhi
57913	Guizhou Longli
57916	Guizhou Luodian
57722	Guizhou Meitan
57800	Guizhou Nayong
56793	Guizhou Panxian
57814	Guizhou Pingba

57921	Guizhou Pingtang
56792	Guizhou Puan
57803	Guizhou Xianxi
57900	Guizhou Qinglong
57932	Guizhou Rongjiang
57923	Guizhou Sandu
57832	Guizhou Sansui
57737	Guizhou Shibing
57734	Guizhou Shiqian
57731	Guizhou Sinan
57647	Guizhou Songtao
57834	Guizhou Taijiang
57840	Guizhou Tianzhu
57606	Guizhou Tongzi
57742	Guizhou Wanshan
57906	Guizhou Wangmo
56691	Guizhou Weining
57728	Guizhou Wengan
57634	Guizhou Wuchuan
57718	Guizhou Xifeng
57907	Guizhou Xingyi
57811	Guizhou Xiuwen
57636	Guizhou Yanhe
57729	Guizhou Yuqing
57739	Guizhou Yuping
57905	Guizhou Zhenfeng
57625	Guizhou Zhengan
57805	Guizhou Zhijin
57910	Guizhou Ziyun
57717	Guizhou Zunyi
59848	Hainan Baisha
59945	Hainan Baoting
59847	Hainan Changjiang
59843	Hainan Chengmai
59845	Hainan Danzhou
59851	Hainan Dingan
59758	Hainan Haikou
59842	Hainan Lingao
59855	Hainan Qonghai
59854	Hainan Tunchang
59951	Hainan Wanning
59856	Hainan Wenchang
54605	Hebei Anxin

53785	Hebei Baixiang
54618	Hebei Botou
54616	Hebei Cangzhou
54535	Hebei Caofeidian
54540	Hebei Changli
54423	Hebei Chengde
54430	Hebei Chengdexian
54404	Hebei Chicheng
54304	Hebei Chongli
54510	Hebei Dachang
54613	Hebei Dacheng
54804	Hebei Daming
53696	Hebei Dingzhou
54713	Hebei Dongguang
54308	Hebei Fengning
53894	Hebei Fengfeng
54541	Hebei Funing
54710	Hebei Fucheng
53690	Hebei Fuping
54506	Hebei Gaobeidian
54603	Hebei Goyang
54301	Hebei Guyuan
54512	Hebei Guan
54707	Hebei Gucheng
54809	Hebei Guantao
54631	Hebei Guangzong
54628	Hebei Haixing
53892	Hebei Handan
54614	Hebei Hejian
54702	Hebei Hengshui
53491	Hebei Huaian
54405	Hebei Huailai
54624	Hebei Huanghua
54640	Hebei Jize
53689	Hebei Jinzhou
54711	Hebei Jingxian
53799	Hebei Julu
53392	Hebei Kangbao
54432	Hebei Kuancheng
53599	Hebei Laiyuan
54539	Hebei Leting
54801	Hebei Linxi
53773	Hebei Linzhang

53680	Hebei Lingshou
54318	Hebei Longhua
53794	Hebei Longyao
54438	Hebei Lulong
53789	Hebei Luancheng
54437	Hebei Luannan
54420	Hebei Luanping
53796	Hebei Ningjin
54319	Hebei Pingquan
54439	Hebei Qianan
54434	Hebei Qianxi
54449	Hebei Qinhuangdao
54436	Hebei Qinglong
54615	Hebei Qingxian
54706	Hebei Qinghe
53682	Hebei Quyang
53893	Hebei Quzhou
54606	Hebei Raoyang
54610	Hebei Renqiu
54503	Hebei Rongcheng
54520	Hebei Sanhe
53781	Hebei Shahe
53397	Hebei Shangyi
53886	Hebei Shexian
54608	Hebei Shenzhou
53596	Hebei Shunping
54534	Hebei Tangshan
53692	Hebei Tangxian
53499	Hebei Wanquan
54800	Hebei-Weixian
54311	Hebei Weichang
53593	Hebei Weixian
53896	Hebei Weixian
54612	Hebei Wenan
53699	Hebei Wuji
53890	Hebei Wuan
54700	Hebei Wuqiang
54703	Hebei Wuyi
54521	Hebei Xianghe
54701	Hebei Xinji
54633	Hebei Xinhe
54425	Hebei Xinglong
53688	Hebei Xingtang

54601	Hebei Xushui
53498	Hebei Xuanhua
53492	Hebei Yangyuan
54519	Hebei Yongqing
54522	Hebei Yutian
53795	Hebei Zanhuang
53399	Hebei Zhangbei
54401	Hebei Zhangjiakou
54408	Hebei Zhulu
54502	Hebei Zhuozhou
54429	Hebei Zunhua
57087	Henan Changge
53998	Henan Changheng
58100	Henan Dancheng
57082	Henan Dengfeng
54903	Henan Fanxian
57179	Henan Fangcheng
53983	Henan Fengqiu
57080	Henan Gongyi
58208	Henan Gushi
57299	Henan Guangshan
57192	Henan Huaiyang
58207	Henan Huangchuan
53985	Henan Huixian
57390	Henan Jigongshan
53978	Henan Jiyuan
57180	Henan Jiaxian
53982	Henan Jiaozuo
53992	Henan Junxian
57091	Henan Kaifeng
57093	Henan Lankao
53889	Henan Linzhou
57183	Henan Linying
57056	Henan Lingbao
57173	Henan Lushan
57066	Henan Luoning
57186	Henan Luohe
57071	Henan Mengjin
57072	Henan Mengzhou
57281	Henan Biyang
58004	Henan Minquan
53993	Henan Neihuang
57169	Henan Neixiang

57178	Henan Nanyang
57176	Henan Nanzhao
57292	Henan Pingyu
54900	Henan Puyang
53974	Henan Qixian
57096	Henan Qixian
53972	Henan Qinyang
54902	Henan Qingfeng
57197	Henan Runan
57078	Henan Ruyang
57075	Henan Ruzhou
57051	Henan Sanmenxia
58301	Henan Shangcheng
58005	Henan Shangqiu
57187	Henan Sheqi
57063	Henan Mianchi
57189	Henan Suiping
54817	Henan Taiqian
57099	Henan Taikang
53991	Henan Tangyin
57273	Henan Tanghe
57285	Henan Tongbai
57079	Henan Wenxian
57177	Henan Wugang
57185	Henan Wuyang
57193	Henan Xihua
57188	Henan Xiping
57156	Henan Xixia
57296	Henan Xixian
58017	Henan Xiayi
57182	Henan Xiangcheng
57070	Henan Xinan
57293	Henan Xincai
57396	Henan Xixian
53986	Henan Xinxiang
57271	Henan Xinye
57297	Henan Xinyang
53984	Henan Xiuwu
57089	Henan Xuchang
57095	Henan Yanling
57184	Henan Yexian
57074	Henan Yichuan
57081	Henan Xingyang

58006	Henan Yucheng
58007	Henan Echeng
57175	Henan Zhenping
57295	Henan Zhengyang
57090	Henan Zhongmou
57290	Henan Zhumadian
50958	Heilongjiang Acheng
50468	Heilongjiang Aihui
50854	Heilongjiang Anda
50867	Heilongjiang Bayan
50755	Heilongjiang Baiquan
50888	Heilongjiang Baoqing
50656	Heilongjiang Beian
50137	Heilongjiang Beijicun
50853	Heilongjiang Beilin
50960	Heilongjiang Binxian
50973	Heilongjiang Boli
50850	Heilongjiang Daqing
50842	Heilongjiang Dumeng
50964	Heilongjiang Zhengfang
50779	Heilongjiang Fuyuan
50788	Heilongjiang Fujin
50742	Heilongjiang Fuyu
50741	Heilongjiang Gannan
50953	Heilongjiang Haerbin
54092	Heilongjiang Hailin
50756	Heilongjiang Hailun
50775	Heilongjiang Hegang
50956	Heilongjiang Hulan
50353	Heilongjiang Huma
50247	Heilongjiang Huzhong
50983	Heilongjiang Hulin
50878	Heilongjiang Huachuan
50879	Heilongjiang Huanan
50987	Heilongjiang Jidong
50978	Heilongjiang Jixi
50442	Heilongjiang Jiagedaqi
50873	Heilongjiang Jiamusi
50673	Heilongjiang Jiayin
50659	Heilongjiang Kedong
50658	Heilongjiang Keshan
50859	Heilongjiang Lansi
50749	Heilongjiang Lindian

50979	Heilongjiang Linkou
50739	Heilongjiang Longjiang
50776	Heilongjiang Luobei
50985	Heilongjiang Mishan
50758	Heilongjiang Minshui
50136	Heilongjiang Mohe
54094	Heilongjiang Mudanjiang
50962	Heilongjiang Mulan
54093	Heilongjiang Muling
50646	Heilongjiang Nehe
50557	Heilongjiang Nenjiang
54098	Heilongjiang Ningan
50971	Heilongjiang Qitaihe
50745	Heilongjiang Qiqihaer
50851	Heilongjiang Qinggang
50861	Heilongjiang Qingan
50892	Heilongjiang Raohe
50968	Heilongjiang Shangzhi
50884	Heilongjiang Shuangyashan
50787	Heilongjiang Suibin
54096	Heilongjiang Suifenhe
50767	Heilongjiang Suileng
50564	Heilongjiang Sunwu
50246	Heilongjiang Tahe
50844	Heilongjiang Tailai
50871	Heilongjiang Tangyuan
50862	Heilongjiang Tieli
50963	Heilongjiang Tonghe
50778	Heilongjiang Tongjiang
50852	Heilongjiang Wangkui
50674	Heilongjiang Wuyiling
54080	Heilongjiang Wuchang
50655	Heilongjiang Wudalianchi
50772	Heilongjiang Wuying
50349	Heilongjiang Xinlin
50566	Heilongjiang Xunke
50965	Heilongjiang Yanshou
50774	Heilongjiang Yichun
50750	Heilongjiang Yian
50877	Heilongjiang Yilang
50858	Heilongjiang Zhaodong
50950	Heilongjiang Zhaozhou
57388	Hubei Anlu

57355	Hubei Padang
57361	Hubei Baokang
57489	Hubei Caidian
57582	Hubei Chibi
57586	Hubei Chongyang
57260	Hubei Danjiangkou
57496	Hubei Ezhou
57447	Hubei Enshi
57259	Hubei Fangxian
57477	Hubei Gongan
57268	Hubei Gucheng
57486	Hubei Hanchuan
57543	Hubei Hefeng
57398	Hubei Hongan
57581	Hubei Honghu
58409	Hubei Huangmei
57583	Hubei Jiayu
57573	Hubei Jianli
57445	Hubei Jianshi
57387	Hubei Jingshan
57377	Hubei Jingmen
57439	Hubei Lichuan
58401	Hubei Luotian
57399	Hubei Macheng
57363	Hubei Nanzhang
58408	Hubei Qichun
57475	Hubei Qianjiang
57484	Hubei Shayang
57362	Hubei Shennongjia
57256	Hubei Shiyan
57571	Hubei Shishou
57469	Hubei Songzi
57381	Hubei Suizhou
57483	Hubei Tianmen
57589	Hubei Tongcheng
57495	Hubei Tuanfeng
57458	Hubei Wufeng
57494	Hubei Wuhan
58501	Hubei Wuxue
57485	Hubei Xiantao
57540	Hubei Xianfeng
57590	Hubei Xianning
57278	Hubei Xiangyang

57386	Hubei Xiaochang
57482	Hubei Xiaogan
57492	Hubei Xinzhou
57359	Hubei Xingshan
57541	Hubei Xuanen
58500	Hubei Yangxin
57453	Hubei Yiling
57370	Hubei Yicheng
57481	Hubei Yingcheng
58402	Hubei Yingshan
57251	Hubei Yunxi
57253	Hubei Yunxian
57279	Hubei Zaoyang
57378	Hubei Zhongxiang
57257	Hubei Zhushan
57249	Hubei Zhuxi
57669	Hunan Anhua
57881	Hunan Anren
57642	Hunan Baojing
57882	Hunan Chaling
57687	Hunan Changsha
57662	Hunan Changde
57874	Hunan Changning
57972	Hunan Chenzhou
57965	Hunan Daoxian
57867	Hunan Dongan
57740	Hunan Fenghuang
57646	Hunan Guzhang
57889	Hunan Guangxi
57973	Hunan Guiyang
57663	Hunan Hanshou
57875	Hunan Hengnan
57777	Hunan Hengshan
57872	Hunan Hengyang
57871	Hunan Hengyangxian
57754	Hunan Hongjiang
57575	Hunan Huarong
57649	Hunan Jishou
57974	Hunan Jiahe
59063	Hunan Jianghua
57969	Hunan Jiangyong
57975	Hunan Lanshan
57760	Hunan Lengshuijiang

57865	Hunan Lengshuitan
57565	Hunan Lixian
57781	Hunan Liling
57978	Hunan Linwu
57688	Hunan Liuyang
57544	Hunan Longshan
57763	Hunan Loudi
57657	Hunan Luxi
57680	Hunan Milo
57574	Hunan Nanxian
57776	Hunan Nanyue
57678	Hunan Ningxiang
57966	Hunan Ningyuan
57682	Hunan Pingjiang
57870	Hunan Qidong
57868	Hunan Qiyang
57985	Hunan Rucheng
57554	Hunan Sangzhi
57771	Hunan Shaoshan
57774	Hunan Shuangfeng
57962	Hunan Shuangpai
57666	Hunan Taojiang
57661	Hunan Taoyuan
57845	Hunan Tongtao
57772	Hunan Xiangxiang
57673	Hunan Xiangyin
57761	Hunan Xinhua
57744	Hunan Xinhuang
57971	Hunan Xintian
57752	Hunan Xupu
57976	Hunan Yizhang
57643	Hunan Yongshun
57887	Hunan Yongxing
57866	Hunan Yongzhou
57779	Hunan Youxian
57671	Hunan Yuanjiang
57655	Hunan Yuanling
57584	Hunan Yueyang
57558	Hunan Zhangjiajie
57745	Hunan Zhijiang
57780	Hunan Zhuzhou
57981	Hunan Zixing
54187	Jilin Antu

50936	Jilin Baicheng
54371	Jilin Baishan
54386	Jilin Changbai
54161	Jilin Changchun
54049	Jilin Changling
50945	Jilin Daan
54065	Jilin Dehui
54261	Jilin Tongfeng
54284	Jilin Donggang
54186	Jilin Dunhua
54285	Jilin Erdao
54063	Jilin Fuyu
54155	Jilin Gujiazi
54286	Jilin Helong
54273	Jilin Huadian
54291	Jilin Huichun
54274	Jilin Huinan
54172	Jilin Jilin Suburb
54377	Jilin Jian
54279	Jilin Jiangyuan
54181	Jilin Jiaohe
54069	Jilin Jiutai
54154	Jilin Lishu
54260	Jilin Liaoyuan
54374	Jilin Linjiang
54267	Jilin Liuhe
54290	Jilin Longjing
54192	Jilin Luozigou
54064	Jilin Nongan
54263	Jilin Panshi
50949	Jilin Qianguo
50948	Jilin Qianan
54076	Jilin Shulan
54142	Jilin Shuangliao
54165	Jilin Shuangyang
50946	Jilin Songyuan
50939	Jilin Taonan
54363	Jilin Tonghua
54362	Jilin Tonghuaxian
54041	Jilin Tongyu
54195	Jilin Wangqing
54169	Jilin Yantongshan
54292	Jilin Yanji

54164	Jilin Yitong
54171	Jilin Yongji
54072	Jilin Yushu
50940	Jilin Zhenlai
58148	Jiangsu Baoying
58352	Jiangsu Changshu
58158	Jiangsu Dafeng
58341	Jiangsu Danyang
58036	Jiangsu Donghai
58251	Jiangsu Dongtai
58012	Jiangsu Fengxian
58143	Jiangsu Funing
58040	Jiangsu Ganyu
58339	Jiangsu Gaochun
58241	Jiangsu Gaoyou
58047	Jiangsu Guanyun
58254	Jiangsu Haian
58360	Jiangsu Haimen
58139	Jiangsu Hongze
58141	Jiangsu Huaian
58146	Jiangsu Jianhu
58250	Jiangsu Jiangyan
58147	Jiangsu Jinhu
58342	Jiangsu Jintan
58257	Jiangsu Jingjiang
58344	Jiangsu Jurong
58356	Jiangsu Kunshan
58340	Jiangsu Lishui
58044	Jiangsu Lianyungang
58140	Jiangsu Lianshui
58235	Jiangsu Liuhe
58265	Jiangsu Lvsi
58238	Jiangsu Nanjing
58259	Jiangsu Nantong
58013	Jiangsu Peixian
58026	Jiangsu Pizhou
58237	Jiangsu Pukou
58269	Jiangsu Qidong
58264	Jiangsu Rudong
58255	Jiangsu Rugao
58038	Jiangsu Shuyang
58135	Jiangsu Sihong
58132	Jiangsu Siyang

58349	Jiangsu Suzhou
58131	Jiangsu Suyu
58130	Jiangsu Suining
58377	Jiangsu Taicang
58249	Jiangsu Taixing
58246	Jiangsu Taizhou
58354	Jiangsu Wuxi
58041	Jiangsu Xiliandao
58045	Jiangsu Xiangshui
58243	Jiangsu Xinghua
58138	Jiangsu Xuyi
58027	Jiangsu Xuzhou
58154	Jiangsu Yancheng
58247	Jiangsu Yangzhong
58242	Jiangsu Yizheng
58346	Jiangsu Yixing
58353	Jiangsu Zhangjiagang
57798	Jiangxi Anfu
58602	Jiangxi Anyi
58907	Jiangxi Anyuan
58519	Jiangxi Poyang
58710	Jiangxi Chongren
57990	Jiangxi Chongyi
58508	Jiangxi Dean
58622	Jiangxi Dexing
59093	Jiangxi Dingnan
58618	Jiangxi Dongxiang
58517	Jiangxi Duchang
57792	Jiangxi Fenyi
58601	Jiangxi Fengxin
57993	Jiangxi Ganxian
58605	Jiangxi Gaoan
58813	Jiangxi Guangchang
58626	Jiangxi Guixi
58510	Jiangxi Hukou
58906	Jiangxi Huichang
57799	Jiangxi Jianxian
58712	Jiangxi Jinxi
58614	Jiangxi Jinxian
58527	Jiangxi Jingdezhen
58600	Jiangxi Jingan
58502	Jiangxi Jiujiang
58706	Jiangxi Lean

58719	Jiangxi Lichuan
57789	Jiangxi Lianhua
58619	Jiangxi Linchuan
59092	Jiangxi Longnan
58506	Jiangxi Lushan
58606	Jiangxi Nanchang
58715	Jiangxi Nancheng
58718	Jiangxi Nanfeng
57992	Jiangxi Nankang
58806	Jiangxi Ningdu
58512	Jiangxi Pengze
57786	Jiangxi Pingxiang
58629	Jiangxi Qianshan
59091	Jiangxi Quannan
58503	Jiangxi Ruichang
57699	Jiangxi Shanggao
57783	Jiangxi Shangli
58623	Jiangxi Shangraoxian
58814	Jiangxi Shicheng
57896	Jiangxi Suichuan
57899	Jiangxi Taihe
57694	Jiangxi Tonggu
57895	Jiangxi Wanan
58615	Jiangxi Wannian
57698	Jiangxi Wanzai
58507	Jiangxi Wuning
58529	Jiangxi Wuyuan
58704	Jiangxi Xiajiang
57883	Jiangxi Xiaping
58693	Jiangxi Xinjian
57796	Jiangxi Xinyu
57995	Jiangxi Xinfeng
58804	Jiangxi Xingguo
57598	Jiangxi Xiushui
59102	Jiangxi Xunwu
57793	Jiangxi Yichun
58705	Jiangxi Yongfeng
57891	Jiangxi Yongxin
58905	Jiangxi Yudu
58612	Jiangxi Yugan
58634	Jiangxi Yushan
54339	Liaoning Anshan
54346	Liaoning Benxi

54483	Liaoning Caohekou
54243	Liaoning Changtu
54579	Liaoning Changhai
54565	Liaoning Changxingdao
54324	Liaoning Chaoyang
54475	Liaoning Dashiqiao
54497	Liaoning Dandong
54494	Liaoning Fengcheng
54351	Liaoning Fushun
54474	Liaoning Gaizhou
54452	Liaoning Jianchang
54326	Liaoning Jianpingxian
54321	Liaoning Jianpingzhen
54568	Liaoning Jinzhou
54244	Liaoning Kangping
54493	Liaoning Kuandian
54453	Liaoning Lianshan
54345	Liaoning Liaoyangxian
54332	Liaoning Liaozhong
54327	Liaoning Lingyuan
54338	Liaoning Panshan
54575	Liaoning Pikou
54569	Liaoning Pulandian
54259	Liaoning Qingyuan
54248	Liaoning Shenbei
54342	Liaoning Shenyang
54340	Liaoning Sujiatun
54454	Liaoning Suizhong
54336	Liaoning Taian
54249	Liaoning Tieling
54563	Liaoning Wafangdian
54252	Liaoning Xifeng
54353	Liaoning Xinbin
54333	Liaoning Xinmin
54455	Liaoning Xingcheng
54486	Liaoning Xiuyan
54325	Liaoning Yangshan
54471	Liaoning Yingkou
54236	Liaoning Zhangwu
54584	Liaoning Zhuanghe
53192	Inner Mongolia Abagaqi
54122	Inner Mongolia Alukeerqinqi
50647	Inner Mongolia Arunqi

54225	Inner Mongolia Aohanqi
54316	Inner Mongolia Balihan
54113	Inner Mongolia Balinyouqi
54027	Inner Mongolia Balinzuoqi
50928	Inner Mongolia Bayaertuhushuo
53446	Inner Mongolia Baotou
54226	Inner Mongolia Baoguotu
53384	Inner Mongolia Chayouhouqi
53481	Inner Mongolia Chayouqianqi
53378	Inner Mongolia Chayouzhongqi
54218	Inner Mongolia Chifeng
53457	Inner Mongolia Dalateqi
53352	Inner Mongolia Damaoqi
53348	Inner Mongolia Dashetai
53419	Inner Mongolia Dengkou
53543	Inner Mongolia Dongsheng
50915	Inner Mongolia Wuzhumuqindong
54208	Inner Mongolia Duolunxian
50425	Inner Mongolia Eerguna
50445	Inner Mongolia Elunchunqi
53529	Inner Mongolia Etukeqi
53730	Inner Mongolia Eduokeqianqi
50525	Inner Mongolia Evenkeqi
53068	Inner Mongolia Erlianhaote
54024	Inner Mongolia Fuhe
54214	Inner Mongolia Gangzi
54031	Inner Mongolia Gaoliban
53357	Inner Mongolia Guyangxian
53231	Inner Mongolia Hailisu
53420	Inner Mongolia Hangjinhouqi
53533	Inner Mongolia Hangjinqi
53469	Inner Mongolia Helingeerxian
53732	Inner Mongolia Henan
53463	Inner Mongolia Huhehaote
53466	Inner Mongolia Huhehaote suburb
53391	Inner Mongolia Huade
50924	Inner Mongolia Houlinguole
53480	Inner Mongolia Jining
54313	Inner Mongolia Kalaqinqi
54134	Inner Mongolia Kailu
54231	Inner Mongolia Kezuohouqi
54047	Inner Mongolia Kezuozhongqi
54117	Inner Mongolia Keshiketengqi

54234	Inner Mongolia Kulun
53475	Inner Mongolia Liangcheng
54115	Inner Mongolia Linxixian
53513	Inner Mongolia Linhe
53149	Inner Mongolia Mandula
50514	Inner Mongolia Manzhouli
50645	Inner Mongolia Moulidawawoer
53083	Inner Mongolia Narenbaolige
54223	Inner Mongolia Neiman
54320	Inner Mongolia Ningchengxian
54132	Inner Mongolia Qinglongshan
53562	Inner Mongolia Qingshuihexian
53385	Inner Mongolia Shangdu
54039	Inner Mongolia Shebotu
53362	Inner Mongolia Siziwang
50834	Inner Mongolia Suolun
54305	Inner Mongolia Taibushiqi
54135	Inner Mongolia Tongliao
50934	Inner Mongolia Tuquan
50434	Inner Mongolia Tulihe
53464	Inner Mongolia Tumutezuoqi
53455	Inner Mongolia Tuyouqi
53467	Inner Mongolia Tuoketuoxian
54213	Inner Mongolia Wengniuteqi
53512	Inner Mongolia Wuhai
50913	Inner Mongolia Wulagai
53433	Inner Mongolia Wulateqianqi
53336	Inner Mongolia Wulatezhongqi
53644	Inner Mongolia Wushenqi
53337	Inner Mongolia Wuyuan
53368	Inner Mongolia Wuchuanxian
54012	Inner Mongolia Xiwuzhumuqin
53367	Inner Mongolia Xilamuren
54102	Inner Mongolia Xilinhaote
53289	Inner Mongolia Xianghuangqi
50548	Inner Mongolia Xiaoergou
50603	Inner Mongolia Xinbaerhuyouqi
50618	Inner Mongolia Xinbaerhuzuoqi
53483	Inner Mongolia Xinghe
50526	Inner Mongolia Yakeshi
53545	Inner Mongolia Ejinhuoluoqi
53522	Inner Mongolia Yikewusu
50639	Inner Mongolia Zhalantun

54026	Inner Mongolia Zhalute
54205	Inner Mongolia Zhenglanqi
54204	Inner Mongolia Zhengxiangbaiqi
53553	Inner Mongolia Zhungeerqi
53472	Inner Mongolia Zhuozi
53817	Ningxia Guyuan
53806	Ningxia Haiyuan
53610	Ningxia Helan
53519	Ningxia Huinong
53910	Ningxia Liupanshan
53727	Ningxia Maihuangshan
53611	Ningxia Pingluo
53517	Ningxia Shitanjing
53615	Ningxia Taole
53810	Ningxia Tongxin
53881	Ningxia Weizhou
53612	Ningxia Wuzhong
53903	Ningxia Xiji
53707	Ningxia Xingren
53723	Ningxia Yanchi
53618	Ningxia Yongning
53705	Ningxia Zhongning
53704	Ningxia Zhongwei
56151	Qinghai Banma
56046	Qinghai Dari
52836	Qinghai Doulan
56045	Qinghai Gander
52856	Qinghai Gonghe
52868	Qinghai Guide
52955	Qinghai Guinan
56065	Qinghai Henan
52863	Qinghai Huzhu
52877	Qinghai Hualong
52963	Qinghai Jianzha
56067	Qinghai Jiuzhi
56033	Qinghai Maduo
56043	Qinghai Maqin
52876	Qinghai Minhe
56125	Qinghai Nangqian
52825	Qinghai Nuomuhong
52875	Qinghai Pingan
52854	Qinghai Qinghaihu 151
56034	Qinghai Qingshuihe

56021	Qinghai Qumacai
52941	Qinghai Shazhuyu
52957	Qinghai Tongde
52974	Qinghai Tongren
56004	Qinghai Tuotuohe
52908	Qinghai Wudaoliang
52866	Qinghai Xining
52943	Qinghai Xinghai
52972	Qinghai Xunhua
56029	Qinghai Yushu
56018	Qinghai Zaduo
52968	Qinghai Zeku
56016	Qinghai Zhiduo
54844	Shandong Anqiu
54734	Shandong Binzhou
58030	Shandong Cangshan
58002	Shandong Caoxian
54841	Shandong Changyi
54751	Shandong Longdao
54776	Shandong Chengshantou
58003	Shandong Chengwu
54814	Shandong Chiping
54909	Shandong Dingtao
54815	Shandong Donge
54911	Shandong Dongping
54819	Shandong Feicheng
54929	Shandong Feixian
54764	Shandong Fushan
54846	Shandong Gaomi
54729	Shandong Gaoqing
54906	Shandong Heze
54833	Shandong Huantai
54943	Shandong Huangdao
54855	Shandong Jimo
54823	Shandong Jinan
54821	Shandong Jiyang
54849	Shandong Jiaozhou
54939	Shandong Junan
54914	Shandong Juye
54904	Shandong Juancheng
54744	Shandong Kenli
54828	Shandong Laiwu
54852	Shandong Laiyang

54749	Shandong Laizhou
54726	Shandong Leling
54910	Shandong Liangshan
54806	Shandong Liaocheng
54802	Shandong Linqing
58032	Shandong Linshu
54938	Shandong Linyi
54712	Shandong Linyi
54753	Shandong Longkou
54923	Shandong Mengyin
54716	Shandong Ningjin
54752	Shandong Penglai
54842	Shandong Pingdu
54925	Shandong Pingyi
54759	Shandong Qixia
54812	Shandong Qihe
54831	Shandong Qingzhou
54945	Shandong Rizhao
54861	Shandong Rushan
54724	Shandong Shanghe
54832	Shandong Shouguang
54920	Shandong Sishui
54827	Shandong Taian
54927	Shandong Tengzhou
54843	Shandong Weifang
54777	Shandong Wendeng
54912	Shandong Wenshang
54940	Shandong Wulian
54709	Shandong Wucheng
54808	Shandong Xinxian
54922	Shandong Xintai
58021	Shandong Xuechen
54765	Shandong Yantai
54916	Shandong Yanzhou
54723	Shandong Yangxin
54932	Shandong Yishui
54836	Shandong Yiyuan
58022	Shandong Yicheng
54907	Shandong Yutai
54905	Shandong Yuncheng
58024	Shandong Zaozhuang
54727	Shandong Zhangqiu
54755	Shandong Zhaoyuan

54848	Shandong Zhucheng
54830	Shandong Zibo
54919	Shandong Zoucheng
54822	Shandong Zouping
53877	Shanxi Anze
53873	Shanxi Changzi
53487	Shanxi Datong
53579	Shanxi Daixian
53676	Shanxi Dingxiang
53585	Shanxi Fanshi
53760	Shanxi Fangshan
53865	Shanxi Fenxi
53966	Shanxi Fushan
53973	Shanxi Gaoping
53763	Shanxi Gujiao
53874	Shanxi Guxian
53590	Shanxi Guangling
53788	Shanxi Heshun
53957	Shanxi Hejin
53564	Shanxi Hequ
53866	Shanxi Hongtong
53582	Shanxi Hunyuan
53859	Shanxi Jixian
53954	Shanxi Jishan
53677	Shanxi Jiancaoping
53965	Shanxi Jiangxian
53860	Shanxi Jiaokou
53863	Shanxi Jiexiu
53666	Shanxi Jingle
53662	Shanxi Kelan
53665	Shanxi Lanxian
53764	Shanxi Lishi
53878	Shanxi Licheng
53659	Shanxi Linxian
53862	Shanxi Lingshi
53981	Shanxi Lingchuan
53753	Shanxi Liulin
53880	Shanxi Lucheng
53577	Shanxi Ningwu
53565	Shanxi Pianguan
53687	Shanxi Pingding
53574	Shanxi Pinglu
53778	Shanxi Pingyao

53864	Shanxi Puxian
53970	Shanxi Qingshui
53872	Shanxi Qinxian
53875	Shanxi Qinyuan
53774	Shanxi Qingxu
57053	Shanxi Ruicheng
53576	Shanxi Shanyin
53575	Shanxi Shenchi
53759	Shanxi Shilou
53780	Shanxi Shouyang
53578	Shanxi Shuozhou
53775	Shanxi Taigu
53956	Shanxi Wanrong
53588	Shanxi Wutaishan
53681	Shanxi Wutaixian
53663	Shanxi Wuzhai
53871	Shanxi Wuxiang
53853	Shanxi Xixian
53953	Shanxi Xiangning
53861	Shanxi Xiangfen
53679	Shanxi Xiaodian
53768	Shanxi Xiaoyi
53674	Shanxi Xinfu
53964	Shanxi Xinjiang
53664	Shanxi Xingxian
53959	Shanxi Yanhu
53486	Shanxi Yanggao
53782	Shanxi Yangquan
53584	Shanxi Yingxian
53852	Shanxi Yonghe
57052	Shanxi Yongji
53478	Shanxi Youyu
53685	Shanxi Yuxian
53787	Shanxi Yushe
53968	Shanxi Yuanqu
53673	Shanxi Yuanping
53767	Shanxi Zhongyang
53786	Shanxi Zuoquan
57245	Shaanxi Ankang
53841	Shaanxi Ansai
57254	Shaanxi Baihe
53941	Shaanxi Baishui
57016	Shaanxi Baoji

57128	Shaanxi Chenggu
53949	Shaanxi Chengcheng
57043	Shaanxi Dali
57153	Shaanxi Danfeng
53725	Shaanxi Dingbian
57113	Shaanxi Fengxian
57134	Shaanxi Foping
57026	Shaanxi Fufeng
53567	Shaanxi Fugu
57042	Shaanxi Fuping
53931	Shaanxi Fuxian
53848	Shaanxi Ganquan
57233	Shaanxi Hanyin
53950	Shaanxi Heyang
53740	Shaanxi Hengshan
57046	Shaanxi Huashan
53944	Shaanxi Huangling
53946	Shaanxi Huanglong
53658	Shaanxi Jiaxian
53735	Shaanxi Jingbian
57247	Shaanxi Langao
57029	Shaanxi Liquan
57022	Shaanxi Linyou
57124	Shaanxi Liuba
57003	Shaanxi Longxian
57106	Shaanxi Lueyang
57057	Shaanxi Luonan
57027	Shaanxi Meixian
57119	Shaanxi Mianxian
57213	Shaanxi Nanzheng
57211	Shaanxi Ningqiang
57137	Shaanxi Ningshan
57248	Shaanxi Pingli
53948	Shaanxi Pucheng
57021	Shaanxi Qianyang
53757	Shaanxi Qingjian
57041	Shaanxi Sanyuan
57155	Shaanxi Shanyang
57154	Shaanxi Shangnan
57143	Shaanxi Shangxian
53651	Shaanxi Shenmu
53754	Shaanxi Suide
53947	Shaanxi Tongchuan

57045	Shaanxi Weinan
53756	Shaanxi Wubao
53738	Shaanxi Wuqi
57034	Shaanxi Wugong
57038	Shaanxi Xingping
57242	Shaanxi Xunyang
53938	Shaanxi Xunyi
53854	Shaanxi Yanchang
53850	Shaanxi Yanchuan
57037	Shaanxi Yaoxian
53857	Shaanxi Yichuan
57030	Shaanxi Yongshou
53646	Shaanxi Yulin
57238	Shaanxi Zhenba
57343	Shaanxi Zhenping
53832	Shaanxi Zhidan
53748	Shaanxi Zichang
57231	Shaanxi Ziyang
57140	Shaanxi Zhashui
58362	Shanghai Baoshan
58463	Shanghai Fengxian
58460	Shanghai Jinshan
58361	Shanghai Minhang
56171	Sichuan Aba
56247	Sichuan Batang
57313	Sichuan Bazhong
56147	Sichuan Baiyu
56273	Sichuan Baoxing
56580	Sichuan Butuo
56593	Sichuan Changning
56181	Sichuan Chongzhou
57328	Sichuan Dachuan
57420	Sichuan Dazhu
56263	Sichuan Danba
56167	Sichuan Daofu
56357	Sichuan Daocheng
56441	Sichuan Derong
56569	Sichuan Dechang
56144	Sichuan Dege
56198	Sichuan Deyang
57503	Sichuan Dongxing
56188	Sichuan Dujiangyan
56387	Sichuan Ebian

56385	Sichuan Emeishan
56399	Sichuan Fushun
56473	Sichuan Ganluo
56146	Sichuan Ganzi
57411	Sichuan Gaoping
56499	Sichuan Gongxian
57415	Sichuan Guangan
57206	Sichuan Guangyuan
56376	Sichuan Hanyuan
57603	Sichuan Hejiang
56185	Sichuan Heishui
56173	Sichuan Hongyuan
56380	Sichuan Hongya
56675	Sichuan Huidong
56671	Sichuan Huili
56382	Sichuan Jiajiang
56389	Sichuan Qianwei
57208	Sichuan Jiange
57600	Sichuan Jiangan
56168	Sichuan Jinchuan
56296	Sichuan Jintang
56584	Sichuan Jinyang
56390	Sichuan Jingyan
56462	Sichuan Jiulong
56097	Sichuan Jiuzhaigou
57329	Sichuan Kaijiang
56374	Sichuan Kangding
57306	Sichuan Langzhong
56485	Sichuan Leibo
56257	Sichuan Litang
56184	Sichuan Lixian
57416	Sichuan Linshui
56286	Sichuan Longquanyi
57507	Sichuan Longchang
56279	Sichuan Lushan
56371	Sichuan Luding
56158	Sichuan Luhuo
56480	Sichuan Mabian
56172	Sichuan Maerkang
56180	Sichuan Maoxian
56487	Sichuan Meigu
56670	Sichuan Miyi
56186	Sichuan Mianzhu

56474	Sichuan Mianning
56280	Sichuan Mingshan
56459	Sichuan Muli
56490	Sichuan Muchuan
57314	Sichuan Nanbu
57216	Sichuan Nanjiang
56493	Sichuan Nanxi
56666	Sichuan Panzhihua
56289	Sichuan Pengshan
56189	Sichuan Pengzhou
57324	Sichuan Pingchang
56494	Sichuan Pingshan
56281	Sichuan Pujiang
56575	Sichuan Puge
57204	Sichuan Qingchuan
56383	Sichuan Qingshen
57413	Sichuan Quxian
56164	Sichuan Rangtang
56297	Sichuan Renshou
56079	Sichuan Ruoergai
56152	Sichuan Seda
57401	Sichuan Shehong
56378	Sichuan Shimian
56038	Sichuan Shiqu
56182	Sichuan Songpan
57405	Sichuan Suining
57320	Sichuan Tongjiang
57237	Sichuan Wanyuan
57217	Sichuan Wangcang
56395	Sichuan Weiyuan
56187	Sichuan Wenjiang
56183	Sichuan Wenchuan
57417	Sichuan Wusheng
56571	Sichuan Xichang
57309	Sichuan Xichong
56478	Sichuan Xide
56443	Sichuan Xiangcheng
56178	Sichuan Xiaojin
56251	Sichuan Xinlong
56496	Sichuan Xingwen
57608	Sichuan Xuyong
56267	Sichuan Yajiang
56665	Sichuan Yanbian

56565	Sichuan Yanyuan
57315	Sichuan Yilong
56491	Sichuan Yibinxian
56373	Sichuan Yingjing
57318	Sichuan Yingshan
56475	Sichuan Yuexi
56479	Sichuan Chaojue
56298	Sichuan Ziyang
56393	Sichuan Zizhong
56396	Sichuan Zigong
54525	Tianjin Baodi
54645	Tianjin Dagang
54622	Tianjin Jinnan
54619	Tianjin Jinghai
54623	Tianjin Tanggu
54523	Tianjin Wuqing
56228	Tibet Basu
56227	Tibet Bomi
56434	Tibet Chayu
56137	Tibet Changdu
55493	Tibet Dangxiong
56116	Tibet Dingqing
55664	Tibet Dingri
55248	Tibet Gaize
55680	Tibet Jiangzi
55569	Tibet Lazi
56128	Tibet Leiwuqi
56312	Tibet Linzhi
56223	Tibet Luolong
56317	Tibet Miling
55593	Tibet Muozhugongka
55299	Tibet Naqu
55572	Tibet Nanmulin
55655	Tibet Nielamu
55437	Tibet Pulan
55228	Tibet Shiquanhe
55598	Tibet Zedang
56331	Tibet Zuogong
51058	Xinjiang Akedala
51628	Xinjiang Akesu
51730	Xinjiang Alaer
51232	Xinjiang Alashankou
51076	Xinjiang Aletai

51704	Xinjiang Atushi
51722	Xinjiang Awati
52101	Xinjiang Balikun
51542	Xinjiang Bayinbuluke
51238	Xinjiang Bole
51060	Xinjiang Buerjin
51826	Xinjiang Cele
51368	Xinjiang Changji
51477	Xinjiang Dabancheng
51145	Xinjiang Emin
51068	Xinjiang Fuhai
51377	Xinjiang Fukang
51087	Xinjiang Fuyun
51707	Xinjiang Jiashi
51053	Xinjiang Habahe
52203	Xinjiang Hami
51156	Xinjiang Hebukesaier
51828	Xinjiang Hetan
52313	Xinjiang Hongliuhe
51367	Xinjiang Hutubi
51378	Xinjiang Jimusaer
51334	Xinjiang Jinghe
51709	Xinjiang Kashi
51720	Xinjiang Kepin
51243	Xinjiang Kelamayi
51656	Xinjiang Kuerle
51526	Xinjiang Kumishi
51829	Xinjiang Luopu
51359	Xinjiang Manasi
51810	Xinjiang Maigaiti
51369	Xinjiang Miquan
51839	Xinjiang Minfeng
51827	Xinjiang Moyu
51482	Xinjiang Mulei
52112	Xinjiang Naomaohu
51818	Xinjiang Pishan
51855	Xinjiang Qiemuo
51357	Xinjiang Shawan
51639	Xinjiang Shaya
51811	Xinjiang Shashe
51133	Xinjiang Tacheng
51747	Xinjiang Tazhong
51470	Xinjiang Tianchi

51468	Xinjiang Tianshandaxigou
51765	Xinjiang Tieqianlike
51573	Xinjiang Tulufan
51572	Xinjiang Tulufandongkan
51571	Xinjiang Tuokexun
51241	Xinjiang Touli
51469	Xinjiang Urumqi Pastoral Test Station
51705	Xinjiang Wuqia
51627	Xinjiang Wushi
51636	Xinjiang Xinhe
51567	Xinjiang Yanqi
51814	Xinjiang Yecheng
52118	Xinjiang Yiwu
51802	Xinjiang Yengjisha
51931	Xinjiang Yutian
51137	Xinjiang Yumin
51717	Xinjiang Yuepuhu
51815	Xinjiang Zepu
56863	Yunnan Anning
56748	Yunnan Baoshan
56752	Yunnan Binchuan
56944	Yunnan Cangyuan
56843	Yunnan Changning
56873	Yunnan Chengjiang
56582	Yunnan Daguan
56751	Yunnan Dali
56444	Yunnan Deqin
56688	Yunnan Dongchuan
56898	Yunnan Eshan
56649	Yunnan Eryuan
56846	Yunnan Fengqing
56641	Yunnan Fugong
56772	Yunnan Fumin
59205	Yunnan Funing
56790	Yunnan Fuyuan
56984	Yunnan Gejiu
56946	Yunnan Gengma
56533	Yunnan Gongshan
59007	Yunnan Guangnan
56654	Yunnan Heqing
56879	Yunnan Huaning
56664	Yunnan Huaping
56646	Yunnan Jianchuan

56977	Yunnan Jiangcheng
56987	Yunnan Jinping
56871	Yunnan Jinning
56856	Yunnan Jingdong
56952	Yunnan Jinggu
56982	Yunnan Kaiyuan
56778	Yunnan Kunming
56645	Yunnan Lanping
56954	Yunnan Lancang
56651	Yunnan Lijiang
56840	Yunnan Lianghe
56951	Yunnan Lincang
56643	Yunnan Liuku
56841	Yunnan Longling
56835	Yunnan Longchuan
56886	Yunnan Luxi
56585	Yunnan Ludian
56777	Yunnan Lufeng
56978	Yunnan Luchun
56891	Yunnan Luoping
56782	Yunnan Malong
56958	Yunnan Menghai
56969	Yunnan Mengla
56949	Yunnan Menglian
56755	Yunnan Midu
56885	Yunnan Mile
56962	Yunnan Mojiang
56766	Yunnan Mouding
56767	Yunnan Nanhua
56567	Yunnan Ninglang
56673	Yunnan Qiaojia
56889	Yunnan Qiubei
56783	Yunnan Qujing
56883	Yunnan Shizong
56842	Yunnan Shidian
56881	Yunnan Shilin
56970	Yunnan Shiping
56862	Yunnan Shuangbai
56950	Yunnan Shuangjiang
56964	Yunnan Simao
56785	Yunnan Songming
56483	Yunnan Suijiang
56739	Yunnan Tengchong

56596	Yunnan Weixin
56757	Yunnan Weishan
56548	Yunnan Weixi
56994	Yunnan Wenshan
56774	Yunnan Wuding
56992	Yunnan Xichou
56948	Yunnan Ximeng
56543	Yunnan Xianggelila
56869	Yunnan Xinping
56697	Yunnan Xuanwei
56497	Yunnan Yanjin
56991	Yunnan Yanshan
56745	Yunnan Yangbi
56764	Yunnan Yaoan
56880	Yunnan Yiliang
56594	Yunnan Yiliang
56870	Yunnan Yimen
56836	Yunnan Yingjiang
56849	Yunnan Yongde
56746	Yunnan Yongping
56669	Yunnan Yongren
56652	Yunnan Yongsheng
56875	Yunnan Yuxi
56966	Yunnan Yuanjiang
56976	Yunnan Yuanyang
56742	Yunnan Yunlong
56854	Yunnan Yunxian
56839	Yunnan Zhenkang
56595	Yunnan Zhenxiong
56867	Yunnan Zhenyuan
58446	Zhejiang Anji
58443	Zhejiang Changxing
58631	Zhejiang Changshan
58467	Zhejiang Cixi
58666	Zhejiang Dachen
58484	Zhejiang Daishan
58454	Zhejiang Deqing
58558	Zhejiang Dongyang
58565	Zhejiang Fenghua
58449	Zhejiang Fuyang
58457	Zhejiang Hangzhou
58665	Zhejiang Hongjia
58450	Zhejiang Huzhou

58451	Zhejiang Jiashan
58452	Zhejiang Jiaxing
58544	Zhejiang Jiande
58632	Zhejiang Jiangshan
58549	Zhejiang Jinhua
58654	Zhejiang Jinyun
58648	Zhejiang Jingning
58537	Zhejiang Kaihua
58656	Zhejiang Leqing
58646	Zhejiang Lishui
58448	Zhejiang Linan
58660	Zhejiang Linhai
58647	Zhejiang Longquan
58547	Zhejiang Longyou
58567	Zhejiang Ninghai
58560	Zhejiang Panan
58751	Zhejiang Pingyang
58546	Zhejiang Pujiang
58570	Zhejiang Putuo
58657	Zhejiang Qingtian
58568	Zhejiang Sanmen
58453	Zhejiang Shaoxing
58472	Zhejiang Shengsi
58556	Zhejiang Shengzhou
58569	Zhejiang Shipu
58644	Zhejiang Suichang
58746	Zhejiang Taishun
58559	Zhejiang Tiantai
58542	Zhejiang Tonglu
58664	Zhejiang Wenling
58750	Zhejiang Wencheng
58642	Zhejiang Wuyi
58652	Zhejiang Xianju
58566	Zhejiang Xiangshan
58555	Zhejiang Xinchang
58562	Zhejiang Yinzhou
58658	Zhejiang Yongjia
58643	Zhejiang Yongkang
58742	Zhejiang Yunhe
58561	Zhejiang Zhenhai
58550	Zhejiang Zhuji
57518	Chongqing Banan
57511	Chongqing Beibei

57514	Chongqing Bishan
57520	Chongqing Changshou
57333	Chongqing Chengkou
57502	Chongqing Dazu
57425	Chongqing Dianjiang
57523	Chongqing Fengdu
57517	Chongqing Jiangjin
57338	Chongqing Kaixian
57519	Chongqing Nanchuan
57537	Chongqing Pengshui
57612	Chongqing Qijiang
57536	Chongqing Qianjiang
57505	Chongqing Rongchang
57438	Chongqing Shizhu
57510	Chongqing Tongliang
57409	Chongqing Tongnan
57509	Chongqing Wansheng
57432	Chongqing Wanzhou
57349	Chongqing Wushan
57345	Chongqing Wuxi
57525	Chongqing Wulong
57506	Chongqing Yongchuan
57513	Chongqing Yubei
57339	Chongqing Yunyang
57437	Chongqing Zhongxian

Table S6. 1743 monitoring stations whose PM2.5 emissions are from residential sector

58102	Anhui Bozhou
58220	Anhui Changfeng
58326	Anhui Chaohu
58236	Anhui Chuzhou
58015	Anhui Dangshan
58225	Anhui Dingyuan
58323	Anhui Feidong
58320	Anhui Feixi
58212	Anhui Fengtai
58222	Anhui Fengyang
58202	Anhui Funan
58203	Anhui Fuyang
58128	Anhui Guzhen
58441	Anhui Guangde
58330	Anhui Hanshan
58127	Anhui Huaiyuan
58437	Anhui Huangshan
58214	Anhui Huoqiu
58314	Anhui Huoshan
58108	Anhui Jieshou
58306	Anhui Jinzhai
58432	Anhui Jingxian
58435	Anhui Jingde
58234	Anhui Laian
58442	Anhui Langxi
58117	Anhui Leysin
58125	Anhui Lingbi
58311	Anhui Luan
58327	Anhui Lujiang
58336	Anhui Maanshan
58118	Anhui Mengcheng
58223	Anhui Mingguang
58431	Anhui Nanling
58436	Anhui Ningguo
58520	Anhui Qimen
58316	Anhui Shucheng
58126	Anhui Sixian
58417	Anhui Susong
58122	Anhui Suzhou
58113	Anhui Suixi

58109	Anhui Taihe
58240	Anhui Tianchang
58112	Anhui Tianzhushan
58319	Anhui Tongcheng
58429	Anhui Tongling
58531	Anhui Tunxi
58114	Anhui Woyang
58329	Anhui Wuwei
58334	Anhui Wuhu
58338	Anhui Wuhuxian
58129	Anhui Wuhe
58530	Anhui Shexian
58016	Anhui Xiaoxian
58433	Anhui Xuancheng
58523	Anhui Yixian
58210	Anhui Yingshang
58317	Anhui Yuexi
54511	Beijing
54499	Beijing Changping
54399	Beijing Haidian
54416	Beijing Miyun
54421	Beijing Miyunshangdianzi
54424	Beijing Pinggu
54398	Beijing Shunyi
54431	Beijing Tongzhou
54597	Beijing Xiayunling
54406	Beijing Yanqing
54501	Beijing Zhaitang
58929	Fujian Anxi
58941	Fujian Changle
59122	Fujian Changtai
58911	Fujian Changting
59133	Fujian Chongwu
58923	Fujian Datian
58748	Fujian Fuan
58754	Fujian Fuding
58942	Fujian Fuqing
58836	Fujian Gutian
58724	Fujian Guangze
58928	Fujian Huaan
58822	Fujian Jianning
58734	Fujian Jianyang
58931	Fujian Jiuxianshan

58848	Fujian Lianjiang
58844	Fujian Minhou
58839	Fujian Shuqing
58824	Fujian Mingxi
59131	Fujian Nanan
59124	Fujian Nanjing
58846	Fujian Ningde
58818	Fujian Ninghuai
59125	Fujian Pinghe
58944	Fujian Pingtan
58946	Fujian Putian
58731	Fujian Pucheng
58828	Fujian Sanming
58725	Fujian Shaowu
58744	Fujian Shouning
58823	Fujian Shunchang
58735	Fujian Songxi
58820	Fujian Taining
59130	Fujian Tongan
58917	Fujian Wuping
58730	Fujian Wuyishan
58843	Fujian Xiapu
58938	Fujian Xiuyu
59113	Fujian Yongding
58932	Fujian Yongtai
58837	Fujian Youxi
59322	Fujian Yunxiao
58926	Fujian Zhangping
59129	Fujian Zhangpu
59320	Fujian Zhaoan
58749	Fujian Zherong
58736	Fujian Zhenghe
58747	Fujian Zhouning
52995	Gansu Anding
52896	Gansu Baiyin
57102	Gansu Chengxian
56095	Gansu Dangchang
56084	Gansu Dibu
52981	Gansu Dongxiang
57001	Gansu Gangu
52884	Gansu Gaolan
52546	Gansu Gaitai
52784	Gansu Gulang

52982	Gansu Guanghe
53934	Gansu Huishui
56080	Gansu Hezuo
52985	Gansu Hezheng
53930	Gansu Huachi
52996	Gansu Huajialing
53927	Gansu Huating
53821	Gansu Huanxian
57110	Gansu Huixian
52993	Gansu Huining
53926	Gansu Jingchuan
52797	Gansu Jingtai
52895	Gansu Jingyuan
53906	Gansu Jingning
52533	Gansu Jiuquan
52988	Gansu Kangle
57105	Gansu Kangxian
53915	Gansu Kongtong
57007	Gansu Lixian
57111	Gansu Liangdang
56081	Gansu Lintan
52986	Gansu Lintao
52557	Gansu Linze
53924	Gansu Lingtai
56092	Gansu Longxi
56071	Gansu Luqu
52323	Gansu Mazongshan
56074	Gansu Maqu
57014	Gansu Maiji
52656	Gansu Minle
52681	Gansu Minqin
56093	Gansu Minxian
57002	Gansu Qinan
57011	Gansu Qingshui
53829	Gansu Qingcheng
52515	Gansu Subei
52643	Gansu Sunan
57006	Gansu Tianshui
52881	Gansu Tianzhu
52998	Gansu Weiyuan
56192	Gansu Wenxian
52787	Gansu Wushaoling
56096	Gansu Wudu

57004	Gansu Wushan
52679	Gansu Wuwei
53923	Gansu Xifeng
52978	Gansu Xiahe
52674	Gansu Yongchang
52885	Gansu Yongdeng
52980	Gansu Yongjing
52983	Gansu Yuzhong
57012	Gansu Zhangjiachuan
56091	Gansu Zhangxian
53925	Gansu Zhenyuan
53935	Gansu Zhengning
56094	Gansu Zhouqu
53917	Gansu Zhuanglang
56082	Gansu Zhuoni
59297	Guangdong Boluo
59312	Guangdong Chaozhou
59285	Guangdong Conghua
59116	Guangdong Daipu
59289	Guangdong Dongguan
59487	Guangdong Doumen
59477	Guangdong Enping
59481	Guangdong Panyu
59310	Guangdong Fengshun
59087	Guangdong Fogang
59653	Guangdong Gaozhou
59271	Guangdong Guangning
59287	Guangdong Guangzhou
59500	Guangdong Haifeng
59099	Guangdong Heping
59293	Guangdong Heyuan
59473	Guangdong Heshan
59284	Guangdong Huadu
59655	Guangdong Huazhou
59270	Guangdong Huaiji
59492	Guangdong Huidong
59114	Guangdong Jiaoling
59306	Guangdong Jiexi
59475	Guangdong Kaiping
57988	Guangdong Lechang
59750	Guangdong Leizhou
59096	Guangdong Lianping
59074	Guangdong Lianshan

59654	Guangdong Lianjiang
59107	Guangdong Longchuan
59290	Guangdong Longmen
59502	Guangdong Lufeng
59462	Guangdong Luoding
59659	Guangdong Maoming
59117	Guangdong Meixian
57996	Guangdong Nanxiong
59106	Guangdong Pingyuan
59314	Guangdong Puning
59280	Guangdong Qingyuan
59313	Guangdong Raoping
57989	Guangdong Renhua
59081	Guangdong Ruyuan
59279	Guangdong Sanshui
59316	Guangdong Shantou
59082	Guangdong Shaoguan
59493	Guangdong Shenzhen
59090	Guangdong Shixing
59276	Guangdong Sihui
59650	Guangdong Suixi
59094	Guangdong Wengyuan
59656	Guangdong Wuchuan
59303	Guangdong Wuhua
59476	Guangdong Xinhui
59470	Guangdong Xinxing
59456	Guangdong Xinyi
59109	Guangdong Xingning
59754	Guangdong Xuwen
59469	Guangdong Yangchun
59075	Guangdong Yangshan
59088	Guangdong Yingde
59268	Guangdong Yunan
59471	Guangdong Yunfu
59488	Guangdong Zhuhai
59304	Guangdong Zijin
59027	Guangxi Bama
59211	Guangxi Baise
59451	Guangxi Beiliu
59238	Guangxi Binyang
59449	Guangxi Bobai
59266	Guangxi Cangwu
59454	Guangxi Cenxi

59421	Guangxi Daxin
59215	Guangxi Debao
59037	Guangxi Duan
59021	Guangxi Fengshan
57964	Guangxi Guanyang
59640	Guangxi Hepu
59023	Guangxi Hechi
59065	Guangxi Hezhou
59441	Guangxi Hengxian
59057	Guangxi Jinxiu
59218	Guangxi Jingxi
59242	Guangxi Laibin
59012	Guangxi Leye
59055	Guangxi Lipu
57954	Guangxi Lingui
59446	Guangxi Lingshan
59015	Guangxi Lingyun
59041	Guangxi Liucheng
57942	Guangxi Longsheng
59229	Guangxi Longan
59457	Guangxi Luchuan
59045	Guangxi Luzhai
59230	Guangxi Mashan
59058	Guangxi Mengshan
59209	Guangxi Napo
59431	Guangxi Nanning
59053	Guangxi Pingle
59255	Guangxi Pingnan
59419	Guangxi Pingxiang
59448	Guangxi Pubei
59632	Guangxi Qinzhou
57960	Guangxi Quanzhou
59452	Guangxi Rongxian
57948	Guangxi Rongshui
57941	Guangxi Sanjiang
59235	Guangxi Shanglin
59429	Guangxi Shangsi
59256	Guangxi Tengxian
59227	Guangxi Tiandeng
57927	Guangxi Tiane
59224	Guangxi Tiandong
59017	Guangxi Tianlin
59265	Guangxi Wuzhou

59237	Guangxi Wuming
59246	Guangxi Wuxuan
59004	Guangxi Xilin
59241	Guangxi Xiangzhou
59038	Guangxi Xincheng
57955	Guangxi Xingan
59034	Guangxi Yizhou
59435	Guangxi Yongning
57949	Guangxi Yongfu
57859	Guangxi Ziyuan
57806	Guizhou Anshun
57707	Guizhou Bijie
57909	Guizhou Ceheng
57735	Guizhou Cengong
57936	Guizhou Congjiang
57708	Guizhou Dafang
57623	Guizhou Daozhen
57637	Guizhou Dejiang
57827	Guizhou Duyun
57922	Guizhou Dushan
57723	Guizhou Fenggang
57821	Guizhou Fuquan
57903	Guizhou Guanling
57824	Guizhou Guiding
56598	Guizhou Hezhang
57822	Guizhou Huangping
57912	Guizhou Huishui
57835	Guizhou Jianhe
57736	Guizhou Jiangkou
57714	Guizhou Jinsha
57844	Guizhou Jinping
57719	Guizhou Kaiyang
57825	Guizhou Kaili
57837	Guizhou Leishan
57839	Guizhou Liping
57926	Guizhou Libo
57807	Guizhou Liuzhi
57913	Guizhou Longli
57916	Guizhou Luodian
57722	Guizhou Meitan
57800	Guizhou Nayong
56793	Guizhou Panxian
57814	Guizhou Pingba

57921	Guizhou Pingtang
56792	Guizhou Puan
57803	Guizhou Xianxi
57900	Guizhou Qinglong
57932	Guizhou Rongjiang
57923	Guizhou Sandu
57832	Guizhou Sansui
57737	Guizhou Shibing
57734	Guizhou Shiqian
57731	Guizhou Sinan
57647	Guizhou Songtao
57834	Guizhou Taijiang
57840	Guizhou Tianzhu
57606	Guizhou Tongzi
57742	Guizhou Wanshan
57906	Guizhou Wangmo
56691	Guizhou Weining
57728	Guizhou Wengan
57634	Guizhou Wuchuan
57718	Guizhou Xifeng
57907	Guizhou Xingyi
57811	Guizhou Xiuwen
57636	Guizhou Yanhe
57729	Guizhou Yuqing
57739	Guizhou Yuping
57905	Guizhou Zhenfeng
57625	Guizhou Zhengan
57805	Guizhou Zhijin
57910	Guizhou Ziyun
57717	Guizhou Zunyi
59848	Hainan Baisha
59945	Hainan Baoting
59847	Hainan Changjiang
59843	Hainan Chengmai
59845	Hainan Danzhou
59851	Hainan Dingan
59758	Hainan Haikou
59842	Hainan Lingao
59855	Hainan Qonghai
59854	Hainan Tunchang
59951	Hainan Wanning
59856	Hainan Wenchang
54605	Hebei Anxin

53785	Hebei Baixiang
54618	Hebei Botou
54616	Hebei Cangzhou
54535	Hebei Caofeidian
54540	Hebei Changli
54423	Hebei Chengde
54430	Hebei Chengdexian
54404	Hebei Chicheng
54304	Hebei Chongli
54510	Hebei Dachang
54613	Hebei Dacheng
54804	Hebei Daming
53696	Hebei Dingzhou
54713	Hebei Dongguang
54308	Hebei Fengning
53894	Hebei Fengfeng
54541	Hebei Funing
54710	Hebei Fucheng
53690	Hebei Fuping
54506	Hebei Gaobeidian
54603	Hebei Goyang
54301	Hebei Guyuan
54512	Hebei Guan
54707	Hebei Gucheng
54809	Hebei Guantao
54631	Hebei Guangzong
54628	Hebei Haixing
53892	Hebei Handan
54614	Hebei Hejian
54702	Hebei Hengshui
53491	Hebei Huaian
54405	Hebei Huailai
54624	Hebei Huanghua
54640	Hebei Jize
53689	Hebei Jinzhou
54711	Hebei Jingxian
53799	Hebei Julu
53392	Hebei Kangbao
54432	Hebei Kuancheng
53599	Hebei Laiyuan
54539	Hebei Leting
54801	Hebei Linxi
53773	Hebei Linzhang

53680	Hebei Lingshou
54318	Hebei Longhua
53794	Hebei Longyao
54438	Hebei Lulong
53789	Hebei Luancheng
54437	Hebei Luannan
54420	Hebei Luanping
53796	Hebei Ningjin
54319	Hebei Pingquan
54439	Hebei Qianan
54434	Hebei Qianxi
54449	Hebei Qinhuangdao
54436	Hebei Qinglong
54615	Hebei Qingxian
54706	Hebei Qinghe
53682	Hebei Quyang
53893	Hebei Quzhou
54606	Hebei Raoyang
54610	Hebei Renqiu
54503	Hebei Rongcheng
54520	Hebei Sanhe
53781	Hebei Shahe
53397	Hebei Shangyi
53886	Hebei Shexian
54608	Hebei Shenzhou
53596	Hebei Shunping
54534	Hebei Tangshan
53692	Hebei Tangxian
53499	Hebei Wanquan
54800	Hebei-Weixian
54311	Hebei Weichang
53593	Hebei Weixian
53896	Hebei Weixian
54612	Hebei Wenan
53699	Hebei Wuji
53890	Hebei Wuan
54700	Hebei Wuqiang
54703	Hebei Wuyi
54521	Hebei Xianghe
54701	Hebei Xinji
54633	Hebei Xinhe
54425	Hebei Xinglong
53688	Hebei Xingtang

54601	Hebei Xushui
53498	Hebei Xuanhua
53492	Hebei Yangyuan
54519	Hebei Yongqing
54522	Hebei Yutian
53795	Hebei Zanhuang
53399	Hebei Zhangbei
54401	Hebei Zhangjiakou
54408	Hebei Zhulu
54502	Hebei Zhuozhou
54429	Hebei Zunhua
57087	Henan Changge
53998	Henan Changheng
58100	Henan Dancheng
57082	Henan Dengfeng
54903	Henan Fanxian
57179	Henan Fangcheng
53983	Henan Fengqiu
57080	Henan Gongyi
58208	Henan Gushi
57299	Henan Guangshan
57192	Henan Huaiyang
58207	Henan Huangchuan
53985	Henan Huixian
57390	Henan Jigongshan
53978	Henan Jiyuan
57180	Henan Jiaxian
53982	Henan Jiaozuo
53992	Henan Junxian
57091	Henan Kaifeng
57093	Henan Lankao
53889	Henan Linzhou
57183	Henan Linying
57056	Henan Lingbao
57173	Henan Lushan
57066	Henan Luoning
57186	Henan Luohe
57071	Henan Mengjin
57072	Henan Mengzhou
57281	Henan Biyang
58004	Henan Minquan
53993	Henan Neihuang
57169	Henan Neixiang

57178	Henan Nanyang
57176	Henan Nanzhao
57292	Henan Pingyu
54900	Henan Puyang
53974	Henan Qixian
57096	Henan Qixian
53972	Henan Qinyang
54902	Henan Qingfeng
57197	Henan Runan
57078	Henan Ruyang
57075	Henan Ruzhou
57051	Henan Sanmenxia
58301	Henan Shangcheng
58005	Henan Shangqiu
57187	Henan Sheqi
57063	Henan Mianchi
57189	Henan Suiping
54817	Henan Taiqian
57099	Henan Taikang
53991	Henan Tangyin
57273	Henan Tanghe
57285	Henan Tongbai
57079	Henan Wenxian
57177	Henan Wugang
57185	Henan Wuyang
57193	Henan Xihua
57188	Henan Xiping
57156	Henan Xixia
57296	Henan Xixian
58017	Henan Xiayi
57182	Henan Xiangcheng
57070	Henan Xinan
57293	Henan Xincai
57396	Henan Xixian
53986	Henan Xinxiang
57271	Henan Xinye
57297	Henan Xinyang
53984	Henan Xiuwu
57089	Henan Xuchang
57095	Henan Yanling
57184	Henan Yexian
57074	Henan Yichuan
57081	Henan Xingyang

58006	Henan Yucheng
58007	Henan Echeng
57175	Henan Zhenping
57295	Henan Zhengyang
57090	Henan Zhongmou
57290	Henan Zhumadian
50958	Heilongjiang Acheng
50468	Heilongjiang Aihui
50854	Heilongjiang Anda
50867	Heilongjiang Bayan
50755	Heilongjiang Baiquan
50888	Heilongjiang Baoqing
50656	Heilongjiang Beian
50137	Heilongjiang Beijicun
50853	Heilongjiang Beilin
50960	Heilongjiang Binxian
50973	Heilongjiang Boli
50850	Heilongjiang Daqing
50842	Heilongjiang Dumeng
50964	Heilongjiang Zhengfang
50779	Heilongjiang Fuyuan
50788	Heilongjiang Fujin
50742	Heilongjiang Fuyu
50741	Heilongjiang Gannan
50953	Heilongjiang Haerbin
54092	Heilongjiang Hailin
50756	Heilongjiang Hailun
50775	Heilongjiang Hegang
50956	Heilongjiang Hulan
50353	Heilongjiang Huma
50247	Heilongjiang Huzhong
50983	Heilongjiang Hulin
50878	Heilongjiang Huachuan
50879	Heilongjiang Huanan
50987	Heilongjiang Jidong
50978	Heilongjiang Jixi
50442	Heilongjiang Jiagedaqi
50873	Heilongjiang Jiamusi
50673	Heilongjiang Jiayin
50659	Heilongjiang Kedong
50658	Heilongjiang Keshan
50859	Heilongjiang Lansi
50749	Heilongjiang Lindian

50979	Heilongjiang Linkou
50739	Heilongjiang Longjiang
50776	Heilongjiang Luobei
50985	Heilongjiang Mishan
50758	Heilongjiang Minshui
50136	Heilongjiang Mohe
54094	Heilongjiang Mudanjiang
50962	Heilongjiang Mulan
54093	Heilongjiang Muling
50646	Heilongjiang Nehe
50557	Heilongjiang Nenjiang
54098	Heilongjiang Ningan
50971	Heilongjiang Qitaihe
50745	Heilongjiang Qiqihaer
50851	Heilongjiang Qinggang
50861	Heilongjiang Qingan
50892	Heilongjiang Raohe
50968	Heilongjiang Shangzhi
50884	Heilongjiang Shuangyashan
50787	Heilongjiang Suibin
54096	Heilongjiang Suifenhe
50767	Heilongjiang Suileng
50564	Heilongjiang Sunwu
50246	Heilongjiang Tahe
50844	Heilongjiang Tailai
50871	Heilongjiang Tangyuan
50862	Heilongjiang Tieli
50963	Heilongjiang Tonghe
50778	Heilongjiang Tongjiang
50852	Heilongjiang Wangkui
50674	Heilongjiang Wuyiling
54080	Heilongjiang Wuchang
50655	Heilongjiang Wudalianchi
50772	Heilongjiang Wuying
50349	Heilongjiang Xinlin
50566	Heilongjiang Xunke
50965	Heilongjiang Yanshou
50774	Heilongjiang Yichun
50750	Heilongjiang Yian
50877	Heilongjiang Yilang
50858	Heilongjiang Zhaodong
50950	Heilongjiang Zhaozhou
57388	Hubei Anlu

57355	Hubei Padang
57361	Hubei Baokang
57489	Hubei Caidian
57582	Hubei Chibi
57586	Hubei Chongyang
57260	Hubei Danjiangkou
57496	Hubei Ezhou
57447	Hubei Enshi
57259	Hubei Fangxian
57477	Hubei Gongan
57268	Hubei Gucheng
57486	Hubei Hanchuan
57543	Hubei Hefeng
57398	Hubei Hongan
57581	Hubei Honghu
58409	Hubei Huangmei
57583	Hubei Jiayu
57573	Hubei Jianli
57445	Hubei Jianshi
57387	Hubei Jingshan
57377	Hubei Jingmen
57439	Hubei Lichuan
58401	Hubei Luotian
57399	Hubei Macheng
57363	Hubei Nanzhang
58408	Hubei Qichun
57475	Hubei Qianjiang
57484	Hubei Shayang
57362	Hubei Shennongjia
57256	Hubei Shiyan
57571	Hubei Shishou
57469	Hubei Songzi
57381	Hubei Suizhou
57483	Hubei Tianmen
57589	Hubei Tongcheng
57495	Hubei Tuanfeng
57458	Hubei Wufeng
57494	Hubei Wuhan
58501	Hubei Wuxue
57485	Hubei Xiantao
57540	Hubei Xianfeng
57590	Hubei Xianning
57278	Hubei Xiangyang

57386	Hubei Xiaochang
57482	Hubei Xiaogan
57492	Hubei Xinzhou
57359	Hubei Xingshan
57541	Hubei Xuanen
58500	Hubei Yangxin
57453	Hubei Yiling
57370	Hubei Yicheng
57481	Hubei Yingcheng
58402	Hubei Yingshan
57251	Hubei Yunxi
57253	Hubei Yunxian
57279	Hubei Zaoyang
57378	Hubei Zhongxiang
57257	Hubei Zhushan
57249	Hubei Zhuxi
57669	Hunan Anhua
57881	Hunan Anren
57642	Hunan Baojing
57882	Hunan Chaling
57687	Hunan Changsha
57662	Hunan Changde
57874	Hunan Changning
57972	Hunan Chenzhou
57965	Hunan Daoxian
57867	Hunan Dongan
57740	Hunan Fenghuang
57646	Hunan Guzhang
57889	Hunan Guangxi
57973	Hunan Guiyang
57663	Hunan Hanshou
57875	Hunan Hengnan
57777	Hunan Hengshan
57872	Hunan Hengyang
57871	Hunan Hengyangxian
57754	Hunan Hongjiang
57575	Hunan Huarong
57649	Hunan Jishou
57974	Hunan Jiahe
59063	Hunan Jianghua
57969	Hunan Jiangyong
57975	Hunan Lanshan
57760	Hunan Lengshuijiang

57865	Hunan Lengshuitan
57565	Hunan Lixian
57781	Hunan Liling
57978	Hunan Linwu
57688	Hunan Liuyang
57544	Hunan Longshan
57763	Hunan Loudi
57657	Hunan Luxi
57680	Hunan Milo
57574	Hunan Nanxian
57776	Hunan Nanyue
57678	Hunan Ningxiang
57966	Hunan Ningyuan
57682	Hunan Pingjiang
57870	Hunan Qidong
57868	Hunan Qiyang
57985	Hunan Rucheng
57554	Hunan Sangzhi
57771	Hunan Shaoshan
57774	Hunan Shuangfeng
57962	Hunan Shuangpai
57666	Hunan Taojiang
57661	Hunan Taoyuan
57845	Hunan Tongtao
57772	Hunan Xiangxiang
57673	Hunan Xiangyin
57761	Hunan Xinhua
57744	Hunan Xinhuang
57971	Hunan Xintian
57752	Hunan Xupu
57976	Hunan Yizhang
57643	Hunan Yongshun
57887	Hunan Yongxing
57866	Hunan Yongzhou
57779	Hunan Youxian
57671	Hunan Yuanjiang
57655	Hunan Yuanling
57584	Hunan Yueyang
57558	Hunan Zhangjiajie
57745	Hunan Zhijiang
57780	Hunan Zhuzhou
57981	Hunan Zixing
54187	Jilin Antu

50936	Jilin Baicheng
54371	Jilin Baishan
54386	Jilin Changbai
54161	Jilin Changchun
54049	Jilin Changling
50945	Jilin Daan
54065	Jilin Dehui
54261	Jilin Tongfeng
54284	Jilin Donggang
54186	Jilin Dunhua
54285	Jilin Erdao
54063	Jilin Fuyu
54155	Jilin Gujiazi
54286	Jilin Helong
54273	Jilin Huadian
54291	Jilin Huichun
54274	Jilin Huinan
54172	Jilin Jilin Suburb
54377	Jilin Jian
54279	Jilin Jiangyuan
54181	Jilin Jiaohe
54069	Jilin Jiutai
54154	Jilin Lishu
54260	Jilin Liaoyuan
54374	Jilin Linjiang
54267	Jilin Liuhe
54290	Jilin Longjing
54192	Jilin Luozigou
54064	Jilin Nongan
54263	Jilin Panshi
50949	Jilin Qianguo
50948	Jilin Qianan
54076	Jilin Shulan
54142	Jilin Shuangliao
54165	Jilin Shuangyang
50946	Jilin Songyuan
50939	Jilin Taonan
54363	Jilin Tonghua
54362	Jilin Tonghuaxian
54041	Jilin Tongyu
54195	Jilin Wangqing
54169	Jilin Yantongshan
54292	Jilin Yanji

54164	Jilin Yitong
54171	Jilin Yongji
54072	Jilin Yushu
50940	Jilin Zhenlai
58148	Jiangsu Baoying
58352	Jiangsu Changshu
58158	Jiangsu Dafeng
58341	Jiangsu Danyang
58036	Jiangsu Donghai
58251	Jiangsu Dongtai
58012	Jiangsu Fengxian
58143	Jiangsu Funing
58040	Jiangsu Ganyu
58339	Jiangsu Gaochun
58241	Jiangsu Gaoyou
58047	Jiangsu Guanyun
58254	Jiangsu Haian
58360	Jiangsu Haimen
58139	Jiangsu Hongze
58141	Jiangsu Huaian
58146	Jiangsu Jianhu
58250	Jiangsu Jiangyan
58147	Jiangsu Jinhu
58342	Jiangsu Jintan
58257	Jiangsu Jingjiang
58344	Jiangsu Jurong
58356	Jiangsu Kunshan
58340	Jiangsu Lishui
58044	Jiangsu Lianyungang
58140	Jiangsu Lianshui
58235	Jiangsu Liuhe
58265	Jiangsu Lvsi
58238	Jiangsu Nanjing
58259	Jiangsu Nantong
58013	Jiangsu Peixian
58026	Jiangsu Pizhou
58237	Jiangsu Pukou
58269	Jiangsu Qidong
58264	Jiangsu Rudong
58255	Jiangsu Rugao
58038	Jiangsu Shuyang
58135	Jiangsu Sihong
58132	Jiangsu Siyang

58349	Jiangsu Suzhou
58131	Jiangsu Suyu
58130	Jiangsu Suining
58377	Jiangsu Taicang
58249	Jiangsu Taixing
58246	Jiangsu Taizhou
58354	Jiangsu Wuxi
58041	Jiangsu Xiliandao
58045	Jiangsu Xiangshui
58243	Jiangsu Xinghua
58138	Jiangsu Xuyi
58027	Jiangsu Xuzhou
58154	Jiangsu Yancheng
58247	Jiangsu Yangzhong
58242	Jiangsu Yizheng
58346	Jiangsu Yixing
58353	Jiangsu Zhangjiagang
57798	Jiangxi Anfu
58602	Jiangxi Anyi
58907	Jiangxi Anyuan
58519	Jiangxi Poyang
58710	Jiangxi Chongren
57990	Jiangxi Chongyi
58508	Jiangxi Dean
58622	Jiangxi Dexing
59093	Jiangxi Dingnan
58618	Jiangxi Dongxiang
58517	Jiangxi Duchang
57792	Jiangxi Fenyi
58601	Jiangxi Fengxin
57993	Jiangxi Ganxian
58605	Jiangxi Gaoan
58813	Jiangxi Guangchang
58626	Jiangxi Guixi
58510	Jiangxi Hukou
58906	Jiangxi Huichang
57799	Jiangxi Jianxian
58712	Jiangxi Jinxi
58614	Jiangxi Jinxian
58527	Jiangxi Jingdezhen
58600	Jiangxi Jingan
58502	Jiangxi Jiujiang
58706	Jiangxi Lean

58719	Jiangxi Lichuan
57789	Jiangxi Lianhua
58619	Jiangxi Linchuan
59092	Jiangxi Longnan
58506	Jiangxi Lushan
58606	Jiangxi Nanchang
58715	Jiangxi Nancheng
58718	Jiangxi Nanfeng
57992	Jiangxi Nankang
58806	Jiangxi Ningdu
58512	Jiangxi Pengze
57786	Jiangxi Pingxiang
58629	Jiangxi Qianshan
59091	Jiangxi Quannan
58503	Jiangxi Ruichang
57699	Jiangxi Shanggao
57783	Jiangxi Shangli
58623	Jiangxi Shangraoxian
58814	Jiangxi Shicheng
57896	Jiangxi Suichuan
57899	Jiangxi Taihe
57694	Jiangxi Tonggu
57895	Jiangxi Wanan
58615	Jiangxi Wannian
57698	Jiangxi Wanzai
58507	Jiangxi Wuning
58529	Jiangxi Wuyuan
58704	Jiangxi Xiajiang
57883	Jiangxi Xiaping
58693	Jiangxi Xinjian
57796	Jiangxi Xinyu
57995	Jiangxi Xinfeng
58804	Jiangxi Xingguo
57598	Jiangxi Xiushui
59102	Jiangxi Xunwu
57793	Jiangxi Yichun
58705	Jiangxi Yongfeng
57891	Jiangxi Yongxin
58905	Jiangxi Yudu
58612	Jiangxi Yugan
58634	Jiangxi Yushan
54339	Liaoning Anshan
54346	Liaoning Benxi

54483	Liaoning Caohekou
54243	Liaoning Changtu
54579	Liaoning Changhai
54565	Liaoning Changxingdao
54324	Liaoning Chaoyang
54475	Liaoning Dashiqiao
54497	Liaoning Dandong
54494	Liaoning Fengcheng
54351	Liaoning Fushun
54474	Liaoning Gaizhou
54452	Liaoning Jianchang
54326	Liaoning Jianpingxian
54321	Liaoning Jianpingzhen
54568	Liaoning Jinzhou
54244	Liaoning Kangping
54493	Liaoning Kuandian
54453	Liaoning Lianshan
54345	Liaoning Liaoyangxian
54332	Liaoning Liaozhong
54327	Liaoning Lingyuan
54338	Liaoning Panshan
54575	Liaoning Pikou
54569	Liaoning Pulandian
54259	Liaoning Qingyuan
54248	Liaoning Shenbei
54342	Liaoning Shenyang
54340	Liaoning Sujiatun
54454	Liaoning Suizhong
54336	Liaoning Taian
54249	Liaoning Tieling
54563	Liaoning Wafangdian
54252	Liaoning Xifeng
54353	Liaoning Xinbin
54333	Liaoning Xinmin
54455	Liaoning Xingcheng
54486	Liaoning Xiuyan
54325	Liaoning Yangshan
54471	Liaoning Yingkou
54236	Liaoning Zhangwu
54584	Liaoning Zhuanghe
53192	Inner Mongolia Abagaqi
50727	Inner Mongolia Aershan
54122	Inner Mongolia Alukeerqinqi

50647	Inner Mongolia Arunqi
54225	Inner Mongolia Aohanqi
54316	Inner Mongolia Balihan
54113	Inner Mongolia Balinyouqi
54027	Inner Mongolia Balinzuoqi
50928	Inner Mongolia Bayaertuhushuo
53446	Inner Mongolia Baotou
54226	Inner Mongolia Baoguotu
53384	Inner Mongolia Chayouhouqi
53481	Inner Mongolia Chayouqianqi
53378	Inner Mongolia Chayouzhongqi
54218	Inner Mongolia Chifeng
53457	Inner Mongolia Dalateqi
53352	Inner Mongolia Damaoqi
53348	Inner Mongolia Dashetai
53419	Inner Mongolia Dengkou
53543	Inner Mongolia Dongsheng
50915	Inner Mongolia Wuzhumuqindong
54208	Inner Mongolia Duolunxian
50425	Inner Mongolia Eerguna
50445	Inner Mongolia Elunchunqi
53529	Inner Mongolia Etukeqi
53730	Inner Mongolia Eduokeqianqi
50525	Inner Mongolia Evenkeqi
53068	Inner Mongolia Erlianhaote
54024	Inner Mongolia Fuhe
54214	Inner Mongolia Gangzi
54031	Inner Mongolia Gaoliban
53357	Inner Mongolia Guyangxian
53231	Inner Mongolia Hailisu
53420	Inner Mongolia Hangjinhouqi
53533	Inner Mongolia Hangjinqi
53469	Inner Mongolia Helingeerxian
53732	Inner Mongolia Henan
53463	Inner Mongolia Huhehaote
53466	Inner Mongolia Huhehaote suburb
53391	Inner Mongolia Huade
50924	Inner Mongolia Houlinguole
53480	Inner Mongolia Jining
54313	Inner Mongolia Kalaqinqi
54134	Inner Mongolia Kailu
54231	Inner Mongolia Kezuohouqi
54047	Inner Mongolia Kezuozhongqi

54117	Inner Mongolia Keshiketengqi
54234	Inner Mongolia Kulun
53475	Inner Mongolia Liangcheng
54115	Inner Mongolia Linxixian
53513	Inner Mongolia Linhe
53149	Inner Mongolia Mandula
50514	Inner Mongolia Manzhouli
50645	Inner Mongolia Moulidawawoer
53083	Inner Mongolia Narenbaolige
54223	Inner Mongolia Neiman
54320	Inner Mongolia Ningchengxian
54132	Inner Mongolia Qinglongshan
53562	Inner Mongolia Qingshuihexian
53385	Inner Mongolia Shangdu
54039	Inner Mongolia Shebotu
53362	Inner Mongolia Siziwang
50834	Inner Mongolia Suolun
54305	Inner Mongolia Taibushiqi
54135	Inner Mongolia Tongliao
50934	Inner Mongolia Tuquan
50434	Inner Mongolia Tulihe
53464	Inner Mongolia Tumutezuoqi
53455	Inner Mongolia Tuyouqi
53467	Inner Mongolia Tuoketuoxian
54213	Inner Mongolia Wengniuteqi
53512	Inner Mongolia Wuhai
50913	Inner Mongolia Wulagai
53433	Inner Mongolia Wulateqianqi
53336	Inner Mongolia Wulatezhongqi
53644	Inner Mongolia Wushenqi
53337	Inner Mongolia Wuyuan
53368	Inner Mongolia Wuchuanxian
54012	Inner Mongolia Xiwuzhumuqin
53367	Inner Mongolia Xilamuren
54102	Inner Mongolia Xilinhaote
53289	Inner Mongolia Xianghuangqi
50548	Inner Mongolia Xiaoergou
50603	Inner Mongolia Xinbaerhuyouqi
50618	Inner Mongolia Xinbaerhuzuoqi
53483	Inner Mongolia Xinghe
50526	Inner Mongolia Yakeshi
53545	Inner Mongolia Ejinhuoluoqi
53522	Inner Mongolia Yikewusu

50639	Inner Mongolia Zhalantun
54026	Inner Mongolia Zhalute
54205	Inner Mongolia Zhenglanqi
54204	Inner Mongolia Zhengxiangbaiqi
53553	Inner Mongolia Zhungeerqi
53472	Inner Mongolia Zhuozi
53817	Ningxia Guyuan
53806	Ningxia Haiyuan
53610	Ningxia Helan
53519	Ningxia Huinong
53910	Ningxia Liupanshan
53727	Ningxia Maihuangshan
53611	Ningxia Pingluo
53517	Ningxia Shitanjing
53615	Ningxia Taole
53810	Ningxia Tongxin
53881	Ningxia Weizhou
53612	Ningxia Wuzhong
53903	Ningxia Xiji
53707	Ningxia Xingren
53723	Ningxia Yanchi
53618	Ningxia Yongning
53705	Ningxia Zhongning
53704	Ningxia Zhongwei
56151	Qinghai Banma
56046	Qinghai Dari
52836	Qinghai Doulan
56045	Qinghai Gander
52856	Qinghai Gonghe
52868	Qinghai Guide
52955	Qinghai Guinan
56065	Qinghai Henan
52863	Qinghai Huzhu
52877	Qinghai Hualong
52963	Qinghai Jianzha
56067	Qinghai Jiuzhi
56033	Qinghai Maduo
56043	Qinghai Maqin
52876	Qinghai Minhe
56125	Qinghai Nangqian
52825	Qinghai Nuomuhong
52875	Qinghai Pingan
52854	Qinghai Qinghaihu 151

56034	Qinghai Qingshuihe
56021	Qinghai Qumacai
52941	Qinghai Shazhuyu
52957	Qinghai Tongde
52974	Qinghai Tongren
56004	Qinghai Tuotuohe
52908	Qinghai Wudaoliang
52866	Qinghai Xining
52943	Qinghai Xinghai
52972	Qinghai Xunhua
56029	Qinghai Yushu
56018	Qinghai Zaduo
52968	Qinghai Zeku
56016	Qinghai Zhiduo
54844	Shandong Anqiu
54734	Shandong Binzhou
58030	Shandong Cangshan
58002	Shandong Caoxian
54841	Shandong Changyi
54751	Shandong Longdao
54776	Shandong Chengshantou
58003	Shandong Chengwu
54814	Shandong Chiping
54909	Shandong Dingtao
54815	Shandong Donge
54911	Shandong Dongping
54819	Shandong Feicheng
54929	Shandong Feixian
54764	Shandong Fushan
54846	Shandong Gaomi
54729	Shandong Gaoqing
54906	Shandong Heze
54833	Shandong Huantai
54943	Shandong Huangdao
54855	Shandong Jimo
54823	Shandong Jinan
54821	Shandong Jiyang
54849	Shandong Jiaozhou
54939	Shandong Junan
54914	Shandong Juye
54904	Shandong Juancheng
54744	Shandong Kenli
54828	Shandong Laiwu

54852	Shandong Laiyang
54749	Shandong Laizhou
54726	Shandong Leling
54910	Shandong Liangshan
54806	Shandong Liaocheng
54802	Shandong Linqing
58032	Shandong Linshu
54938	Shandong Linyi
54712	Shandong Linyi
54753	Shandong Longkou
54923	Shandong Mengyin
54716	Shandong Ningjin
54752	Shandong Penglai
54842	Shandong Pingdu
54925	Shandong Pingyi
54759	Shandong Qixia
54812	Shandong Qihe
54831	Shandong Qingzhou
54945	Shandong Rizhao
54861	Shandong Rushan
54724	Shandong Shanghe
54832	Shandong Shouguang
54920	Shandong Sishui
54827	Shandong Taian
54927	Shandong Tengzhou
54843	Shandong Weifang
54777	Shandong Wendeng
54912	Shandong Wenshang
54940	Shandong Wulian
54709	Shandong Wucheng
54808	Shandong Xinxian
54922	Shandong Xintai
58021	Shandong Xuechen
54765	Shandong Yantai
54916	Shandong Yanzhou
54723	Shandong Yangxin
54932	Shandong Yishui
54836	Shandong Yiyuan
58022	Shandong Yicheng
54907	Shandong Yutai
54905	Shandong Yuncheng
58024	Shandong Zaozhuang
54727	Shandong Zhangqiu

54755	Shandong Zhaoyuan
54848	Shandong Zhucheng
54830	Shandong Zibo
54919	Shandong Zoucheng
54822	Shandong Zouping
53877	Shanxi Anze
53873	Shanxi Changzi
53487	Shanxi Datong
53579	Shanxi Daixian
53676	Shanxi Dingxiang
53585	Shanxi Fanshi
53760	Shanxi Fangshan
53865	Shanxi Fenxi
53966	Shanxi Fushan
53973	Shanxi Gaoping
53763	Shanxi Gujiao
53874	Shanxi Guxian
53590	Shanxi Guangling
53788	Shanxi Heshun
53957	Shanxi Hejin
53564	Shanxi Hequ
53866	Shanxi Hongtong
53582	Shanxi Hunyuan
53859	Shanxi Jixian
53954	Shanxi Jishan
53677	Shanxi Jiancaoping
53965	Shanxi Jiangxian
53860	Shanxi Jiaokou
53863	Shanxi Jiexiu
53666	Shanxi Jingle
53662	Shanxi Kelan
53665	Shanxi Lanxian
53764	Shanxi Lishi
53878	Shanxi Licheng
53659	Shanxi Linxian
53862	Shanxi Lingshi
53981	Shanxi Lingchuan
53753	Shanxi Liulin
53880	Shanxi Lucheng
53577	Shanxi Ningwu
53565	Shanxi Pianguan
53687	Shanxi Pingding
53574	Shanxi Pinglu

53778	Shanxi Pingyao
53864	Shanxi Puxian
53970	Shanxi Qingshui
53872	Shanxi Qinxian
53875	Shanxi Qinyuan
53774	Shanxi Qingxu
57053	Shanxi Ruicheng
53576	Shanxi Shanyin
53575	Shanxi Shenchi
53759	Shanxi Shilou
53780	Shanxi Shouyang
53578	Shanxi Shuozhou
53775	Shanxi Taigu
53956	Shanxi Wanrong
53588	Shanxi Wutaishan
53681	Shanxi Wutaixian
53663	Shanxi Wuzhai
53871	Shanxi Wuxiang
53853	Shanxi Xixian
53953	Shanxi Xiangning
53861	Shanxi Xiangfen
53679	Shanxi Xiaodian
53768	Shanxi Xiaoyi
53674	Shanxi Xinfu
53964	Shanxi Xinjiang
53664	Shanxi Xingxian
53959	Shanxi Yanhu
53486	Shanxi Yanggao
53782	Shanxi Yangquan
53584	Shanxi Yingxian
53852	Shanxi Yonghe
57052	Shanxi Yongji
53478	Shanxi Youyu
53685	Shanxi Yuxian
53787	Shanxi Yushe
53968	Shanxi Yuanqu
53673	Shanxi Yuanping
53767	Shanxi Zhongyang
53786	Shanxi Zuoquan
57245	Shaanxi Ankang
53841	Shaanxi Ansai
57254	Shaanxi Baihe
53941	Shaanxi Baishui

57016	Shaanxi Baoji
57128	Shaanxi Chenggu
53949	Shaanxi Chengcheng
57043	Shaanxi Dali
57153	Shaanxi Danfeng
53725	Shaanxi Dingbian
57113	Shaanxi Fengxian
57134	Shaanxi Foping
57026	Shaanxi Fufeng
53567	Shaanxi Fugu
57042	Shaanxi Fuping
53931	Shaanxi Fuxian
53848	Shaanxi Ganquan
57233	Shaanxi Hanyin
53950	Shaanxi Heyang
53740	Shaanxi Hengshan
57046	Shaanxi Huashan
53944	Shaanxi Huangling
53946	Shaanxi Huanglong
53658	Shaanxi Jiaxian
53735	Shaanxi Jingbian
57247	Shaanxi Langao
57029	Shaanxi Liquan
57022	Shaanxi Linyou
57124	Shaanxi Liuba
57003	Shaanxi Longxian
57106	Shaanxi Lueyang
57057	Shaanxi Luonan
57027	Shaanxi Meixian
57119	Shaanxi Mianxian
57213	Shaanxi Nanzheng
57211	Shaanxi Ningqiang
57137	Shaanxi Ningshan
57248	Shaanxi Pingli
53948	Shaanxi Pucheng
57021	Shaanxi Qianyang
53757	Shaanxi Qingjian
57041	Shaanxi Sanyuan
57155	Shaanxi Shanyang
57154	Shaanxi Shangnan
57143	Shaanxi Shangxian
53651	Shaanxi Shenmu
53754	Shaanxi Suide

53947	Shaanxi Tongchuan
57045	Shaanxi Weinan
53756	Shaanxi Wubao
53738	Shaanxi Wuqi
57034	Shaanxi Wugong
57038	Shaanxi Xingping
57242	Shaanxi Xunyang
53938	Shaanxi Xunyi
53854	Shaanxi Yanchang
53850	Shaanxi Yanchuan
57037	Shaanxi Yaoxian
53857	Shaanxi Yichuan
57030	Shaanxi Yongshou
53646	Shaanxi Yulin
57238	Shaanxi Zhenba
57343	Shaanxi Zhenping
53832	Shaanxi Zhidan
53748	Shaanxi Zichang
57231	Shaanxi Ziyang
57140	Shaanxi Zhashui
58362	Shanghai Baoshan
58463	Shanghai Fengxian
58460	Shanghai Jinshan
58361	Shanghai Minhang
56171	Sichuan Aba
56247	Sichuan Batang
57313	Sichuan Bazhong
56147	Sichuan Baiyu
56273	Sichuan Baoxing
56580	Sichuan Butuo
56593	Sichuan Changning
56181	Sichuan Chongzhou
57328	Sichuan Dachuan
57420	Sichuan Dazhu
56263	Sichuan Danba
56167	Sichuan Daofu
56357	Sichuan Daocheng
56441	Sichuan Derong
56569	Sichuan Dechang
56144	Sichuan Dege
56198	Sichuan Deyang
57503	Sichuan Dongxing
56188	Sichuan Dujiangyan

56387	Sichuan Ebian
56385	Sichuan Emeishan
56399	Sichuan Fushun
56473	Sichuan Ganluo
56146	Sichuan Ganzi
57411	Sichuan Gaoping
56499	Sichuan Gongxian
57415	Sichuan Guangan
57206	Sichuan Guangyuan
56376	Sichuan Hanyuan
57603	Sichuan Hejiang
56185	Sichuan Heishui
56173	Sichuan Hongyuan
56380	Sichuan Hongya
56675	Sichuan Huidong
56671	Sichuan Huili
56382	Sichuan Jiajiang
56389	Sichuan Qianwei
57208	Sichuan Jiange
57600	Sichuan Jiangan
56168	Sichuan Jinchuan
56296	Sichuan Jintang
56584	Sichuan Jinyang
56390	Sichuan Jingyan
56462	Sichuan Jiulong
56097	Sichuan Jiuzhaigou
57329	Sichuan Kaijiang
56374	Sichuan Kangding
57306	Sichuan Langzhong
56485	Sichuan Leibo
56257	Sichuan Litang
56184	Sichuan Lixian
57416	Sichuan Linshui
56286	Sichuan Longquanyi
57507	Sichuan Longchang
56279	Sichuan Lushan
56371	Sichuan Luding
56158	Sichuan Luhuo
56480	Sichuan Mabian
56172	Sichuan Maerkang
56180	Sichuan Maoxian
56487	Sichuan Meigu
56670	Sichuan Miyi

56186	Sichuan Mianzhu
56474	Sichuan Mianning
56280	Sichuan Mingshan
56459	Sichuan Muli
56490	Sichuan Muchuan
57314	Sichuan Nanbu
57216	Sichuan Nanjiang
56493	Sichuan Nanxi
56666	Sichuan Panzhihua
56289	Sichuan Pengshan
56189	Sichuan Pengzhou
57324	Sichuan Pingchang
56494	Sichuan Pingshan
56281	Sichuan Pujiang
56575	Sichuan Puge
57204	Sichuan Qingchuan
56383	Sichuan Qingshen
57413	Sichuan Quxian
56164	Sichuan Rangtang
56297	Sichuan Renshou
56079	Sichuan Ruoergai
56152	Sichuan Seda
57401	Sichuan Shehong
56378	Sichuan Shimian
56038	Sichuan Shiqu
56182	Sichuan Songpan
57405	Sichuan Suining
57320	Sichuan Tongjiang
57237	Sichuan Wanyuan
57217	Sichuan Wangcang
56395	Sichuan Weiyuan
56187	Sichuan Wenjiang
56183	Sichuan Wenchuan
57417	Sichuan Wusheng
56571	Sichuan Xichang
57309	Sichuan Xichong
56478	Sichuan Xide
56443	Sichuan Xiangcheng
56178	Sichuan Xiaojin
56251	Sichuan Xinlong
56496	Sichuan Xingwen
57608	Sichuan Xuyong
56267	Sichuan Yajiang

56665	Sichuan Yanbian
56565	Sichuan Yanyuan
57315	Sichuan Yilong
56491	Sichuan Yibinxian
56373	Sichuan Yingjing
57318	Sichuan Yingshan
56475	Sichuan Yuexi
56479	Sichuan Chaojue
56298	Sichuan Ziyang
56393	Sichuan Zizhong
56396	Sichuan Zigong
54525	Tianjin Baodi
54645	Tianjin Dagang
54622	Tianjin Jinnan
54619	Tianjin Jinghai
54623	Tianjin Tanggu
54523	Tianjin Wuqing
56228	Tibet Basu
56227	Tibet Bomi
56434	Tibet Chayu
56137	Tibet Changdu
55493	Tibet Dangxiong
56116	Tibet Dingqing
55664	Tibet Dingri
55248	Tibet Gaize
55680	Tibet Jiangzi
55569	Tibet Lazi
56128	Tibet Leiwuqi
56312	Tibet Linzhi
56223	Tibet Luolong
56317	Tibet Miling
55593	Tibet Muozhugongka
55299	Tibet Naqu
55572	Tibet Nanmulin
55655	Tibet Nielamu
55437	Tibet Pulan
55228	Tibet Shiquanhe
55598	Tibet Zedang
56331	Tibet Zuogong
51058	Xinjiang Akedala
51628	Xinjiang Akesu
51730	Xinjiang Alaer
51232	Xinjiang Alashankou

51076	Xinjiang Aletai
51704	Xinjiang Atushi
51722	Xinjiang Awati
52101	Xinjiang Balikun
51542	Xinjiang Bayinbuluke
51238	Xinjiang Bole
51060	Xinjiang Buerjin
51826	Xinjiang Cele
51368	Xinjiang Changji
51477	Xinjiang Dabancheng
51145	Xinjiang Emin
51068	Xinjiang Fuhai
51377	Xinjiang Fukang
51087	Xinjiang Fuyun
51707	Xinjiang Jiashi
51053	Xinjiang Habahe
52203	Xinjiang Hami
51156	Xinjiang Hebukesaier
51828	Xinjiang Hetan
52313	Xinjiang Hongliuhe
51367	Xinjiang Hutubi
51378	Xinjiang Jimusaer
51334	Xinjiang Jinghe
51709	Xinjiang Kashi
51720	Xinjiang Kepin
51243	Xinjiang Kelamayi
51656	Xinjiang Kuerle
51526	Xinjiang Kumishi
51829	Xinjiang Luopu
51359	Xinjiang Manasi
51810	Xinjiang Maigaiti
51369	Xinjiang Miquan
51839	Xinjiang Minfeng
51827	Xinjiang Moyu
51482	Xinjiang Mulei
52112	Xinjiang Naomaohu
51818	Xinjiang Pishan
51855	Xinjiang Qiemuo
51357	Xinjiang Shawan
51639	Xinjiang Shaya
51811	Xinjiang Shashe
51133	Xinjiang Tacheng
51747	Xinjiang Tazhong

51470	Xinjiang Tianchi
51468	Xinjiang Tianshandaxigou
51765	Xinjiang Tieqianlike
51573	Xinjiang Tulufan
51572	Xinjiang Tulufandongkan
51571	Xinjiang Tuokexun
51241	Xinjiang Touli
51469	Xinjiang Urumqi Pastoral Test Station
51705	Xinjiang Wuqia
51627	Xinjiang Wushi
51636	Xinjiang Xinhe
51567	Xinjiang Yanqi
51814	Xinjiang Yecheng
52118	Xinjiang Yiwu
51802	Xinjiang Yengjisha
51931	Xinjiang Yutian
51137	Xinjiang Yumin
51717	Xinjiang Yuepuhu
51815	Xinjiang Zepu
56863	Yunnan Anning
56748	Yunnan Baoshan
56752	Yunnan Binchuan
56944	Yunnan Cangyuan
56843	Yunnan Changning
56873	Yunnan Chengjiang
56582	Yunnan Daguan
56751	Yunnan Dali
56444	Yunnan Deqin
56688	Yunnan Dongchuan
56898	Yunnan Eshan
56649	Yunnan Eryuan
56846	Yunnan Fengqing
56641	Yunnan Fugong
56772	Yunnan Fumin
59205	Yunnan Funing
56790	Yunnan Fuyuan
56984	Yunnan Gejiu
56946	Yunnan Gengma
56533	Yunnan Gongshan
59007	Yunnan Guangnan
56654	Yunnan Heqing
56879	Yunnan Huaning
56664	Yunnan Huaping

56646	Yunnan Jianchuan
56977	Yunnan Jiangcheng
56987	Yunnan Jinping
56871	Yunnan Jinning
56856	Yunnan Jingdong
56952	Yunnan Jinggu
56982	Yunnan Kaiyuan
56778	Yunnan Kunming
56645	Yunnan Lanping
56954	Yunnan Lancang
56651	Yunnan Lijiang
56840	Yunnan Lianghe
56951	Yunnan Lincang
56643	Yunnan Liuku
56841	Yunnan Longling
56835	Yunnan Longchuan
56886	Yunnan Luxi
56585	Yunnan Ludian
56777	Yunnan Lufeng
56978	Yunnan Luchun
56891	Yunnan Luoping
56782	Yunnan Malong
56958	Yunnan Menghai
56969	Yunnan Mengla
56949	Yunnan Menglian
56755	Yunnan Midu
56885	Yunnan Mile
56962	Yunnan Mojiang
56766	Yunnan Mouding
56767	Yunnan Nanhua
56567	Yunnan Ninglang
56673	Yunnan Qiaojia
56889	Yunnan Qiubei
56783	Yunnan Qujing
56883	Yunnan Shizong
56842	Yunnan Shidian
56881	Yunnan Shilin
56970	Yunnan Shiping
56862	Yunnan Shuangbai
56950	Yunnan Shuangjiang
56964	Yunnan Simao
56785	Yunnan Songming
56483	Yunnan Suijiang

56739	Yunnan Tengchong
56596	Yunnan Weixin
56757	Yunnan Weishan
56548	Yunnan Weixi
56994	Yunnan Wenshan
56774	Yunnan Wuding
56992	Yunnan Xichou
56948	Yunnan Ximeng
56543	Yunnan Xianggelila
56869	Yunnan Xinping
56697	Yunnan Xuanwei
56497	Yunnan Yanjin
56991	Yunnan Yanshan
56745	Yunnan Yangbi
56764	Yunnan Yaoan
56880	Yunnan Yiliang
56594	Yunnan Yiliang
56870	Yunnan Yimen
56836	Yunnan Yingjiang
56849	Yunnan Yongde
56746	Yunnan Yongping
56669	Yunnan Yongren
56652	Yunnan Yongsheng
56875	Yunnan Yuxi
56966	Yunnan Yuanjiang
56976	Yunnan Yuanyang
56742	Yunnan Yunlong
56854	Yunnan Yunxian
56839	Yunnan Zhenkang
56595	Yunnan Zhenxiong
56867	Yunnan Zhenyuan
58446	Zhejiang Anji
58443	Zhejiang Changxing
58631	Zhejiang Changshan
58467	Zhejiang Cixi
58666	Zhejiang Dachen
58484	Zhejiang Daishan
58454	Zhejiang Deqing
58558	Zhejiang Dongyang
58565	Zhejiang Fenghua
58449	Zhejiang Fuyang
58457	Zhejiang Hangzhou
58665	Zhejiang Hongjia

58450	Zhejiang Huzhou
58451	Zhejiang Jiashan
58452	Zhejiang Jiaxing
58544	Zhejiang Jiande
58632	Zhejiang Jiangshan
58549	Zhejiang Jinhua
58654	Zhejiang Jinyun
58648	Zhejiang Jingning
58537	Zhejiang Kaihua
58656	Zhejiang Leqing
58646	Zhejiang Lishui
58448	Zhejiang Linan
58660	Zhejiang Linhai
58647	Zhejiang Longquan
58547	Zhejiang Longyou
58567	Zhejiang Ninghai
58560	Zhejiang Panan
58751	Zhejiang Pingyang
58546	Zhejiang Pujiang
58570	Zhejiang Putuo
58657	Zhejiang Qingtian
58568	Zhejiang Sanmen
58453	Zhejiang Shaoxing
58472	Zhejiang Shengsi
58556	Zhejiang Shengzhou
58569	Zhejiang Shipu
58644	Zhejiang Suichang
58746	Zhejiang Taishun
58559	Zhejiang Tiantai
58542	Zhejiang Tonglu
58664	Zhejiang Wenling
58750	Zhejiang Wencheng
58642	Zhejiang Wuyi
58652	Zhejiang Xianju
58566	Zhejiang Xiangshan
58555	Zhejiang Xinchang
58562	Zhejiang Yinzhou
58658	Zhejiang Yongjia
58643	Zhejiang Yongkang
58742	Zhejiang Yunhe
58561	Zhejiang Zhenhai
58550	Zhejiang Zhuji
57518	Chongqing Banan

57511	Chongqing Beibei
57514	Chongqing Bishan
57520	Chongqing Changshou
57333	Chongqing Chengkou
57502	Chongqing Dazu
57425	Chongqing Dianjiang
57523	Chongqing Fengdu
57517	Chongqing Jiangjin
57338	Chongqing Kaixian
57519	Chongqing Nanchuan
57537	Chongqing Pengshui
57612	Chongqing Qijiang
57536	Chongqing Qianjiang
57505	Chongqing Rongchang
57438	Chongqing Shizhu
57510	Chongqing Tongliang
57409	Chongqing Tongnan
57509	Chongqing Wansheng
57432	Chongqing Wanzhou
57349	Chongqing Wushan
57345	Chongqing Wuxi
57525	Chongqing Wulong
57506	Chongqing Yongchuan
57513	Chongqing Yubei
57339	Chongqing Yunyang
57437	Chongqing Zhongxian

Table S7. 1744 monitoring stations whose PM2.5 emissions are from transportation sector

58015	Anhui Dangshan
58016	Anhui Xiaoxian
58102	Anhui Bozhou
58108	Anhui Jieshou
58109	Anhui Taihe
58112	Anhui Tianzhushan
58113	Anhui Suixi
58114	Anhui Woyang
58117	Anhui Leysin
58118	Anhui Mengcheng
58122	Anhui Suzhou
58125	Anhui Lingbi
58126	Anhui Sixian
58127	Anhui Huaiyuan
58128	Anhui Guzhen
58129	Anhui Wuhe
58202	Anhui Funan
58203	Anhui Fuyang
58210	Anhui Yingshang
58212	Anhui Fengtai
58214	Anhui Huoqiu
58220	Anhui Changfeng
58222	Anhui Fengyang
58223	Anhui Mingguang
58225	Anhui Dingyuan
58234	Anhui Laian
58236	Anhui Chuzhou
58240	Anhui Tianchang
58306	Anhui Jinzhai
58311	Anhui Luan
58314	Anhui Huoshan
58316	Anhui Shucheng
58317	Anhui Yuexi
58319	Anhui Tongcheng
58320	Anhui Feixi
58323	Anhui Feidong
58326	Anhui Chaohu
58327	Anhui Lujiang
58329	Anhui Wuwei
58330	Anhui Hanshan

58334	Anhui Wuhu
58336	Anhui Maanshan
58338	Anhui Wuhuxian
58417	Anhui Susong
58429	Anhui Tongling
58431	Anhui Nanling
58432	Anhui Jingxian
58433	Anhui Xuancheng
58435	Anhui Jingde
58436	Anhui Ningguo
58437	Anhui Huangshan
58441	Anhui Guangde
58442	Anhui Langxi
58520	Anhui Qimen
58523	Anhui Yixian
58530	Anhui Shexian
58531	Anhui Tunxi
54398	Beijing Shunyi
54399	Beijing Haidian
54406	Beijing Yanqing
54416	Beijing Miyun
54421	Beijing Miyunshangdianzi
54424	Beijing Pinggu
54431	Beijing Tongzhou
54499	Beijing Changping
54501	Beijing Zhaitang
54511	Beijing
54597	Beijing Xiayunling
58724	Fujian Guangze
58725	Fujian Shaowu
58730	Fujian Wuyishan
58731	Fujian Pucheng
58734	Fujian Jianyang
58735	Fujian Songxi
58736	Fujian Zhenghe
58744	Fujian Shouning
58747	Fujian Zhouning
58748	Fujian Fuan
58749	Fujian Zherong
58754	Fujian Fuding
58818	Fujian Ninghuai
58820	Fujian Taining
58822	Fujian Jianning

58823	Fujian Shunchang
58824	Fujian Mingxi
58828	Fujian Sanming
58836	Fujian Gutian
58837	Fujian Youxi
58839	Fujian Shuqing
58843	Fujian Xiapu
58844	Fujian Minhou
58846	Fujian Ningde
58848	Fujian Lianjiang
58911	Fujian Changting
58917	Fujian Wuping
58923	Fujian Datian
58926	Fujian Zhangping
58928	Fujian Huaan
58929	Fujian Anxi
58931	Fujian Jiuxianshan
58932	Fujian Yongtai
58938	Fujian Xiuyu
58941	Fujian Changle
58942	Fujian Fuqing
58944	Fujian Pingtan
58946	Fujian Putian
59113	Fujian Yongding
59122	Fujian Changtai
59124	Fujian Nanjing
59125	Fujian Pinghe
59129	Fujian Zhangpu
59130	Fujian Tongan
59131	Fujian Nanan
59133	Fujian Chongwu
59320	Fujian Zhaoan
59322	Fujian Yunxiao
52323	Gansu Mazongshan
52515	Gansu Subei
52533	Gansu Jiuquan
52546	Gansu Gaitai
52557	Gansu Linze
52643	Gansu Sunan
52656	Gansu Minle
52674	Gansu Yongchang
52679	Gansu Wuwei
52681	Gansu Minqin

52784	Gansu Gulang
52787	Gansu Wushaoling
52797	Gansu Jingtai
52881	Gansu Tianzhu
52884	Gansu Gaolan
52885	Gansu Yongdeng
52895	Gansu Jingyuan
52896	Gansu Baiyin
52978	Gansu Xiahe
52980	Gansu Yongjing
52981	Gansu Dongxiang
52982	Gansu Guanghe
52983	Gansu Yuzhong
52985	Gansu Hezheng
52986	Gansu Lintao
52988	Gansu Kangle
52993	Gansu Huining
52995	Gansu Anding
52996	Gansu Huajialing
52998	Gansu Weiyuan
53821	Gansu Huanxian
53829	Gansu Qingcheng
53906	Gansu Jingning
53915	Gansu Kongtong
53917	Gansu Zhuanglang
53923	Gansu Xifeng
53924	Gansu Lingtai
53925	Gansu Zhenyuan
53926	Gansu Jingchuan
53927	Gansu Huating
53930	Gansu Huachi
53934	Gansu Huishui
53935	Gansu Zhengning
56071	Gansu Luqu
56074	Gansu Maqu
56080	Gansu Hezuo
56081	Gansu Lintan
56082	Gansu Zhuoni
56084	Gansu Dibu
56091	Gansu Zhangxian
56092	Gansu Longxi
56093	Gansu Minxian
56094	Gansu Zhouqu

56095	Gansu Dangchang
56096	Gansu Wudu
56192	Gansu Wenxian
57001	Gansu Gangu
57002	Gansu Qinan
57004	Gansu Wushan
57006	Gansu Tianshui
57007	Gansu Lixian
57011	Gansu Qingshui
57012	Gansu Zhangjiachuan
57014	Gansu Maiji
57102	Gansu Chengxian
57105	Gansu Kangxian
57110	Gansu Huixian
57111	Gansu Liangdang
57988	Guangdong Lechang
57989	Guangdong Renhua
57996	Guangdong Nanxiong
59074	Guangdong Lianshan
59075	Guangdong Yangshan
59081	Guangdong Ruyuan
59082	Guangdong Shaoguan
59087	Guangdong Fogang
59088	Guangdong Yingde
59090	Guangdong Shixing
59094	Guangdong Wengyuan
59096	Guangdong Lianping
59099	Guangdong Heping
59106	Guangdong Pingyuan
59107	Guangdong Longchuan
59109	Guangdong Xingning
59114	Guangdong Jiaoling
59116	Guangdong Daipu
59117	Guangdong Meixian
59268	Guangdong Yunan
59270	Guangdong Huaiji
59271	Guangdong Guangning
59276	Guangdong Sihui
59279	Guangdong Sanshui
59280	Guangdong Qingyuan
59284	Guangdong Huadu
59285	Guangdong Conghua
59287	Guangdong Guangzhou

59289	Guangdong Dongguan
59290	Guangdong Longmen
59293	Guangdong Heyuan
59297	Guangdong Boluo
59303	Guangdong Wuhua
59304	Guangdong Zijin
59306	Guangdong Jiexi
59310	Guangdong Fengshun
59312	Guangdong Chaozhou
59313	Guangdong Raoping
59314	Guangdong Puning
59316	Guangdong Shantou
59456	Guangdong Xinyi
59462	Guangdong Luoding
59469	Guangdong Yangchun
59470	Guangdong Xinxing
59471	Guangdong Yunfu
59473	Guangdong Heshan
59475	Guangdong Kaiping
59476	Guangdong Xinhui
59477	Guangdong Enping
59481	Guangdong Panyu
59487	Guangdong Doumen
59488	Guangdong Zhuhai
59492	Guangdong Huidong
59493	Guangdong Shenzhen
59500	Guangdong Haifeng
59502	Guangdong Lufeng
59650	Guangdong Suixi
59653	Guangdong Gaozhou
59654	Guangdong Lianjiang
59655	Guangdong Huazhou
59656	Guangdong Wuchuan
59659	Guangdong Maoming
59750	Guangdong Leizhou
59754	Guangdong Xuwen
57859	Guangxi Ziyuan
57927	Guangxi Tiane
57941	Guangxi Sanjiang
57942	Guangxi Longsheng
57948	Guangxi Rongshui
57949	Guangxi Yongfu
57954	Guangxi Lingui

57955	Guangxi Xingan
57960	Guangxi Quanzhou
57964	Guangxi Guanyang
59004	Guangxi Xilin
59012	Guangxi Leye
59015	Guangxi Lingyun
59017	Guangxi Tianlin
59021	Guangxi Fengshan
59023	Guangxi Hechi
59027	Guangxi Bama
59034	Guangxi Yizhou
59037	Guangxi Duan
59038	Guangxi Xincheng
59041	Guangxi Liucheng
59045	Guangxi Luzhai
59053	Guangxi Pingle
59055	Guangxi Lipu
59057	Guangxi Jinxiu
59058	Guangxi Mengshan
59065	Guangxi Hezhou
59209	Guangxi Napo
59211	Guangxi Baise
59215	Guangxi Debao
59218	Guangxi Jingxi
59224	Guangxi Tiandong
59227	Guangxi Tiandeng
59229	Guangxi Longan
59230	Guangxi Mashan
59235	Guangxi Shanglin
59237	Guangxi Wuming
59238	Guangxi Binyang
59241	Guangxi Xiangzhou
59242	Guangxi Laibin
59246	Guangxi Wuxuan
59255	Guangxi Pingnan
59256	Guangxi Tengxian
59265	Guangxi Wuzhou
59266	Guangxi Cangwu
59419	Guangxi Pingxiang
59421	Guangxi Daxin
59429	Guangxi Shangsi
59431	Guangxi Nanning
59435	Guangxi Yongning

59441	Guangxi Hengxian
59446	Guangxi Lingshan
59448	Guangxi Pubei
59449	Guangxi Bobai
59451	Guangxi Beiliu
59452	Guangxi Rongxian
59454	Guangxi Cenxi
59457	Guangxi Luchuan
59632	Guangxi Qinzhou
59640	Guangxi Hepu
56598	Guizhou Hezhang
56691	Guizhou Weining
56792	Guizhou Puan
56793	Guizhou Panxian
57606	Guizhou Tongzi
57623	Guizhou Daozhen
57625	Guizhou Zhengan
57634	Guizhou Wuchuan
57636	Guizhou Yanhe
57637	Guizhou Dejiang
57647	Guizhou Songtao
57707	Guizhou Bijie
57708	Guizhou Dafang
57714	Guizhou Jinsha
57717	Guizhou Zunyi
57718	Guizhou Xifeng
57719	Guizhou Kaiyang
57722	Guizhou Meitan
57723	Guizhou Fenggang
57728	Guizhou Wengan
57729	Guizhou Yuqing
57731	Guizhou Sinan
57734	Guizhou Shiqian
57735	Guizhou Cengong
57736	Guizhou Jiangkou
57737	Guizhou Shibing
57739	Guizhou Yuping
57742	Guizhou Wanshan
57800	Guizhou Nayong
57803	Guizhou Xianxi
57805	Guizhou Zhijin
57806	Guizhou Anshun
57807	Guizhou Liuzhi

57811	Guizhou Xiuwen
57814	Guizhou Pingba
57821	Guizhou Fuquan
57822	Guizhou Huangping
57824	Guizhou Guiding
57825	Guizhou Kaili
57827	Guizhou Duyun
57832	Guizhou Sansui
57834	Guizhou Taijiang
57835	Guizhou Jianhe
57837	Guizhou Leishan
57839	Guizhou Liping
57840	Guizhou Tianzhu
57844	Guizhou Jinping
57900	Guizhou Qinglong
57903	Guizhou Guanling
57905	Guizhou Zhenfeng
57906	Guizhou Wangmo
57907	Guizhou Xingyi
57909	Guizhou Ceheng
57910	Guizhou Ziyun
57912	Guizhou Huishui
57913	Guizhou Longli
57916	Guizhou Luodian
57921	Guizhou Pingtang
57922	Guizhou Dushan
57923	Guizhou Sandu
57926	Guizhou Libo
57932	Guizhou Rongjiang
57936	Guizhou Congjiang
59758	Hainan Haikou
59842	Hainan Lingao
59843	Hainan Chengmai
59845	Hainan Danzhou
59847	Hainan Changjiang
59848	Hainan Baisha
59851	Hainan Dingan
59854	Hainan Tunchang
59855	Hainan Qonghai
59856	Hainan Wenchang
59945	Hainan Baoting
59951	Hainan Wanning
53392	Hebei Kangbao

53397	Hebei Shangyi
53399	Hebei Zhangbei
53491	Hebei Huaian
53492	Hebei Yangyuan
53498	Hebei Xuanhua
53499	Hebei Wanquan
53593	Hebei Weixian
53596	Hebei Shunping
53599	Hebei Laiyuan
53680	Hebei Lingshou
53682	Hebei Quyang
53688	Hebei Xingtang
53689	Hebei Jinzhou
53690	Hebei Fuping
53692	Hebei Tangxian
53696	Hebei Dingzhou
53699	Hebei Wuji
53773	Hebei Linzhang
53781	Hebei Shahe
53785	Hebei Baixiang
53789	Hebei Luancheng
53794	Hebei Longyao
53795	Hebei Zanhuang
53796	Hebei Ningjin
53799	Hebei Julu
53886	Hebei Shexian
53890	Hebei Wuan
53892	Hebei Handan
53893	Hebei Quzhou
53894	Hebei Fengfeng
53896	Hebei Weixian
54301	Hebei Guyuan
54304	Hebei Chongli
54308	Hebei Fengning
54311	Hebei Weichang
54318	Hebei Longhua
54319	Hebei Pingquan
54401	Hebei Zhangjiakou
54404	Hebei Chicheng
54405	Hebei Huailai
54408	Hebei Zhulu
54420	Hebei Luanping
54423	Hebei Chengde

54425	Hebei Xinglong
54429	Hebei Zunhua
54430	Hebei Chengdexian
54432	Hebei Kuancheng
54434	Hebei Qianxi
54436	Hebei Qinglong
54437	Hebei Luannan
54438	Hebei Lulong
54439	Hebei Qianan
54449	Hebei Qinhuangdao
54502	Hebei Zhuozhou
54503	Hebei Rongcheng
54506	Hebei Gaobeidian
54510	Hebei Dachang
54512	Hebei Guan
54519	Hebei Yongqing
54520	Hebei Sanhe
54521	Hebei Xianghe
54522	Hebei Yutian
54534	Hebei Tangshan
54535	Hebei Caofeidian
54539	Hebei Leting
54540	Hebei Changli
54541	Hebei Funing
54601	Hebei Xushui
54603	Hebei Goyang
54605	Hebei Anxin
54606	Hebei Raoyang
54608	Hebei Shenzhou
54610	Hebei Renqiu
54612	Hebei Wenan
54613	Hebei Dacheng
54614	Hebei Hejian
54615	Hebei Qingxian
54616	Hebei Cangzhou
54618	Hebei Botou
54624	Hebei Huanghua
54628	Hebei Haixing
54631	Hebei Guangzong
54633	Hebei Xinhe
54640	Hebei Jize
54700	Hebei Wuqiang
54701	Hebei Xinji

54702	Hebei Hengshui
54703	Hebei Wuyi
54706	Hebei Qinghe
54707	Hebei Gucheng
54710	Hebei Fucheng
54711	Hebei Jingxian
54713	Hebei Dongguang
54800	Hebei-Weixian
54801	Hebei Linxi
54804	Hebei Daming
54809	Hebei Guantao
53889	Henan Linzhou
53972	Henan Qinyang
53974	Henan Qixian
53978	Henan Jiyuan
53982	Henan Jiaozuo
53983	Henan Fengqiu
53984	Henan Xiuwu
53985	Henan Huixian
53986	Henan Xinxiang
53991	Henan Tangyin
53992	Henan Junxian
53993	Henan Neihuang
53998	Henan Changheng
54817	Henan Taiqian
54900	Henan Puyang
54902	Henan Qingfeng
54903	Henan Fanxian
57051	Henan Sanmenxia
57056	Henan Lingbao
57063	Henan Mianchi
57066	Henan Luoning
57070	Henan Xinan
57071	Henan Mengjin
57072	Henan Mengzhou
57074	Henan Yichuan
57075	Henan Ruzhou
57078	Henan Ruyang
57079	Henan Wenxian
57080	Henan Gongyi
57081	Henan Xingyang
57082	Henan Dengfeng
57087	Henan Changge

57089	Henan Xuchang
57090	Henan Zhongmou
57091	Henan Kaifeng
57093	Henan Lankao
57095	Henan Yanling
57096	Henan Qixian
57099	Henan Taikang
57156	Henan Xixia
57169	Henan Neixiang
57173	Henan Lushan
57175	Henan Zhenping
57176	Henan Nanzhao
57177	Henan Wugang
57178	Henan Nanyang
57179	Henan Fangcheng
57180	Henan Jiaxian
57182	Henan Xiangcheng
57183	Henan Linying
57184	Henan Yexian
57185	Henan Wuyang
57186	Henan Luohe
57187	Henan Sheqi
57188	Henan Xiping
57189	Henan Suiping
57192	Henan Huaiyang
57193	Henan Xihua
57197	Henan Runan
57271	Henan Xinye
57273	Henan Tanghe
57281	Henan Biyang
57285	Henan Tongbai
57290	Henan Zhumadian
57292	Henan Pingyu
57293	Henan Xincai
57295	Henan Zhengyang
57296	Henan Xixian
57297	Henan Xinyang
57299	Henan Guangshan
57390	Henan Jigongshan
57396	Henan Xixian
58004	Henan Minquan
58005	Henan Shangqiu
58006	Henan Yucheng

58007	Henan Echeng
58017	Henan Xiayi
58100	Henan Dancheng
58207	Henan Huangchuan
58208	Henan Gushi
58301	Henan Shangcheng
50136	Heilongjiang Mohe
50137	Heilongjiang Beijicun
50246	Heilongjiang Tahe
50247	Heilongjiang Huzhong
50349	Heilongjiang Xinlin
50353	Heilongjiang Huma
50442	Heilongjiang Jiagedaqi
50468	Heilongjiang Aihui
50557	Heilongjiang Nenjiang
50564	Heilongjiang Sunwu
50566	Heilongjiang Xunke
50646	Heilongjiang Nehe
50655	Heilongjiang Wudalianchi
50656	Heilongjiang Beian
50658	Heilongjiang Keshan
50659	Heilongjiang Kedong
50673	Heilongjiang Jiayin
50674	Heilongjiang Wuyiling
50739	Heilongjiang Longjiang
50741	Heilongjiang Gannan
50742	Heilongjiang Fuyu
50745	Heilongjiang Qiqihaer
50749	Heilongjiang Lindian
50750	Heilongjiang Yian
50755	Heilongjiang Baiquan
50756	Heilongjiang Hailun
50758	Heilongjiang Minshui
50767	Heilongjiang Suileng
50772	Heilongjiang Wuying
50774	Heilongjiang Yichun
50775	Heilongjiang Hegang
50776	Heilongjiang Luobei
50778	Heilongjiang Tongjiang
50779	Heilongjiang Fuyuan
50787	Heilongjiang Suibin
50788	Heilongjiang Fujin
50842	Heilongjiang Dumeng

50844	Heilongjiang Tailai
50850	Heilongjiang Daqing
50851	Heilongjiang Qinggang
50852	Heilongjiang Wangkui
50853	Heilongjiang Beilin
50854	Heilongjiang Anda
50858	Heilongjiang Zhaodong
50859	Heilongjiang Lansi
50861	Heilongjiang Qingan
50862	Heilongjiang Tieli
50867	Heilongjiang Bayan
50871	Heilongjiang Tangyuan
50873	Heilongjiang Jiamusi
50877	Heilongjiang Yilang
50878	Heilongjiang Huachuan
50879	Heilongjiang Huanan
50884	Heilongjiang Shuangyashan
50888	Heilongjiang Baoqing
50892	Heilongjiang Raohe
50950	Heilongjiang Zhaozhou
50953	Heilongjiang Haerbin
50956	Heilongjiang Hulan
50958	Heilongjiang Acheng
50960	Heilongjiang Binxian
50962	Heilongjiang Mulan
50963	Heilongjiang Tonghe
50964	Heilongjiang Zhengfang
50965	Heilongjiang Yanshou
50968	Heilongjiang Shangzhi
50971	Heilongjiang Qitaihe
50973	Heilongjiang Boli
50978	Heilongjiang Jixi
50979	Heilongjiang Linkou
50983	Heilongjiang Hulin
50985	Heilongjiang Mishan
50987	Heilongjiang Jidong
54080	Heilongjiang Wuchang
54092	Heilongjiang Hailin
54093	Heilongjiang Muling
54094	Heilongjiang Mudanjiang
54096	Heilongjiang Suifenhe
54098	Heilongjiang Ningan
57249	Hubei Zhuxi

57251	Hubei Yunxi
57253	Hubei Yunxian
57256	Hubei Shiyan
57257	Hubei Zhushan
57259	Hubei Fangxian
57260	Hubei Danjiangkou
57268	Hubei Gucheng
57278	Hubei Xiangyang
57279	Hubei Zaoyang
57355	Hubei Padang
57359	Hubei Xingshan
57361	Hubei Baokang
57362	Hubei Shennongjia
57363	Hubei Nanzhang
57370	Hubei Yicheng
57377	Hubei Jingmen
57378	Hubei Zhongxiang
57381	Hubei Suizhou
57386	Hubei Xiaochang
57387	Hubei Jingshan
57388	Hubei Anlu
57398	Hubei Hongan
57399	Hubei Macheng
57439	Hubei Lichuan
57445	Hubei Jianshi
57447	Hubei Enshi
57453	Hubei Yiling
57458	Hubei Wufeng
57469	Hubei Songzi
57475	Hubei Qianjiang
57477	Hubei Gongan
57481	Hubei Yingcheng
57482	Hubei Xiaogan
57483	Hubei Tianmen
57484	Hubei Shayang
57485	Hubei Xiantao
57486	Hubei Hanchuan
57489	Hubei Caidian
57492	Hubei Xinzhou
57494	Hubei Wuhan
57495	Hubei Tuanfeng
57496	Hubei Ezhou
57540	Hubei Xianfeng

57541	Hubei Xuanen
57543	Hubei Hefeng
57571	Hubei Shishou
57573	Hubei Jianli
57581	Hubei Honghu
57582	Hubei Chibi
57583	Hubei Jiayu
57586	Hubei Chongyang
57589	Hubei Tongcheng
57590	Hubei Xianning
58401	Hubei Luotian
58402	Hubei Yingshan
58408	Hubei Qichun
58409	Hubei Huangmei
58500	Hubei Yangxin
58501	Hubei Wuxue
57544	Hunan Longshan
57554	Hunan Sangzhi
57558	Hunan Zhangjiajie
57565	Hunan Lixian
57574	Hunan Nanxian
57575	Hunan Huarong
57584	Hunan Yueyang
57642	Hunan Baojing
57643	Hunan Yongshun
57646	Hunan Guzhang
57649	Hunan Jishou
57655	Hunan Yuanling
57657	Hunan Luxi
57661	Hunan Taoyuan
57662	Hunan Changde
57663	Hunan Hanshou
57666	Hunan Taojiang
57669	Hunan Anhua
57671	Hunan Yuanjiang
57673	Hunan Xiangyin
57678	Hunan Ningxiang
57680	Hunan Milo
57682	Hunan Pingjiang
57687	Hunan Changsha
57688	Hunan Liuyang
57740	Hunan Fenghuang
57744	Hunan Xinhuang

57745	Hunan Zhijiang
57752	Hunan Xupu
57754	Hunan Hongjiang
57760	Hunan Lengshuijiang
57761	Hunan Xinhua
57763	Hunan Loudi
57771	Hunan Shaoshan
57772	Hunan Xiangxiang
57774	Hunan Shuangfeng
57776	Hunan Nanyue
57777	Hunan Hengshan
57779	Hunan Youxian
57780	Hunan Zhuzhou
57781	Hunan Liling
57845	Hunan Tongtao
57865	Hunan Lengshuitan
57866	Hunan Yongzhou
57867	Hunan Dongan
57868	Hunan Qiyang
57870	Hunan Qidong
57871	Hunan Hengyangxian
57872	Hunan Hengyang
57874	Hunan Changning
57875	Hunan Hengnan
57881	Hunan Anren
57882	Hunan Chaling
57887	Hunan Yongxing
57889	Hunan Guangxi
57962	Hunan Shuangpai
57965	Hunan Daoxian
57966	Hunan Ningyuan
57969	Hunan Jiangyong
57971	Hunan Xintian
57972	Hunan Chenzhou
57973	Hunan Guiyang
57974	Hunan Jiahe
57975	Hunan Lanshan
57976	Hunan Yizhang
57978	Hunan Linwu
57981	Hunan Zixing
57985	Hunan Rucheng
59063	Hunan Jianghua
50936	Jilin Baicheng

50939	Jilin Taonan
50940	Jilin Zhenlai
50945	Jilin Daan
50946	Jilin Songyuan
50948	Jilin Qianan
50949	Jilin Qianguo
54041	Jilin Tongyu
54049	Jilin Changling
54063	Jilin Fuyu
54064	Jilin Nongan
54065	Jilin Dehui
54069	Jilin Jiutai
54072	Jilin Yushu
54076	Jilin Shulan
54142	Jilin Shuangliao
54154	Jilin Lishu
54155	Jilin Gujiazi
54161	Jilin Changchun
54164	Jilin Yitong
54165	Jilin Shuangyang
54169	Jilin Yantongshan
54171	Jilin Yongji
54172	Jilin Jilin Suburb
54181	Jilin Jiaohe
54186	Jilin Dunhua
54187	Jilin Antu
54192	Jilin Luozigou
54195	Jilin Wangqing
54260	Jilin Liaoyuan
54261	Jilin Tongfeng
54263	Jilin Panshi
54267	Jilin Liuhe
54273	Jilin Huadian
54274	Jilin Huinan
54279	Jilin Jiangyuan
54284	Jilin Donggang
54285	Jilin Erdao
54286	Jilin Helong
54290	Jilin Longjing
54291	Jilin Huichun
54292	Jilin Yanji
54362	Jilin Tonghuaxian
54363	Jilin Tonghua

54371	Jilin Baishan
54374	Jilin Linjiang
54377	Jilin Jian
54386	Jilin Changbai
58012	Jiangsu Fengxian
58013	Jiangsu Peixian
58026	Jiangsu Pizhou
58027	Jiangsu Xuzhou
58036	Jiangsu Donghai
58038	Jiangsu Shuyang
58040	Jiangsu Ganyu
58041	Jiangsu Xiliandao
58044	Jiangsu Lianyungang
58045	Jiangsu Xiangshui
58047	Jiangsu Guanyun
58130	Jiangsu Suining
58131	Jiangsu Suyu
58132	Jiangsu Siyang
58135	Jiangsu Sihong
58138	Jiangsu Xuyi
58139	Jiangsu Hongze
58140	Jiangsu Lianshui
58141	Jiangsu Huaian
58143	Jiangsu Funing
58146	Jiangsu Jianhu
58147	Jiangsu Jinhu
58148	Jiangsu Baoying
58154	Jiangsu Yancheng
58158	Jiangsu Dafeng
58235	Jiangsu Liuhe
58237	Jiangsu Pukou
58238	Jiangsu Nanjing
58241	Jiangsu Gaoyou
58242	Jiangsu Yizheng
58243	Jiangsu Xinghua
58246	Jiangsu Taizhou
58247	Jiangsu Yangzhong
58249	Jiangsu Taixing
58250	Jiangsu Jiangyan
58251	Jiangsu Dongtai
58254	Jiangsu Haian
58255	Jiangsu Rugao
58257	Jiangsu Jingjiang

58259	Jiangsu Nantong
58264	Jiangsu Rudong
58265	Jiangsu Lvsi
58269	Jiangsu Qidong
58339	Jiangsu Gaochun
58340	Jiangsu Lishui
58341	Jiangsu Danyang
58342	Jiangsu Jintan
58344	Jiangsu Jurong
58346	Jiangsu Yixing
58349	Jiangsu Suzhou
58352	Jiangsu Changshu
58353	Jiangsu Zhangjiagang
58354	Jiangsu Wuxi
58356	Jiangsu Kunshan
58360	Jiangsu Haimen
58377	Jiangsu Taicang
57598	Jiangxi Xiushui
57694	Jiangxi Tonggu
57698	Jiangxi Wanzai
57699	Jiangxi Shanggao
57786	Jiangxi Pingxiang
57789	Jiangxi Lianhua
57792	Jiangxi Fenyi
57793	Jiangxi Yichun
57796	Jiangxi Xinyu
57798	Jiangxi Anfu
57799	Jiangxi Jianxian
57883	Jiangxi Xiaping
57891	Jiangxi Yongxin
57895	Jiangxi Wanan
57896	Jiangxi Suichuan
57899	Jiangxi Taihe
57990	Jiangxi Chongyi
57992	Jiangxi Nankang
57993	Jiangxi Ganxian
57995	Jiangxi Xinfeng
58502	Jiangxi Jiujiang
58503	Jiangxi Ruichang
58506	Jiangxi Lushan
58507	Jiangxi Wuning
58508	Jiangxi Dean
58510	Jiangxi Hukou

58512	Jiangxi Pengze
58517	Jiangxi Duchang
58519	Jiangxi Poyang
58527	Jiangxi Jingdezhen
58529	Jiangxi Wuyuan
58600	Jiangxi Jingan
58601	Jiangxi Fengxin
58602	Jiangxi Anyi
58605	Jiangxi Gaoan
58606	Jiangxi Nanchang
58612	Jiangxi Yugan
58614	Jiangxi Jinxian
58615	Jiangxi Wannian
58618	Jiangxi Dongxiang
58619	Jiangxi Linchuan
58622	Jiangxi Dexing
58623	Jiangxi Shangraoxian
58626	Jiangxi Guixi
58629	Jiangxi Qianshan
58634	Jiangxi Yushan
58693	Jiangxi Xinjian
58704	Jiangxi Xiajiang
58705	Jiangxi Yongfeng
58706	Jiangxi Lean
58710	Jiangxi Chongren
58712	Jiangxi Jinxi
58715	Jiangxi Nancheng
58718	Jiangxi Nanfeng
58719	Jiangxi Lichuan
58804	Jiangxi Xingguo
58806	Jiangxi Ningdu
58813	Jiangxi Guangchang
58814	Jiangxi Shicheng
58905	Jiangxi Yudu
58906	Jiangxi Huichang
58907	Jiangxi Anyuan
59091	Jiangxi Quannan
59092	Jiangxi Longnan
59093	Jiangxi Dingnan
59102	Jiangxi Xunwu
57783	Jiangxi Shangli
54236	Liaoning Zhangwu
54243	Liaoning Changtu

54244	Liaoning Kangping
54248	Liaoning Shenbei
54249	Liaoning Tieling
54252	Liaoning Xifeng
54259	Liaoning Qingyuan
54321	Liaoning Jianpingzhen
54324	Liaoning Chaoyang
54325	Liaoning Yangshan
54326	Liaoning Jianpingxian
54327	Liaoning Lingyuan
54332	Liaoning Liaozhong
54333	Liaoning Xinmin
54336	Liaoning Taian
54338	Liaoning Panshan
54339	Liaoning Anshan
54340	Liaoning Sujiatun
54342	Liaoning Shenyang
54345	Liaoning Liaoyangxian
54346	Liaoning Benxi
54351	Liaoning Fushun
54353	Liaoning Xinbin
54452	Liaoning Jianchang
54453	Liaoning Lianshan
54454	Liaoning Suizhong
54455	Liaoning Xingcheng
54471	Liaoning Yingkou
54474	Liaoning Gaizhou
54475	Liaoning Dashiqiao
54483	Liaoning Caohekou
54486	Liaoning Xiuyan
54493	Liaoning Kuandian
54494	Liaoning Fengcheng
54497	Liaoning Dandong
54563	Liaoning Wafangdian
54568	Liaoning Jinzhou
54569	Liaoning Pulandian
54575	Liaoning Pikou
54579	Liaoning Changhai
54584	Liaoning Zhuanghe
54565	Liaoning Changxingdao
50425	Inner Mongolia Eerguna
50434	Inner Mongolia Tulihe
50445	Inner Mongolia Elunchunqi

50514	Inner Mongolia Manzhouli
50525	Inner Mongolia Evenkeqi
50526	Inner Mongolia Yakeshi
50548	Inner Mongolia Xiaoergou
50603	Inner Mongolia Xinbaerhuyouqi
50618	Inner Mongolia Xinbaerhuzuoqi
50639	Inner Mongolia Zhalantun
50645	Inner Mongolia Moulidawawoer
50647	Inner Mongolia Arunqi
50727	Inner Mongolia Aershan
50834	Inner Mongolia Suolun
50913	Inner Mongolia Wulagai
50915	Inner Mongolia Wuzhumuqindong
50924	Inner Mongolia Houlinguole
50928	Inner Mongolia Bayaertuhushuo
50934	Inner Mongolia Tuquan
53068	Inner Mongolia Erlianhaote
53083	Inner Mongolia Narenbaolige
53149	Inner Mongolia Mandula
53192	Inner Mongolia Abagaqi
53231	Inner Mongolia Hailisu
53289	Inner Mongolia Xianghuangqi
53336	Inner Mongolia Wulatezhongqi
53337	Inner Mongolia Wuyuan
53348	Inner Mongolia Dashetai
53352	Inner Mongolia Damaoqi
53357	Inner Mongolia Guyangxian
53362	Inner Mongolia Siziwang
53367	Inner Mongolia Xilamuren
53368	Inner Mongolia Wuchuanxian
53378	Inner Mongolia Chayouzhongqi
53384	Inner Mongolia Chayouhouqi
53385	Inner Mongolia Shangdu
53391	Inner Mongolia Huade
53419	Inner Mongolia Dengkou
53420	Inner Mongolia Hangjinhouqi
53433	Inner Mongolia Wulateqianqi
53446	Inner Mongolia Baotou
53455	Inner Mongolia Tuyouqi
53457	Inner Mongolia Dalateqi
53463	Inner Mongolia Huhehaote
53464	Inner Mongolia Tumutezuoqi
53466	Inner Mongolia Huhehaote suburb

53467	Inner Mongolia Tuoketuoxian
53469	Inner Mongolia Helingeerxian
53472	Inner Mongolia Zhuozi
53475	Inner Mongolia Liangcheng
53480	Inner Mongolia Jining
53481	Inner Mongolia Chayouqianqi
53483	Inner Mongolia Xinghe
53512	Inner Mongolia Wuhai
53513	Inner Mongolia Linhe
53522	Inner Mongolia Yikewusu
53529	Inner Mongolia Etukeqi
53533	Inner Mongolia Hangjinqi
53543	Inner Mongolia Dongsheng
53545	Inner Mongolia Ejinhuoluoqi
53553	Inner Mongolia Zhungeerqi
53562	Inner Mongolia Qingshuihexian
53644	Inner Mongolia Wushenqi
53730	Inner Mongolia Eduokeqianqi
53732	Inner Mongolia Henan
54012	Inner Mongolia Xiwuzhumuqin
54024	Inner Mongolia Fuhe
54026	Inner Mongolia Zhalute
54027	Inner Mongolia Balinzuoqi
54031	Inner Mongolia Gaoliban
54039	Inner Mongolia Shebotu
54047	Inner Mongolia Kezuozhongqi
54102	Inner Mongolia Xilinhaote
54113	Inner Mongolia Balinyouqi
54115	Inner Mongolia Linxixian
54117	Inner Mongolia Keshiketengqi
54122	Inner Mongolia Alukeerqinqi
54132	Inner Mongolia Qinglongshan
54134	Inner Mongolia Kailu
54135	Inner Mongolia Tongliao
54204	Inner Mongolia Zhengxiangbaiqi
54205	Inner Mongolia Zhenglanqi
54208	Inner Mongolia Duolunxian
54213	Inner Mongolia Wengniuteqi
54214	Inner Mongolia Gangzi
54218	Inner Mongolia Chifeng
54223	Inner Mongolia Neiman
54225	Inner Mongolia Aohanqi
54226	Inner Mongolia Baoguotu

54231	Inner Mongolia Kezuohouqi
54234	Inner Mongolia Kulun
54305	Inner Mongolia Taibushiqi
54313	Inner Mongolia Kalaqinqi
54316	Inner Mongolia Balihan
54320	Inner Mongolia Ningchengxian
53517	Ningxia Shitanjing
53519	Ningxia Huinong
53610	Ningxia Helan
53611	Ningxia Pingluo
53612	Ningxia Wuzhong
53615	Ningxia Taole
53618	Ningxia Yongning
53704	Ningxia Zhongwei
53705	Ningxia Zhongning
53707	Ningxia Xingren
53723	Ningxia Yanchi
53727	Ningxia Maihuangshan
53806	Ningxia Haiyuan
53810	Ningxia Tongxin
53817	Ningxia Guyuan
53881	Ningxia Weizhou
53903	Ningxia Xiji
53910	Ningxia Liupanshan
52602	Qinghai Lenghu
52825	Qinghai Nuomuhong
52836	Qinghai Doulan
52854	Qinghai Qinghaihu 151
52856	Qinghai Gonghe
52863	Qinghai Huzhu
52866	Qinghai Xining
52868	Qinghai Guide
52875	Qinghai Pingan
52876	Qinghai Minhe
52877	Qinghai Hualong
52908	Qinghai Wudaoliang
52941	Qinghai Shazhuyu
52943	Qinghai Xinghai
52955	Qinghai Guinan
52957	Qinghai Tongde
52963	Qinghai Jianzha
52968	Qinghai Zeku
52972	Qinghai Xunhua

52974	Qinghai Tongren
56004	Qinghai Tuotuohe
56016	Qinghai Zhiduo
56018	Qinghai Zaduo
56021	Qinghai Qumacai
56029	Qinghai Yushu
56033	Qinghai Maduo
56034	Qinghai Qingshuihe
56043	Qinghai Maqin
56045	Qinghai Gander
56046	Qinghai Dari
56065	Qinghai Henan
56067	Qinghai Jiuzhi
56125	Qinghai Nangqian
56151	Qinghai Banma
54709	Shandong Wucheng
54712	Shandong Linyi
54716	Shandong Ningjin
54723	Shandong Yangxin
54724	Shandong Shanghe
54726	Shandong Leling
54727	Shandong Zhangqiu
54729	Shandong Gaoqing
54734	Shandong Binzhou
54744	Shandong Kenli
54749	Shandong Laizhou
54751	Shandong Longdao
54752	Shandong Penglai
54753	Shandong Longkou
54755	Shandong Zhaoyuan
54759	Shandong Qixia
54764	Shandong Fushan
54765	Shandong Yantai
54776	Shandong Chengshantou
54777	Shandong Wendeng
54802	Shandong Linqing
54806	Shandong Liaocheng
54808	Shandong Xinxian
54812	Shandong Qihe
54814	Shandong Chiping
54815	Shandong Donge
54819	Shandong Feicheng
54821	Shandong Jiyang

54822	Shandong Zouping
54823	Shandong Jinan
54827	Shandong Taian
54828	Shandong Laiwu
54830	Shandong Zibo
54831	Shandong Qingzhou
54832	Shandong Shouguang
54833	Shandong Huantai
54836	Shandong Yiyuan
54841	Shandong Changyi
54842	Shandong Pingdu
54843	Shandong Weifang
54844	Shandong Anqiu
54846	Shandong Gaomi
54848	Shandong Zhucheng
54849	Shandong Jiaozhou
54852	Shandong Laiyang
54855	Shandong Jimo
54861	Shandong Rushan
54904	Shandong Juancheng
54905	Shandong Yuncheng
54906	Shandong Heze
54907	Shandong Yutai
54909	Shandong Dingtao
54910	Shandong Liangshan
54911	Shandong Dongping
54912	Shandong Wenshang
54914	Shandong Juye
54916	Shandong Yanzhou
54919	Shandong Zoucheng
54920	Shandong Sishui
54922	Shandong Xintai
54923	Shandong Mengyin
54925	Shandong Pingyi
54927	Shandong Tengzhou
54929	Shandong Feixian
54932	Shandong Yishui
54938	Shandong Linyi
54939	Shandong Junan
54940	Shandong Wulian
54943	Shandong Huangdao
54945	Shandong Rizhao
58002	Shandong Caoxian

58003	Shandong Chengwu
58021	Shandong Xuechen
58022	Shandong Yicheng
58024	Shandong Zaozhuang
58030	Shandong Cangshan
58032	Shandong Linshu
53478	Shanxi Youyu
53486	Shanxi Yanggao
53487	Shanxi Datong
53564	Shanxi Hequ
53565	Shanxi Pianguan
53574	Shanxi Pinglu
53575	Shanxi Shenchi
53576	Shanxi Shanyin
53577	Shanxi Ningwu
53578	Shanxi Shuozhou
53579	Shanxi Daixian
53582	Shanxi Hunyuan
53584	Shanxi Yingxian
53585	Shanxi Fanshi
53588	Shanxi Wutaishan
53590	Shanxi Guangling
53659	Shanxi Linxian
53662	Shanxi Kelan
53663	Shanxi Wuzhai
53664	Shanxi Xingxian
53665	Shanxi Lanxian
53666	Shanxi Jingle
53673	Shanxi Yuanping
53674	Shanxi Xinfu
53676	Shanxi Dingxiang
53677	Shanxi Jiancaoping
53679	Shanxi Xiaodian
53681	Shanxi Wutaixian
53685	Shanxi Yuxian
53687	Shanxi Pingding
53753	Shanxi Liulin
53759	Shanxi Shilou
53760	Shanxi Fangshan
53763	Shanxi Gujiao
53764	Shanxi Lishi
53767	Shanxi Zhongyang
53768	Shanxi Xiaoyi

53774	Shanxi Qingxu
53775	Shanxi Taigu
53778	Shanxi Pingyao
53780	Shanxi Shouyang
53782	Shanxi Yangquan
53786	Shanxi Zuoquan
53787	Shanxi Yushe
53788	Shanxi Heshun
53852	Shanxi Yonghe
53853	Shanxi Xixian
53859	Shanxi Jixian
53860	Shanxi Jiaokou
53861	Shanxi Xiangfen
53862	Shanxi Lingshi
53863	Shanxi Jiexiu
53864	Shanxi Puxian
53865	Shanxi Fenxi
53866	Shanxi Hongtong
53871	Shanxi Wuxiang
53872	Shanxi Qinxian
53873	Shanxi Changzi
53874	Shanxi Guxian
53875	Shanxi Qinyuan
53877	Shanxi Anze
53878	Shanxi Licheng
53880	Shanxi Lucheng
53953	Shanxi Xiangning
53954	Shanxi Jishan
53956	Shanxi Wanrong
53957	Shanxi Hejin
53959	Shanxi Yanhu
53964	Shanxi Xinjiang
53965	Shanxi Jiangxian
53966	Shanxi Fushan
53968	Shanxi Yuanqu
53970	Shanxi Qingshui
53973	Shanxi Gaoping
53981	Shanxi Lingchuan
57052	Shanxi Yongji
57053	Shanxi Ruicheng
53567	Shaanxi Fugu
53646	Shaanxi Yulin
53651	Shaanxi Shenmu

53658	Shaanxi Jiaxian
53725	Shaanxi Dingbian
53735	Shaanxi Jingbian
53738	Shaanxi Wuqi
53740	Shaanxi Hengshan
53748	Shaanxi Zichang
53754	Shaanxi Suide
53756	Shaanxi Wubao
53757	Shaanxi Qingjian
53832	Shaanxi Zhidan
53841	Shaanxi Ansai
53848	Shaanxi Ganquan
53850	Shaanxi Yanchuan
53854	Shaanxi Yanchang
53857	Shaanxi Yichuan
53931	Shaanxi Fuxian
53938	Shaanxi Xunyi
53941	Shaanxi Baishui
53944	Shaanxi Huangling
53946	Shaanxi Huanglong
53947	Shaanxi Tongchuan
53948	Shaanxi Pucheng
53949	Shaanxi Chengcheng
53950	Shaanxi Heyang
57003	Shaanxi Longxian
57016	Shaanxi Baoji
57021	Shaanxi Qianyang
57022	Shaanxi Linyou
57026	Shaanxi Fufeng
57027	Shaanxi Meixian
57029	Shaanxi Liquan
57030	Shaanxi Yongshou
57034	Shaanxi Wugong
57037	Shaanxi Yaoxian
57038	Shaanxi Xingping
57041	Shaanxi Sanyuan
57042	Shaanxi Fuping
57043	Shaanxi Dali
57045	Shaanxi Weinan
57046	Shaanxi Huashan
57057	Shaanxi Luonan
57106	Shaanxi Lueyang
57113	Shaanxi Fengxian

57119	Shaanxi Mianxian
57124	Shaanxi Liuba
57128	Shaanxi Chenggu
57134	Shaanxi Foping
57137	Shaanxi Ningshan
57140	Shaanxi Zhashui
57143	Shaanxi Shangxian
57153	Shaanxi Danfeng
57154	Shaanxi Shangnan
57155	Shaanxi Shanyang
57211	Shaanxi Ningqiang
57213	Shaanxi Nanzheng
57231	Shaanxi Ziyang
57233	Shaanxi Hanyin
57238	Shaanxi Zhenba
57242	Shaanxi Xunyang
57245	Shaanxi Ankang
57247	Shaanxi Langao
57248	Shaanxi Pingli
57254	Shaanxi Baihe
57343	Shaanxi Zhenping
58361	Shanghai Minhang
58362	Shanghai Baoshan
58460	Shanghai Jinshan
58463	Shanghai Fengxian
56038	Sichuan Shiqu
56079	Sichuan Ruoergai
56097	Sichuan Jiuzhaigou
56144	Sichuan Dege
56146	Sichuan Ganzi
56147	Sichuan Baiyu
56152	Sichuan Seda
56158	Sichuan Luhuo
56164	Sichuan Rangtang
56167	Sichuan Daofu
56168	Sichuan Jinchuan
56171	Sichuan Aba
56172	Sichuan Maerkang
56173	Sichuan Hongyuan
56178	Sichuan Xiaojin
56180	Sichuan Maoxian
56181	Sichuan Chongzhou
56182	Sichuan Songpan

56183	Sichuan Wenchuan
56184	Sichuan Lixian
56185	Sichuan Heishui
56186	Sichuan Mianzhu
56187	Sichuan Wenjiang
56188	Sichuan Dujiangyan
56189	Sichuan Pengzhou
56198	Sichuan Deyang
56247	Sichuan Batang
56251	Sichuan Xinlong
56257	Sichuan Litang
56263	Sichuan Danba
56267	Sichuan Yajiang
56273	Sichuan Baoxing
56279	Sichuan Lushan
56280	Sichuan Mingshan
56281	Sichuan Pujiang
56286	Sichuan Longquanyi
56289	Sichuan Pengshan
56296	Sichuan Jintang
56297	Sichuan Renshou
56298	Sichuan Ziyang
56357	Sichuan Daocheng
56371	Sichuan Luding
56373	Sichuan Yingjing
56374	Sichuan Kangding
56376	Sichuan Hanyuan
56378	Sichuan Shimian
56380	Sichuan Hongya
56382	Sichuan Jiajiang
56383	Sichuan Qingshen
56385	Sichuan Emeishan
56387	Sichuan Ebian
56389	Sichuan Qianwei
56390	Sichuan Jingyan
56393	Sichuan Zizhong
56395	Sichuan Weiyuan
56396	Sichuan Zigong
56399	Sichuan Fushun
56441	Sichuan Derong
56443	Sichuan Xiangcheng
56459	Sichuan Muli
56462	Sichuan Jiulong

56473	Sichuan Ganluo
56474	Sichuan Mianning
56475	Sichuan Yuexi
56478	Sichuan Xide
56479	Sichuan Chaojue
56480	Sichuan Mabian
56485	Sichuan Leibo
56487	Sichuan Meigu
56490	Sichuan Muchuan
56491	Sichuan Yibinxian
56493	Sichuan Nanxi
56494	Sichuan Pingshan
56496	Sichuan Xingwen
56499	Sichuan Gongxian
56565	Sichuan Yanyuan
56569	Sichuan Dechang
56571	Sichuan Xichang
56575	Sichuan Puge
56580	Sichuan Butuo
56584	Sichuan Jinyang
56593	Sichuan Changning
56665	Sichuan Yanbian
56666	Sichuan Panzhihua
56670	Sichuan Miyi
56671	Sichuan Huili
56675	Sichuan Huidong
57204	Sichuan Qingchuan
57206	Sichuan Guangyuan
57208	Sichuan Jiange
57216	Sichuan Nanjiang
57217	Sichuan Wangcang
57237	Sichuan Wanyuan
57306	Sichuan Langzhong
57309	Sichuan Xichong
57313	Sichuan Bazhong
57314	Sichuan Nanbu
57315	Sichuan Yilong
57318	Sichuan Yingshan
57320	Sichuan Tongjiang
57324	Sichuan Pingchang
57328	Sichuan Dachuan
57329	Sichuan Kaijiang
57401	Sichuan Shehong

57405	Sichuan Suining
57411	Sichuan Gaoping
57413	Sichuan Quxian
57415	Sichuan Guangan
57416	Sichuan Linshui
57417	Sichuan Wusheng
57420	Sichuan Dazhu
57503	Sichuan Dongxing
57507	Sichuan Longchang
57600	Sichuan Jiangan
57603	Sichuan Hejiang
57608	Sichuan Xuyong
54523	Tianjin Wuqing
54525	Tianjin Baodi
54619	Tianjin Jinghai
54622	Tianjin Jinnan
54623	Tianjin Tanggu
54645	Tianjin Dagang
55228	Tibet Shiquanhe
55248	Tibet Gaize
55299	Tibet Naqu
55437	Tibet Pulan
55493	Tibet Dangxiong
55569	Tibet Lazi
55572	Tibet Nanmulin
55593	Tibet Muozhugongka
55598	Tibet Zedang
55655	Tibet Nielamu
55664	Tibet Dingri
55680	Tibet Jiangzi
56116	Tibet Dingqing
56128	Tibet Leiwuqi
56137	Tibet Changdu
56223	Tibet Luolong
56227	Tibet Bomi
56228	Tibet Basu
56312	Tibet Linzhi
56317	Tibet Miling
56331	Tibet Zuogong
56434	Tibet Chayu
51053	Xinjiang Habahe
51058	Xinjiang Akedala
51060	Xinjiang Buerjin

51068	Xinjiang Fuhai
51076	Xinjiang Aletai
51087	Xinjiang Fuyun
51133	Xinjiang Tacheng
51137	Xinjiang Yumin
51145	Xinjiang Emin
51156	Xinjiang Hebukesaier
51232	Xinjiang Alashankou
51238	Xinjiang Bole
51241	Xinjiang Touli
51243	Xinjiang Kelamayi
51334	Xinjiang Jinghe
51357	Xinjiang Shawan
51359	Xinjiang Manasi
51367	Xinjiang Hutubi
51368	Xinjiang Changji
51369	Xinjiang Miquan
51377	Xinjiang Fukang
51378	Xinjiang Jimusaer
51468	Xinjiang Tianshandaxigou
51469	Xinjiang Urumqi Pastoral Test Station
51470	Xinjiang Tianchi
51477	Xinjiang Dabancheng
51482	Xinjiang Mulei
51526	Xinjiang Kumishi
51542	Xinjiang Bayinbuluke
51567	Xinjiang Yanqi
51571	Xinjiang Tuokexun
51572	Xinjiang Tulufandongkan
51573	Xinjiang Tulufan
51627	Xinjiang Wushi
51628	Xinjiang Akesu
51636	Xinjiang Xinhe
51639	Xinjiang Shaya
51656	Xinjiang Kuerle
51704	Xinjiang Atushi
51705	Xinjiang Wuqia
51707	Xinjiang Jiashi
51709	Xinjiang Kashi
51717	Xinjiang Yuepuhu
51720	Xinjiang Kepin
51722	Xinjiang Awati
51730	Xinjiang Alaer

51747	Xinjiang Tazhong
51765	Xinjiang Tieqianlike
51802	Xinjiang Yengjisha
51810	Xinjiang Maigaiti
51811	Xinjiang Shashe
51814	Xinjiang Yecheng
51815	Xinjiang Zepu
51818	Xinjiang Pishan
51826	Xinjiang Cele
51827	Xinjiang Moyu
51828	Xinjiang Hetan
51829	Xinjiang Luopu
51839	Xinjiang Minfeng
51855	Xinjiang Qiemuo
51931	Xinjiang Yutian
52101	Xinjiang Balikun
52112	Xinjiang Naomaohu
52118	Xinjiang Yiwu
52203	Xinjiang Hami
52313	Xinjiang Hongliuhe
56444	Yunnan Deqin
56483	Yunnan Suijiang
56497	Yunnan Yanjin
56533	Yunnan Gongshan
56543	Yunnan Xianggelila
56548	Yunnan Weixi
56567	Yunnan Ninglang
56582	Yunnan Daguan
56585	Yunnan Ludian
56594	Yunnan Yiliang
56595	Yunnan Zhenxiong
56596	Yunnan Weixin
56641	Yunnan Fugong
56643	Yunnan Liuku
56645	Yunnan Lanping
56646	Yunnan Jianchuan
56649	Yunnan Eryuan
56651	Yunnan Lijiang
56652	Yunnan Yongsheng
56654	Yunnan Heqing
56664	Yunnan Huaping
56669	Yunnan Yongren
56673	Yunnan Qiaojia

56688	Yunnan Dongchuan
56697	Yunnan Xuanwei
56739	Yunnan Tengchong
56742	Yunnan Yunlong
56745	Yunnan Yangbi
56746	Yunnan Yongping
56748	Yunnan Baoshan
56751	Yunnan Dali
56752	Yunnan Binchuan
56755	Yunnan Midu
56757	Yunnan Weishan
56764	Yunnan Yaoan
56766	Yunnan Mouding
56767	Yunnan Nanhua
56772	Yunnan Fumin
56774	Yunnan Wuding
56777	Yunnan Lufeng
56778	Yunnan Kunming
56782	Yunnan Malong
56783	Yunnan Qujing
56785	Yunnan Songming
56790	Yunnan Fuyuan
56835	Yunnan Longchuan
56836	Yunnan Yingjiang
56839	Yunnan Zhenkang
56840	Yunnan Lianghe
56841	Yunnan Longling
56842	Yunnan Shidian
56843	Yunnan Changning
56846	Yunnan Fengqing
56849	Yunnan Yongde
56854	Yunnan Yunxian
56856	Yunnan Jingdong
56862	Yunnan Shuangbai
56863	Yunnan Anning
56867	Yunnan Zhenyuan
56869	Yunnan Xinping
56870	Yunnan Yimen
56871	Yunnan Jinning
56873	Yunnan Chengjiang
56875	Yunnan Yuxi
56879	Yunnan Huaning
56880	Yunnan Yiliang

56881	Yunnan Shilin
56883	Yunnan Shizong
56885	Yunnan Mile
56886	Yunnan Luxi
56889	Yunnan Qiubei
56891	Yunnan Luoping
56898	Yunnan Eshan
56944	Yunnan Cangyuan
56946	Yunnan Gengma
56948	Yunnan Ximeng
56949	Yunnan Menglian
56950	Yunnan Shuangjiang
56951	Yunnan Lincang
56952	Yunnan Jinggu
56954	Yunnan Lancang
56958	Yunnan Menghai
56962	Yunnan Mojiang
56964	Yunnan Simao
56966	Yunnan Yuanjiang
56969	Yunnan Mengla
56970	Yunnan Shiping
56976	Yunnan Yuanyang
56977	Yunnan Jiangcheng
56978	Yunnan Luchun
56982	Yunnan Kaiyuan
56984	Yunnan Gejiu
56987	Yunnan Jinping
56991	Yunnan Yanshan
56992	Yunnan Xichou
56994	Yunnan Wenshan
59007	Yunnan Guangnan
59205	Yunnan Funing
58443	Zhejiang Changxing
58446	Zhejiang Anji
58448	Zhejiang Linan
58449	Zhejiang Fuyang
58450	Zhejiang Huzhou
58451	Zhejiang Jiashan
58452	Zhejiang Jiaxing
58453	Zhejiang Shaoxing
58454	Zhejiang Deqing
58457	Zhejiang Hangzhou
58467	Zhejiang Cixi

58472	Zhejiang Shengsi
58484	Zhejiang Daishan
58537	Zhejiang Kaihua
58542	Zhejiang Tonglu
58544	Zhejiang Jiande
58546	Zhejiang Pujiang
58547	Zhejiang Longyou
58549	Zhejiang Jinhua
58550	Zhejiang Zhuji
58555	Zhejiang Xinchang
58556	Zhejiang Shengzhou
58558	Zhejiang Dongyang
58559	Zhejiang Tiantai
58560	Zhejiang Panan
58561	Zhejiang Zhenhai
58562	Zhejiang Yinzhou
58565	Zhejiang Fenghua
58566	Zhejiang Xiangshan
58567	Zhejiang Ninghai
58568	Zhejiang Sanmen
58569	Zhejiang Shipu
58570	Zhejiang Putuo
58631	Zhejiang Changshan
58632	Zhejiang Jiangshan
58642	Zhejiang Wuyi
58643	Zhejiang Yongkang
58644	Zhejiang Suichang
58646	Zhejiang Lishui
58647	Zhejiang Longquan
58652	Zhejiang Xianju
58654	Zhejiang Jinyun
58656	Zhejiang Leqing
58657	Zhejiang Qingtian
58658	Zhejiang Yongjia
58660	Zhejiang Linhai
58664	Zhejiang Wenling
58665	Zhejiang Hongjia
58666	Zhejiang Dachen
58742	Zhejiang Yunhe
58746	Zhejiang Taishun
58750	Zhejiang Wencheng
58751	Zhejiang Pingyang
58648	Zhejiang Jingning

57333	Chongqing Chengkou
57338	Chongqing Kaixian
57339	Chongqing Yunyang
57345	Chongqing Wuxi
57349	Chongqing Wushan
57409	Chongqing Tongnan
57425	Chongqing Dianjiang
57432	Chongqing Wanzhou
57437	Chongqing Zhongxian
57438	Chongqing Shizhu
57502	Chongqing Dazu
57505	Chongqing Rongchang
57506	Chongqing Yongchuan
57509	Chongqing Wansheng
57510	Chongqing Tongliang
57511	Chongqing Beibei
57513	Chongqing Yubei
57514	Chongqing Bishan
57517	Chongqing Jiangjin
57518	Chongqing Banan
57519	Chongqing Nanchuan
57520	Chongqing Changshou
57523	Chongqing Fengdu
57525	Chongqing Wulong
57536	Chongqing Qianjiang
57537	Chongqing Pengshui
57612	Chongqing Qijiang

Table S8. Isolated symbols at bottom in Figure 1

54471	Liaoning Yingkou	51704	Xinjiang Atushi	53756	Shaanxi Wubao
56434	Tibet Chayu	56688	Yunnan Dongchuan	50978	Heilongjiang Jixi
50987	Heilongjiang Jidong	54284	Jilin Donggang	53857	Shaanxi Yichuan
51076	Xinjiang Aletai	54453	Liaoning Lianshan	54523	Tianjin Wuqing
54041	Jilin Tongyu	54584	Liaoning Zhuanghe	54420	Hebei Luanping
54424	Beijing Pinggu	50659	Heilongjiang Kedong	54645	Tianjin Dagang

Table S9. Isolated symbols at bottom in Figure 2

51133	Xinjiang Tacheng	54499	Beijing Changping	54424	Beijing Pinggu
54399	Beijing Haidian	54431	Beijing Tongzhou	54645	Tianjin Dagang
56434	Tibet Chayu	50987	Heilongjiang Jidong		

Table S10. Isolated symbols at bottom in Figure 3

51628	Xinjiang Akesu
51704	Xinjiang Atushi
53618	Ningxia Yongning
53953	Shanxi Xiangning
58022	Shandong Yicheng
58664	Zhejiang Wenling
53688	Hebei Xingtang
58269	Jiangsu Qidong
58361	Shanghai Minhang
54539	Hebei Leting
53567	Shaanxi Fugu
53487	Shanxi Datong
57339	Chongqing Yunyang
53469	Inner Mongolia Helingeerxian
57081	Henan Xingyang
57510	Chongqing Tongliang
52876	Qinghai Minhe
53610	Ningxia Helan
53564	Shanxi Hequ
57887	Hunan Yongxing
53481	Inner Mongolia Chayouqianqi
58606	Jiangxi Nanchang
58705	Jiangxi Yongfeng
53972	Henan Qinyang
58362	Shanghai Baoshan
53576	Shanxi Shanyin
54142	Jilin Shuangliao
53519	Ningxia Huinong
59758	Hainan Haikou
59945	Hainan Baoting
59313	Guangdong Raoping
53615	Ningxia Taole
51368	Xinjiang Changji
54286	Jilin Helong
53773	Hebei Linzhang
53464	Inner Mongolia Tumutezuoqi
53466	Inner Mongolia Huhehaote suburb
59113	Fujian Yongding
58463	Shanghai Fengxian
57173	Henan Lushan

52884	Gansu Gaolan
53480	Inner Mongolia Jining
53517	Ningxia Shitanjing
53982	Henan Jiaozuo
56493	Sichuan Nanxi
57187	Henan Sheqi
51377	Xinjiang Fukang
58453	Zhejiang Shaoxing
58457	Zhejiang Hangzhou
58744	Fujian Shouning
58750	Zhejiang Wencheng
53983	Henan Fengqiu
57082	Henan Dengfeng
56835	Yunnan Longchuan
58615	Jiangxi Wannian
50878	Heilongjiang Huachuan
57042	Shaanxi Fuping
54523	Tianjin Wuqing
54623	Tianjin Tanggu
57859	Guangxi Ziyuan
54927	Shandong Tengzhou
53949	Shaanxi Chengcheng
53680	Hebei Lingshou
57537	Chongqing Pengshui
57612	Chongqing Qijiang
53611	Ningxia Pingluo
53878	Shanxi Licheng
58002	Shandong Caoxian
58450	Zhejiang Huzhou
58706	Jiangxi Lean

Table S11. Isolated symbols at bottom in Figure 4

53878	Shanxi Licheng
53973	Shanxi Gaoping
57509	Chongqing Wansheng
53463	Inner Mongolia Huhehaote
58003	Shandong Chengwu
57345	Chongqing Wuxi
54304	Hebei Chongli
54535	Hebei Caofeidian
59287	Guangdong Guangzhou
53553	Inner Mongolia Zhungeerqi
58362	Shanghai Baoshan
53953	Shanxi Xiangning
54080	Heilongjiang Wuchang
54320	Inner Mongolia Ningchengxian
53781	Hebei Shahe
53938	Shaanxi Xunyi
58361	Shanghai Minhang
58501	Hubei Wuxue
53519	Ningxia Huinong
58021	Shandong Xuechen
56189	Sichuan Pengzhou
58926	Fujian Zhangping
54716	Shandong Ningjin
58022	Shandong Yicheng
57271	Henan Xinye
54186	Jilin Dunhua
53688	Hebei Xingtang
53925	Gansu Zhenyuan
54523	Tianjin Wuqing
58247	Jiangsu Yangzhong
54909	Shandong Dingtao
53866	Shanxi Hongtong
54623	Tianjin Tanggu
57740	Hunan Fenghuang
57845	Hunan Tongtao
52313	Xinjiang Hongliuhe
57954	Guangxi Lingui
54916	Shandong Yanzhou
58457	Zhejiang Hangzhou
58269	Jiangsu Qidong

58463	Shanghai Fengxian
50934	Inner Mongolia Tuquan
54945	Shandong Rizhao
58484	Zhejiang Daishan
54340	Liaoning Sujiatun
54927	Shandong Tengzhou
53892	Hebei Handan

Table S12. Isolated symbols at bottom in Figure 7

50603	Inner Mongolia Xinbaerhuyouqi
52602	Qinghai Lenghu
56116	Tibet Dingqing
51482	Xinjiang Mulei
52118	Xinjiang Yiwu
50639	Inner Mongolia Zhalantun
56016	Qinghai Zhiduo
56223	Tibet Luolong
53433	Inner Mongolia Wulateqianqi
52313	Xinjiang Hongliuhe
50727	Inner Mongolia Aershan
56018	Qinghai Zaduo
56227	Tibet Bomi
51572	Xinjiang Tulufandongkan
56444	Yunnan Deqin
50924	Inner Mongolia Houlinguole
56144	Sichuan Dege
56331	Tibet Zuogong
51639	Xinjiang Shaya
58472	Zhejiang Shengsi
59754	Guangdong Xuwen
55248	Tibet Gaize
51156	Xinjiang Hebukesaier
51765	Xinjiang Tieqianlike
52515	Gansu Subei
51241	Xinjiang Touli
55437	Tibet Pulan
53231	Inner Mongolia Hailisu
51839	Xinjiang Minfeng
51468	Xinjiang Tianshandaxigou
55664	Tibet Dingri
51470	Xinjiang Tianchi
51931	Xinjiang Yutian

Table S13. Isolated symbols at bottom in Figure 8

50434	Inner Mongolia Tulihe
53433	Inner Mongolia Wulateqianqi
55437	Tibet Pulan
51468	Xinjiang Tianshandaxigou
51765	Xinjiang Tieqianlike
52515	Gansu Subei
50603	Inner Mongolia Xinbaerhuyouqi
52602	Qinghai Lenghu
55664	Tibet Dingri
53231	Inner Mongolia Hailisu
51839	Xinjiang Minfeng
50639	Inner Mongolia Zhalantun
56016	Qinghai Zhiduo
56116	Tibet Dingqing
51470	Xinjiang Tianchi
51931	Xinjiang Yutian
50913	Inner Mongolia Wulagai
56018	Qinghai Zaduo
56223	Tibet Luolong
51482	Xinjiang Mulei
52118	Xinjiang Yiwu
59754	Guangdong Xuwen
56144	Sichuan Dege
56227	Tibet Bomi
51572	Xinjiang Tulufandongkan
52313	Xinjiang Hongliuhe
56331	Tibet Zuogong
56147	Sichuan Baiyu
50924	Inner Mongolia Houlinguole
51639	Xinjiang Shaya
56444	Yunnan Deqin
51156	Xinjiang Hebukesaier
55248	Tibet Gaize
51241	Xinjiang Touli
51722	Xinjiang Awati
58472	Zhejiang Shengsi
